# Magnesium hydride alkene insertion and catalytic hydrosilylation†
†Electronic supplementary information (ESI) available: Full experimental details and ^1^H and ^13^C{^1^H} NMR spectra. Details of the X-ray analysis of compounds **12** and **15**. Details of the computational analysis. CCDC [1912200 and 1912201]. For ESI and crystallographic data in CIF or other electronic format see DOI: 10.1039/c9sc02056j


**DOI:** 10.1039/c9sc02056j

**Published:** 2019-07-22

**Authors:** Lucia Garcia, Chiara Dinoi, Mary F. Mahon, Laurent Maron, Michael S. Hill

**Affiliations:** a Department of Chemistry , University of Bath , Claverton Down , Bath , BA2 7AY , UK . Email: msh27@bath.ac.uk; b Université de Toulouse et CNRS , INSA , UPS , UMR 5215 , LPCNO , 135 Avenue de Rangueil , F-31077 Toulouse , France

## Abstract

The dimeric β-diketiminato magnesium hydride, [(BDI)MgH]_2_, reacts at 80 °C with the terminal alkenes, 1-hexene, 1-octene, 3-phenyl-1-propene and 3,3-dimethyl-butene to provide the respective *n*-hexyl, *n*-octyl, 3-phenylpropyl and 3,3-dimethyl-butyl magnesium organometallics. The facility for and the regiodiscrimination of these reactions are profoundly affected by the steric demands of the alkene reagent. Reactions with the phenyl-substituted alkenes, styrene and 1,1-diphenylethene, require a more elevated temperature of 100 °C with styrene providing a mixture of the 2-phenylethyl and 1-phenylethyl products over 7 days. Although the reaction with 1,1-diphenylethene yields the magnesium 1,1-diphenylethyl derivative as the sole reaction product, only 64% conversion was achieved over a 21 day timeframe. Reactions with the α,ω-dienes, 1,5-hexadiene and 1,7-octadiene, provided divergent results. The initial 5-alkenyl magnesium reaction product of the shorter chain diene undergoes 5-*exo*-trig cyclisation *via* intramolecular carbomagnesiation to provide a cyclopentylmethyl derivative, which was shown by X-ray diffraction analysis to exist as a three-coordinate monomer. In contrast, 1,7-octadiene provided a mixture of two compounds, a magnesium oct-7-en-1-yl derivative and a dimagnesium-octane-1,4-diide, as a result of single or two-fold activation of the terminal C=C double bonds. The magnesium hydride was unreactive towards internal alkenes apart from the strained bicycle, norbornene, allowing the characterisation of the resultant three-coordinate magnesium norbornyl derivative by X-ray diffraction analysis. Computational analysis of the reaction between [(BDI)MgH]_2_ and 1-hexene using density functional theory (DFT) indicated that the initial Mg–H/C=C insertion process is rate determining and takes place at the intact magnesium hydride dimer. This exothermic reaction (Δ*H* = –14.1 kcal mol^–1^) traverses a barrier of 18.9 kcal mol^–1^ and results in the rupture of the dinuclear structure into magnesium alkyl and hydride species. Although the latter three-coordinate hydride derivative may be prone to redimerisation, it can also provide a further pathway to magnesium alkyl species through its direct reaction with a further equivalent of 1-hexene, which occurs *via* a lower barrier of 15.1 kcal mol^–1^. This Mg–H/C=C insertion reactivity provides the basis for the catalytic hydrosilylation of terminal alkenes with PhSiH_3_, which proceeds with a preference for the formation of the anti-Markovnikov organosilane product. Further DFT calculations reveal that the catalytic reaction is predicated on a sequence of Mg–H/C=C insertion and classical Si–H/Mg–C σ-bond metathesis reactions, the latter of which, with a barrier height of 24.9 kcal mol^–1^, is found to be rate determining.

## Abstract 2

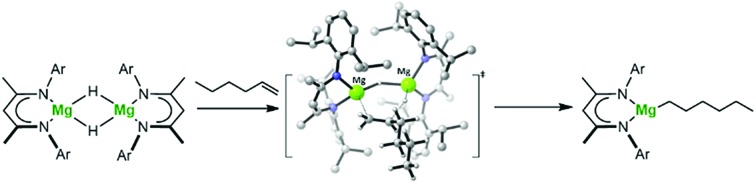
The β-diketiminato magnesium hydride, [(BDI)MgH]]_2_, reacts with alkenes and catalyses their hydrosilylation with PhSiH_3_.

## Introduction

**Scheme 1:**
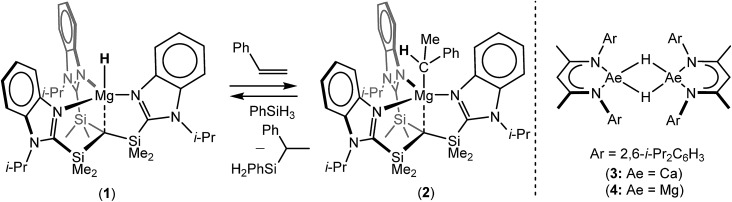
The reactivity of compounds **1** and **2** and the structures of compounds **3** and **4**.

During the 120 years since their initial description, Grignard's eponymous reagents have provided one of the foundation stones of organic and organometallic chemistry.^1^ Despite their utility, the synthesis of Grignard reagents and, more generally, organomagnesium compounds remains almost exclusively dependent on the ‘direct reaction’ of an organohalide with elemental magnesium. These reactions are also necessarily performed in ethereal solvents and alternative general synthetic methods for the formation of Mg–C bonds are something of a rarity.^2^ The successful hydridomagnesiation of alkenes, therefore, would present an attractive but, as yet, underexploited route to Mg–C bond formation, particularly in cases when the use of ether solvents or the presence of residual halide is disadvantageous. Although the transition metal promoted addition of alkenes to MgH_2_ has been described,^3^–^9^ and a significant number of well-defined Mg–H bonded species have now been reported,^10^ the first fully authenticated example of the direct insertion of an olefin into the Mg–H bond of any molecular compound was only provided by Parkin and co-workers in 2017.^11^ In this case, reaction of the terminal magnesium hydride derivative, [Tism^i-PrBenz^]MgH (**1**, Tism^i-PrBenz^ = tris-[(1-isopropylbenzimidazol-2-yl)-dimethylsilyl]methyl), with styrene ensued with the formation of an isolable 1-phenylethyl derivative, [Tism^i-PrBenz^]MgCH(Me)Ph (**2**) (Scheme 1). Compound **2** was also shown to react with PhSiH_3_ to provide PhC(SiH_2_Ph)HMe and reform compound **1**,^11^ a reaction sequence that also provided a basis for the catalytic Markovnikov hydrosilylation of styrene.

It has previously been shown that the β-diketiminato calcium hydride, [(BDI)CaH]_2_ (**3**; BDI = HC{CMe_2_NDipp}_2_; Dipp = 2,6-i-Pr_2_C_6_H_3_), reacts as a dimer *via* highly polarised pathways, even with unactivated terminal alkenes, to provide exceptionally potent calcium alkyl nucleophiles, [(BDI)CaR]_2_.^12^–^15^ In related observations, Harder and co-workers have also very recently divulged that similar alkene insertion reactivity may be achieved with β-diketiminato hydride derivatives of calcium's heavier congener, strontium.^16^ In contrast, and despite its heavy use to effect the reductive hydroboration or hydrosilylation of an array of polar C=E (E = O,^17^,^18^ NR^19^–^22^) and C≡E (E = O,^23^,^24^ N,^25^ NR^26^) bonded substrates, no analogous reactivity between Jones' lighter, but similarly dimeric, magnesium hydride, [(BDI)MgH]_2_ (**4**),^8^,^27^ with any C=C bonded small molecule appears to have been described.^28^

**Scheme 2:**
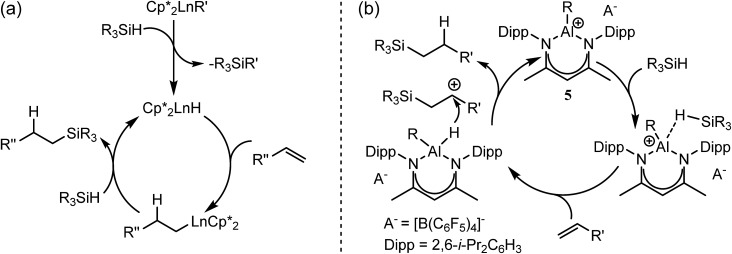
Schematic alkene hydrosilylation mechanisms deduced for redox inactive catalysts derived from (a) organolanthanides; (b) the Lewis acid p-block centre of **5**.^50^

Notwithstanding impressive recent advances in first row transition metal chemistry,^29^–^31^ the catalytic hydrosilylation of alkenes remains the preserve of precious late transition metals.^32^–^37^ Organolanthanide complexes have also been known to enable the addition of Si–H bonds to terminal alkenes since the early 1990s.^37^ In these cases, the mechanistic process is based on the conversion of an organometallic pre-catalyst into a catalytic hydride by σ-bond metathesis and a subsequent sequence of polarised alkene insertion and Si–H/Ln–C metathesis reactions (Scheme 2a).

In contrast to the relative maturity of these advances, the implementation of main group derivatives for catalytic hydrosilylation is much less developed. Several systems derived from p-block electrophiles such as AlCl_3_,^38^–^43^ B(C_6_F_5_)_3_,^44^,^45^ the silylium cation, [Et_3_Si(C_6_H_6_)][B(C_6_F_5_)_3_],^46^ and the phosphonium derivative [(SIMes)PFPh_2_][B(C_6_F_5_)_4_]^–^ (SIMes = 1,3-dimesitylimidazolidin-2-ylidene)^47^,^48^ have, however, been described. In such cases, the hydrosilylation reaction has been proposed to take place *via* silane hydride abstraction by the potent Lewis acid centre and sequential delivery of the silylium cation and hydride to the C=C double bond.^49^ Of most relevance to the current research, Nikonov and co-workers have reported the cationic hydrido aluminium β-diketiminate derivative [(BDI)AlH]^+^[B(C_6_F_5_)_4_]^–^ (**5**) as a catalyst for the hydrosilylation of alkenes and alkynes.^50^ Notably, the cationic component of **5** is strictly isoelectronic to a monomeric unit of **4** and, although the exact details could not be elucidated, the authors suggested that the most likely mechanism involves alkene attack on an incipient silylium ion generated through hydride abstraction by the highly Lewis acidic aluminium cation (Scheme 2b).

Hydrosilylation mediated by s-block centres was pioneered by Harder and co-workers who employed highly polar potassium, calcium and strontium benzyl species as pre-catalysts for the silane reduction of conjugated 1,1-diphenylethylene, styrene and diene substrates.^51^ The observation of anti-Markovnikov alongside the expected Markovnikov products was rationalised as a consequence of polarised 1,1-C=C insertion into the M–Si bond of a metal silanide, which is apparently formed by M–C/silane metathesis in competition with the expected hydride intermediates. This supposition was vindicated by Okuda's subsequent development of several group 1 and calcium silanide and hydridosilicate species, which also yield the anti-Markovnikov products for the hydrosilylation of 1,1-diphenylethylene and similarly activated alkenes.^52^–^54^ Although this s-block-catalysed alkene hydrosilylation is currently limited to a very narrow range of conjugated substrates, recent observations have established that heavier alkaline earth (Ca, Sr, Ba) hydrides (*e.g.***3**) may be converted to *n*-alkyl species by their reaction with unactivated alkenes.^12^,^15^,^16^,^55^,^56^ In this joint synthetic and computational study, we demonstrate that terminal alkenes and unhindered alkynes also react directly with compound **4** and that subsequent silane metathesis of the resultant organomagnesium species provides a basis for the hydrosilylation of C–C unsaturated substrates.

## Results and discussion

### Stoichiometric reactions of compound **4** with alkenes

**Scheme 3:**
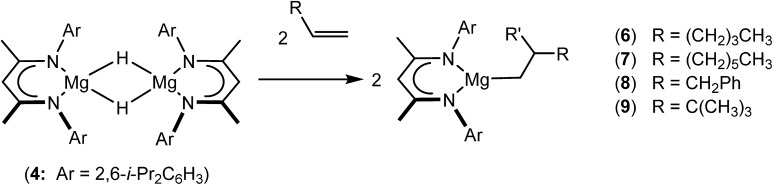
Synthesis of the magnesium alkyl compounds **6–9**.

An initial reaction was performed between the dimeric magnesium hydride (**4**) and two molar equivalents of 1-hexene at 80 °C. Monitoring by ^1^H NMR spectroscopy over a period of 4 hours evidenced complete consumption of the starting materials and the production of a single new base-free β-diketiminato *n*-hexyl magnesium derivative (**6**; Scheme 3). The emergence of compound **6** was signified by the appearance of a high field magnesium-bound α-methylene triplet signal at *δ* –0.25 ppm. Compound **6** was readily isolated in analytically pure form as a colourless solid, samples of which provided ^1^H and ^13^C{^1^H} NMR spectra that were entirely consistent with the anticipated formulation. Although a number of methyl- and *n*-butyl magnesium β-diketiminate derivatives have been reported, all prior syntheses of these compounds have been dependent upon either deprotonation of the diimine ligand precursor with a diorganomagnesium or salt metathesis between the lithiated β-diketiminate and a Grignard reagent.^57^–^66^

The successful synthesis of compound **6** prompted extension of this reactivity to several further terminal alkenes (Scheme 3). Although reaction of 1-octene at 80 °C also proceeded to completion within an analogous 4 hour time period to provide the magnesium *n*-octyl complex (**7**), similar thermal treatment of both 3-phenyl-1-propene and 3,3-dimethyl-1-butene indicated that the facility of these C=C insertion reactions is significantly perturbed by the relative steric demands of the alkene substrate. In these latter cases, reactions at 80 °C between compound **4** and the unsaturated organic reagents required 2 days and 21 days to achieve complete conversion to the respective magnesium 3-phenyl-propyl (**8**) and 3,3-dimethyl-1-butyl (**9**) complexes, which were nevertheless isolated in analytically pure form.

These observations were underscored by the reactions of **4** with both styrene and 1,1-diphenylethene. Whereas the terminal hydride derivative **1** was reported to react readily with styrene within 2 hours at room temperature to yield compound **2** (Scheme 1),^11^ a similar reaction performed with compound **4** required 7 days at 100 °C to achieve complete consumption of the hydride reagent. This reaction was also found to be significantly less discriminating and provided approximately equal quantities of both potential regioisomers, the β-diketiminato magnesium 2-phenylethyl (**10A**) and 1-phenylethyl (**10B**) complexes, resulting from either 1,2- or 2,1-C=C insertion, respectively. This noteworthy kinetic effect was exacerbated by the introduction of additional phenyl substitution. Despite benefitting from the benzhydrylic substitution pattern that has previously been observed to greatly facilitate analogous reactions with molecular calcium hydrides,^13^,^14^,^51^,^55^,^56^,^67^,^68^ 1,1-diphenylethene was found to react only very sluggishly with compound **4**, requiring 6 weeks at 100 °C to achieve 64% conversion to the 1,1-diphenylethyl complex (**11**). Although pure bulk samples of compounds **10A**/**B** and **11** could not be isolated, the structures of these organomagnesium derivatives were assigned with a high degree of certainty by *in situ* studies of the two reactions by ^1^H and ^13^C{^1^H} NMR spectroscopy.

**Scheme 4:**
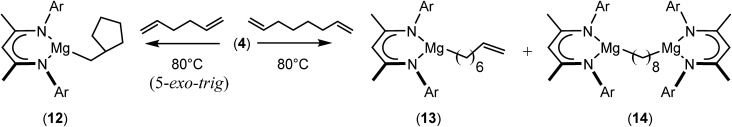
Synthesis of compounds **12–14**.

**Fig. 1:**
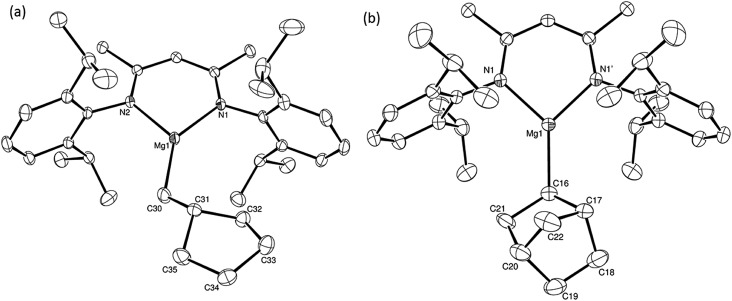
ORTEP representation of (a) compound **12** [one of the 2 molecules in the asymmetric unit] and (b) compound **15** (30% probability ellipsoids). Hydrogen atoms have been omitted for clarity throughout and, for similar reasons, only one component is shown where disorder has been modelled. Selected bond lengths (Å) and angles (°) (**12**) Mg1–N1 2.0122(14), Mg1–N2 2.0104(14), Mg1–C30 2.0987(19), C30–C31 1.516(3), N2–Mg1–N1 93.38(6), N1–Mg1–C30 140.54(7), N2–Mg1–C30 126.07(7); (**15**) Mg1–N1 2.0128(12), Mg1–C16 2.096(2), N1–Mg1–N1' 93.45(7), N1–Mg1–C16 133.25(4), N11–Mg1–C16 133.25(4). Symmetry operation to generate the primed atom 1 + *x*, 3/2 – *y*, +*z*.

We have recently reported that reactions of the calcium hydride (**3**) with both 1,5-hexadiene and 1,7-octadiene proceed *via* initial formation of the respective open chain 5-alkenyl and 7-en-1-yl derivatives. The shorter chain species is rapidly consumed, however, through an intramolecular carbocalciation reaction to provide an isolable calcium cyclopentylmethyl derivative.^13^,^14^ A reaction between 1,5-hexadiene and compound **4** at 80 °C similarly resulted in complete and selective conversion to a single new compound, which was identified as the magnesium cyclopentylmethyl derivative (**12**) resulting from facile 5-*exo-trig* cyclization (Scheme 4). The formation of compound **12** was readily established by the emergence of new BDI methine and upfield methylene doublet signals with relative intensities of 1 : 2 at *δ* 4.72 and –0.07 ppm, respectively, while its solid state structure was confirmed through an X-ray diffraction analysis performed on single crystals isolated from *n*-hexane solution. Although the addition of Grignard reagents to alkenes and similar intramolecular cyclisations of unsaturated organomagnesium reagents have been studied for over 50 years,^69^ compound **12** appears to provide the first example of a resultantly cyclised organomagnesium to be structurally characterised. The results of this analysis are shown in Fig. 1a, which confirms the outcome of the ring closure process and the identity of compound **12** as a mononuclear species comprising a three-coordinate magnesium centre.

A similar reaction performed between compound **4** and 1,7-octadiene, provided no indication for significantly less favorable 7-*exo-trig* or 8-*endo-trig* ring closure (Scheme 4). Rather, a mixture of two new compounds, the oct-7-en-1-yl derivative (**13**) and the dimagnesio-octane-1,4-diide (**14**) were formed in effectively equimolar quantities through complete consumption of the hydride reagent at 80 °C over the course of 5 hours. Although compounds **13** and **14** proved to be inseparable, irrespective of variations in the reaction stoichiometry, the compounds could be readily discriminated by both ^1^H and ^13^C NMR spectroscopy.

Reminiscent of the reactivity of the calcium hydride (**3**),^14^ compound **4** was found to be completely unreactive towards the internal alkenes, 2,3-dimethyl-2-butene, cyclopentene and cyclohexene but to react smoothly, albeit slowly, with both the strained bicyclic alkene, norbornene (3 days at 80 °C) and the internal alkyne, diphenylacetylene (6 days at 80 °C), to provide the magnesium norbornyl (**15**) and (*E*)-(1,2-diphenylvinyl) (**16**) derivatives. Although monitoring of the reaction with norbornene revealed tentative evidence that the production of **15** occurs *via* the formation of a dinuclear hydridonorbornyl-dimagnesium intermediate (Fig. S22†) analogous to the crystallographically characterised product of the reaction between the calcium hydride (**3**) and norbornene,^14^ this species could not be isolated for definitive characterisation. The ultimate outcome of the previously described reaction was also limited by facile intramolecular C–H activation of a BDI isopropyl substituent at more elevated temperatures. In contrast, the magnesium norbornyl derivative (**15**) was found to be thermally stable allowing its isolation in high (>90%) yield. The formation of compound **15** was signified in its ^1^H NMR spectrum by a new BDI methine singlet at *δ* 4.93 ppm and a characteristic upfield doublet of doublets of doublets signal at *δ* –1.31 ppm, which emerged in a 1 : 1 ratio by integration. The constitution of compound **16** was readily established by ^1^H NMR spectroscopy through the appearance of new BDI methine and vinylic C–H singlet resonances, each of which developed simultaneously and with identical 1H integrals at *δ* 4.93 and 5.85 ppm. Compound **15** was also characterised by single crystal X-ray diffraction analysis (Fig. 1b), which confirmed its solid state constitution as a further three-coordinate magnesium organometallic. Although compound **15** appears to be the first norbornylmagnesium derivative to be structurally characterised, its structure is otherwise unremarkable and the relevant Mg–N and Mg–C distances are closely comparable with those of compound **12** despite its secondary alkyl character.

**Fig. 2:**
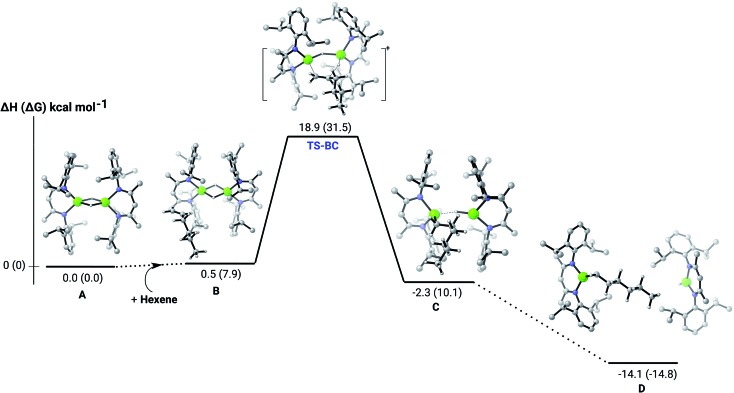
DFT (B3PW91) computed enthalpy reaction profile (corresponding free energies are shown in parenthesis) at room temperature for the first Mg–H/C=C insertion of 1-hexene into the dinuclear magnesium hydride **4**(**A**).

**Fig. 3:**
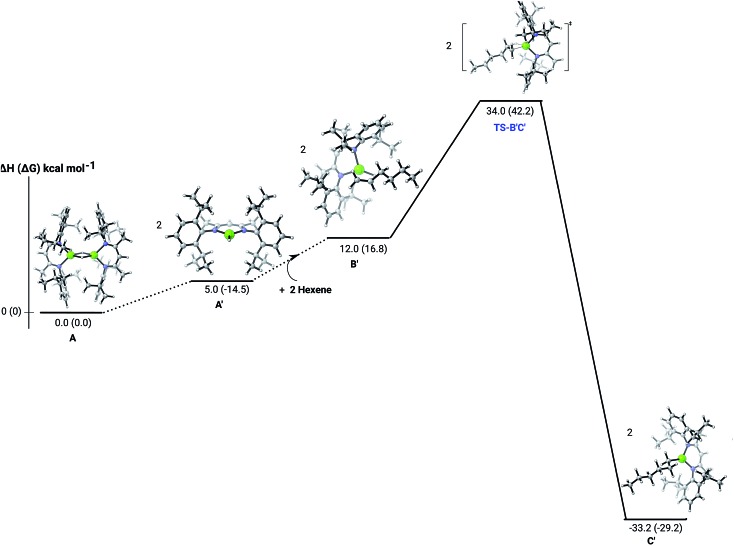
DFT (B3PW91) computed enthalpy reaction profile (corresponding free energies are shown in parenthesis) at room temperature for the first Mg–H/C=C insertion of 1-hexene into the mononuclear hydride **B′**.

**Fig. 4:**
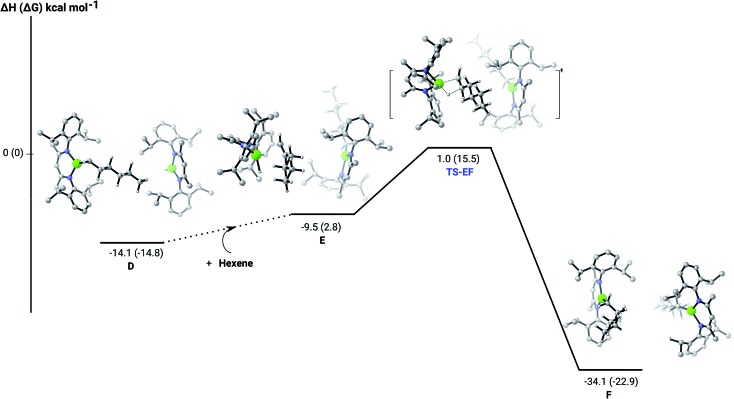
DFT (B3PW91) computed enthalpy reaction profile (corresponding free energies are shown in parenthesis) at room temperature for the second Mg–H/C=C 1-hexene insertion into the mononuclear magnesium hydride monomer (**D**).

**Fig. 5:**
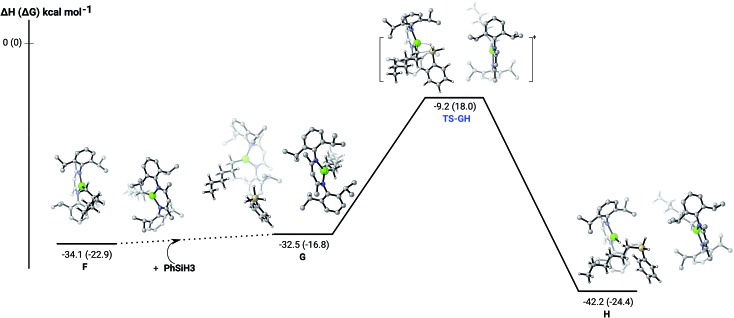
DFT (B3PW91) computed enthalpy reaction profile (corresponding free energies are shown in parenthesis) at room temperature for the Mg–C/Si–H metathesis starting from complex **F**.

Further insight into the Mg–H/C=C insertion mechanism was provided by density functional theory (DFT) calculations carried out with the same computational approach (B3PW91, see ESI†) previously used to describe the stepwise formation and further hydrogenation of the dicalcium di-*n*-hexyl derivatives.^12^–^14^ Although the relevant free energies are also presented (shown in parenthesis in Fig. 2–5), we limit our discussion to changes in enthalpy due to potential entropic errors introduced by adjustments to species molecularity during the reactions. The computed Mg–H/C=C insertion profile shown in Fig. 2 indicates that the first exothermic (Δ*H* = –14.1 kcal mol^–1^) 1-hexene insertion takes place into the dimeric magnesium hydride, **4**(**A**), *via* an accessible barrier of 18.9 kcal mol^–1^. For comparison, we also computed an initial insertion step on the mononuclear magnesium compound (Fig. 3), that is not kinetically competitive. Although the initial insertion step can occur without any necessary rupture of the dimeric magnesium hydride, dissociation of the resultant dimagnesium alkyl–hydrido complex (**C**) to form a proximal but non-bonding pair of dissimilar three-coordinate magnesium alkyl and hydrido complexes (**D**) is significantly exothermic (Δ*H* = –11.8 kcal mol^–1^ with respect to **C**). Although the hydridic component may redimerise to **4**(**A**), the low coordinate magnesium hydride also holds the potential to undergo a second 1-hexene insertion *via* an accessible barrier of 15.1 kcal mol^–1^ (Fig. 4). This value is lower by 5.9 and 3.8 kcal mol^–1^ than both of those computed for the first C=C insertion at **4**(**A**) and the three-coordinate hydride generated by its monomerisation, respectively. Although this latter observation appears somewhat surprising, it is possibly ascribed to a stabilising interaction between the hexyl and hydrido complexes at the transition state (**TS-EF**). A transition state corresponding to the second 1-hexene insertion into the dinuclear magnesium hydride complex **C** could be also located (Fig. S36†), although its high barrier (Δ*H* = 29.2 kcal mol^–1^, **TS-C1C2**) led us to discard this possible pathway. The dimerisation of complex **F**, yielding complex **F1**, was computed to be endothermic by 17.0 kcal mol^–1^ allowing us to also discount this possibility. Consistent with the experimental results, therefore, the overall result of Mg–H/C=C insertion may be assessed to be a facile process affording three-coordinated, mononuclear alkyl species (**F**).

### Magnesium-catalysed hydrosilylation

Given the unexpectedly broad reactivity of compound **4** with C–C multiple bonds, we next turned our attention to its potential to mediate catalytic hydrosilylation of alkenyl substrates with phenylsilane. An initial reaction was performed in C_6_D_6_ between 1-hexene and PhSiH_3_ in the presence of 5 mol% **4**. Monitoring of the reaction performed at 60 °C by ^1^H NMR spectroscopy indicated that, although somewhat sluggish, the reaction proceeded with complete conversion to the product of *anti*-Markovnikov silane addition, *n*-hexyl(phenyl)silane, over the course of one week. An otherwise identical reaction performed at 80 °C provided similar observations but in the shorter timeframe of 4 days. We suggest that the observation of the *anti*-Markovnikov product is consistent with both the high regioselectivity of the stoichiometric insertion reaction to provide the terminal *n*-hexylmagnesium product (**6**) and the operation of a mechanism dependent upon a sequence of Mg–H/C=C insertion and Mg–C/Si–H metathesis events, which is broadly analogous to that envisioned for organolanthanide-based hydrosilylation catalysis (Scheme 2a, *vide infra*).

These deductions were borne out by a subsequent assessment of a series of catalytic hydrosilylation reactions performed with PhSiH_3_ and a range of alkene substrates (Table 1). Consistent with the observations outlined for 1-hexene (entry 1), hydrosilylation of 1-octene (entry 2) and 3,3-dimethyl-1-butene (entry 3) delivered the anti-Markovnikov products with absolute selectivity, albeit the latter reaction required 30 days to achieve similarly high conversions to the more sterically encumbered (3,3-dimethylbutyl)(phenyl)silane product. Analogous reactions performed with PhSiH_3_ and vinylsilanes (entries 4 and 5) were less successful, providing, at best, only stoichiometric (based on Mg) conversion to the unsymmetrical α,ω-disilane product for the Ph_3_Si-substituted substrate (entry 4) and no evidence of any reaction for Me_3_Si(CH=CH_2_) (entry 5). In line with the expectation provided by the synthesis of compound **8**, allylbenzene yielded, primarily, the anti-Markovnikov product (entry 6), while only the Markovnikov product was observed for the catalysis performed with 1,1-diphenylethene (entry 7). The observation of this latter product indicates that the C=C insertion reaction is likely to be dictated by similar stereoelectronic considerations to those operant during Parkin and co-workers synthesis of compound **2** (Scheme 1). In the current case, however, the rate of reaction was also significantly perturbed through the introduction of the terminal diphenyl substitution pattern such that the catalysis was found to be completely suppressed with a maximum conversion to the silane product of 22%, even after 30 days at 100 °C. Although the reaction between **4** and styrene was found to provide compounds **10A** and **10B** in approximately equal proportions, the catalytic reaction ensued with a significant (*ca.* 2 : 1) bias toward the production of the Markovnikov product, phenyl(1-phenylethyl)silane (entry 8).

**Table 1:** Catalytic hydrosilylation of alkenes mediated by compound **4** (5%, C_6_D_6_)

Entry	Substrate	Hydrosilylation product	*T* (°C)	*t* (days)	Conv. (%)
1	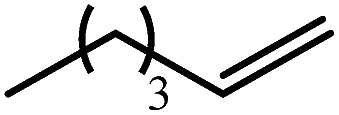	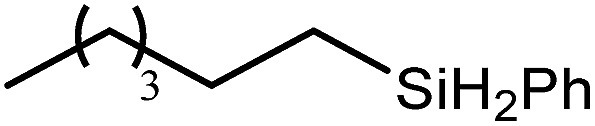	60	7	97
			80	4	99
2	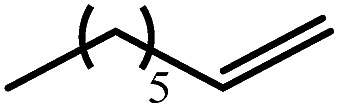	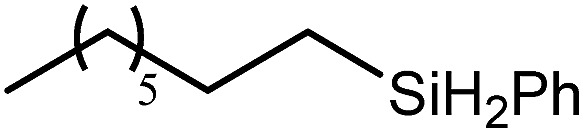	60	7	97
			80	4	99
3	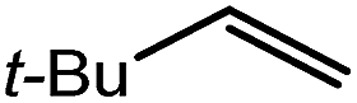	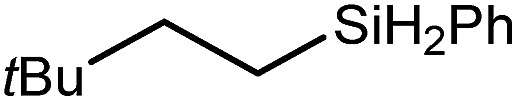	80	30	96
4	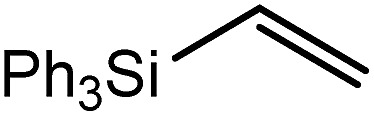	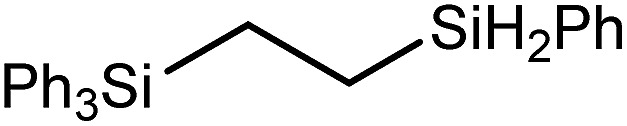	80	14	10
				30	10
5	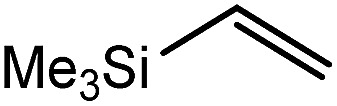	—	80	7	0
6	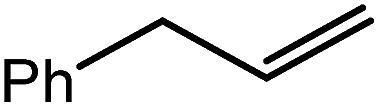	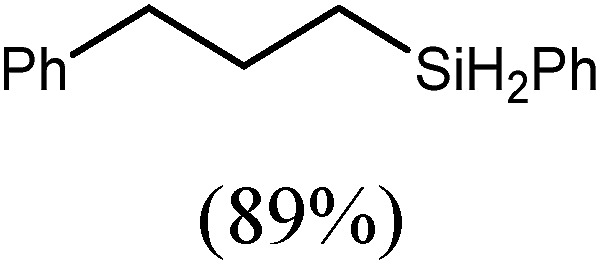	60	15	96
		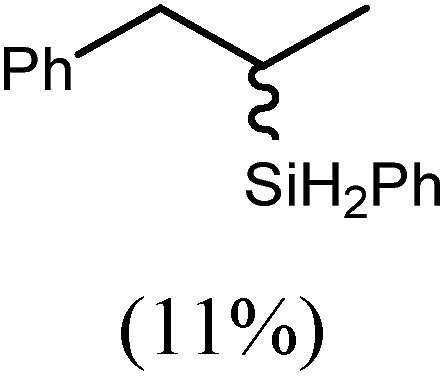	80	5	97
7	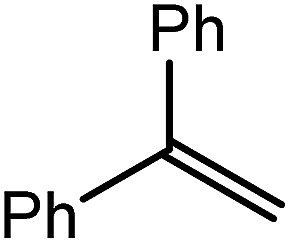	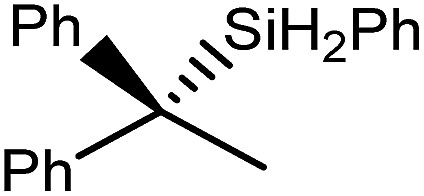	80	30	22
				44	22
8	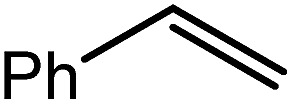	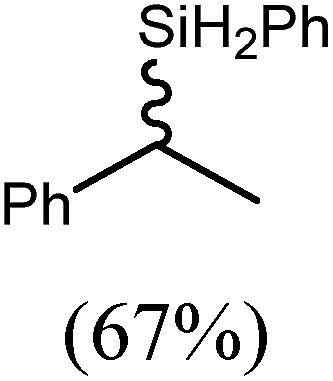	80	21	96
		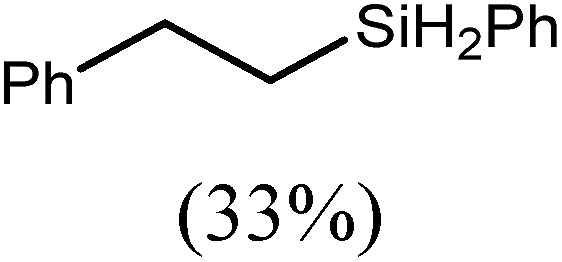			
9	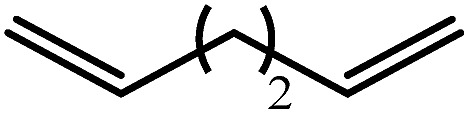	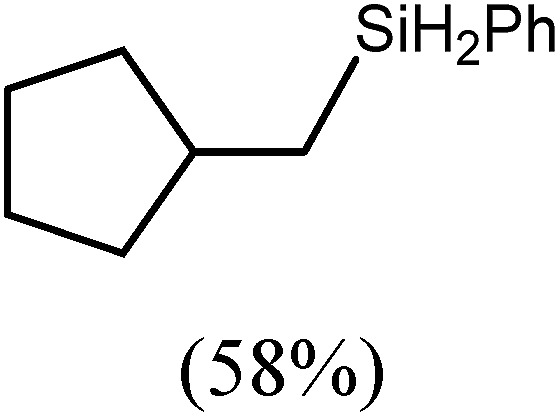	80	14	97
		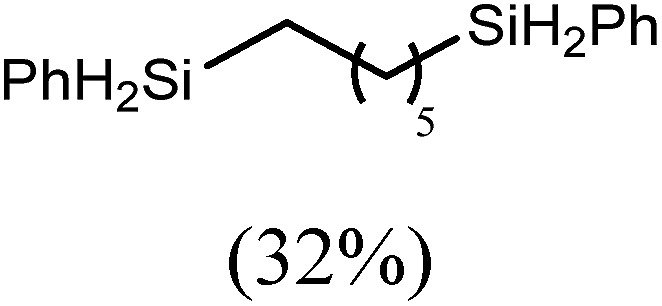			
		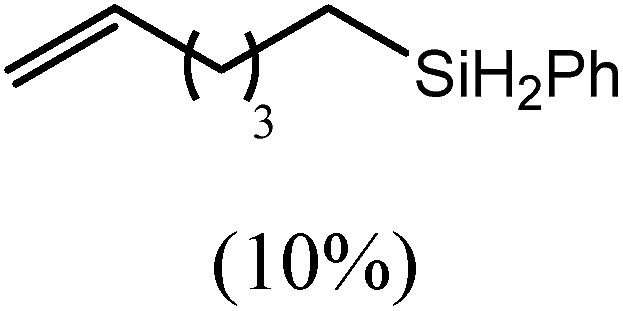			
10	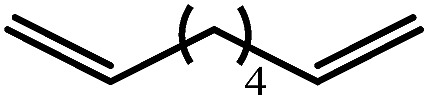	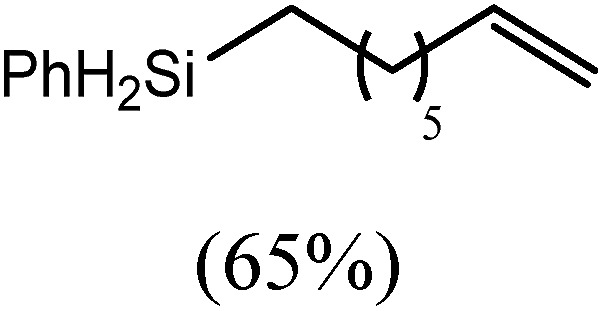	80	6	99
		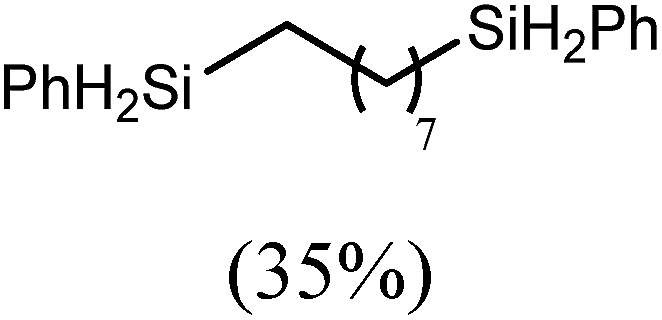			
11	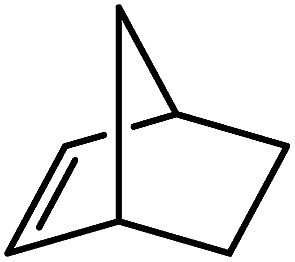	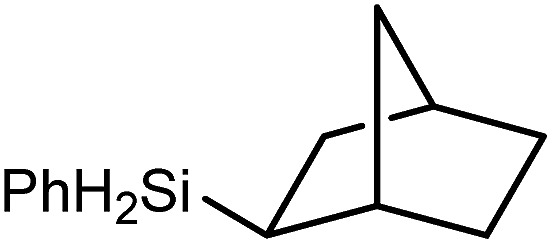	80	16	97
12	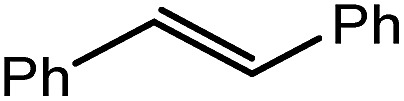	—	80	14	0
			100	28	0
13	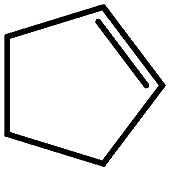	—	80	7	0
			100	4	0
14	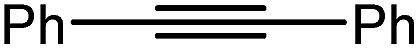	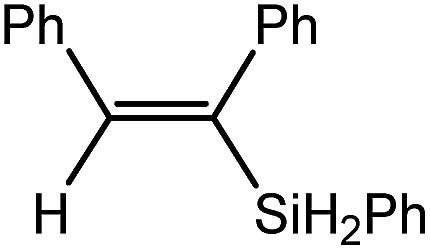	60	30	57
			80	30	95

The reaction of equimolar quantities of 1,5-hexadiene and PhSiH_3_ provided an approximate 1 : 3 : 6 distribution of the open chain alkenylsilane, the symmetrical α,ω-disilane and cyclised cyclopentylmethylsilane products (entry 9). Underlining the ease of formation of compound **12**, the preponderance of this latter compound suggests that intramolecular carbomagnesiation is competitive with Mg–C/Si–H metathesis under the conditions of the catalysis. Similarly, although dimagnesiation of 1,7-octadiene to form compound **14** was competitive with its monomagnesiation under stoichiometric conditions, the product of its monohydrosilylation was found to predominate under the conditions of the catalysis (entry 10).

Consistent with the successful synthesis of compound **15**, norbornene was the only internal alkene (entries 11–13) to undergo catalytic hydrosilylation in the presence of compound **4**, providing the racemic *exo*-2-(silyl)norbornane product. The likely operation of an insertion-metathesis mechanism similar to that depicted in Scheme 2a was also underscored by the successful hydrosilylation of diphenylacetylene (entry 14) to provide (*E*)-(1,2-diphenylvinyl)(phenyl)silane as the sole reaction product.

The hydrosilylation of 1-hexene in the presence of PhSiH_3_ was studied by density functional theory (DFT, B3PW91) calculations. Fig. 5 shows the silylation reaction subsequent to the second 1-hexene insertion, *i.e.* from complex **F**. The associated transition state displays a classical Mg–C/Si–H metathesis arrangement, *via* a barrier of 24.9 kcal mol^–1^ (**TS-GH**). In accordance with the experimental observations, the hydrosilylation step is the rate determining process affording, in the case of 1-hexene, the experimentally observed *anti*-Markovnikov *n*-hexyl(phenyl)silane product exclusively. For completeness, we also computed the hydrosilylation reaction subsequent to the first 1-hexene insertion from the *n*-hexyl hydrido complex **D** (Fig. S37†) and from the dimagnesium di-*n*-hexyl complex **F1** (Fig. S38†). Although both processes invoke similar Mg–C/Si–H metathesis *via* respective barriers of 25.0 kcal mol^–1^ (**TS-D1D2**) and 35.4 kcal mol^–1^ (**TS-F2F3**), the magnitude of (**TS-F2F3**) allows us to discount the latter pathway. While (**TS-D1D2**) is competitive with that computed for (**TS-GH**), the barrier found for the subsequent necessary 1-hexene insertion (15.1 kcal mol^–1^) is considerably lower than that associated with the Si–H/Mg–C metathesis reaction (24.9 kcal mol^–1^), suggesting that the latter step is rate determining and that the hydrosilylation process is, in any case, more likely to occur from **F** after complete alkylation of complex **4**.

## Conclusions

A dimeric β-diketiminato magnesium hydride (**4**) derivative reacts directly with terminal alkenes, the strained internal alkene, norbornene, and diphenylacetylene to provide the corresponding organomagnesium derivatives. Although the dinuclear structure of the magnesium hydride is retained during its initial reaction with 1-hexene, which occurs *via* a kinetic barrier (18.9 kcal mol^–1^) comparable to that deduced for the analogous reaction of the hydridocalcium compound (**3**),^12^ the resultant dimagnesium hydridoalkyl intermediate is found to be labile towards its rupture into mononuclear magnesium alkyl and hydride species. Catalytic hydrosilylation is, therefore, observed to occur *via* rate determining Si–H/Mg–C metathesis (24.9 kcal mol^–1^) of the three-coordinate organomagnesium derivative.

These observations reveal that hydridomagnesium compounds may display a much broader reactivity with alkenyl substrates than previously appreciated. The comparable reactivity of Parkin's terminal magnesium hydride (**1**) and compound **4** with styrene, however, hints that even more expansive substrate scope and catalytic activity may be achievable through the adoption of more sophisticated ligand design. Although not a central focus of our own research, we hope that our observations will prompt others toward a more wide-ranging and sustainable future.

## Conflicts of interest

There are no conflicts of interest to declare.

## Supplementary Material

Supplementary information

Crystal structure data
