# Supplementary material for: Magnesium hydride alkene insertion and catalytic hydrosilylation
Source: Chem Sci. 2019 Jul 22;10(35):8108–18. doi: 10.1039/c9sc02056j (PMC6839609; doi:10.1039/c9sc02056j)
Supplement: Supplementary file 1 [file SC-010-C9SC02056J-s001.pdf]

Electronic Supplementary Material (ESI) for Chemical Science.
This journal is © The Royal Society of Chemistry 2019

Supplementary Information for

Magnesium hydride alkene insertion and catalytic hydrosilylation

Lucia Garcia,[a] Chiara Dinoi,[b] Mary F. Mahon,[a] Laurent Maron[b]* and Michael S. Hill[a]

[a]*Department of Chemistry, University of Bath, Claverton Down, Bath, BA2 7AY, UK.* [b]*Université de Toulouse et CNRS, INSA, UPS, UMR 5215, LPCNO, 135 Avenue de Rangueil, F-31077 Toulouse, France*

General considerations and starting materials

All manipulations were carried out using standard Schlenk line and glovebox techniques under an inert atmosphere of argon. NMR experiments were conducted in J. Young tap NMR tubes prepared and sealed in a Glovebox. NMR spectra were collected on a Bruker AV300 spectrometer operating at 300.2 MHz (^{1}H) and 75.5 MHz (^{13}C) or an Agilent ProPulse spectrometer operating at 500 MHz (^{1}H) and 126 MHz (^{13}C). The spectra were referenced relative to residual protio solvent resonances. C_6D_6 was purchased from Sigma-Aldrich Corp., dried over a potassium mirror before distilling under argon and storing over molecular sieves in the glovebox. Phenylsilane (97%), allyltriphenylsilane (98%) and allyltrimethylsilane (98%) were purchased from Sigma-Aldrich Corp. and used without further purification. Diphenylacetylene (98%) and *trans*-stilbene (96%) were purchased from Sigma-Aldrich Corp., recrystalised from ethanol and dried under high vacuum. Liquid alkenes: 1,1-diphenylethene (97%), 3,3-dimethyl-1-butene (97%), 1-hexene (97%), 1,5-hexadiene (97%), 1-octene (98%), 1,7-octadiene (98%), allylbenzene (98%) and styrene (99%) were purchased from Sigma-Aldrich Corp., dried over calcium hydride and distilled under argon before use. Compound **4**, $[(BDI)MgH]_2$, (BDI = HC{(Me)CN-2,6-$i$$Pr_2C_6H_3$}$_2$) was synthesised by a literature procedure.[1] Microanalysis was performed by Mr. S. Boyer of London Metropolitan Enterprises.

Synthesis, spectroscopic and analytical data for new compounds

[(BDI)Mg{$(CH_2)_5CH_3$}] (6)

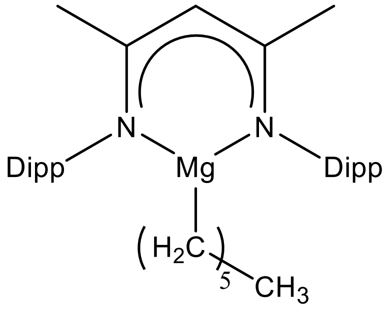

In a sealed J. Young NMR tube, $[(BDI)MgH]_2$ (20 mg, 0.024 mmol) and 1-hexene (6 μL, 0.048 mmol) were dissolved in C_6D_6 (0.5 mL). After heating the resulting colourless solution at 80 ºC for 4 hours, the solvent was removed under vacuum. Yield: 24 mg, 95%. ^{1}H NMR (500 MHz, C_6D_6, 298K): δ 7.17 – 7.14 (6H, C*H* Dipp), 4.94 (s, 1H, C*H*{C(CH$_3$)NDipp}$_2$), 3.16 (sept, $^{3}J_{HH}$ = 6.9 Hz, 4H, C*H*(CH$_3$)$_2$), 1.67 (s, 6H, CH{C(C*H*$_3$)NDipp}$_2$), 1.41 – 1.29 (4H, CH$_2$), 1.28 (d, $^{3}J_{HH}$ = 6.9 Hz, 12H, CH(C*H*$_3$)$_2$), 1.16 (d, $^{3}J_{HH}$ = 6.9 Hz, 12H, CH(C*H*$_3$)$_2$), 1.04 – 0.93 (4H, CH$_2$), 0.91 – 0.85 (m, 3H, C*H*$_3$), -0.22 – -0.27 ppm (m, 2H, MgC*H*$_2$). ^{13}C{^{1}H} NMR (125.8 MHz, C_6D_6, 298K): δ 169.3 (s, N*C*(Me)), 143.9 (s, N*C* Dipp), 141.9 (s, *C*-^{i}Pr), 126.0 (*p*-*C*H Dipp), 124.1 (*m*-*C*H Dipp), 95.4 (s, *C*H{C(Me)NDipp}$_2$), 38.1 (s, *C*H$_2$), 32.4 (s, *C*H$_2$), 28.8 (s, MgCH$_2$*C*H$_2$), 28.7 (s, C*H*(CH$_3$)$_2$ Dipp), 24.7 (s, C-*C*H$_3$ Dipp), 24.5 (s, *C*H$_3$), 23.6 (s, CH{C(*C*H$_3$)NDipp}$_2$), 23.5 (s, C-*C*H$_3$ Dipp), 14.6 (s, *C*H$_2$), 5.9 ppm (s, Mg-*C*H$_2$). Elemental analysis (%). Found: C 78.87, H 10.56, N 5.05. Calculated for $C_{35}H_{54}N_2Mg$: C 79.75, H 10.33, N 5.31.

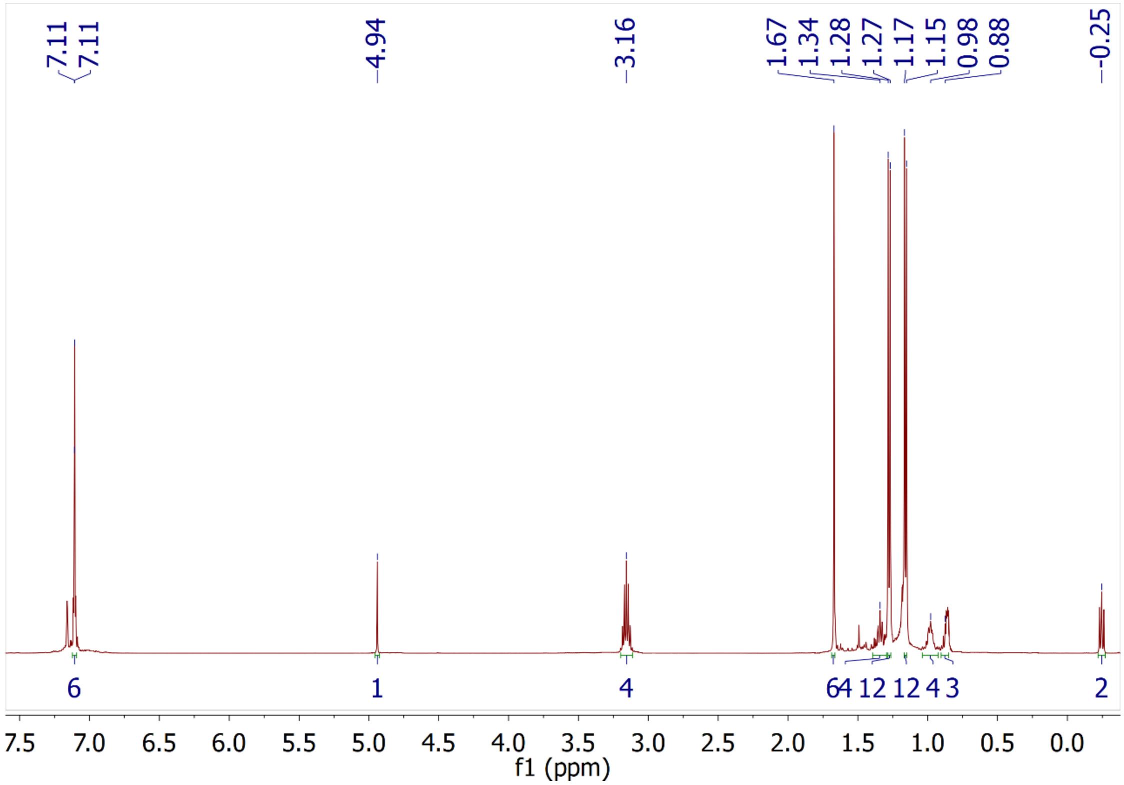

Figure S1. ^{1}H NMR spectrum (500 MHz, C_6D_6, 298K) of compound **6**.

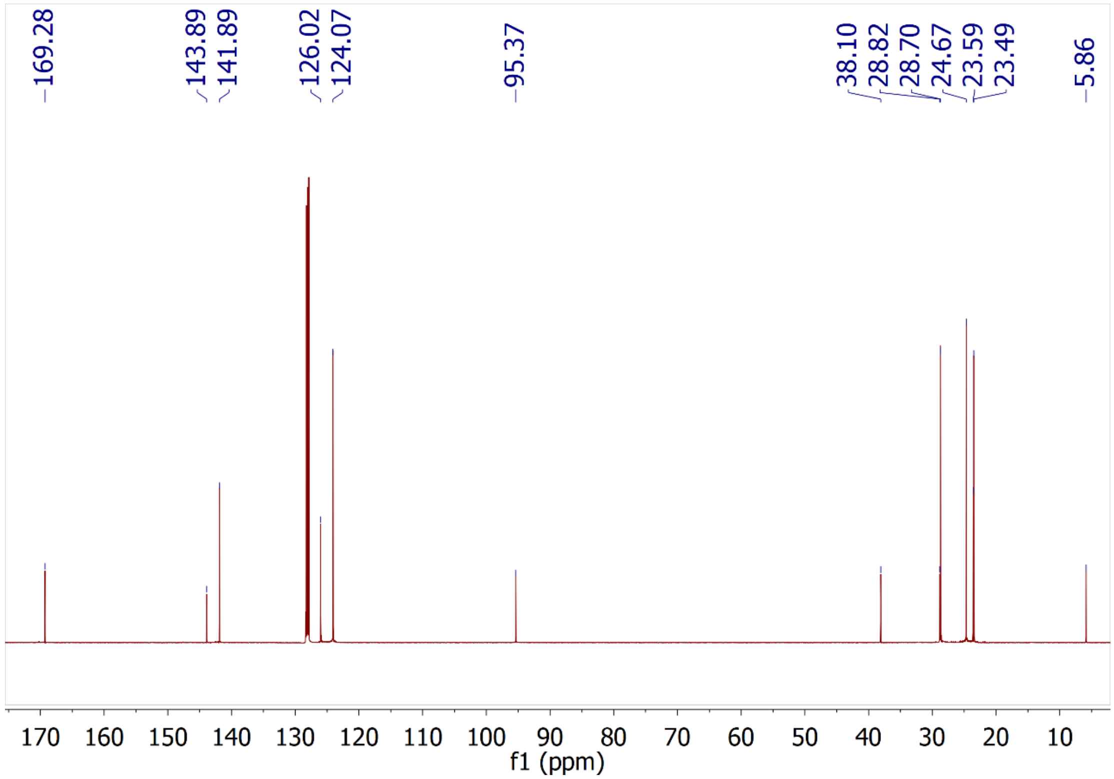

Figure S2. $^{13}C\{^{1}H\}$ NMR spectrum (125.8 MHz, C_6D_6, 298K) of compound **6**.

[(BDI)Mg{$(CH_2)_7CH_3$}] (7)

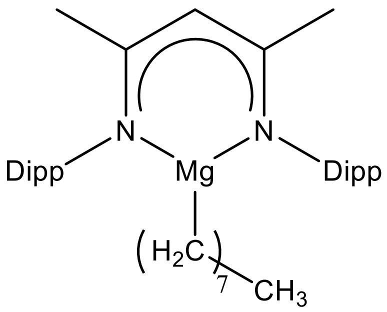

In a sealed J. Young NMR tube, 1-octene (7.7 μL, 0.048 mmol) was added to a C_6D_6 (0.5 mL) solution of $[(BDI)MgH]_2$ (20 mg, 0.024 mmol). The colourless solution was heated for 4 hours at 80 °C and then evaporated to dryness in vacuo. Yield: 27 mg, 96%. ^{1}H NMR (500 MHz, C_6D_6, 298K): δ 7.14 – 7.10 (6H, C*H* Dipp), 4.95 (s, 1H, C*H*{C(CH_3)NDipp}$_2$), 3.17 (sept, $^3J_{HH}$ = 6.9 Hz, 4H, C*H*(CH_3)$_2$), 1.67 (s, 6H, CH{C(CH_3)NDipp}$_2$), 1.44 – 1.31 (m, 2H, $MgCH_2CH_2$), 1.29 (d, $^3J_{HH}$ = 6.9 Hz, 12H, CH(CH_3)$_2$), 1.16 (d, $^3J_{HH}$ = 6.9 Hz, 12H, CH(CH_3)$_2$), 1.31 – 0.84 (13H, CH_2 and CH_3), -0.21 ppm (m, 2H, MgCH_2). ^{13}C{^{1}H} NMR (125.8 MHz, C_6D_6, 298K): δ 169.3 (s, N*C*(Me)), 143.9 (s, N*C* Dipp), 141.9 (s, *C*-^{i}Pr), 126.0 (*p*-*C*H Dipp), 124.1 (*m*-*C*H Dipp), 95.4 (s, *C*H{C(Me)NDipp}$_2$), 38.5 (s, $MgCH_2CH_2$*C*H_2), 32.5 – 23.5 (*C*H_2 and *C*H_3), 28.9 (s, MgCH*C*H_2), 28.7 (s, *C*H(CH_3)$_2$ Dipp), 24.7 (s, C-*C*H_3 Dipp), 23.6 (s, CH{C(*C*H_3)NDipp}$_2$), 23.5 (s, C-*C*H_3 Dipp), 5.9 ppm (s, Mg-*C*H_2). Elemental analysis (%). Found: C 81.56, H 10.98, N 4.91. Calculated for $C_{37}H_{58}MgN_2$: C 80.05, H 10.53, N 5.05.

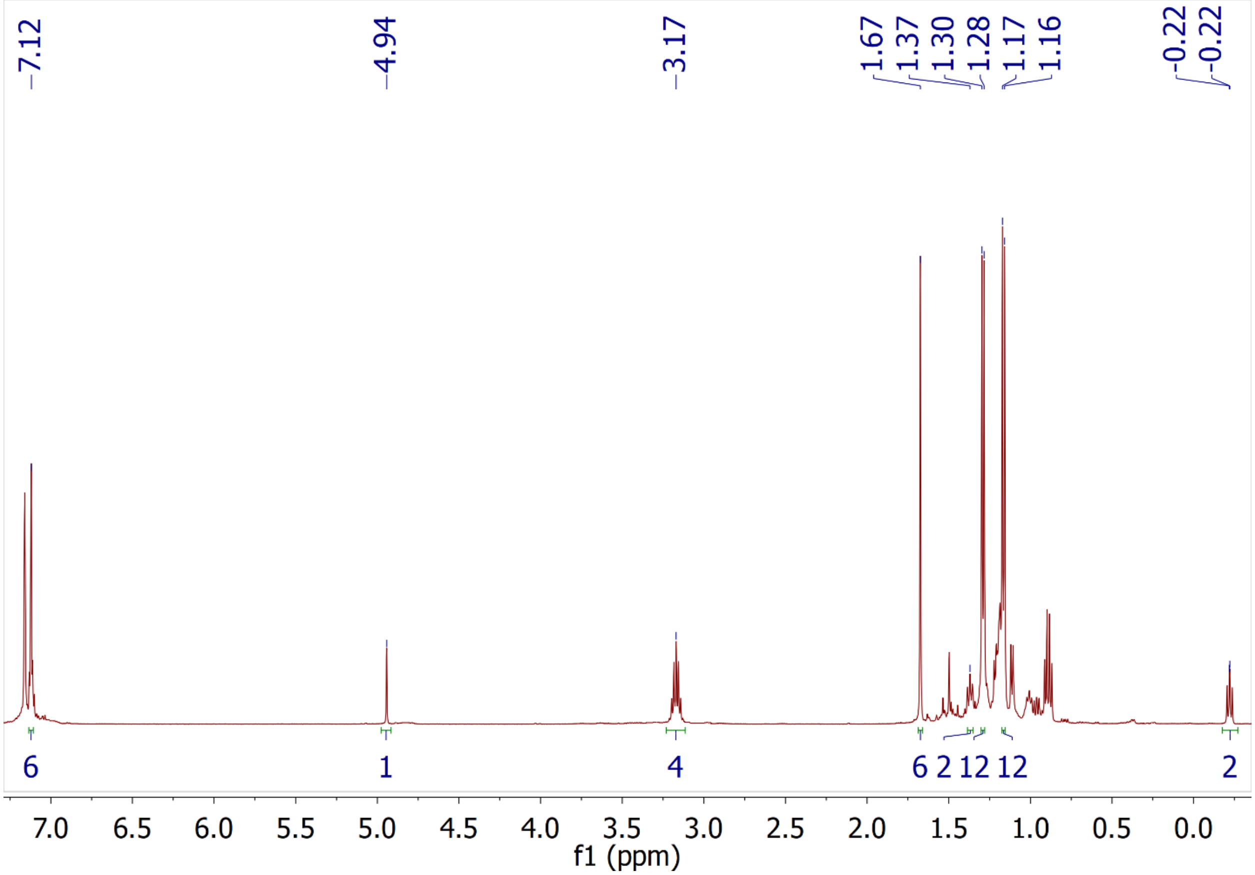

Figure S3. ^{1}H NMR spectrum (500 MHz, C_6D_6, 298K) of compound **7**.

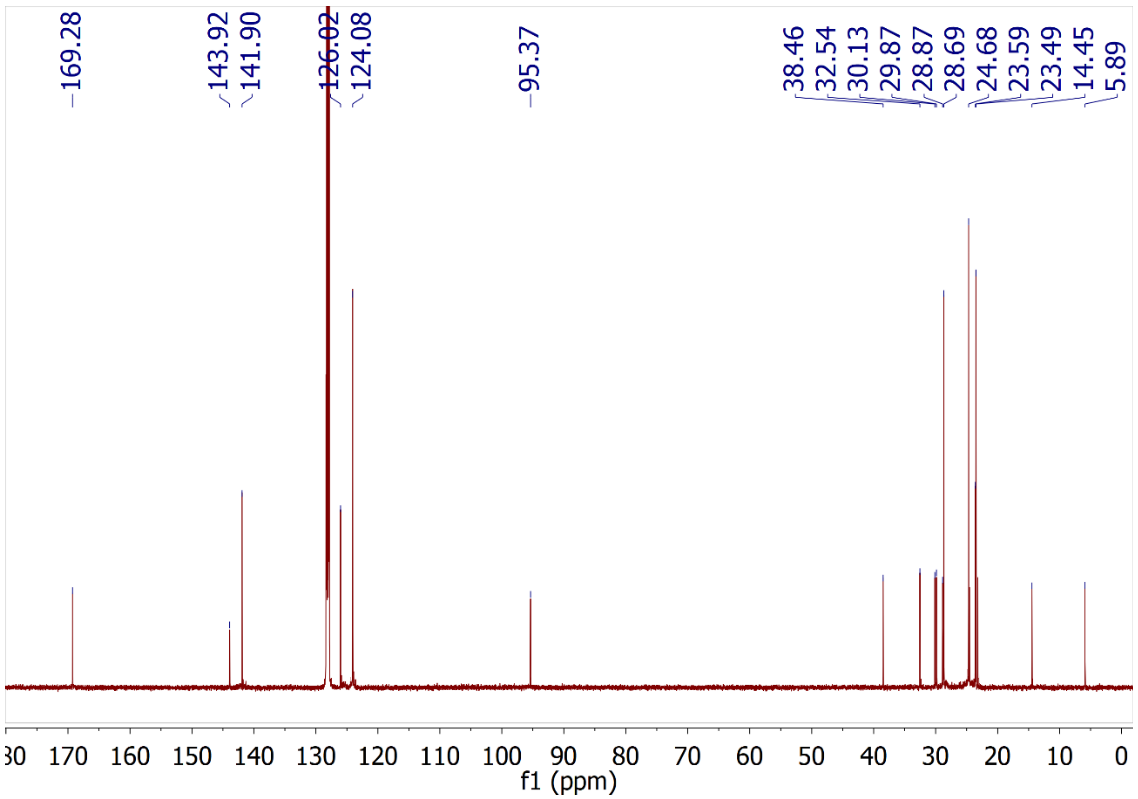

Figure S4. $^{13}C\{^{1}H\}$ NMR spectrum (125.8 MHz, C_6D_6, 298K) of compound **7**.

[(BDI)Mg{$(CH_2)_3$Ph}] (8)

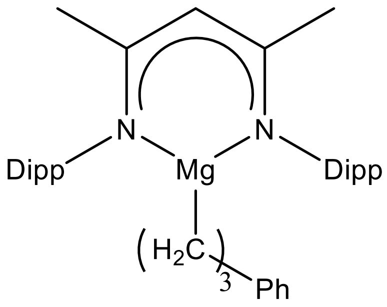

In a sealed J. Young NMR tube, allylbenzene (6.4 μL, 0.048 mmol) was added to a C_6D_6 (0.5 mL) solution of $[(BDI)MgH]_2$ (20 mg, 0.024 mmol). The colourless solution was heated for 2 days at 80ºC and then evaporated to dryness in vacuo. Yield: 25 mg, 92%. ^{1}H NMR (500 MHz, C_6D_6, 298K): δ 7.12 – 7.08 (6H, C*H* Dipp), 7.07 – 6.96 (5H, C*H* Ph), 4.93 (s, 1H, C*H*{C(CH_3)NDipp}$_2$), 3.14 (sept, $^3J_{HH}$ = 6.9 Hz, 4H, C*H*(CH_3)$_2$), 2.25 (m, 2H, PhC*H*$_2$), 1.66 (s, 6H, CH{C(C*H*$_3$)NDipp}$_2$), 1.64 – 1.57 (m, 2H, MgCH$_2$C*H*$_2$), 1.24 (d, $^3J_{HH}$ = 6.9 Hz, 12H, CH(C*H*$_3$)$_2$), 1.14 (d, $^3J_{HH}$ = 6.9 Hz, 12H, CH(C*H*$_3$)$_2$), -0.21 ppm (m, 2H, MgC*H*$_2$). ^{13}C{^{1}H} NMR (125.8 MHz, C_6D_6, 298K): δ 169.3 (s, N*C*(Me)), 144.5 (s, *C* Ph), 143.8 (s, N*C* Dipp), 141.9 (s, *C*-^{i}Pr), 127.7 (*o*-*C*H Ph), 126.1 – 125.2 (*C*H Dipp and Ph), 124.1 (*m*-*C*H Dipp), 95.4 (s, *C*H{C(Me)NDipp}$_2$), 44.7 (s, MgCH$_2$CH$_2$*C*H$_2$), 31.1 (s, MgCH$_2$*C*H$_2$), 28.7 (s, *C*H(CH_3)$_2$ Dipp), 24.7 (s, C-*C*H$_3$ Dipp), 23.5 (s, CH{C(*C*H$_3$)NDipp}$_2$), 23.4 (s, C-*C*H$_3$ Dipp), 6.0 ppm (s, Mg*C*H$_2$). Elemental analysis (%). Found: C 79.99, H 9.33, N 5.16. Calculated for $C_{38}H_{52}MgN_2$: C 80.34, H 9.34, N 4.99.

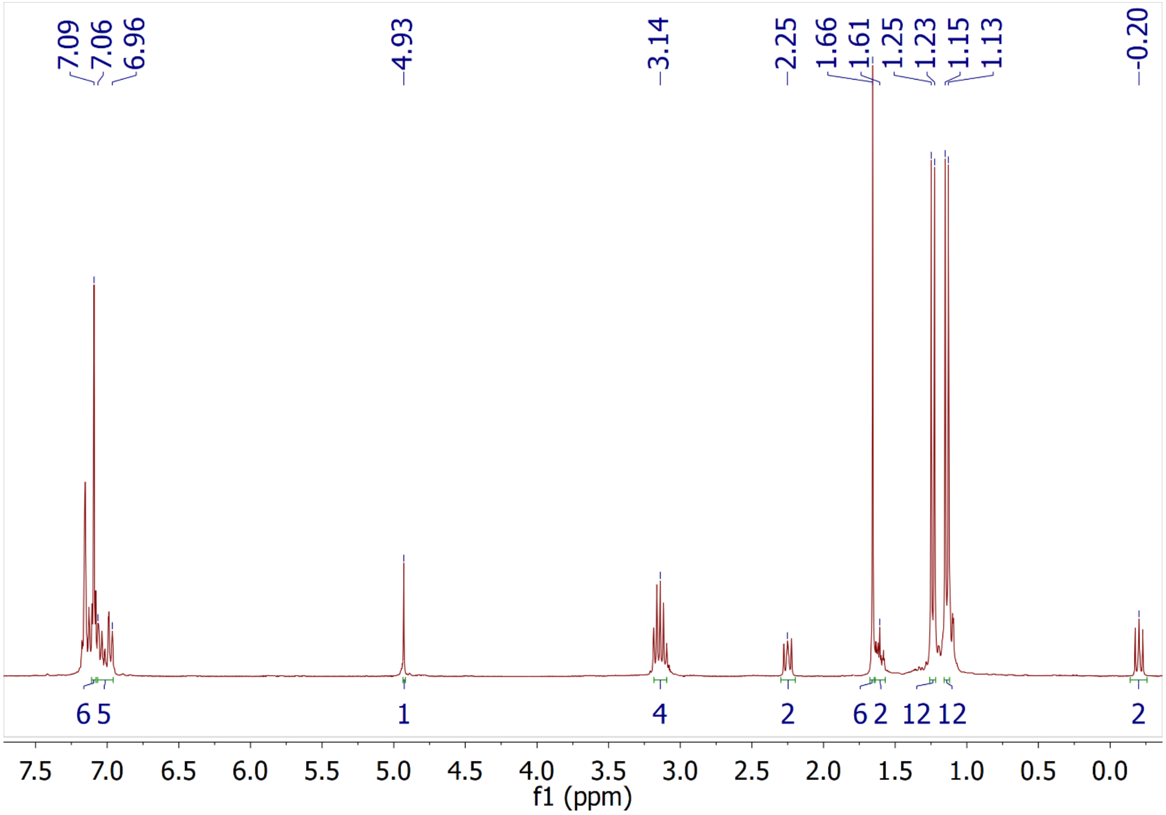

Figure S5 ^{1}H NMR spectrum (500 MHz, C_6D_6, 298K) of compound **8**.

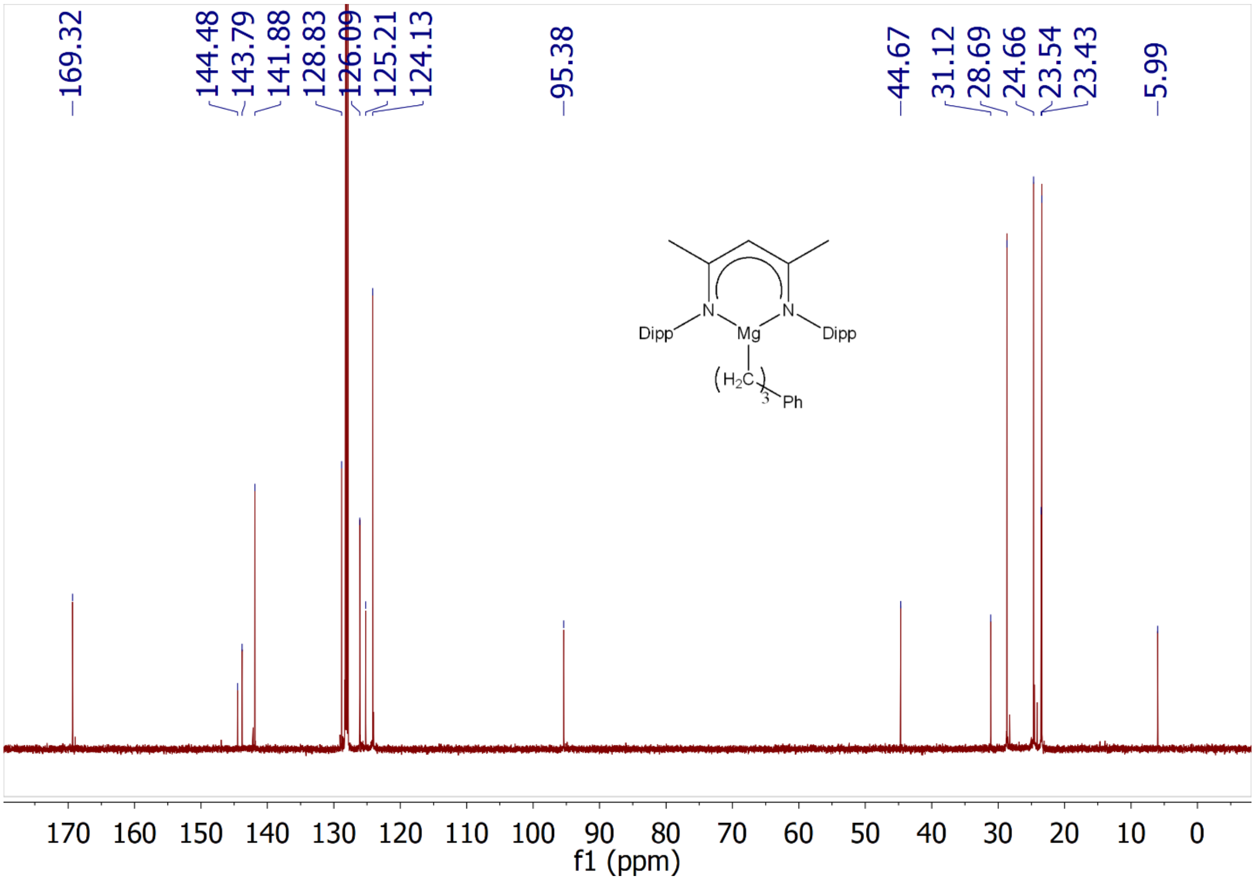

Figure S6. $^{13}C\{^{1}H\}$ NMR spectrum (125.8 MHz, C_6D_6, 298K) of compound **8**.

[(BDI)Mg{$(CH_2)_2C(CH_3)_3$}] (9)

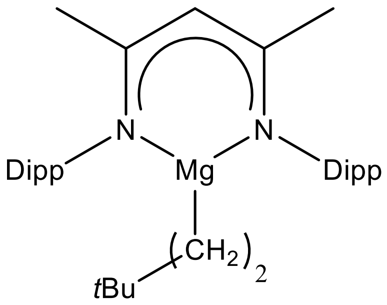

$[(BDI)MgH]_2$ (20 mg, 0.024 mmol) and 3,3-dimethyl-1-butene (6.0 µL, 0.048 mmol) were dissolved in C_6D_6 (0.5 mL) in a sealed J. Young NMR tube. After heating the resulting colourless solution at 80 ºC for 21 days, the solvent was removed under vacuum. Yield: 25 mg, 91%. ^{1}H NMR (500 MHz, C_6D_6, 298K): δ 7.13 – 7.09 (6H, C*H* Dipp), 4.94 (s, 1H, C*H*{C(CH_3)NDipp}$_2$), 3.16 (sept, $^3J_{HH}$ = 6.9 Hz, 4H, C*H*(CH_3)$_2$), 1.67 (s, 6H, CH{C(C*H*$_3$)NDipp}$_2$), 1.29 (d, $^3J_{HH}$ = 6.9 Hz, 12H, CH(C*H*$_3$)$_2$), 1.16 (d, $^3J_{HH}$ = 6.9 Hz, 12H, CH(C*H*$_3$)$_2$), 0.94 – 0.88 (m, 2H, MgCH_2C*H*$_2$), 0.79 (s, 9H, C(C*H*$_3$)$_3$), -0.44 – -0.48 ppm (m, 2H, MgC*H*$_2$); ^{13}C{^{1}H} NMR (125.8 MHz, C_6D_6, 298K): δ 169.3 (s, N*C*(Me)), 143.8 (s, N*C* Dipp), 141.9 (s, *C*-^{i}Pr), 126.0 (*p*-*C*H Dipp), 124.1 (*m*-*C*H Dipp), 95.3 (s, *C*H{C(Me)NDipp}$_2$), 43.0 (s, MgCH*C*H$_2$), 32.5 (s, MgCH_2CH_2*C*), 28.8 (s, *C*H(CH_3)$_2$ Dipp), 28.7 (s, C(*C*H$_3$)$_3$), 24.6 (s, C-*C*H$_3$ Dipp), 23.5 (s, CH{C(*C*H$_3$)NDipp}$_2$), 23.5 (s, C-*C*H$_3$ Dipp), -2.7 ppm (s, Mg-*C*H$_2$). Elemental analysis (%). Found: C 77.93, H 9.60, N 4.83. Calculated for $C_{35}H_{54}MgN_2$: C 79.75, H 10.33, N 5.31.

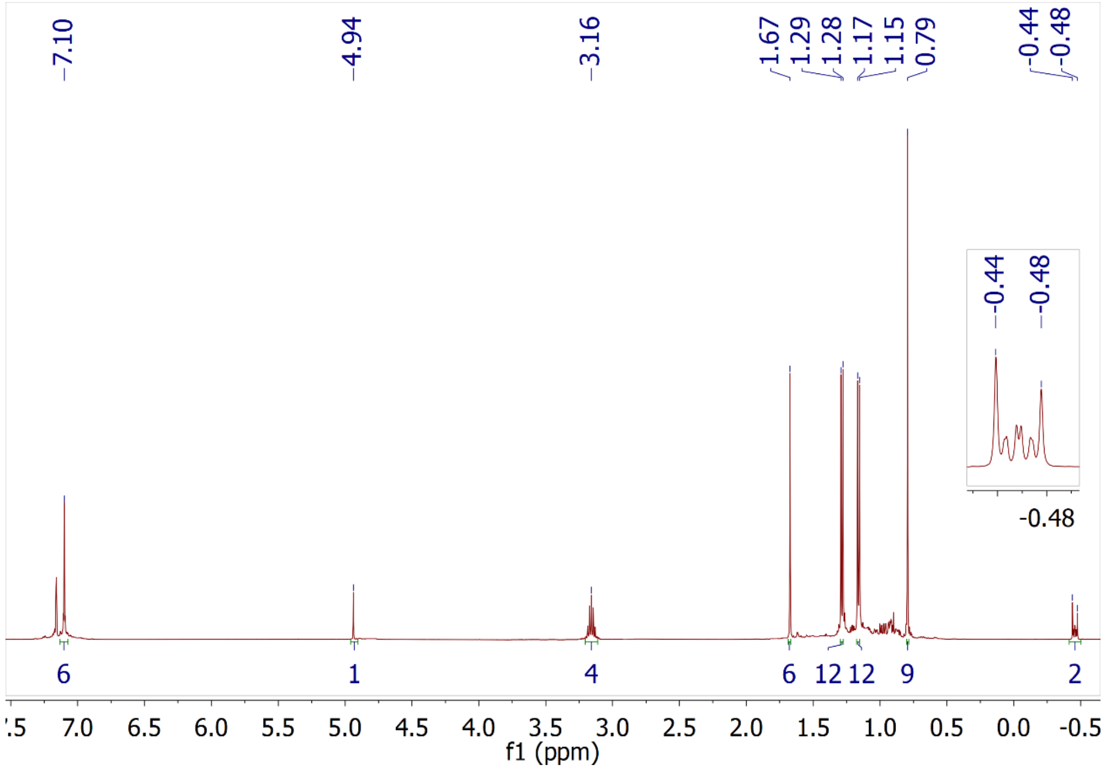

Figure S7. ^{1}H NMR spectrum (500 MHz, C_6D_6, 298K) of compound **9**.

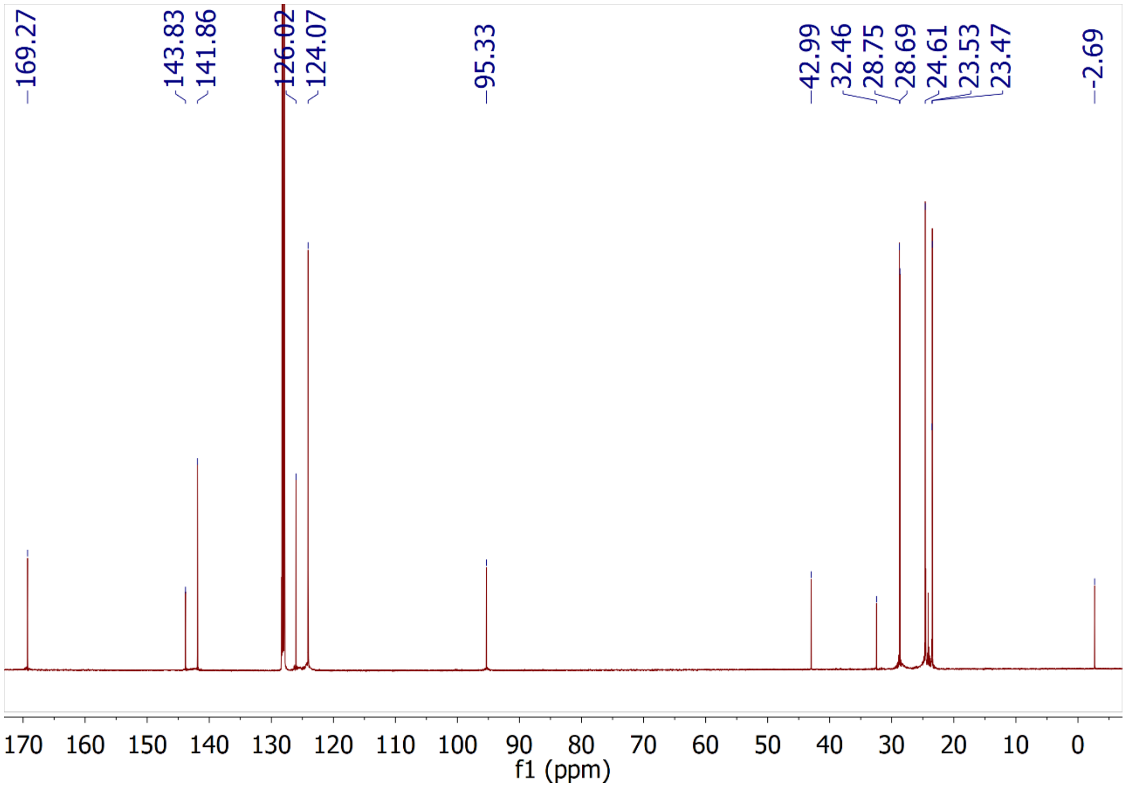

Figure S8. $^{13}C\{^{1}H\}$ NMR spectrum (125.8 MHz, C_6D_6, 298K) of compound **9**.

[(BDI)Mg{$(CH_2)_2$Ph}] and [(BDI)Mg{CH(CH_3)(Ph)}] (10A,10B)

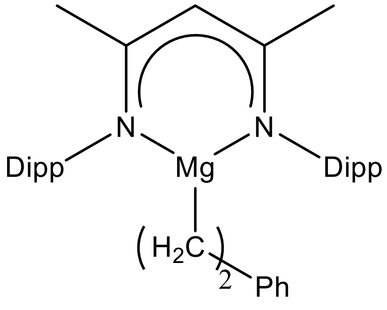

A

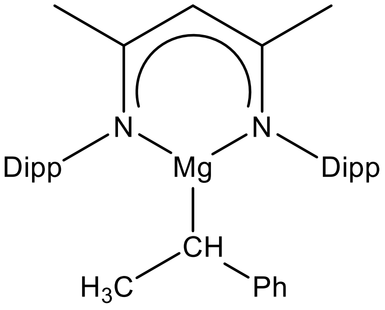

B

[(BDI)MgH]$_2$ (20 mg, 0.024 mmol) and styrene (5.6 μL, 0.048 mmol) were dissolved in C_6D_6 (0.5 mL) in a sealed J. Young NMR tube. After heating the resultant colourless solution for 7 days at 80 ºC, the solvent was removed. Yield: 24 mg, 89%. ^{1}H NMR (500 MHz, C_6D_6, 298K): δ <u>**A** (55%)</u>: 7.26 – 6.96 (6H, C*H* Dipp and Ph), 4.94 (s, 1H, C*H*{C(CH_3)NDipp}$_2$), 3.14 (sept, $^3J_{HH}$ = 6.9 Hz, 4H, C*H*(CH_3)$_2$), 2.44 (m, 2H, MgCH$_2$C*H*$_2$), 1.67 (s, 6H, CH{C(C*H*$_3$)NDipp}$_2$), 1.23 (d, $^3J_{HH}$ = 6.9 Hz, 12H, CH(C*H*$_3$)$_2$), 1.16 (d, $^3J_{HH}$ = 6.9 Hz, 12H, CH(C*H*$_3$)$_2$), 0.01 ppm (m, 2H, MgC*H*$_2$); <u>**B** (45%)</u>: 7.26 – 6.96 (6H, C*H* Dipp), 6.93 (m, 2H, *m*-C*H* Ph), 6.71 (m, 1H, *p*-C*H* Ph), 6.39 (m, 2H, *o*-C*H* Ph), 4.89 (s, 1H, C*H*{C(CH_3)NDipp}$_2$), 3.07 (sept, $^3J_{HH}$ = 6.9 Hz, 2H, C*H*(CH_3)$_2$), 2.97 (sept, $^3J_{HH}$ = 6.9 Hz, 2H, C*H*(CH_3)$_2$), 1.92 (c, $^3J_{HH}$ = 7.1 Hz, 1H, C*H*Ph), 1.64 (s, 6H, CH{C(C*H*$_3$)NDipp}$_2$), 1.26 (d, $^3J_{HH}$ = 6.9 Hz, 6H, CH(C*H*$_3$)$_2$), 1.14 (d, $^3J_{HH}$ = 7.1 Hz, 3H, PhCHC*H*$_3$), 1.12 (d, $^3J_{HH}$ = 6.9 Hz, 3H, CH(C*H*$_3$)$_2$), 1.11 (d, $^3J_{HH}$ = 6.9 Hz, 6H, CH(C*H*$_3$)$_2$), 0.98 (d, $^3J_{HH}$ = 6.9 Hz, 3H, CH(C*H*$_3$)$_2$), 0.95 ppm (d, $^3J_{HH}$ = 6.9 Hz, 6H, CH(C*H*$_3$)$_2$). ^{13}C{^{1}H} NMR (125.8 MHz, C_6D_6, 298K): δ <u>**A** (55%)</u>: 169.4 (s, N*C*(Me)), 151.1 (s, *C* Ph), 143.7 (s, N*C* Dipp), 141.9 (s, *C*-^{i}Pr), 127.7 (*o*-*C*H Dipp), 128.8 – 124.2 (*C*H Dipp and Ph), 95.4 (s, *C*H{C(Me)NDipp}$_2$), 34.5 (s, MgCH$_2$*C*H$_2$), 28.7 (s, *C*H(CH_3)$_2$ Dipp), 24.6 (s, C-*C*H$_3$ Dipp), 23.5 (s, CH{C(*C*H$_3$)NDipp}$_2$), 23.4 (s, C-*C*H$_3$ Dipp), 7.7 ppm (s, Mg*C*H$_2$); <u>**B** (45%)</u>: 169.5 (s, N*C*(Me)), 156.7 (s, *C* Ph), 144.1 (s, N*C* Dipp), 141.7 (s, *C*-^{i}Pr), 141.4 (s, *C*-^{i}Pr), 128.5 (s, *m*-*C*H Ph), 128.8 – 124.2 (*C*H Dipp), 123.0 (s, *o*-*C*H Ph), 118.5 (s, *p*-*C*H Ph), 95.2 (s, *C*H{C(Me)NDipp}$_2$), 28.9 (s, *C*H(CH_3)$_2$ Dipp), 28.7 (s, *C*H(CH_3)$_2$ Dipp), 26.7 (s, Ph*C*HCH$_3$), 24.9, 24.4, 23.8, 23.8, 23.4 (s, C-*C*H$_3$ Dipp), 23.2 (s, CH{C(*C*H$_3$)NDipp}$_2$), 16.4 ppm (s, PhCH*C*H$_3$). Elemental analysis (%). Found: C 82.02, H 9.30, N 4.94. Calculated for $C_{37}H_{50}MgN_2$: C 81.23, H 9.21, N 5.12.

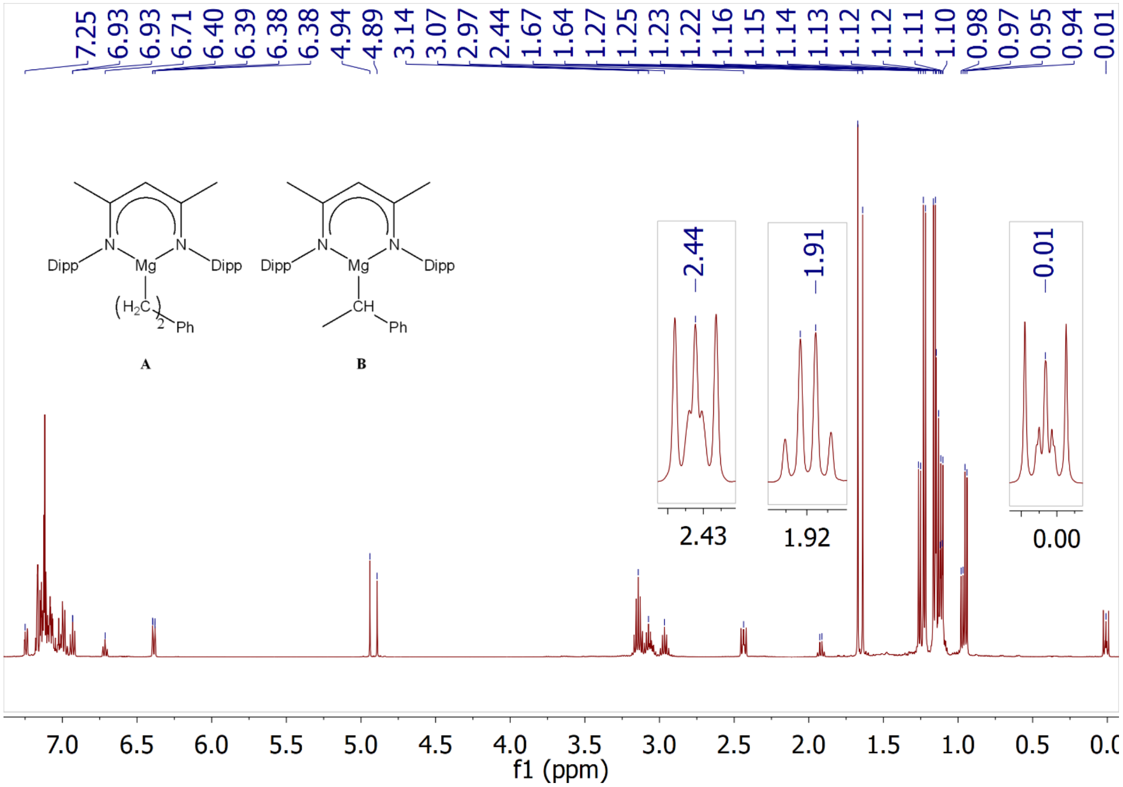

Figure S9. ^{1}H NMR spectrum (500 MHz, C_6D_6, 298K) of compounds **10A** (55%) and **10B** (45%).

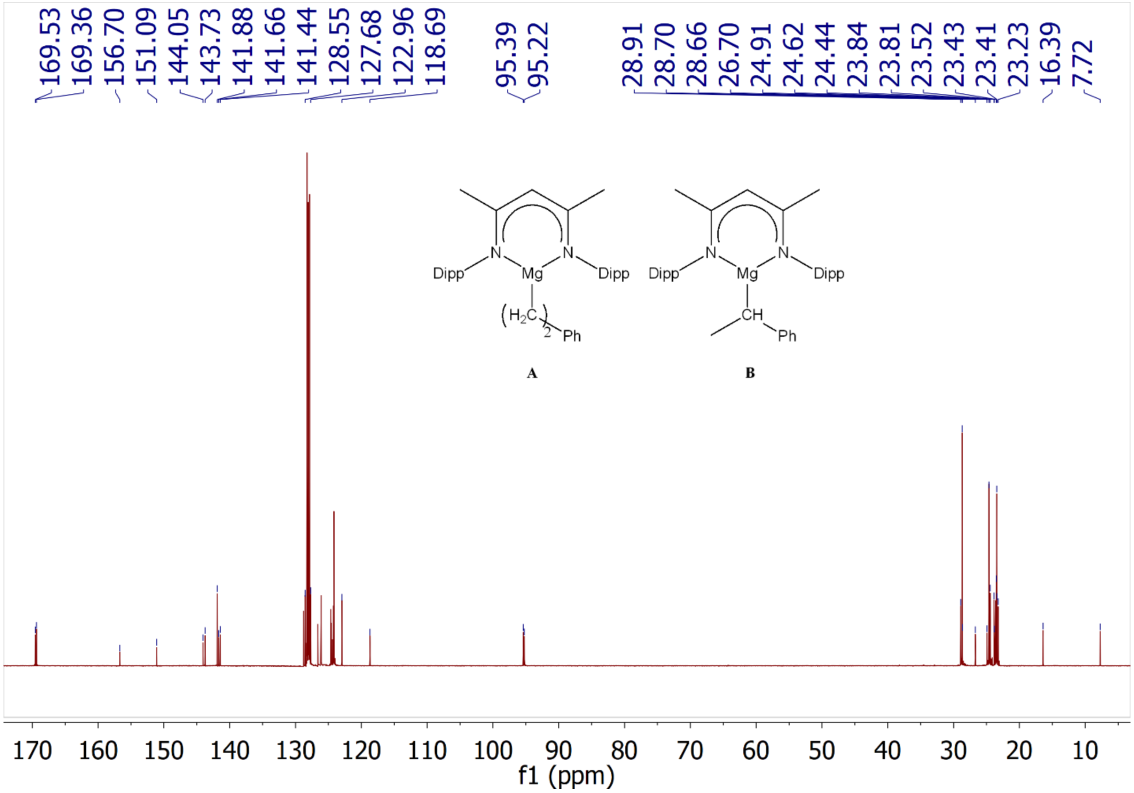

Figure S10. $^{13}C\{^{1}H\}$ NMR spectrum (125.8 MHz, C_6D_6, 298K) of compounds **10A** (55%) and **10B** (45%).

[(BDI)Mg{C(Ph)$_2$(CH$_3$)}] (11)

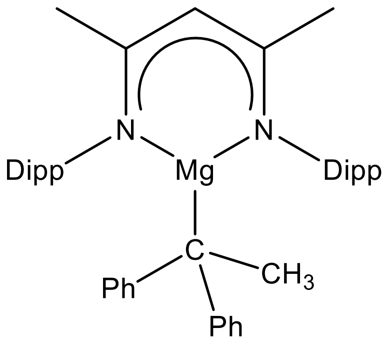

[(BDI)MgH]$_2$ (20 mg, 0.024 mmol) and 1,1-diphenylethylene (8.6 µL, 0.048 mmol) were dissolved in C_6D_6 (0.5 mL) in a sealed J. Young NMR tube. After heating the resulting colorless solution at 100ºC for 6 weeks the reaction reached a conversion of 64% (by NMR). ^{1}H NMR (500 MHz, C_6D_6, 298K): δ 7.13 – 7.09 (6H, C*H* Dipp), 6.99 – 6.97 (4H, C*H* Ph), 6.88 – 6.86 (4H, C*H* Ph), 6.85 – 6.81 (2H, C*H* Ph), 4.92 (s, 1H, C*H*{C(CH$_3$)NDipp}$_2$), 3.01 (sept, $^3J_{HH}$ = 6.9 Hz, 4H, C*H*(CH$_3$)$_2$), 1.63 (s, 6H, CH{C(C*H*$_3$)NDipp}$_2$), 1.26 (s, 3H, MgC(C*H*$_3$)), 1.08 (d, $^3J_{HH}$ = 6.9 Hz, 12H, CH(C*H*$_3$)$_2$), 0.94 ppm (d, $^3J_{HH}$ = 6.9 Hz, 12H, CH(C*H*$_3$)$_2$); ^{13}C{^{1}H} NMR (125.8 MHz, C_6D_6, 298K): δ 170.9 (s, N*C*(Me)), 156.0 (s, C$_{ipso}$ Ph), 145.1 (s, N*C* Dipp), 142.4 (s, *C*-^{i}Pr), 129.4, 127.7 (*C*H Ph), 126.6 (*p*-*C*H Dipp), 124.8 (*m*-*C*H Dipp), 122.3 (*C*H Ph), 96.1 (s, *C*H{C(Me)NDipp}$_2$), 46.1 (s, Mg-*C*), 29.4 (s, *C*H(CH$_3$)$_2$ Dipp), 27.4 (s, MgC-*C*H$_3$), 24.9 (s, CH{C(*C*H$_3$)NDipp}$_2$), 24.8, 24.3 ppm (s, C-*C*H$_3$ Dipp).

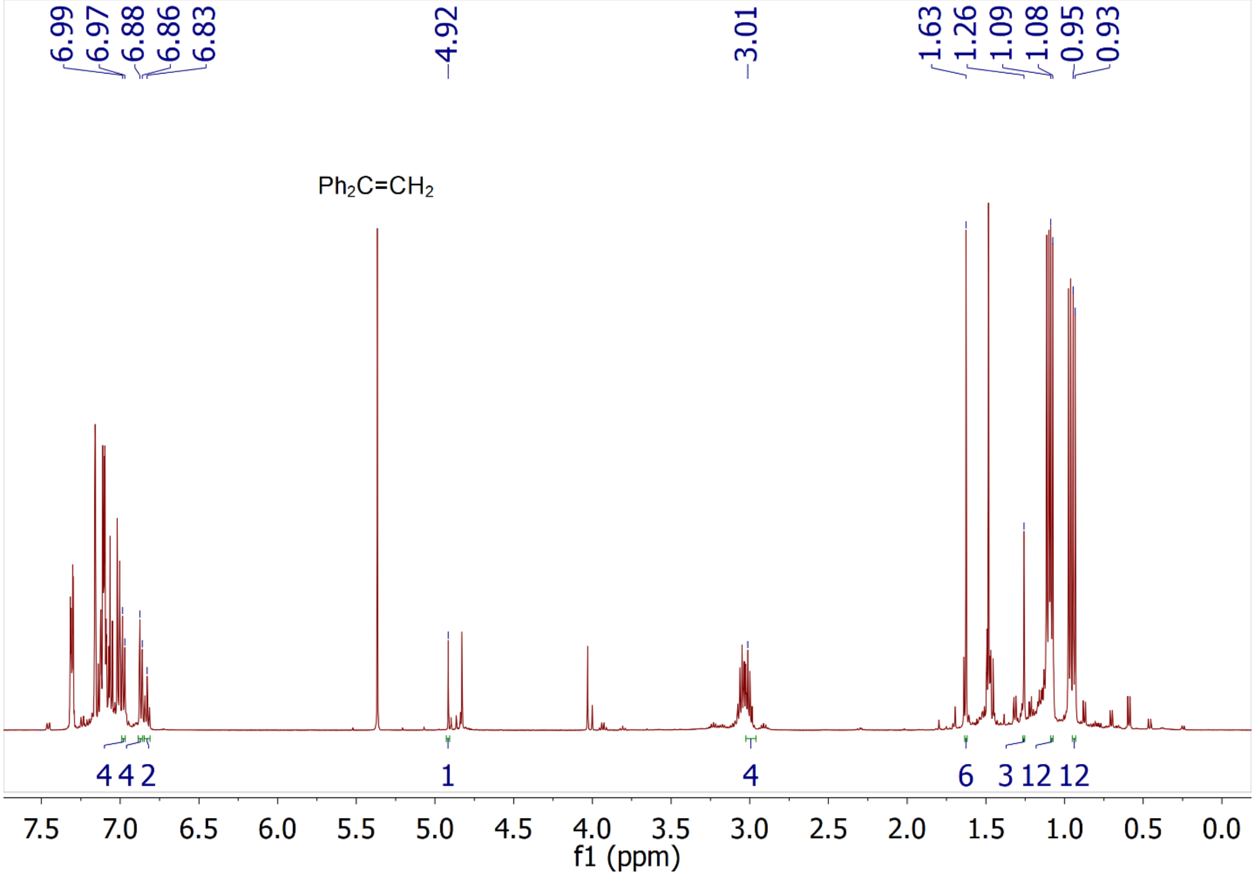

Figure S11. ^{1}H NMR spectrum (500 MHz, C_6D_6, 298K) of reaction between [(BDI)MgH]$_2$ (0.024 mmol) and 1,1-diphenylethylene (0.048 mmol) in C_6D_6 after 6 weeks at 100ºC.

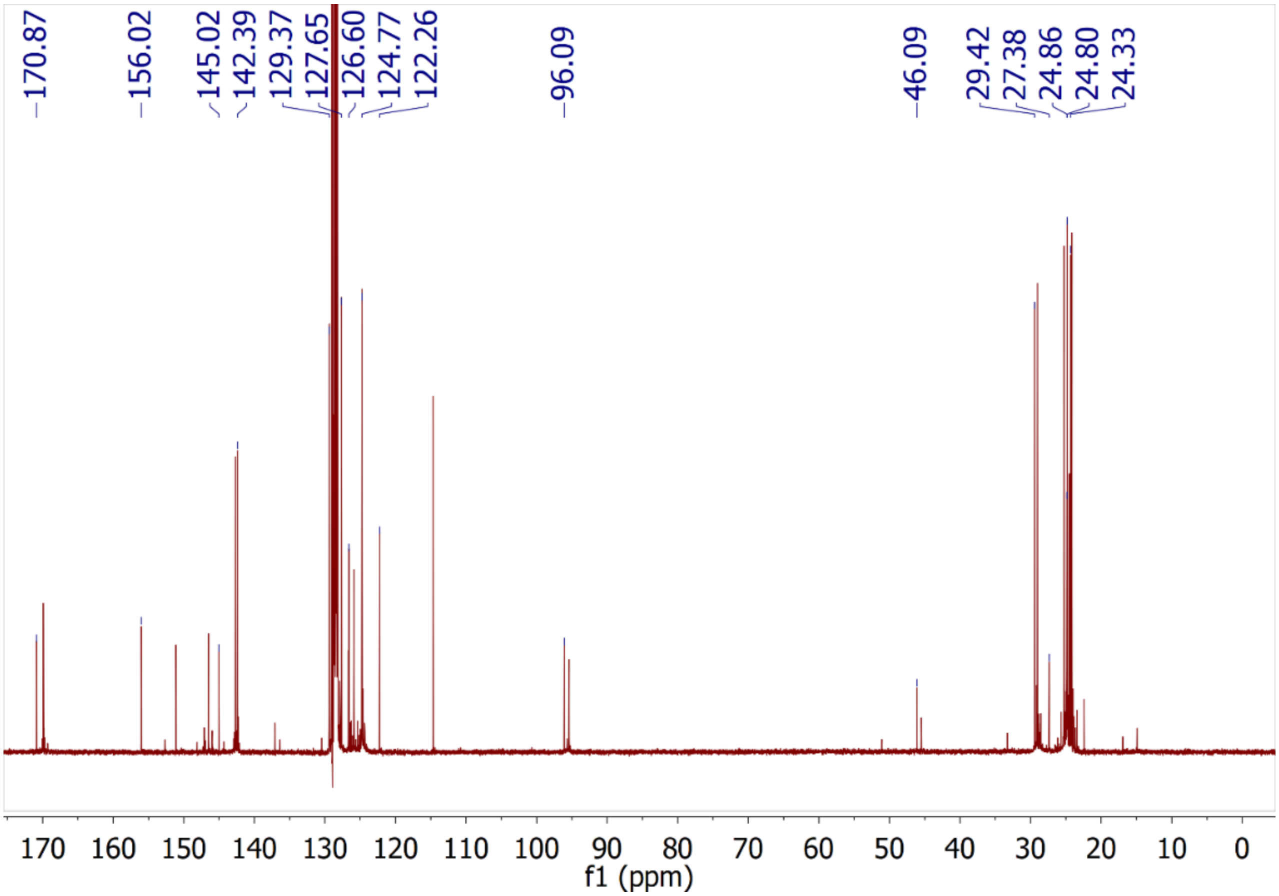

Figure S12. $^{13}C\{^{1}H\}$ NMR spectrum (125.8 MHz, C_6D_6, 298K) of reaction between $[(BDI)MgH]_2$ (0.024 mmol) and 1,1-diphenylethylene (0.048 mmol) in C_6D_6 after 6 weeks at 100ºC.

[(BDI)Mg{$CH_2CH(CH_2)_4$}] (12)

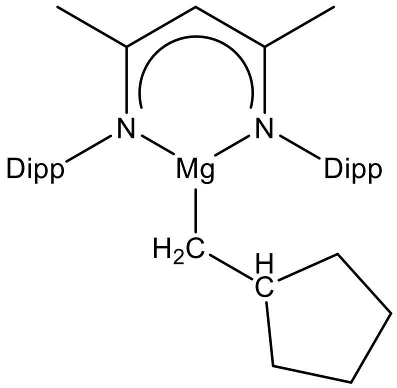

In a sealed J. Young NMR tube, 1,5-hexadiene (5.8 μL, 0.048 mmol) was added to a C_6D_6 (0.5 mL) solution of [(BDI)MgH]$_2$ (20 mg, 0.024 mmol). The reaction was heated for 1.5 days at 80ºC and then evaporated to dryness in vacuo. Colourless single crystals of **12** suitable for X-ray crystallography were grown by slow evaporation of a saturated hexane solution at room temperature. Yield: 22 mg, 94%. ^{1}H NMR (500 MHz, C_6D_6, 298K): δ 7.14 – 7.08 (6H, C*H* Dipp), 4.94 (s, 1H, C*H*{C(CH_3)NDipp}$_2$), 3.16 (sept, $^3J_{HH}$ = 6.9 Hz, 4H, C*H*(CH_3)$_2$), 1.98 – 1.85 (m, 1H, MgCH_2C*H*), 1.67 (s, 6H, CH{C(CH_3)NDipp}$_2$), 1.61 – 1.53 (4H, CH_2), 1.46 – 1.38 (4H, CH_2), 1.29 (d, $^3J_{HH}$ = 6.9 Hz, 12H, CH(CH_3)$_2$), 1.15 (d, $^3J_{HH}$ = 6.9 Hz, 12H, CH(CH_3)$_2$), 0.72 – 0.58 (2H, CHCH_2), -0.06 ppm (d, $^3J_{HH}$ = 6.8 Hz, 2H, MgCH_2). ^{13}C{^{1}H} NMR (125.8 MHz, C_6D_6, 298K): δ 169.3 (s, N*C*(Me)), 144.0 (s, N*C* Dipp), 141.9 (s, *C*-^{i}Pr), 126.0 (s, *p*-*C*H Dipp), 124.1 (s, *m*-*C*H Dipp), 95.4 (s, *C*H{C(Me)NDipp}$_2$), 41.3 (s, MgCH_2*C*H), 40.4 (s, CH_2), 28.8 (s, C*H*(CH_3)$_2$ Dipp), 25.8 (s, CH_2), 24.6 (s, C-*C*H_3 Dipp), 23.7 (s, CH{C(*C*H_3)NDipp}$_2$), 23.6 (s, C-*C*H_3 Dipp), 13.7 ppm (s, Mg*C*H_2). Elemental analysis (%). Found: C 79.02, H 9.80, N 3.42. Calculated for $C_{35}H_{52}MgN_2$: C 80.06, H 9.98, N 3.33.

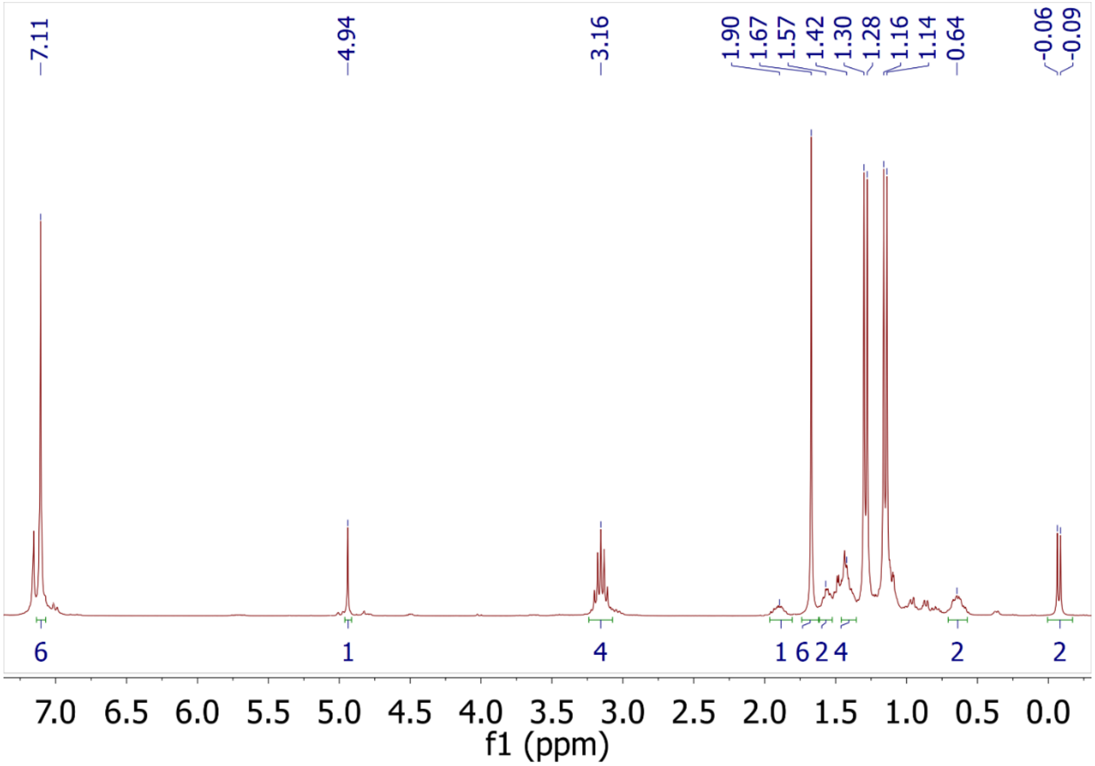

Figure S13. ^{1}H NMR spectrum (500 MHz, C_6D_6, 298K) of compound **12**.

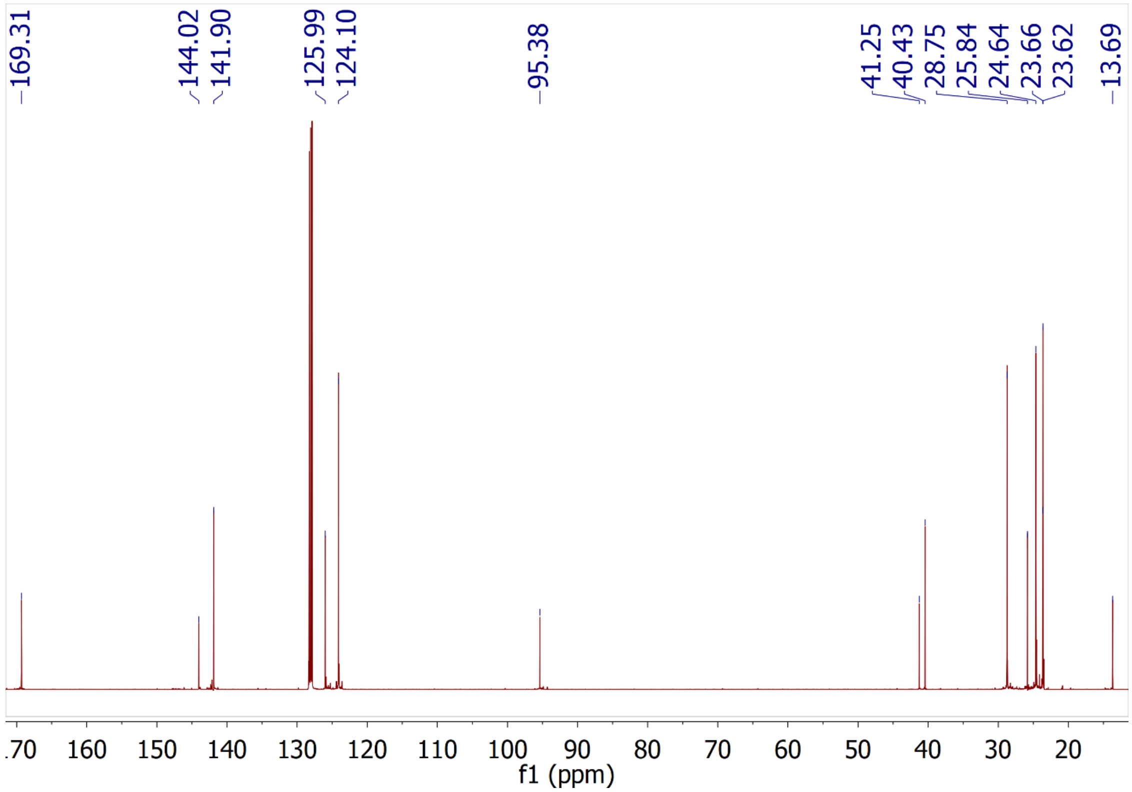

Figure S14. $^{13}C\{^1H\}$ NMR spectrum (125.8 MHz, C_6D_6, 298K) of compound **12**.

[(BDI)Mg{$(CH_2)_6CH{=}CH_2$}] (13) and [(BDI)Mg$(CH_2)_6$Mg(BDI)] (14)

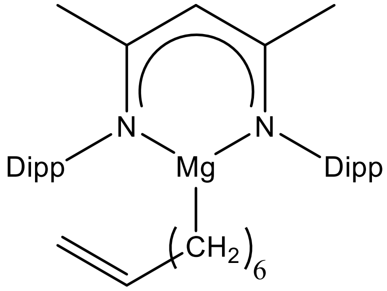

A

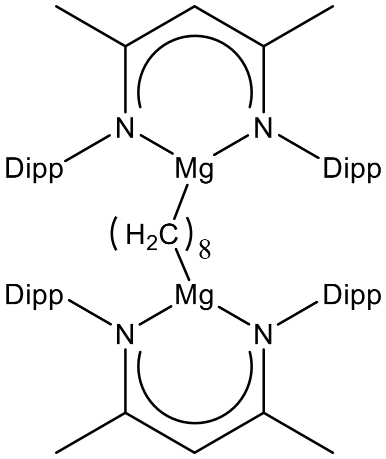

B

In a sealed J. Young NMR tube, C_6D_6 (0.5 mL) was added to a mixture of $[(BDI)MgH]_2$ (20 mg, 0.024 mmol) and 1,7-octadiene (7.2 μL, 0.048 mmol). After heating the resulting colourless solution for 5 hours at 80 ºC, the solvent was removed under vacuum. Yield: 23 mg, 88% (50% **13** + 50% **14**). ^{1}H NMR (500 MHz, C_6D_6, 298K): δ **13** (50%): 7.15 – 7.09 (6H, C*H* Dipp), 5.80 (ddt, $^3J_{HH}$ = 17.0, 10.3, 6.7 Hz, 1H, C*H*=CH_2), 5.02 (ddt, $^3J_{HH}$ = 17.1, $^4J_{HH}$ = $^2J_{HH}$ = 1.8 Hz, 1H, CH=CH_2), 4.97 (ddt, $^3J_{HH}$ = 10.3, $^2J_{HH}$ =1.9 Hz, $^4J_{HH}$ = 1.0, 1H, CH=CH_2), 4.94 (s, 1H, C*H*{C(CH_3)NDipp}$_2$), 3.16 (sept, $^3J_{HH}$ = 6.9 Hz, 4H, C*H*(CH_3)$_2$), 1.96 – 1.90 (m, 2H, CH_2CH=CH_2), 1.66 (s, 6H, CH{C(CH_3)NDipp}$_2$), 1.40 – 1.26 (m, 1H, MgCH_2CH_2), 1.28 (d, $^3J_{HH}$ = 6.9 Hz, 12H, CH(CH_3)$_2$), 1.27 – 1.17 (m, 2H, C$H_2$$CH_2$CH=CH), 1.16 (d, $^3J_{HH}$ = 6.9 Hz, 12H, CH(CH_3)$_2$), 1.00 – 0.90 (m, 2H, CH_2), 0.90 – 0.82 (m, 2H, CH_2), -0.24 ppm (t, $^3J_{HH}$ = 7.9 Hz, 2H, MgCH_2); **14** (50%): 7.15 – 7.09 (12H, C*H* Dipp), 4.94 (s, 2H, C*H*{C(CH_3)NDipp}$_2$), 3.16 (sept, $^3J_{HH}$ = 6.9 Hz, 8H, C*H*(CH_3)$_2$), 1.67 (s, 12H, CH{C(CH_3)NDipp}$_2$), 1.38 – 1.28 (m, 2H, MgCH_2CH_2), 1.28 (d, $^3J_{HH}$ = 6.9 Hz, 12H, CH(CH_3)$_2$), 1.16 (d, $^3J_{HH}$ = 6.9 Hz, 12H, CH(CH_3)$_2$), 1.40 – 0.82 (8H, CH_2), -0.23 ppm (t, $^3J_{HH}$ = 7.9 Hz, 4H, MgCH_2); ^{13}C{^{1}H} NMR (125.8 MHz, C_6D_6, 298K): δ **13** (50%): 169.2 (s, N*C*(Me)), 143.9 (s, N*C* Dipp), 141.8 (s, *C*-^{i}Pr), 139.8 (s, *C*H=CH_2), 126.1 (*p*-*C*H Dipp), 124.1 (*m*-*C*H Dipp), 114.0 (s, CH=*C*H_2), 95.3 (s, *C*H{C(Me)NDipp}$_2$), 34.5 (s, *C*H_2CH=CH_2), 30.4 – 29.0 (s, *C*H_2), 28.8 (s, MgCH_2*C*H_2), 28.7 (s, *C*H(CH_3)$_2$ Dipp), 24.7 (s, C-*C*H_3 Dipp), 23.6 (s, CH{C(*C*H_3)NDipp}$_2$), 23.5 (s, C-*C*H_3 Dipp), 6.1 ppm (s, Mg*C*H_2); **14** (50%): 169.3 (s, N*C*(Me)), 143.9 (s, N*C* Dipp), 141.9 (s, *C*-^{i}Pr), 126.0 (*p*-*C*H Dipp), 124.1 (*m*-*C*H Dipp), 95.4 (s, *C*H{C(Me)NDipp}$_2$), 30.4 – 29.0 (s, *C*H_2), 28.7 (s, MgCH_2*C*H_2), 28.7 (s, *C*H(CH_3)$_2$ Dipp), 24.7 (s, C-*C*H_3 Dipp), 23.5 (s, C-*C*H_3 Dipp), 23.6 (s, CH{C(*C*H_3)NDipp}$_2$), 5.8 ppm (s, Mg*C*H_2). Elemental analysis (%). Found: C 78.93, H 10.55, N 5.26. Calculated for **13** ($C_{66}H_{108}Mg_2N_4$) + **14** ($C_{37}H_{56}MgN_2$): C 79.33, H 10.60, N 5.39.

13: In a sealed J. Young NMR tube, C_6D_6 (0.5 mL) was added to a mixture of $[(BDI)MgH]_2$ (20 mg, 0.024 mmol) and 1,7-octadiene (14.4 μL, 0.096 mmol). After 5 days at room temperature, the solvent was removed under vacuum (Figure S.15).

14: In a sealed J. Young NMR tube, C_6D_6 (0.5 mL) was added to a mixture of $[(BDI)MgH]_2$ (16 mg, 0.020 mmol) and 1,7-octadiene (1.5 μL, 0.010 mmol). After heating the resulting colorless solution for 2 days at 80ºC, the solvent was removed under vacuum (Figure S.16).

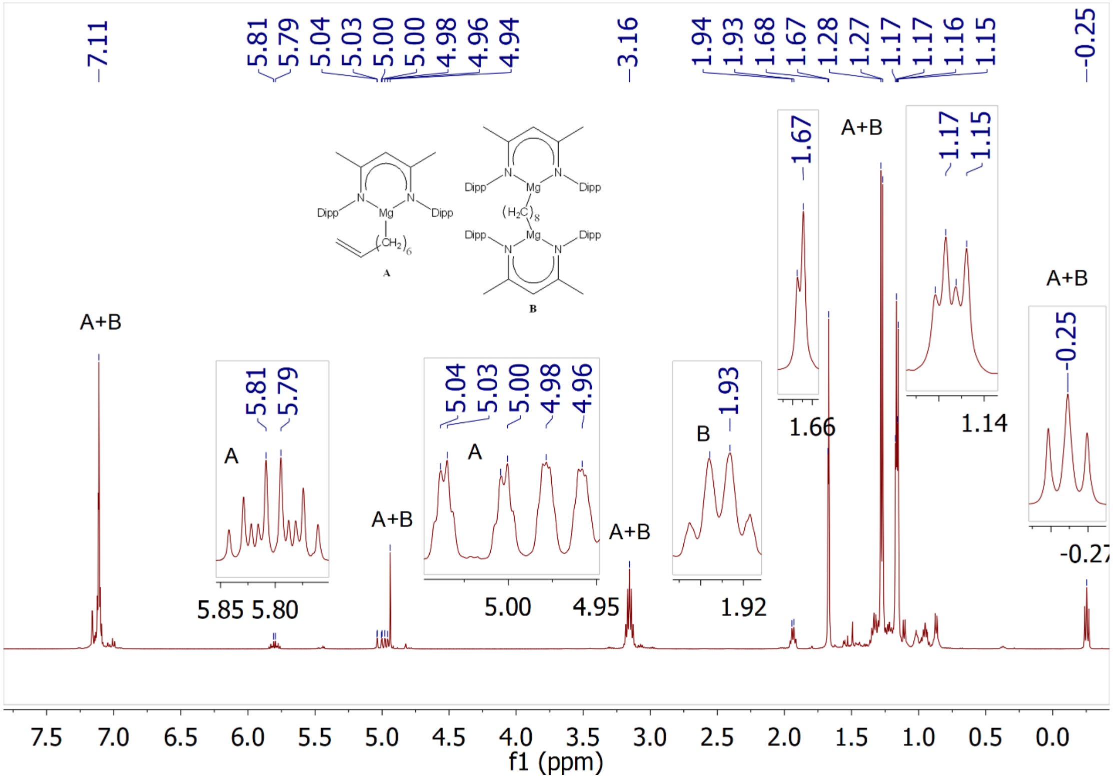

Figure S15. ^{1}H NMR spectrum (500 MHz, C_6D_6, 298K) of compounds **13** (50%) and **14** (50%).

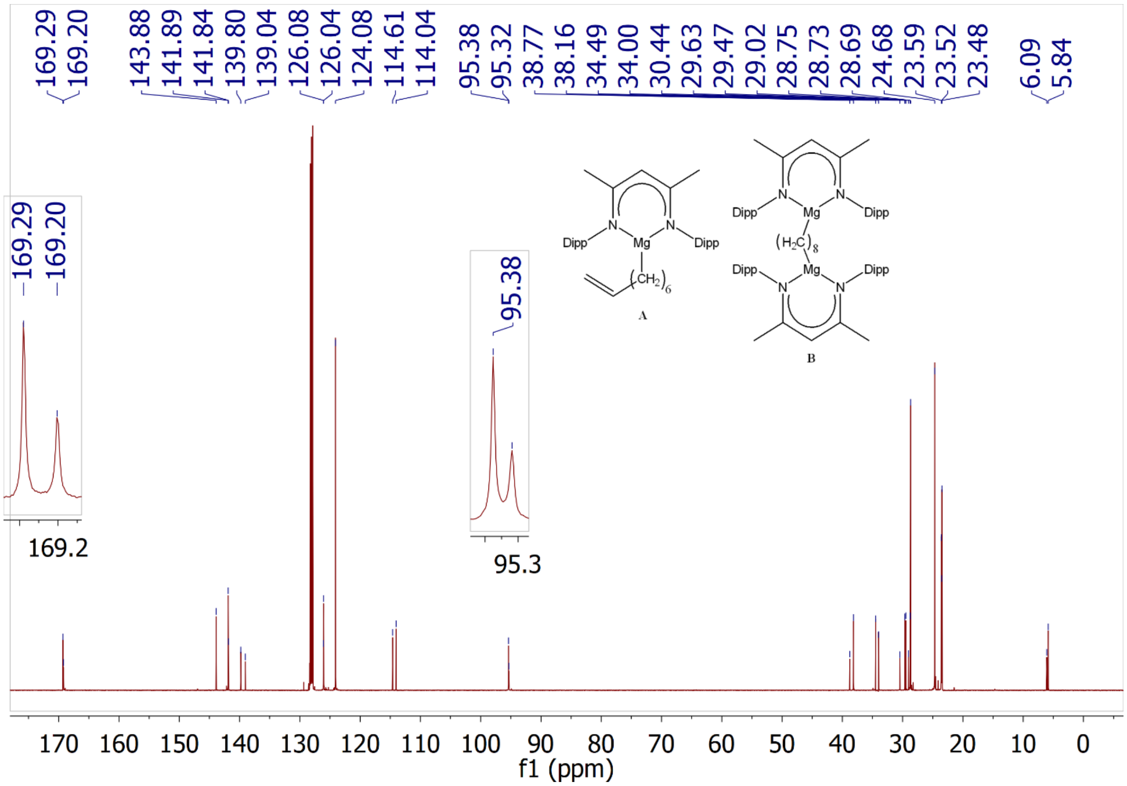

Figure S16. $^{13}C\{^{1}H\}$ NMR spectrum (125.8 MHz, C_6D_6, 298K) of compounds **13** (50%) and **14** (50%).

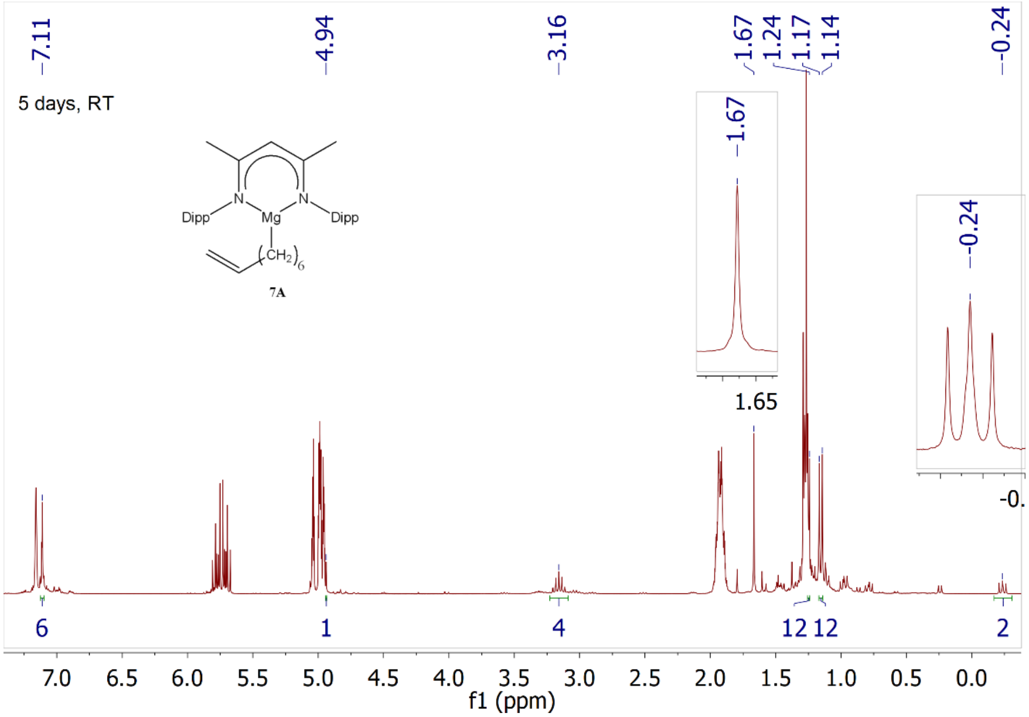

Figure S17. Stacked ^{1}H NMR spectra (500 MHz, C_6D_6, 298K) of reaction between [(BDI)MgH]$_2$ (0.024 mmol) and 1,7-octadiene (0.096 mmol) at RT after 5 days. Assignment of major species: 1,7-octadiene (CH_2-CH=CH_2 = 5.74 ppm). **13** (BDI methine = 4.94).

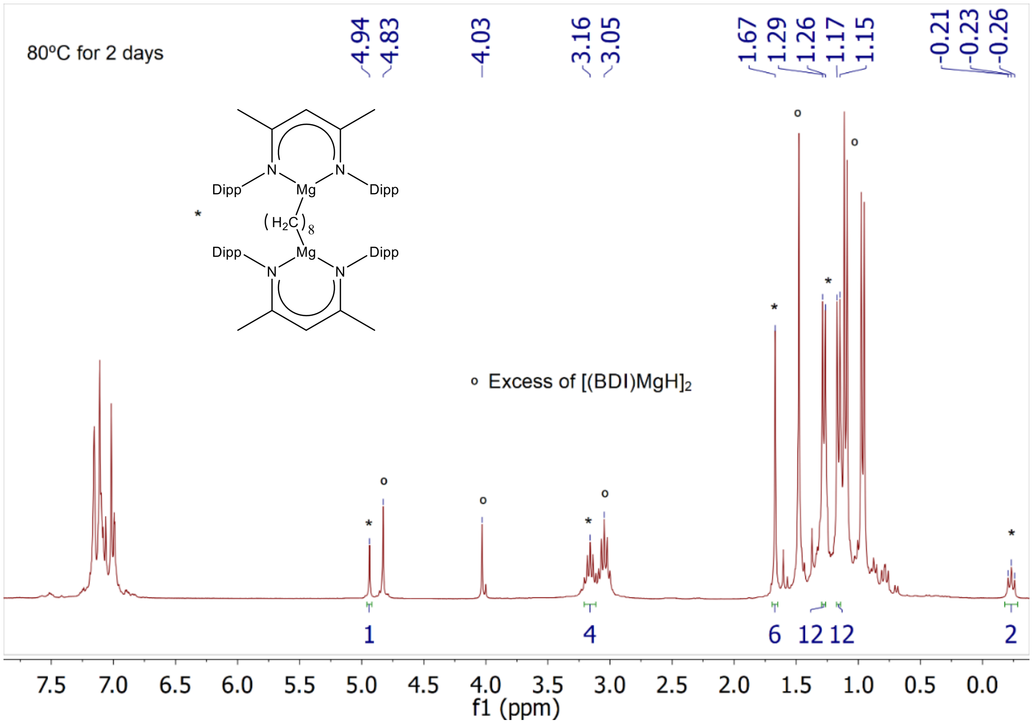

Figure S18. Stacked ^{1}H NMR spectra (500 MHz, C_6D_6, 298K) of reaction between $[(BDI)MgH]_2$ (0.020 mmol) and 1,7-octadiene (0.010 mmol) at 80ºC after 2 days. Assignment of major species: $[(BDI)MgH]_2$: (BDI methine = 4.83, MgH = 4.03 ppm) in 1:1 ratio. **14** (BDI methine = 4.94 ppm).

[(BDI)Mg{$CHCH_2CHCH_2CH(CH_2)_2$)}] (15)

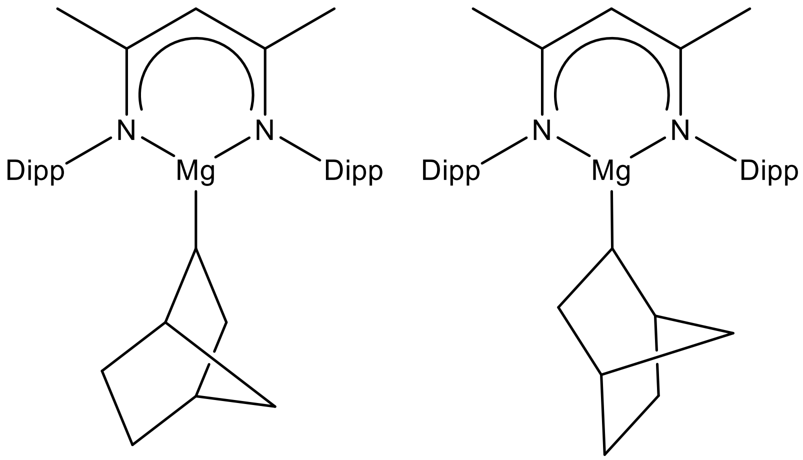

[(BDI)MgH]$_2$ (20 mg, 0.024 mmol) and norbornene (4.6 mg, 0.048 mmol) were dissolved in C_6D_6 (0.5 mL) in a sealed J. Young NMR tube. After heating the resulting colorless solution for 3 days at 80 ºC, the solvent was removed under vacuum. Colourless single crystals of **15** suitable for X-ray crystallography were grown by slow evaporation of a saturated hexane solution at room temperature. Yield: 24 mg, 92%. ^{1}H NMR (500 MHz, C_6D_6, 298K): δ 7.13 – 7.11 (6H, C*H* Dipp), 4.93 (s, 1H, C*H*{C(CH_3)NDipp}$_2$), 3.15 (sept, $^3J_{HH}$ = 6.9 Hz, 2H, C*H*(CH_3)$_2$), 3.14 (sept, $^3J_{HH}$ = 6.9 Hz, 2H, C*H*(CH_3)$_2$), 2.20 – 2.17 (m, 1H, C*H*$_2$), 1.82 – 1.80 (m, 1H, MgCHCH$_2$C*H*), 1.70 – 1.00 (m, 5H, C*H*$_2$), 1.67 (s, 6H, CH{C(C*H*$_3$)NDipp}$_2$), 1.42 – 1.32 (m, 1H, MgCHC*H*), 1.32 (d, $^3J_{HH}$ = 6.9 Hz, 6H, CH(C*H*$_3$)$_2$), 1.31 (d, $^3J_{HH}$ = 6.9 Hz, 6H, CH(C*H*$_3$)$_2$), 1.15 (d, $^3J_{HH}$ = 6.9 Hz, 12H, CH(C*H*$_3$)$_2$), 1.18 – 1.13 (m, 1H, MgCHC*H*$_2$), 1.02 – 0.99 (m, 1H, MgCHC*H*$_2$), -0.14 ppm (ddd, $^3J_{HH}$ = 9.9, 7.3, 2.4 Hz, 1H, MgC*H*); ^{13}C{^{1}H} NMR (125.8 MHz, C_6D_6, 298K): δ 169.3 (s, N*C*(Me)), 144.4 (s, N*C* Dipp), 141.8 (s, *C*-^{i}Pr), 141.8 (s, *C*-^{i}Pr), 126.0 (*p*-*C*H Dipp), 124.1 (*m*-*C*H Dipp), 124.1 (*m*-*C*H Dipp), 95.3 (s, *C*H{C(Me)NDipp}$_2$), 40.9 (s, MgCH*C*H$_2$), 40.7 (s, MgCHCH$_2$*C*H), 38.7 (s, *C*H$_2$), 37.4 (s, *C*H$_2$), 35.8 (s, MgCH*C*H), 30.6 (s, *C*H$_2$), 28.8 (s, C*H*(CH_3)$_2$ Dipp), 24.6, 24.5 (s, C-*C*H$_3$ Dipp), 24.1 (s, Mg-*C*H), 23.6, 23.6 (s, C-*C*H$_3$ Dipp), 23.5 ppm (s, CH{C(*C*H$_3$)NDipp}$_2$). Elemental analysis (%). Found: C 79.02, H 9.53, N 5.16. Calculated for $C_{36}H_{52}MgN_2$: C 80.50, H 9.76, N 5.22.

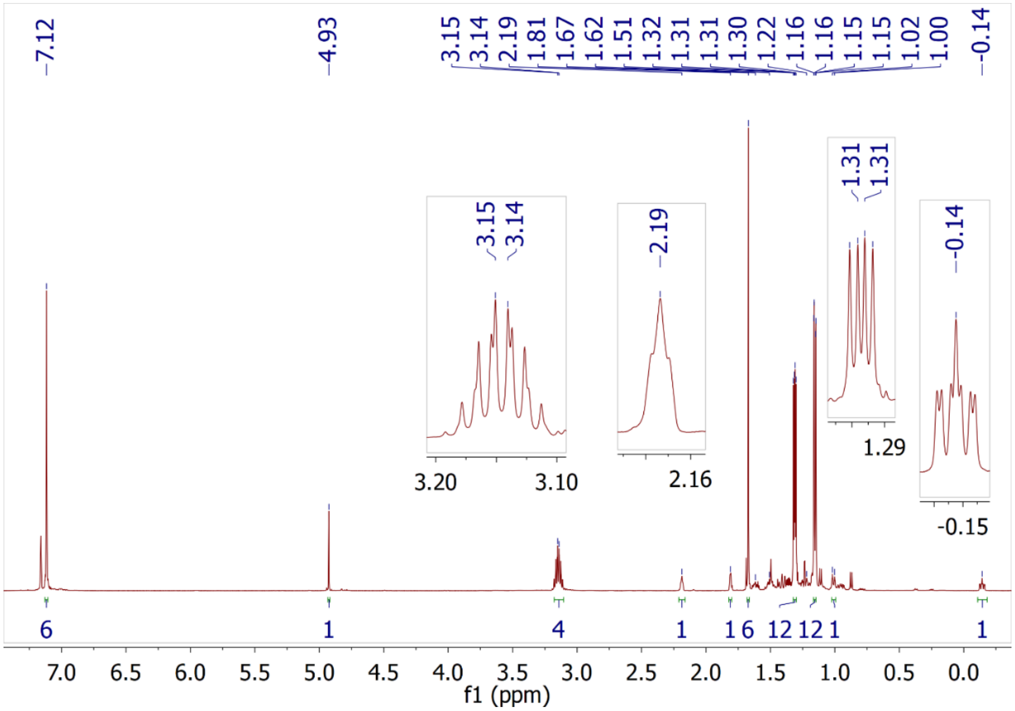

Figure S19. ^{1}H NMR spectrum (500 MHz, C_6D_6, 298K) of compound **15**.

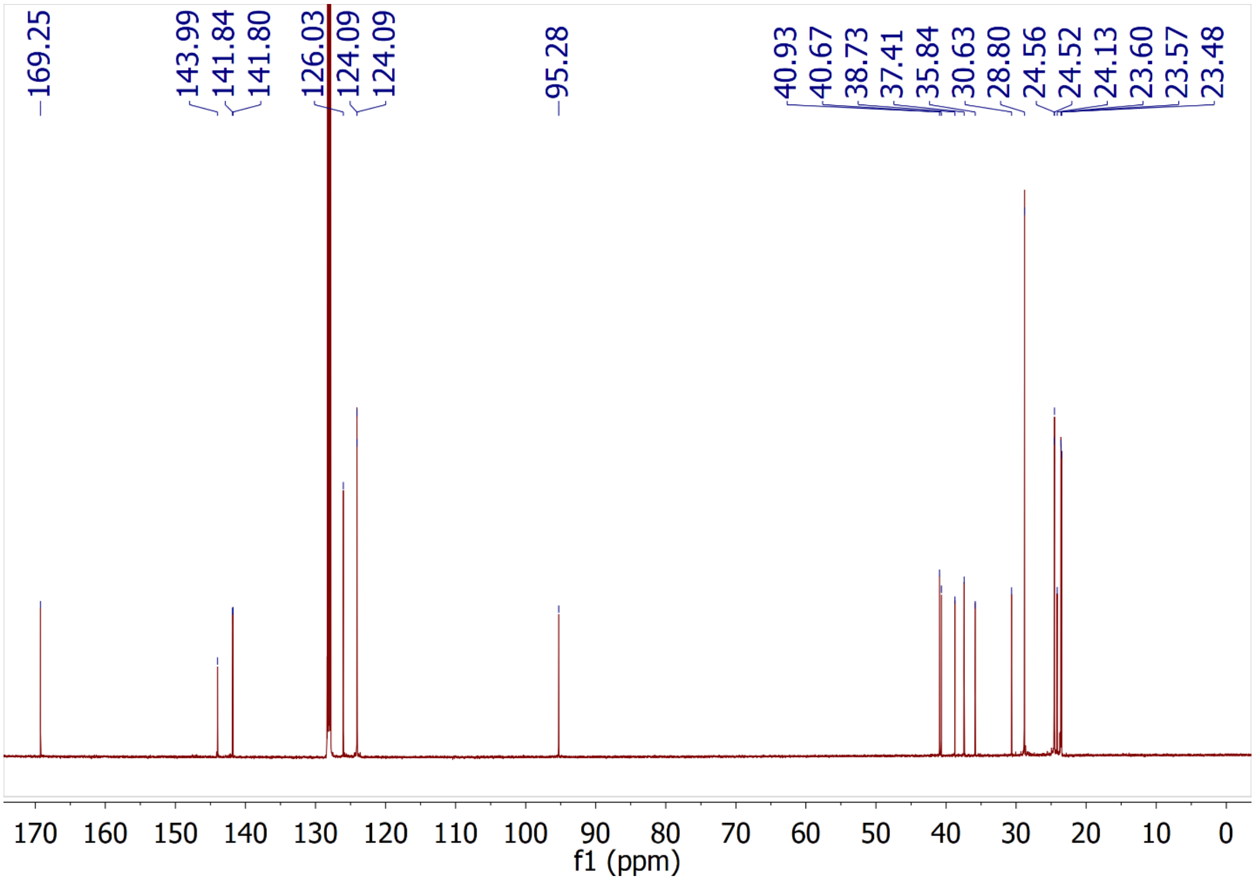

Figure S20. $^{13}C\{^{1}H\}$ NMR spectrum (125.8 MHz, C_6D_6, 298K) of compound **15**.

Stoichiometric insertion of 1-Hexene and norbornene into the Mg-H bond of $[(BDI)MgH]_2$. Possible observation of the dimeric hydride complexes.

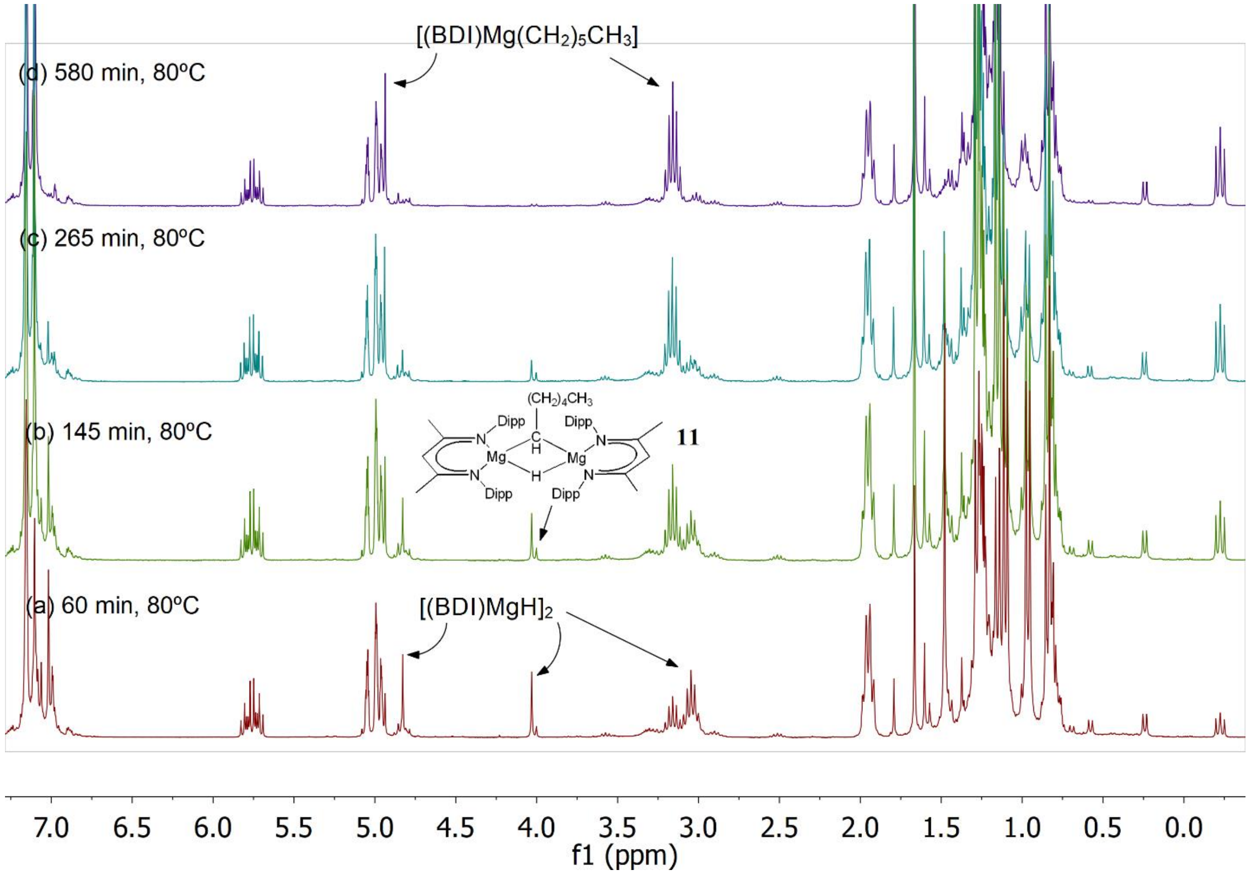

Figure S21. Stacked ^{1}H NMR spectra (300 MHz, C_6D_6, 298K) of reaction between $[(BDI)MgH]_2$ (0.012 mmol) and 1-hexene (0.064 mmol) in C_6D_6 at 80ºC after (a) 60, (b) 145, (c) 265 and (d) 580 min. Assignment of major species: $[(BDI)MgH]_2$: (BDI methine = 4.84, Mg*H* = 4.03 ppm) in 1:1 ratio. **4**: (BDI methine = 4.94 ppm). 1-Hexene: (C*H*=CH$_2$ = 5.76 ppm). Dimagnesium hydrido-hexyl species (Mg*H* = 4.00 ppm).

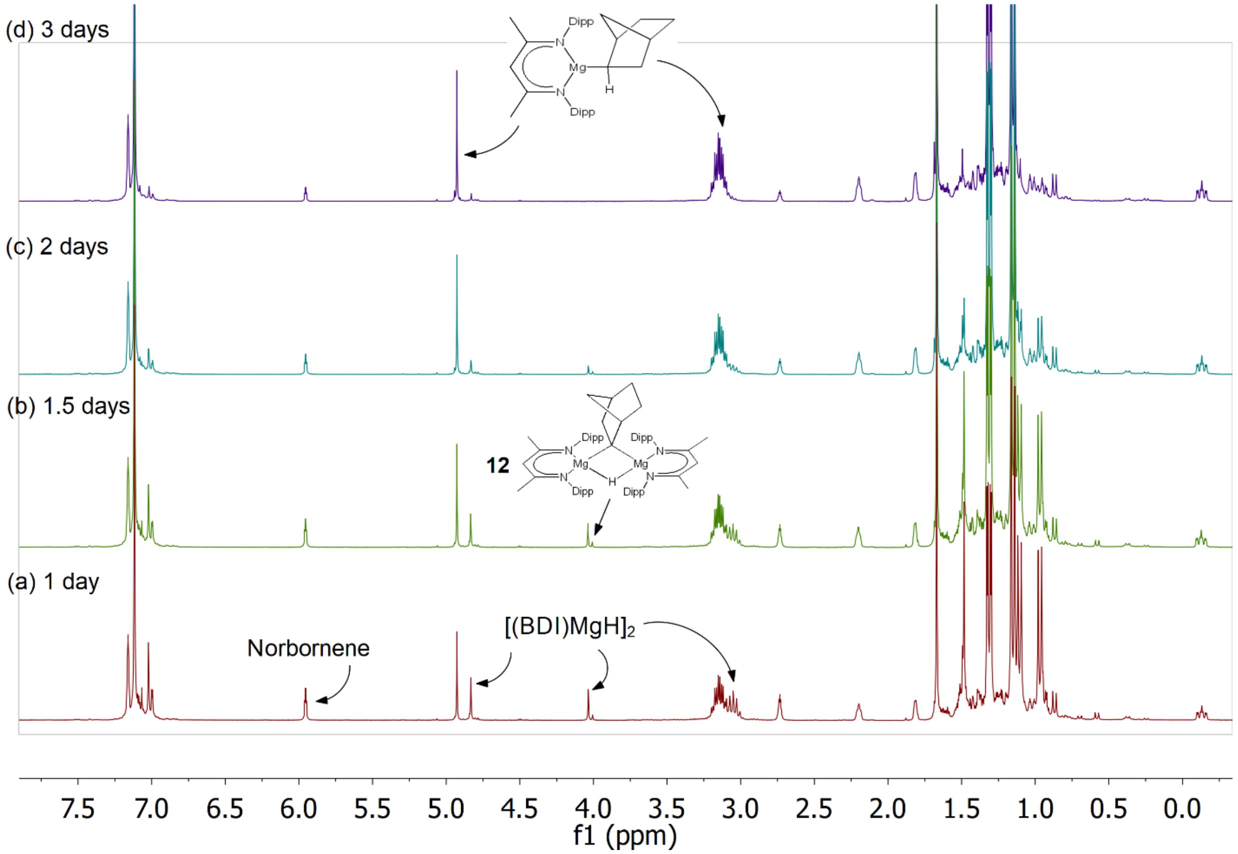

Figure S22. Stacked ^{1}H NMR spectra (300 MHz, C_6D_6, 298K) of reaction between $[(BDI)MgH]_2$ (0.012 mmol) and norbornene (0.024 mmol) in C_6D_6 at 80ºC after (a) 1, (b) 1.5, (c) 2 and (d) 3 days. Assignment of major species: $[(BDI)MgH]_2$: (BDI methine = 4.84, Mg*H* = 4.03 ppm) in 1:1 ratio. **15**: (BDI methine = 4.93 ppm). Norbornene: (=C*H* = 5.95 ppm). Dimagnesium hydrido-norbornyl species: (Mg*H* = 4.01 ppm).

[(BDI)Mg{C(Ph)=CHPh}] (16)

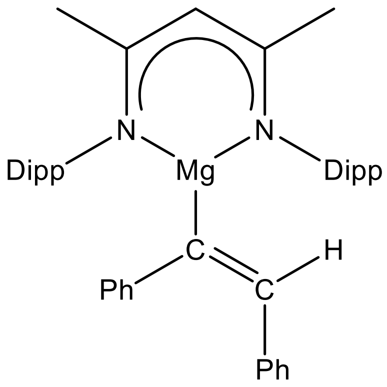

In a sealed J. Young NMR tube, C_6D_6 (0.5 mL) was added to a mixture of $[(BDI)MgH]_2$ (20 mg, 0.024 mmol) and diphenylacetylene (8.7 mg, 0.048 mmol). After heating the resulting colourless solution for 6 days at 80 ºC, the solvent was removed under vacuum. Yield: 28 mg, 92%. ^{1}H NMR (500 MHz, C_6D_6, 298K): δ 7.54 – 7.50 (1H, C*H* Ph), 7.26 – 6.90 (6H, C*H* Dipp and Ph), 6.87 – 6.77 (2H, C*H* Ph), 6.47 – 6.40 (m, 2H, *o*-C*H* Ph), 5.85 (s, 1H, =C*H*Ph), 4.93 (s, 1H, C*H*{C(CH_3)NDipp}$_2$), 3.11 (sept, $^{3}J_{HH}$ = 6.9 Hz, 4H, C*H*(CH_3)$_2$), 1.67 (s, 6H, CH{C(CH_3)NDipp}$_2$), 1.17 (d, $^{3}J_{HH}$ = 6.9 Hz, 12H, CH(CH_3)$_2$), 1.14 ppm (d, $^{3}J_{HH}$ = 6.9 Hz, 12H, CH(CH_3)$_2$). ^{13}C{^{1}H} NMR (125.8 MHz, C_6D_6, 298K): δ 170.2 (s, Mg*C*), 169.8 (s, N*C*(Me)), 152.0 (s, *C* Ph), 143.8 (s, N*C* Dipp), 141.9 (s, *C*-^{i}Pr), 139.8 (s, *C* Ph), 139.3 (s, =*C*HPh), 132.0 (s, *m*-*C*H Ph), 129.6 (s, *o*-*C*H Ph), 128.7 – 126.2 (*C*H Dipp), 125.6 (s, *C*H Ph), 125.1 (s, *o*-*C*H Ph), 124.5 (*m*-*C*H Dipp), 122.2 (*C*H Dipp), 95.3 (s, *C*H{C(Me)NDipp}$_2$), 28.8 (s, *C*H(CH_3)$_2$ Dipp), 24.5, 23.5 (s, C-*C*H_3 Dipp), 23.4 ppm (s, CH{C(*C*H_3)NDipp}$_2$). Elemental analysis (%). Found: C 83.23, H 8.43, N 4.54. Calculated for $C_{43}H_{52}MgN_2$: C 83.14, H 8.44, N 4.51.

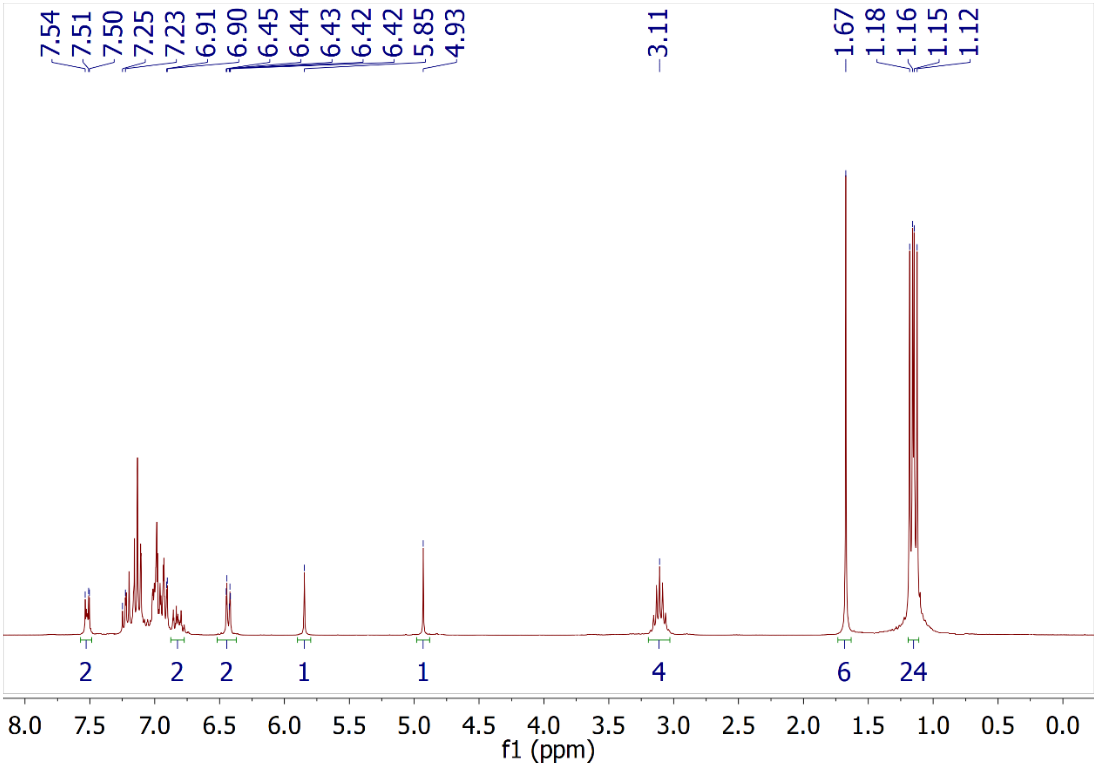

Figure S23. ^{1}H NMR spectrum (500 MHz, C_6D_6, 298K) of compound **16**.

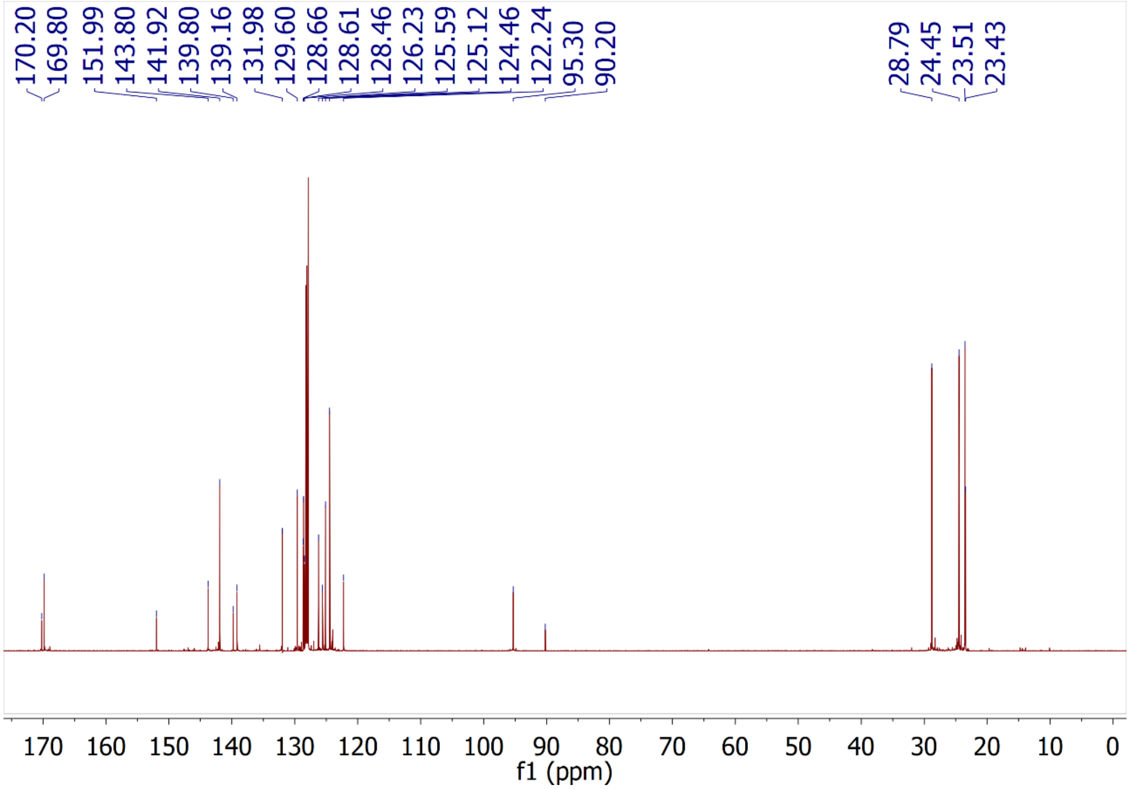

Figure S24. $^{13}C\{^1H\}$ NMR spectrum (125.8 MHz, C_6D_6, 298K) of compound **16**.

General Method for Catalytic Hydrosilylation mediated by [(BDI)MgH]$_2$

Catalyst, [(BDI)MgH]$_2$ (5 mol%), substrate (0.010 mmol) and phenylsilane (0.025 mmol) were dissolved in C_6D_6 (0.4 mL) in a J. Young NMR tube, and the resulting solution heated at 80 ºC. The reactions were monitored via ^{1}H NMR spectroscopy with their progress evidenced by the consumption of the olefinic resonances alongside the emergence of the respective SiH_2 resonances.

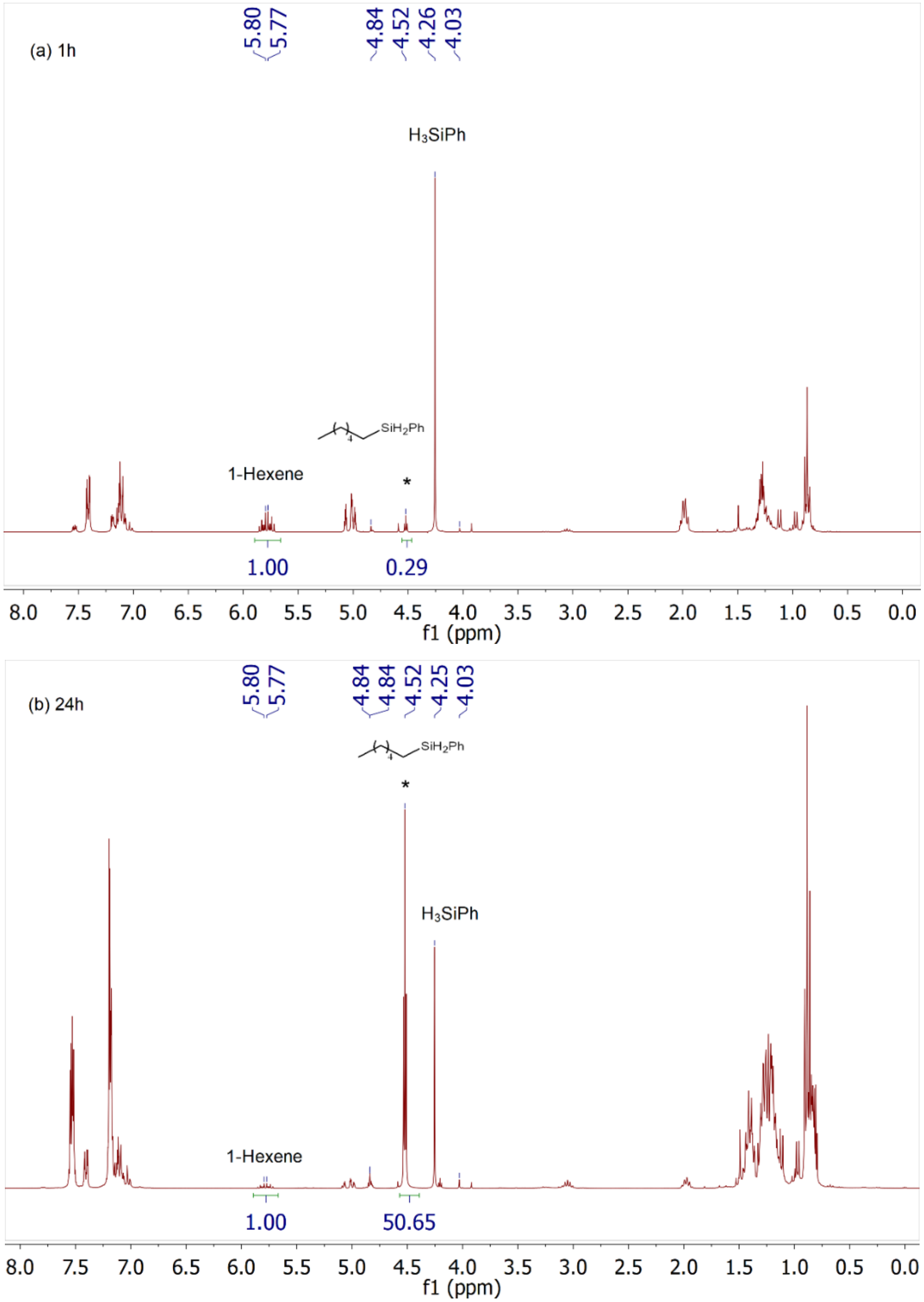

Figure S25. ^{1}H NMR spectra (300 MHz) of catalytic hydrosilylation of 1-hexene (0.338 mmol) mediated by $[(BDI)MgH]_2$ (10%) in C_6D_6 (0.4 mL) after (a) 1 hour and (b) 24 hours at 80ºC. Assignment of major species: $[(BDI)MgH]_2$: (BDI methine = 4.84, MgH = 4.03 ppm) in 1:1 ratio. H_3SiPh: (H_3Si = 4.25 ppm). 1-Hexene: (CH=CH_2 = 5.78 ppm). $PhH_2Si(CH_2)_5CH_3$: (H_2Si = 4.52 ppm).

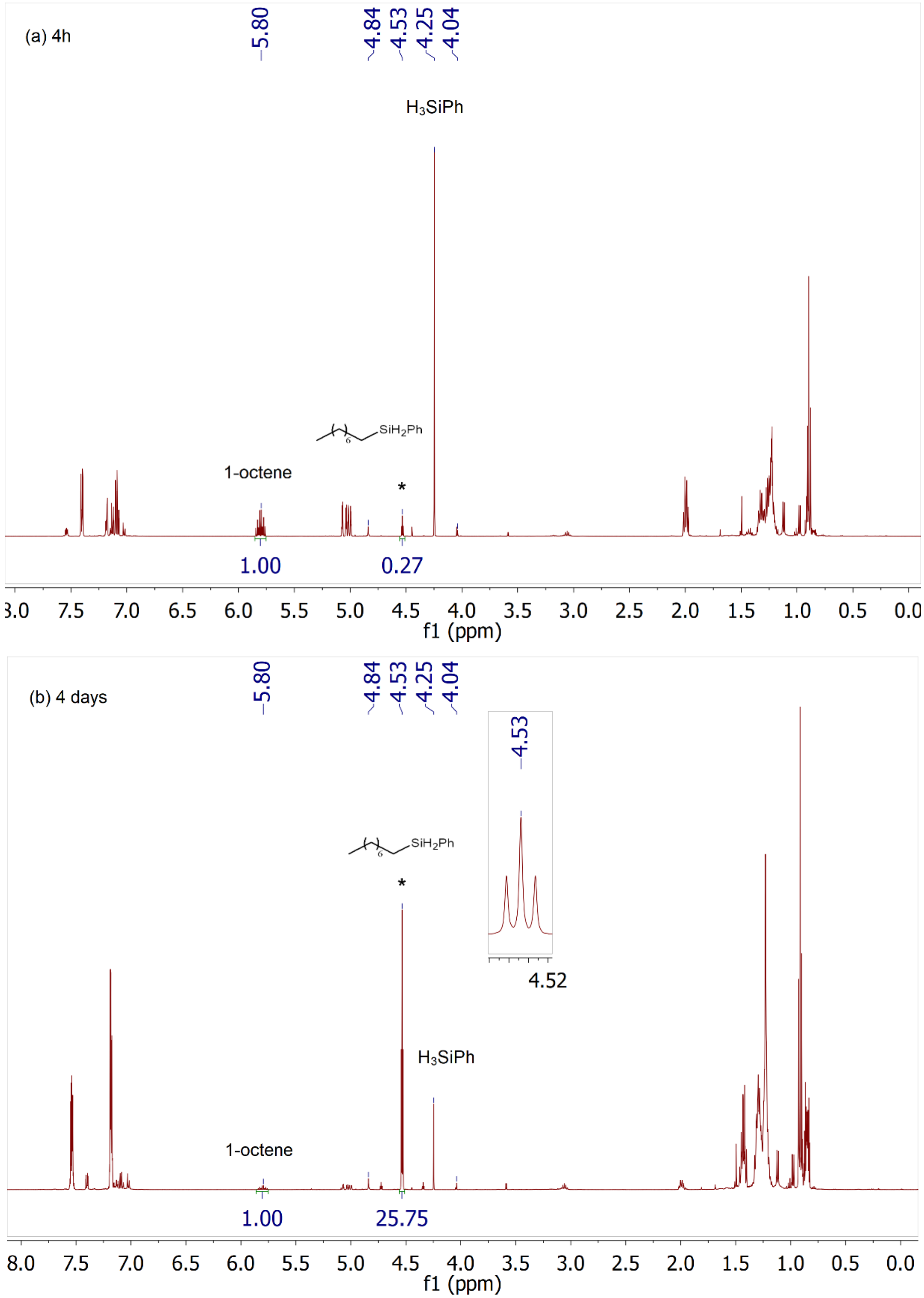

Figure S26. ^{1}H NMR spectra (300 MHz) of catalytic hydrosilylation of 1-octene (0.108 mmol) mediated by $[(BDI)MgH]_2$ (5%) in C_6D_6 (0.4 mL) after (a) 4 hours and (b) 4 days at 80ºC. Assignment of major species: $[(BDI)MgH]_2$: (BDI methine = 4.84, MgH = 4.04 ppm) in 1:1 ratio. H_3SiPh: (H_3Si = 4.25 ppm). 1-Octene: (CH=CH$_2$ = 5.80 ppm). $PhH_2Si(CH_2)_7CH_3$: (H_2Si = 4.53 ppm).

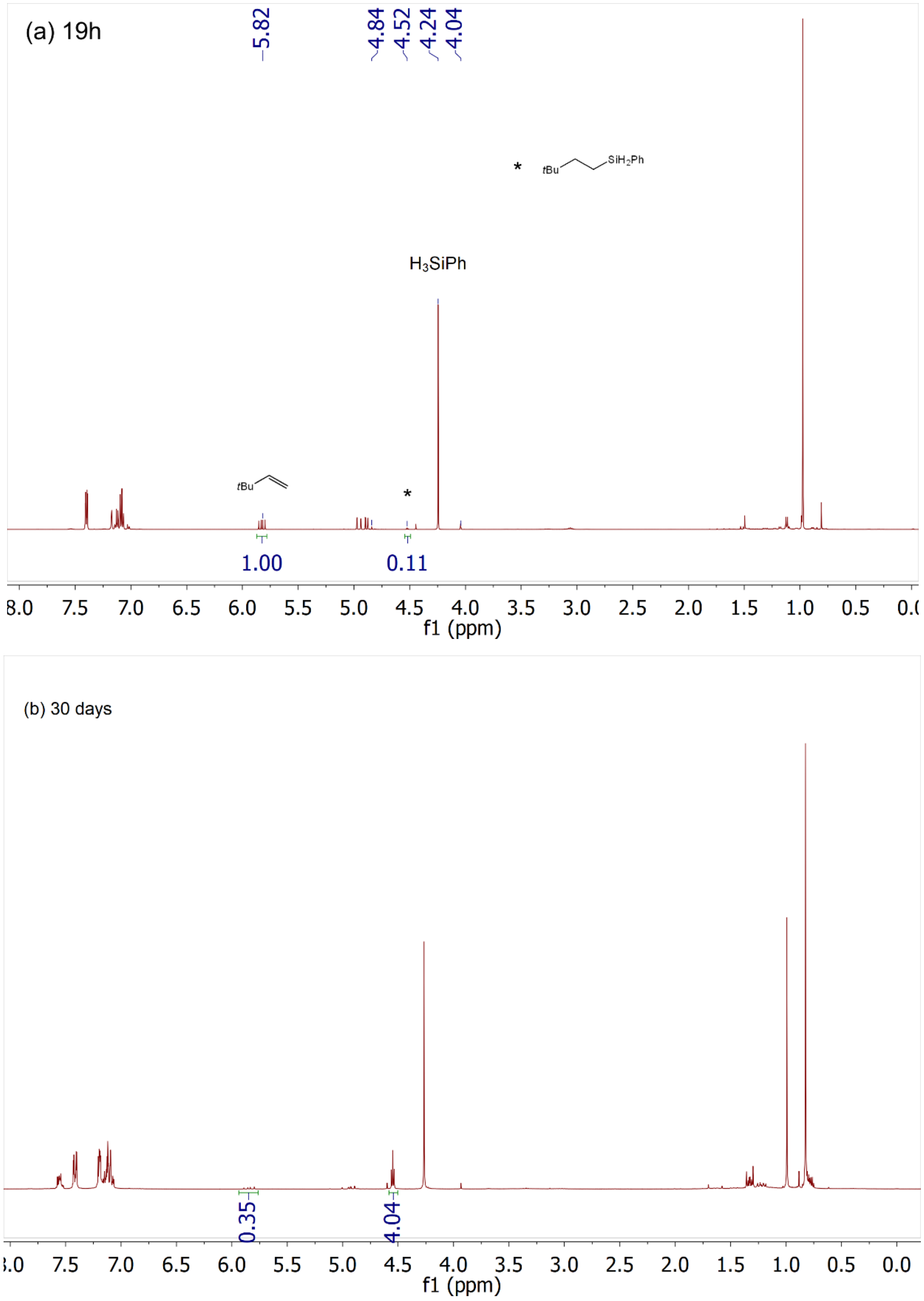

Figure S27. ^{1}H NMR spectra (300 MHz) of catalytic hydrosilylation of 3,3-dimethyl-1-butene (0.106 mmol) mediated by [(BDI)MgH]$_2$ (5%) in C_6D_6 (0.4 mL) after (a) 19 hours and (b) 30 days at 80ºC. Assignment of major species: [(BDI)MgH]$_2$: (BDI methine = 4.84, Mg*H* = 4.04 ppm) in 1:1 ratio. H_3SiPh: (H_3Si = 4.24 ppm). 3,3-dimethyl-1-butene: (C*H*=CH = 5.82 ppm). *t*Bu(CH_2)$_2$SiH_2Ph: (H_2Si = 4.52 ppm).

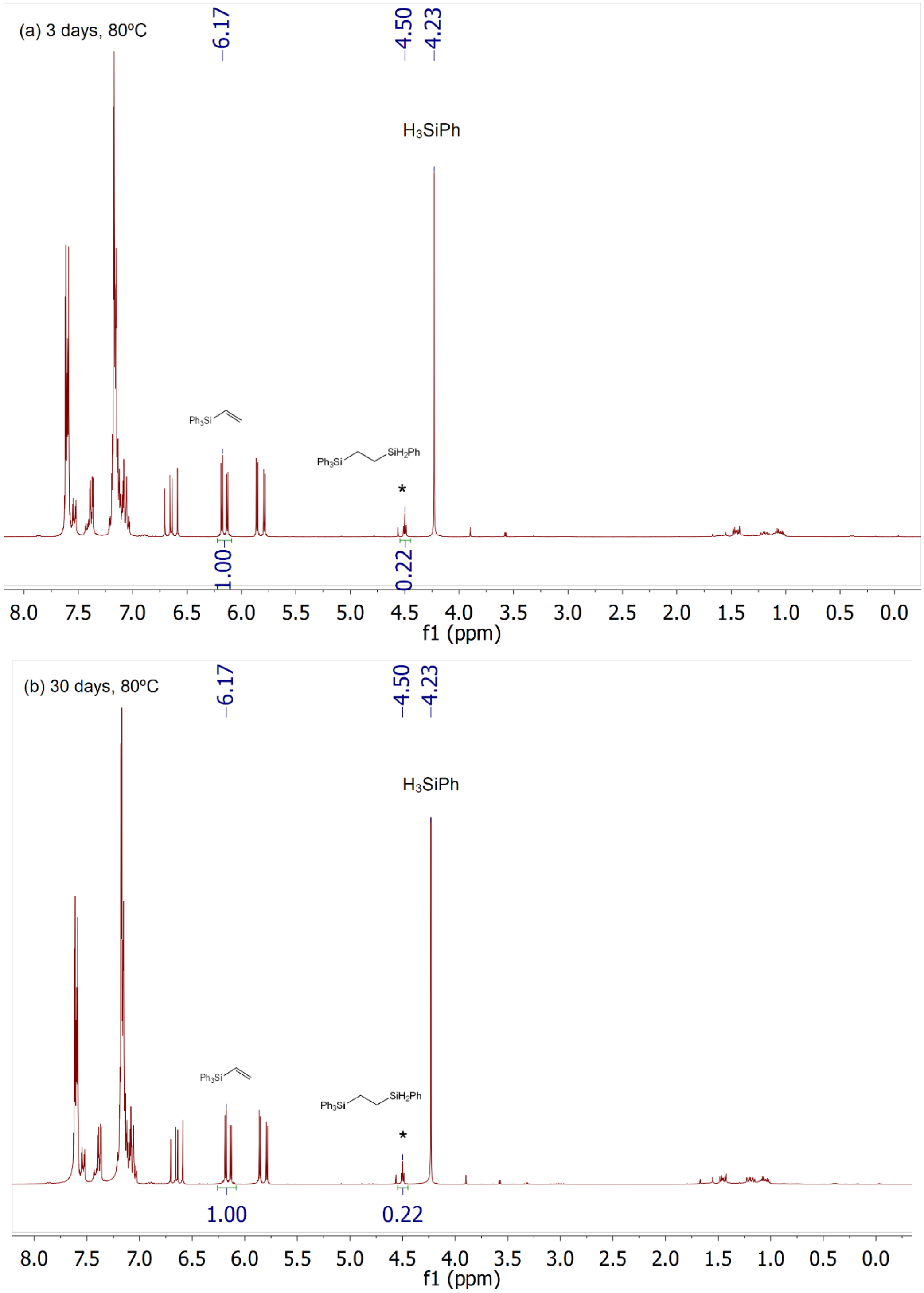

Figure S28. ^{1}H NMR spectra (300 MHz) of catalytic hydrosilylation of allyltriphenylsilane (0.107 mmol) mediated by [(BDI)MgH]$_2$ (5%) in C_6D_6 (0.4 mL) after (a) 3 days and (b) 30 days at 80ºC. Assignment of major species: H_3SiPh: (H_3Si = 4.23 ppm). Allyltriphenylsilane: (CH=CH_2 = 6.17 ppm). $Ph_3Si(CH_2)_2SiH_2Ph$: (H_2Si = 4.50 ppm).

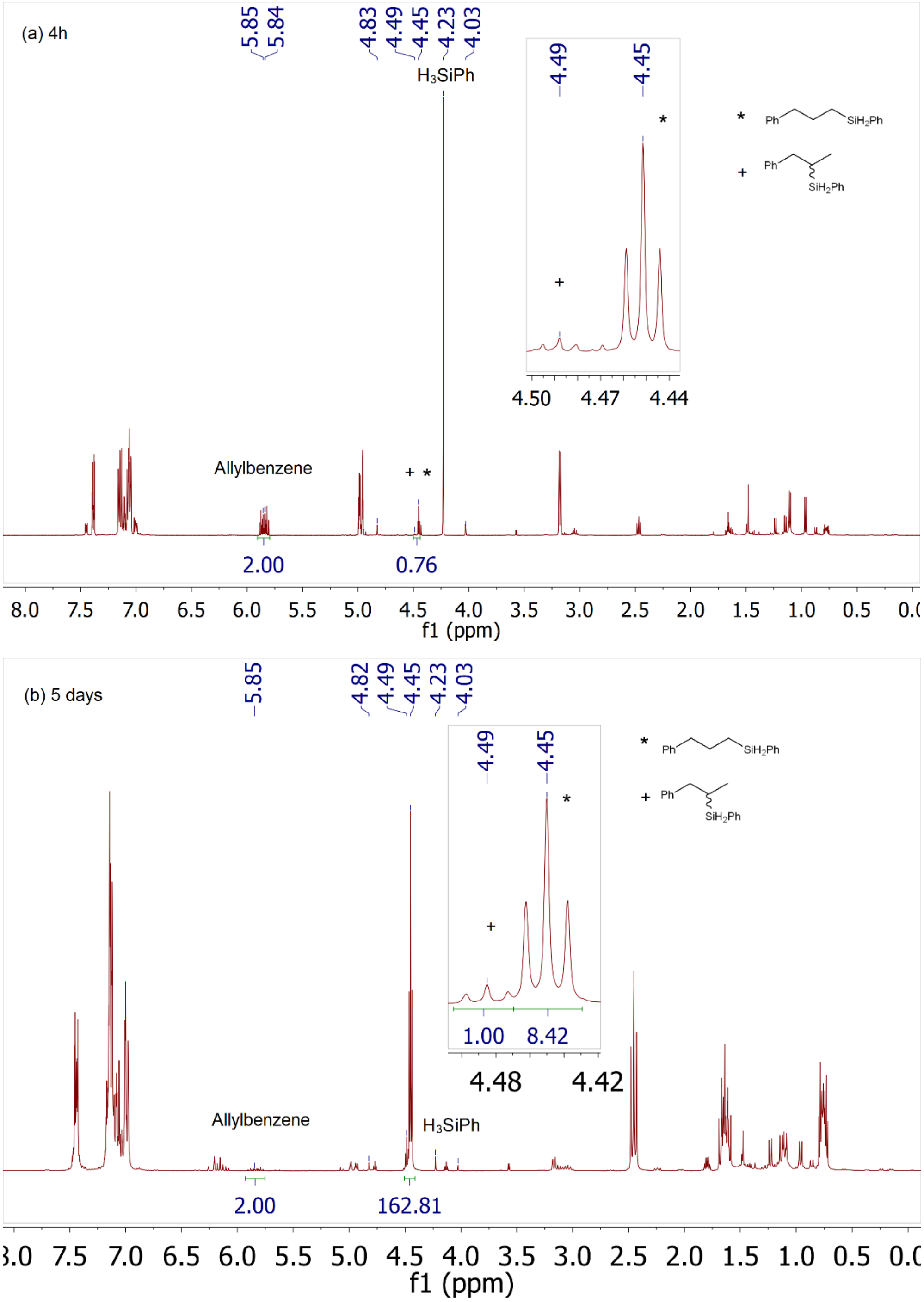

Figure S29. ^{1}H NMR spectra (300 MHz) of catalytic hydrosilylation of allylbenzene (0.111 mmol) mediated by $[(BDI)MgH]_2$ (5%) in C_6D_6 (0.4 mL) after (a) 4 hours and (b) 5 days at 80ºC. Assignment of major species: $[(BDI)MgH]_2$: (BDI methine = 4.83, Mg*H* = 4.03 ppm) in 1:1 ratio. H_3SiPh: (H_3Si = 4.23 ppm). Allylbenzene: (C*H*=CH_2 = 5.85 ppm). $Ph(CH_2)_3SiH_2Ph$: (H_2Si = 4.45 ppm). $PhCH_2CH(SiH_2Ph)CH_3$: (H_2Si = 4.49 ppm).

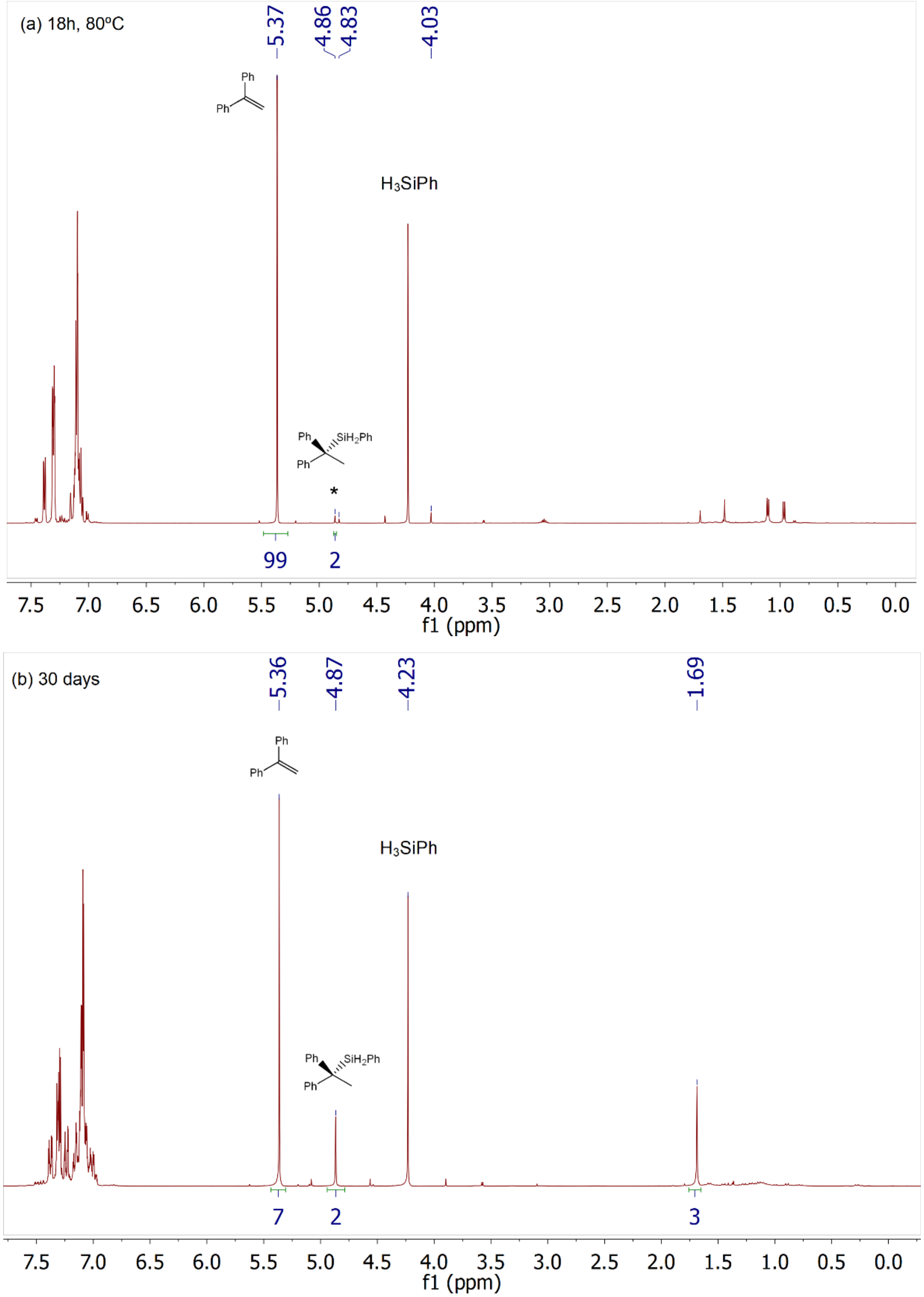

Figure S30. ^{1}H NMR spectra (300 MHz) of catalytic hydrosilylation of 1,1-diphenylethylene (0.107 mmol) mediated by $[(BDI)MgH]_2$ (5%) in C_6D_6 (0.4 mL) after (a) 18 h and (b) 30 days at 80ºC. Assignment of major species: $[(BDI)MgH]_2$: (BDI methine = 4.83, MgH = 4.03 ppm) in 1:1 ratio. H_3SiPh: (H_3Si = 4.23 ppm). Diphenylethylene: (CH_2 = 5.37 ppm). $Ph_2C(SiH_2Ph)CH_3$: (H_2Si = 4.87 ppm).

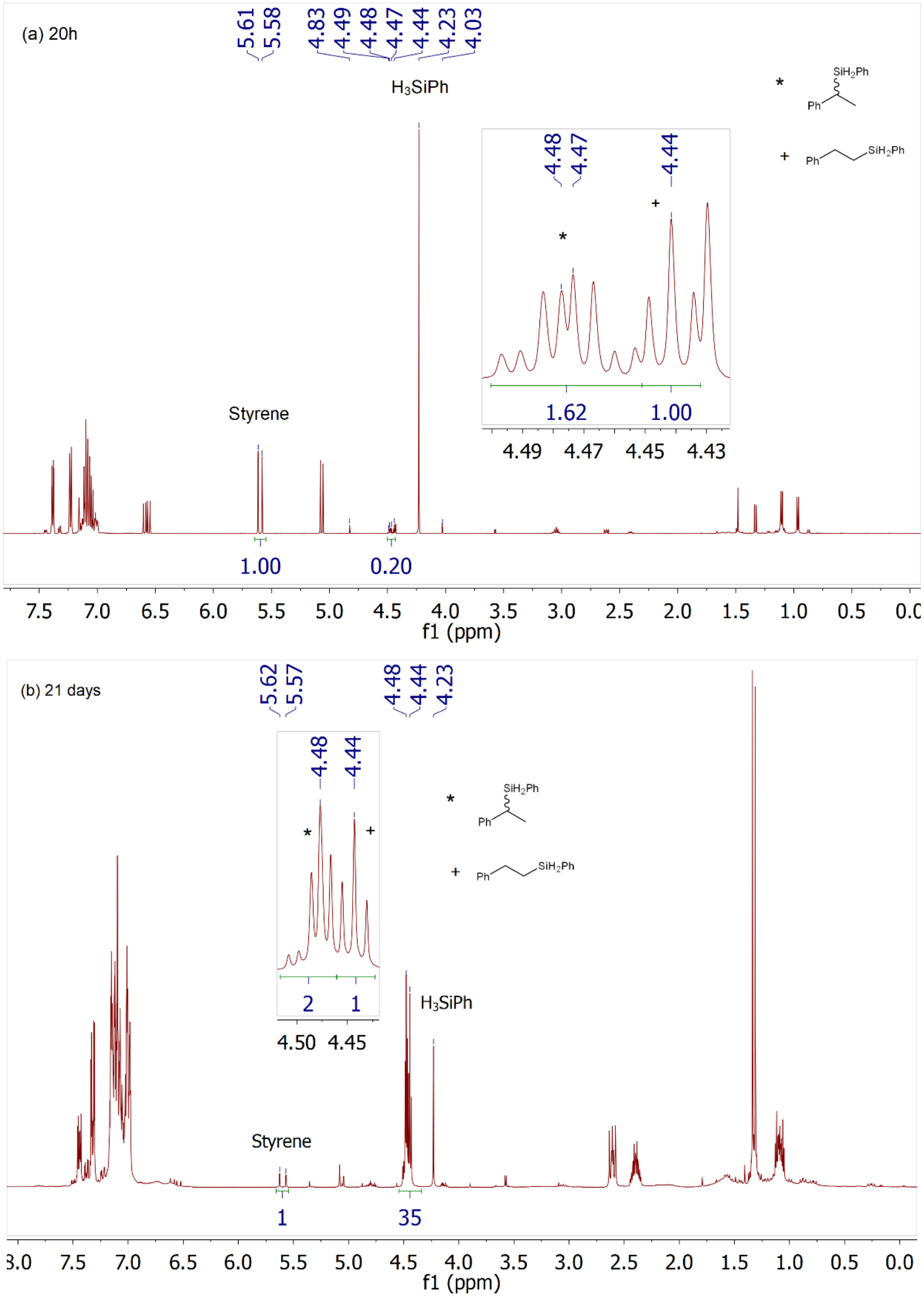

Figure S31. ^{1}H NMR spectra (300 MHz) of catalytic hydrosilylation of styrene (0.106 mmol) mediated by $[(BDI)MgH]_2$ (5%) in C_6D_6 (0.4 mL) after (a) 20 hours and (b) 21 days at 80ºC. Assignment of major species: $[(BDI)MgH]_2$: (BDI methine = 4.83, Mg*H* = 4.03 ppm) in 1:1 ratio. H_3SiPh: (H_3Si = 4.23 ppm). Styrene: (C*H*=CH_2 = 5.60 ppm). $PhCH(SiH_2Ph)CH_3$: (H_2Si = 4.48 ppm). $Ph(CH_2)_2SiH_2Ph$: (H_2Si = 4.44 ppm).

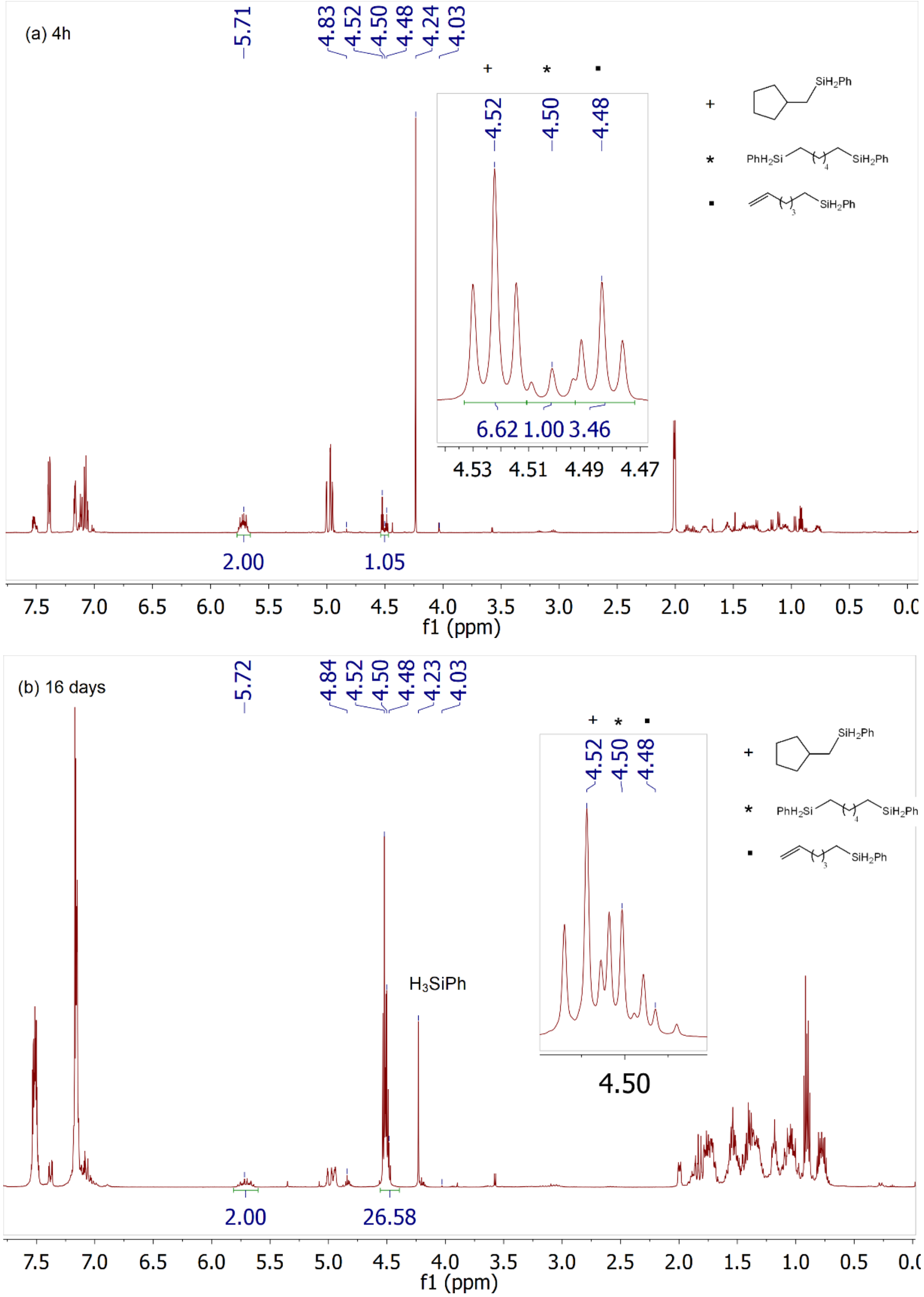

Figure S32. ^{1}H NMR spectra (300 MHz) of catalytic hydrosilylation of 1,5-hexadiene (0.109 mmol) mediated by $[(BDI)MgH]_2$ (5%) in C_6D_6 (0.4 mL) after (a) 4 hours and (b) 16 days at 80ºC. Assignment of major species: $[(BDI)MgH]_2$: (BDI methine = 4.84, Mg*H* = 4.03 ppm) in 1:1 ratio. H_3SiPh: (H_3Si = 4.24 ppm). 1,5-Hexadiene: (C*H*=CH_2 = 5.72 ppm). $(CH_2)_4CHCH_2SiH_2Ph$: (H_2Si = 4.52 ppm). $PhH_2Si(CH_2)_6SiH_2Ph$: (H_2Si = 4.50 ppm). CH_2=$CH(CH_2)_4SiH_2Ph$: (H_2Si = 4.48 ppm).

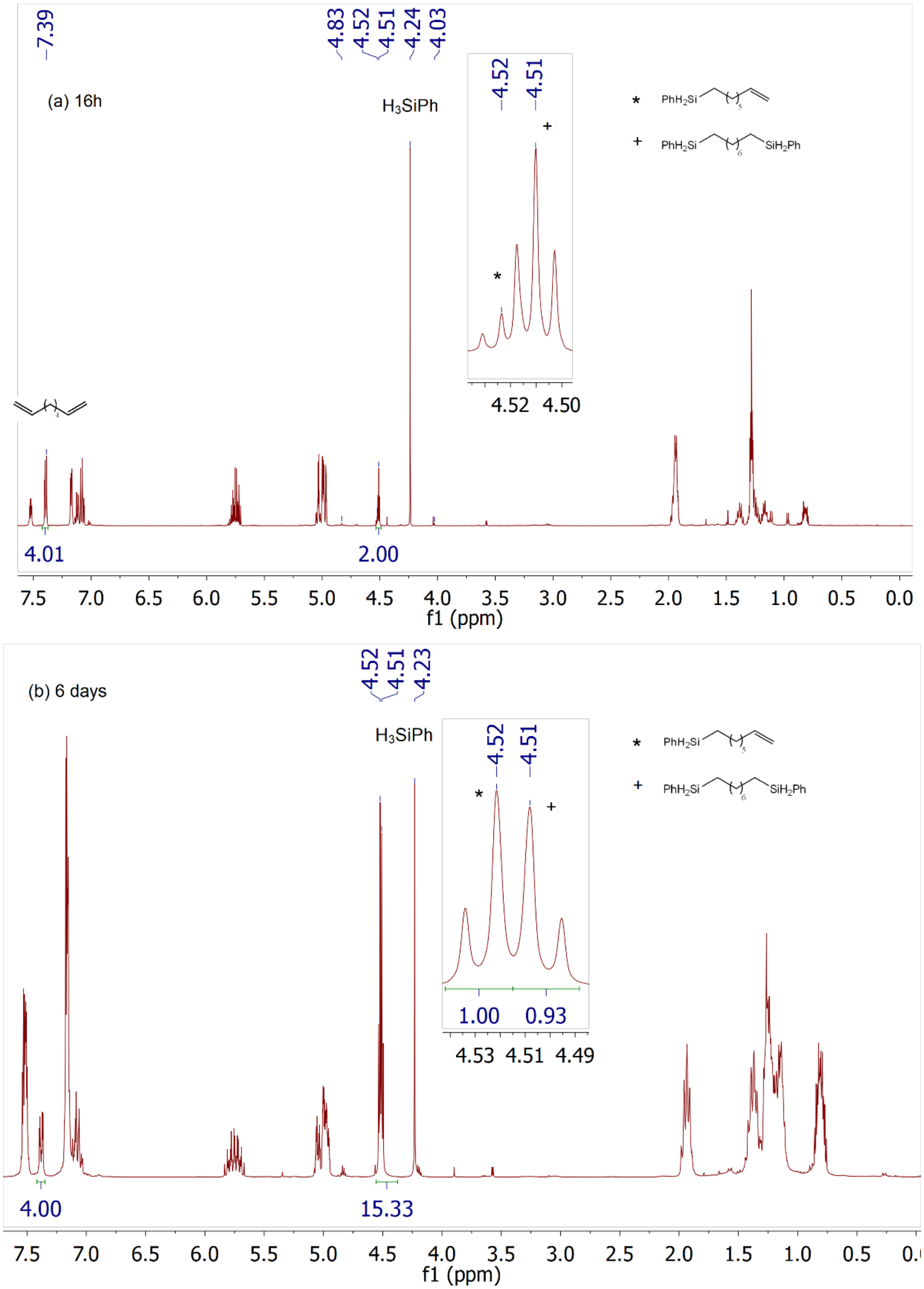

Figure S33. ^{1}H NMR spectra (300 MHz) of catalytic hydrosilylation of 1,7-octadiene (0.106 mmol) mediated by $[(BDI)MgH]_2$ (5%) in C_6D_6 (0.4 mL) after (a) 16 hours and (b) 6 days at 80ºC. Assignment of major species: $[(BDI)MgH]_2$: (BDI methine = 4.83, Mg*H* = 4.03 ppm) in 1:1 ratio. H_3SiPh: (H_3Si = 4.24 ppm). 1,7-octadiene: (C*H*=CH_2 = 7.39 ppm). $PhH_2Si(CH_2)_6CH{=}CH_2$: (H_2Si = 4.52 ppm). $PhH_2Si(CH_2)_8SiH_2Ph$: (H_2Si = 4.51 ppm).

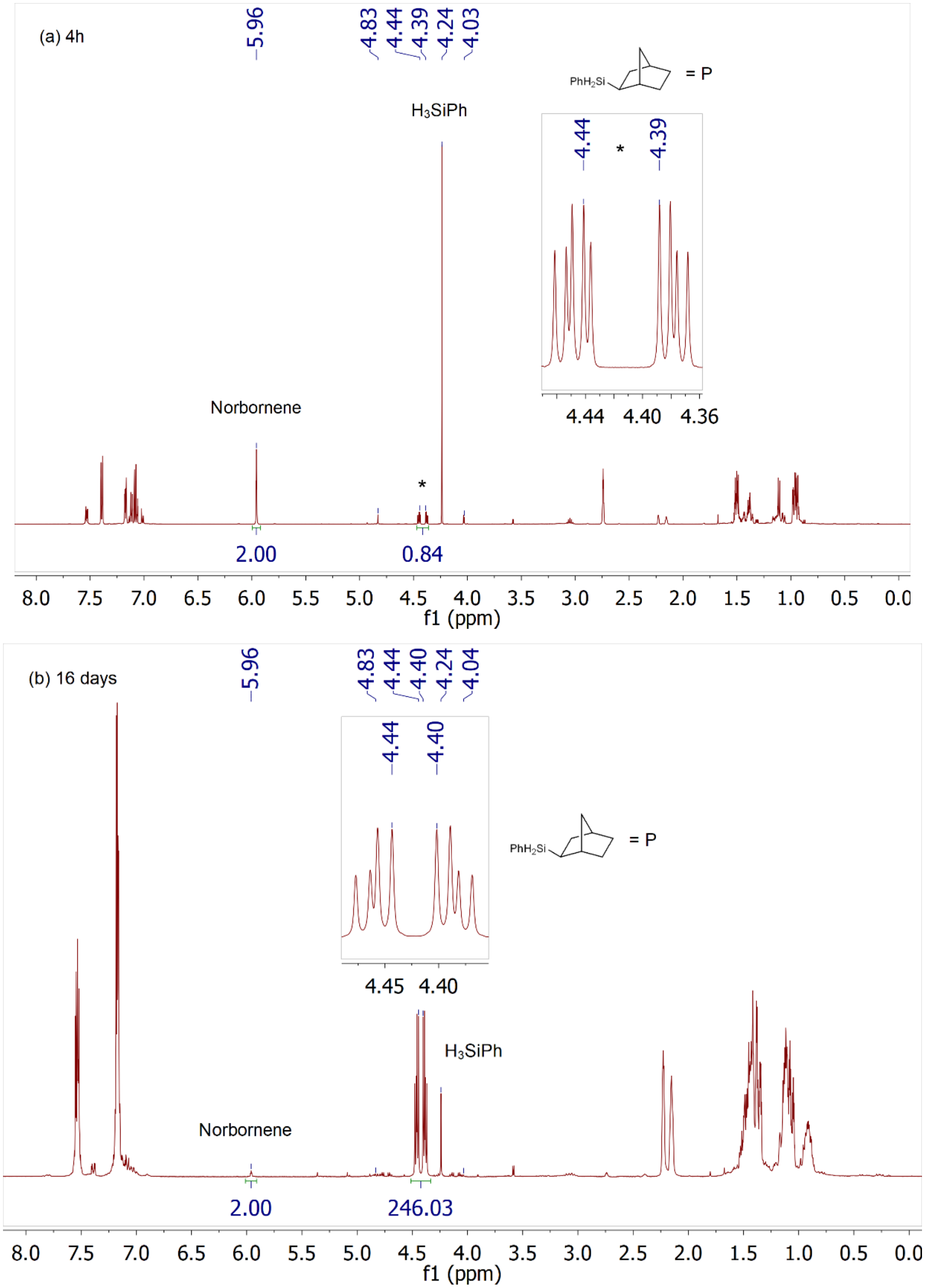

Figure S34. ^{1}H NMR spectra (300 MHz) of catalytic hydrosilylation of norbornene (0.106 mmol) mediated by $[(BDI)MgH]_2$ (5%) in C_6D_6 (0.4 mL) after (a) 4 hours and (b) 16 days at 80ºC. Assignment of major species: $[(BDI)MgH]_2$: (BDI methine = 4.83, Mg*H* = 4.03 ppm) in 1:1 ratio. H_3SiPh: (H_3Si = 4.24 ppm). Norbornene: (C*H*=CH = 5.96 ppm). P: (H_2Si = 4.42 ppm).

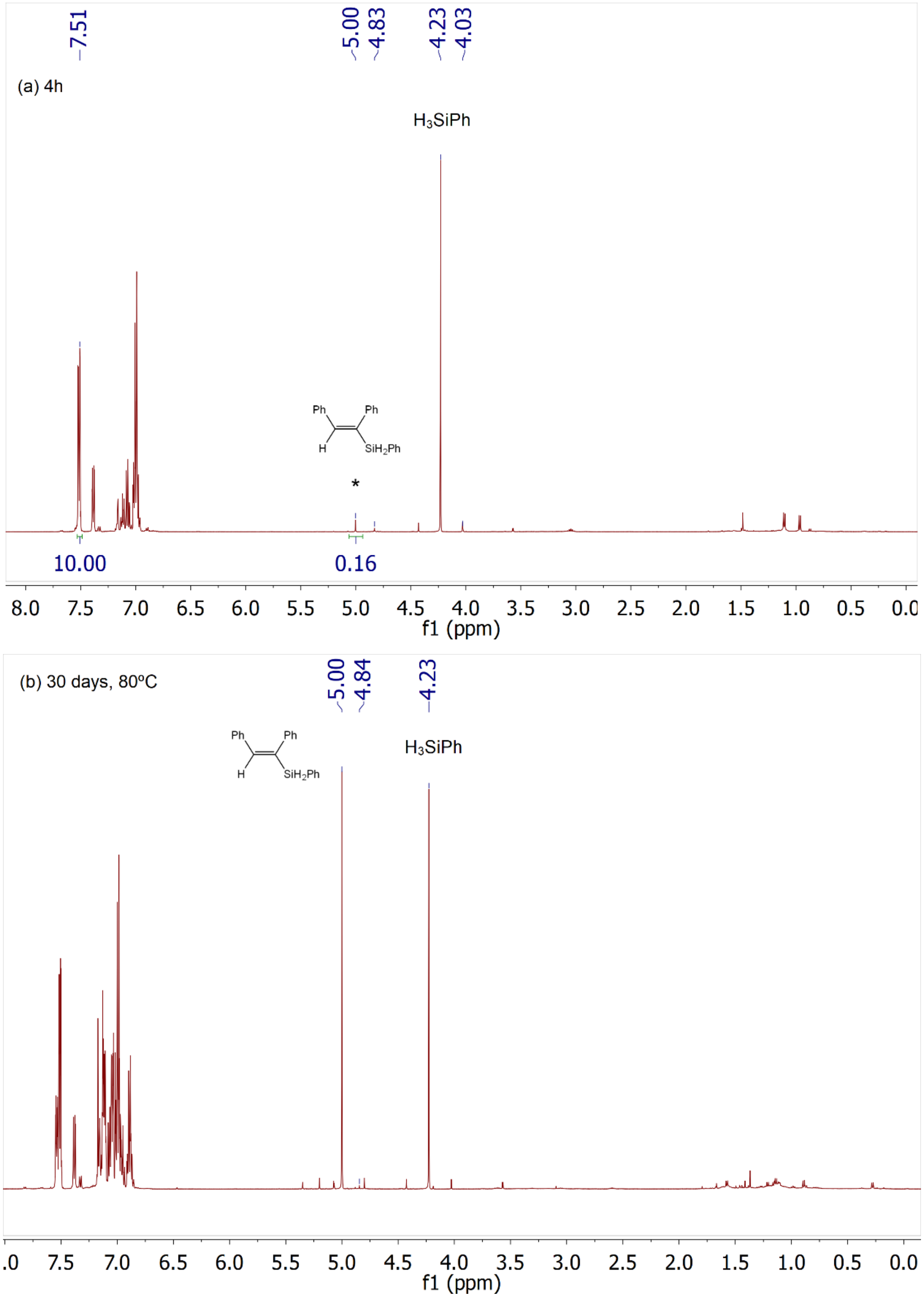

Figure S35. Stacked ^{1}H NMR spectra (300 MHz) of catalytic hydrosilylation of diphenylacetylene (0.107 mmol) mediated by $[(BDI)MgH]_2$ (5%) in C_6D_6 (0.4 mL) after (a) 4 hours and (b) 30 days at 80ºC. Assignment of major species: $[(BDI)MgH]_2$: (BDI methine = 4.83, MgH = 4.03 ppm) in 1:1 ratio. H_3SiPh: (H_3Si = 4.23 ppm). Diphenylacetylene: (CH Ph = 7.51 ppm). PhHC=CPh(SiH_2Ph): (H_2Si = 5.00 ppm).

Single Crystal X-Ray Diffraction Analysis

Single Crystals X-ray diffraction data for compounds **12** and **15** were collected on a SuperNova, Dual Cu at zero, EosS2 diffractometer. The crystals were kept at 150.00(10) K during data collection. Using Olex2,[2] the structures were solved via the olex2.solve[3] structure solution program using Charge Flipping and refined with the ShelXL[4] refinement package using Least Squares minimisation.

The asymmetric unit in **12** comprises 2 molecules. Disorder over 2 sites was modelled for C16 (60:40 ratio), C30-31 (90:10 ratio) and C65-67 (75:25). Chemically comparative bonds involving fractional occupancy carbon atoms were restrained to being similar in distance during the final least squares, and ADP restraints were also included in disordered regions, to assist convergence.

The asymmetric unit in **15** contains half of one molecule, with Mg1, C2, H2, C16 and H16 located co-incident with a crystallographic mirror plane. As such the norbornane ligand is disordered with itself, and the atoms therein are present in the asymmetric unit at half site-occupancy. Unsurprisingly, perhaps, C14 and C15 in the isopropyl group most proximate to the norbornane is also disordered over 2 adjacent sites, and was thus modelled, in a 50:50 ratio. H16 was located and refined subject to being bonded to C16 at a distance of 0.98 Å.

Table S1. Single Crystal X-ray Data Parameters for compounds **12** and **15**.

Compound	**12**	**15**
Empirical formula	$C_{35}H_{52}MgN_2$	$C_{36}H_{52}MgN_2$
Formula weight	525.09	537.10
Temperature/K	150.00(10)	150.15(10)
Crystal system	monoclinic	orthorhombic
Space group	$P2_1/c$	*P*nma
a/Å	19.0530(3)	18.7502(3)
b/Å	20.7671(2)	20.6951(4)
c/Å	17.0951(2)	8.5719(2)
α/°	90	90
β/°	103.4030(10)	90
γ/°	90	90
Volume/Å^3	6579.88(15)	3326.22(11)
Z	8	4
ρ_{calc} g/cm^3	1.060	1.073
μ/mm^{-1}	0.625	0.629
F(000)	2304.0	1176.0
Crystal size/mm^3	0.203 × 0.136 × 0.061	0.144 × 0.075 × 0.068
Radiation	CuKα (λ = 1.54184)	CuKα (λ = 1.54184)
2Θ range for data collection/°	6.392 to 146.952	8.546 to 146.72
Index ranges	$-18 \le h \le 23$, $-21 \le k \le 25$, $-21 \le l \le 19$	$-21 \le h \le 23$, $-25 \le k \le 25$, $-7 \le l \le 10$
Reflections collected	51062	28832
Independent reflections	13116 [R_{int} = 0.0391, R_{sigma} = 0.0405]	3443 [R_{int} = 0.0511, R_{sigma} = 0.0255]
Data/restraints/parameters	13116/23/749	3443/3/236
Goodness-of-fit on F^2	1.043	1.053
Final R indexes [I>=2σ (I)]	R_1 = 0.0511, wR_2 = 0.1390	R_1 = 0.0466, wR_2 = 0.1164
Final R indexes [all data]	R_1 = 0.0760, wR_2 = 0.1563	R_1 = 0.0598, wR_2 = 0.1232
Largest diff. peak/hole / e Å^{-3}	0.42/-0.41	0.31/-0.21

Computational studies

Calculations were carried out using the Gaussian09 package[5] at the DFT level by means of the hybrid density functional B3PW91.[6] A triple-zeta 6-311G basis set augmented by a polarisation and diffuse function was used for the Mg atom. Polarized all electron triple-zeta 6-311G(d,p) basis set were used for N, whereas a polarised all electron double-zeta 6-31G(d) basis set were used for the C and H atoms. The nature of the optimised stationary point, minima or transition state, has been verified by means of analytical frequency calculation at 298.15 K and 1 atm. The geometry optimisations have been achieved without any geometrical constraints. IRC calculations were carried out in order to confirm the connectivity between reactant(s), transition state and product(s). Energy data are reported in the gas phase.

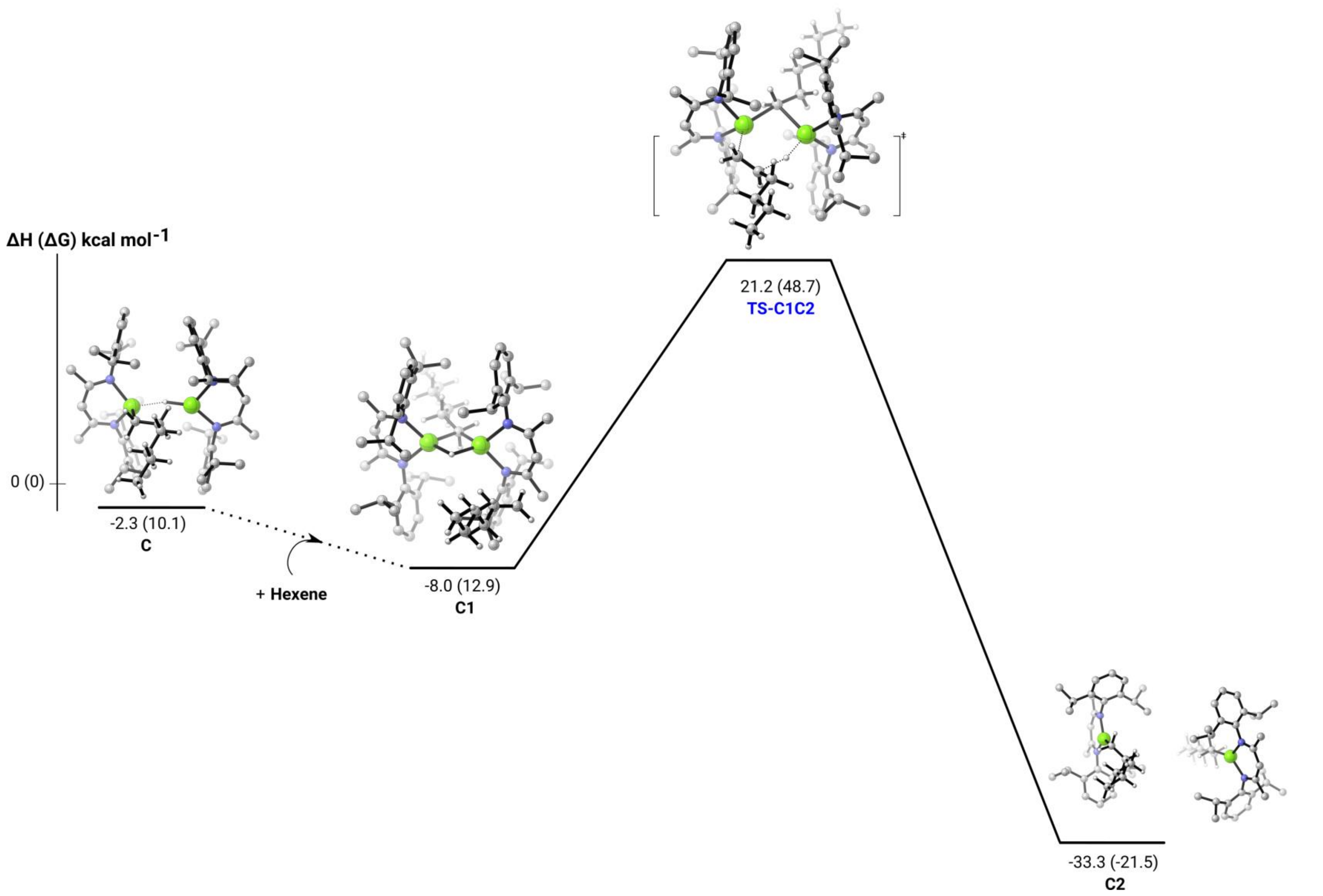

Figure S36. DFT (B3PW91) computed enthalpy reaction profile at room temperature for the second Mg-H/C=C 1-hexene insertion into the dimagnesium hydrido-*n*-hexyl species (**C**).

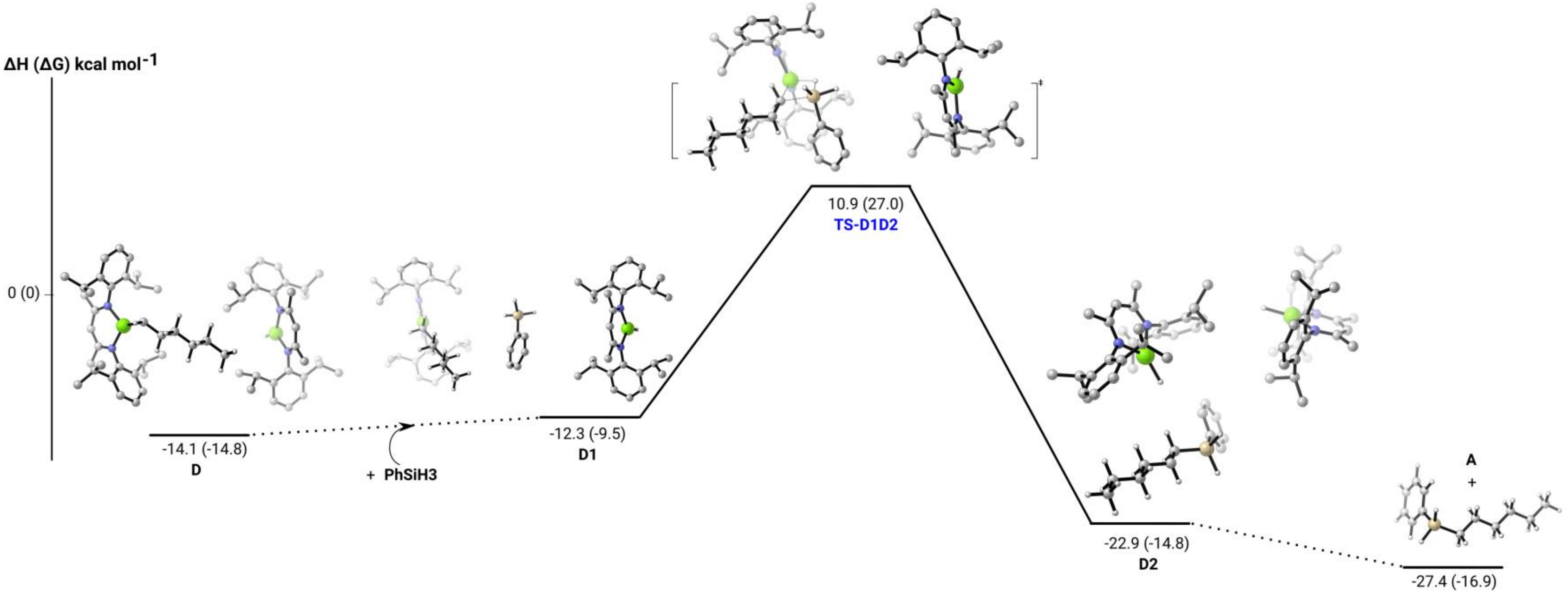

Figure S37. DFT (B3PW91) computed enthalpy reaction profile at room temperature for the Mg-C/Si-H metathesis process starting from complex **D**.

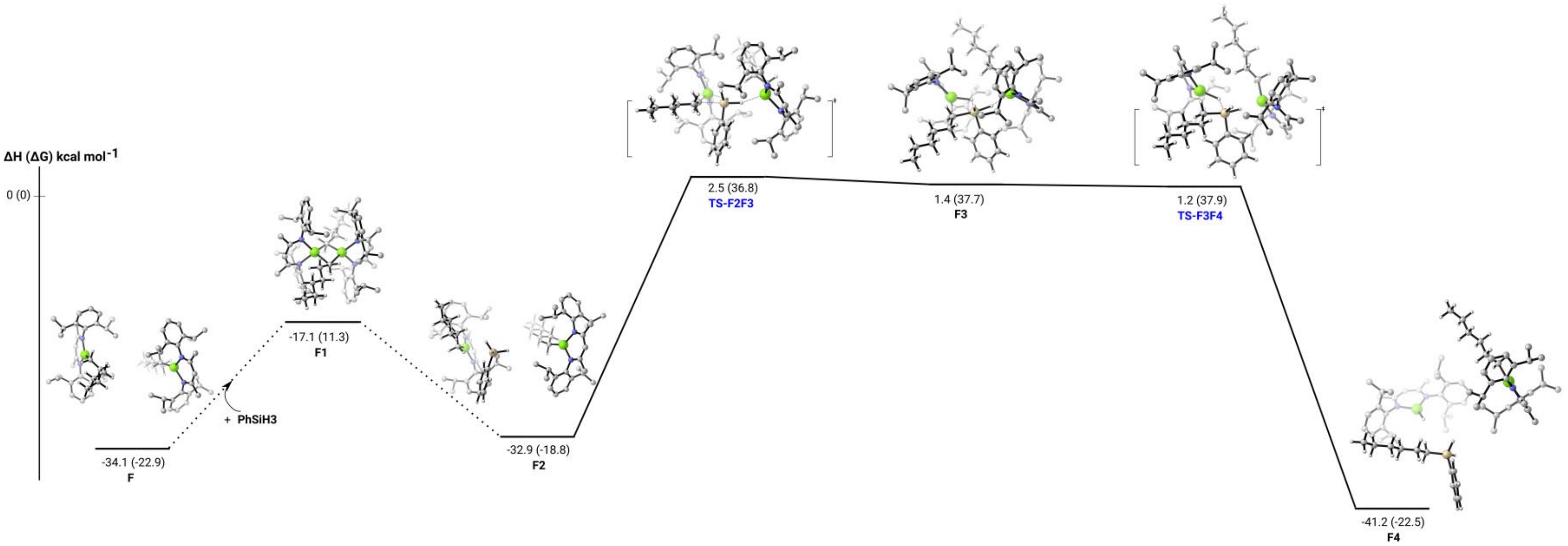

Figure S38. DFT (B3PW91) computed enthalpy reaction profile at room temperature for the Mg-C/Si-H metathesis process starting from the dimagnesium di-*n*-hexyl complex **F1**.

Cartesian Coordinates and Computed Energies (in Hartrees)

148
[A] scf done: -2879.147081 / Energies= -2877.777664 / Enthalpies= -2877.776719 / Free Energies= -2877.960296

C	22.618155	22.181070	2.678863
C	23.571517	21.219743	3.090397
C	24.873550	21.618010	3.475026
C	25.175634	22.982743	3.498806
C	24.241056	23.938304	3.115379
C	22.979079	23.532158	2.698461
N	23.215416	19.830056	3.085188
C	23.506962	19.151893	1.972324
C	24.288588	19.823375	0.860020
C	25.952798	20.601749	3.823519
C	26.161578	20.500674	5.336369
C	21.238629	21.791338	2.163332
C	20.111974	22.347315	3.044666
C	23.104503	17.834118	1.700459
C	22.162413	17.024834	2.356443
C	21.743912	15.784246	1.591215
N	21.611240	17.294042	3.542598
C	20.542651	16.430662	3.954863
C	19.207268	16.787834	3.652018
C	18.176948	15.938200	4.067803
C	18.442860	14.768582	4.769773
C	19.760346	14.419513	5.045079
C	20.828081	15.220531	4.629872
C	18.860673	18.029147	2.839428
C	17.986603	19.014549	3.626769
C	22.257571	14.757143	4.875801
C	22.686071	14.999577	6.325080
C	18.180166	17.662138	1.510900
C	22.474278	13.283378	4.501096
C	27.289644	20.894535	3.125758
C	21.035206	22.228839	0.704147
Mg	22.306369	18.889866	4.752904
Mg	22.180509	19.505765	7.682422
N	22.875902	21.101419	8.892769
C	23.944643	21.964639	8.480560
C	25.279974	21.607123	8.783201
C	26.310430	22.456674	8.367576
C	26.044696	23.626527	7.665936
C	24.727259	23.975897	7.390766
C	23.659399	23.174969	7.805824
C	25.626385	20.365520	9.595427
C	26.500419	19.380279	8.807844
C	22.229980	23.638638	7.559995
C	21.801312	23.396150	6.110773
C	22.324666	21.370773	10.078862
C	21.382231	20.561777	10.734710
C	20.979431	19.244116	10.462810
C	20.197375	18.572893	11.574963
C	22.743421	22.611238	10.844158
N	21.271046	18.565809	9.350049
C	20.914734	17.176175	9.344878
C	19.612756	16.778043	8.959932
C	19.310541	15.413340	8.936053
C	20.244922	14.457680	9.319712

C	21.506807	14.863701	9.737028
C	21.867862	16.214752	9.756737
C	18.533679	17.794410	8.611212
C	18.325119	17.895406	7.098327
C	23.247255	16.604331	10.272756
C	24.374161	16.048314	9.391767
C	26.306813	20.732064	10.924120
C	22.013587	25.112475	7.934587
C	17.196700	17.501801	9.308800
C	23.450144	16.166700	11.731976
H	21.184267	19.831143	6.008927
H	22.502136	23.523271	10.289639
H	22.245719	22.656770	11.815059
H	23.825287	22.625114	11.007860
H	21.018426	20.961558	11.673552
H	20.722424	17.684057	11.939013
H	20.050011	19.254776	12.415101
H	19.217378	18.232614	11.228541
H	24.687953	19.855268	9.836651
H	25.691529	21.411231	11.524015
H	26.489818	19.829738	11.520328
H	27.274220	21.220758	10.757028
H	27.449173	19.839643	8.504999
H	26.738723	18.505141	9.424310
H	25.992761	19.030225	7.904060
H	27.340470	22.196170	8.601386
H	26.860020	24.271378	7.346499
H	24.522296	24.900396	6.857411
H	21.571466	23.038066	8.196519
H	20.946683	25.359846	7.880760
H	22.363431	25.336541	8.948871
H	22.537092	25.788137	7.247975
H	21.872919	22.336950	5.844976
H	20.761314	23.711550	5.960586
H	22.432500	23.955521	5.412949
H	18.876883	18.775829	8.955704
H	16.725341	16.592155	8.918550
H	17.312759	17.374993	10.391391
H	16.496587	18.328194	9.137791
H	17.563936	18.650337	6.865493
H	19.249442	18.181527	6.587053
H	17.997587	16.938873	6.678908
H	18.318558	15.094272	8.627630
H	19.984923	13.401820	9.307350
H	22.229111	14.116531	10.058701
H	23.315043	17.696981	10.245323
H	23.433389	15.074499	11.829668
H	24.420844	16.517125	12.103511
H	22.673288	16.570019	12.390481
H	24.308583	16.432737	8.369458
H	25.352031	16.331999	9.799162
H	24.340430	14.953191	9.342336
H	23.302585	18.564536	6.426283
H	21.984966	14.872308	2.145983
H	22.241950	15.738539	0.620494
H	20.662104	15.770379	1.427124
H	23.468274	17.434464	0.761549
H	23.763394	20.712278	0.496346
H	24.435661	19.141667	0.019689
H	25.268713	20.163598	1.206145

H	19.799034	18.539421	2.597978
H	18.795402	16.982755	0.911198
H	17.997135	18.564259	0.914389
H	17.212762	17.173514	1.678209
H	17.037894	18.555195	3.929774
H	17.748214	19.889496	3.010063
H	18.494269	19.364886	4.530439
H	17.146875	16.198459	3.833866
H	17.627636	14.123667	5.089338
H	19.965458	13.495174	5.578659
H	22.916009	15.357910	4.239379
H	23.541217	13.036181	4.555048
H	22.124614	13.059347	3.486744
H	21.950783	12.607568	5.187570
H	22.614163	16.058728	6.590987
H	23.726144	14.684437	6.475303
H	22.054982	14.439959	7.022795
H	25.609663	19.620312	3.479014
H	27.760967	21.804216	3.515975
H	27.173415	21.021368	2.043188
H	27.989871	20.068211	3.296632
H	26.922845	19.745779	5.569039
H	25.237350	20.214461	5.847768
H	26.489132	21.457197	5.755795
H	26.167667	23.301916	3.806963
H	24.500958	24.994190	3.127643
H	22.256602	24.279254	2.377013
H	21.170950	20.698684	2.190870
H	21.051840	23.321036	0.606369
H	20.064410	21.878312	0.332961
H	21.811873	21.825533	0.045412
H	20.178022	21.963069	4.067011
H	19.133994	22.063389	2.637704
H	20.145532	23.442453	3.093893

166
[B] scf done: -3114.906809 / Energies= -3113.359810 / Enthalpies= -3113.358866 / Free Energies= -3113.571399

C	2.823426	1.535891	-3.209186
C	1.462976	1.911801	-3.302696
C	1.088994	3.276220	-3.259961
C	2.083493	4.234299	-3.043421
C	3.420615	3.874448	-2.915613
C	3.781535	2.536097	-3.013940
N	0.461479	0.898748	-3.468525
C	0.121033	0.603699	-4.725089
C	0.716860	1.393674	-5.873728
C	-0.349473	3.719984	-3.491638
C	-0.454797	4.887695	-4.483994
C	3.281172	0.091642	-3.372221
C	4.258796	-0.064389	-4.547981
C	-0.736065	-0.441426	-5.106516
C	-1.240292	-1.514069	-4.353592
C	-1.888985	-2.617819	-5.165392
N	-1.149325	-1.631040	-3.026612
C	-1.570295	-2.883445	-2.468606
C	-2.928067	-3.087913	-2.127455
C	-3.292467	-4.300186	-1.534413
C	-2.361168	-5.309941	-1.319701
C	-1.040893	-5.117770	-1.709260
C	-0.619647	-3.915782	-2.287156

C	-3.997202	-2.047485	-2.433498
C	-5.227354	-2.654330	-3.125289
C	0.825166	-3.783759	-2.753795
C	1.177371	-4.843603	-3.810017
C	-4.425505	-1.284493	-1.177570
C	1.822860	-3.850726	-1.589541
C	-1.052596	4.077600	-2.179971
C	3.906681	-0.469475	-2.088198
Mg	-0.440784	-0.052754	-1.803467
Mg	-0.701512	0.619289	1.109566
N	-1.581427	-0.345288	2.779816
C	-1.224934	-0.052905	4.032380
C	-0.384412	1.008843	4.406564
C	0.077685	2.101461	3.652428
N	-0.022908	2.215442	2.326802
C	0.367598	3.476383	1.764728
C	1.724876	3.720237	1.446836
C	2.062952	4.941488	0.856909
C	1.105174	5.921344	0.620038
C	-0.216149	5.688900	0.982465
C	-0.611455	4.476276	1.556971
C	2.818350	2.710717	1.769138
C	4.037358	3.352863	2.447989
C	-2.060752	4.302634	1.994824
C	-2.449680	5.328174	3.072113
C	-2.583511	-1.359626	2.623357
C	-2.210065	-2.722992	2.559118
C	-3.207460	-3.679563	2.348761
C	-4.546623	-3.319748	2.246698
C	-4.906647	-1.982883	2.367386
C	-3.946246	-0.984261	2.559287
C	-0.768206	-3.169829	2.759438
C	-0.644498	-4.335716	3.752023
C	-4.404205	0.456129	2.753994
C	-5.351318	0.590535	3.957309
C	-1.781605	-0.865367	5.184848
C	-5.065682	1.034023	1.495892
C	-0.097670	-3.534021	1.432813
C	0.690818	3.225839	4.463744
C	3.259088	1.935690	0.525088
C	-3.041068	4.380465	0.816698
C	3.623201	-0.656702	4.483047
C	4.192900	-1.202610	3.406966
C	5.604977	-1.704619	3.331328
C	5.690349	-3.200326	2.995192
C	7.126619	-3.709813	2.868354
C	7.207555	-5.197226	2.531724
H	0.489161	-0.346924	-0.131261
H	-1.628536	0.912048	-0.561858
H	1.731052	3.405633	4.176272
H	0.155304	4.165251	4.294993
H	0.662202	2.998061	5.531386
H	-0.183667	1.074855	5.469153
H	-2.873978	-0.798421	5.214217
H	-1.538870	-1.927091	5.083750
H	-1.388770	-0.509320	6.139559
H	2.404012	1.979996	2.471255
H	3.754146	3.969250	3.309128
H	4.719050	2.570184	2.800136
H	4.600179	3.991053	1.756384

H	3.683396	2.605642	-0.229647
H	4.018974	1.190613	0.790383
H	2.417987	1.409467	0.063273
H	3.099432	5.135364	0.593995
H	1.391568	6.869417	0.170888
H	-0.958889	6.467389	0.822228
H	-2.157185	3.306353	2.439507
H	-3.470967	5.140743	3.425752
H	-1.781387	5.284669	3.938866
H	-2.417766	6.351861	2.679941
H	-2.849276	3.591312	0.083860
H	-4.072646	4.270215	1.172069
H	-2.970047	5.344849	0.299366
H	-3.516465	1.061216	2.966656
H	-6.290598	0.048780	3.793173
H	-4.901435	0.200691	4.876894
H	-5.603272	1.644526	4.126781
H	-5.397075	2.063448	1.678276
H	-4.373608	1.046277	0.648628
H	-5.945594	0.448817	1.202628
H	-5.957614	-1.706174	2.318522
H	-5.307937	-4.080930	2.093295
H	-2.931397	-4.728502	2.280111
H	-0.213380	-2.324010	3.178903
H	-1.070439	-5.261783	3.348396
H	0.412502	-4.531660	3.967841
H	-1.150683	-4.125982	4.701424
H	-0.100224	-2.686786	0.740186
H	0.945433	-3.830404	1.599293
H	-0.615442	-4.363913	0.940995
H	-2.930219	-2.773687	-4.867485
H	-1.376017	-3.571909	-5.008771
H	-1.866005	-2.383868	-6.231842
H	-0.930317	-0.503170	-6.170504
H	0.325511	1.045068	-6.831811
H	1.806880	1.290331	-5.888441
H	0.508910	2.463444	-5.781372
H	-3.560639	-1.316057	-3.122202
H	-4.957018	-3.246132	-4.007372
H	-5.907231	-1.856403	-3.447173
H	-5.792008	-3.308164	-2.450208
H	-4.861315	-1.959028	-0.433415
H	-5.173040	-0.521773	-1.428921
H	-3.575631	-0.779889	-0.708330
H	-4.328950	-4.462670	-1.250879
H	-2.667716	-6.249916	-0.866936
H	-0.319724	-5.919676	-1.566876
H	0.934065	-2.801120	-3.224880
H	2.194301	-4.681172	-4.187673
H	0.493138	-4.812832	-4.664845
H	1.138461	-5.856308	-3.391059
H	1.658249	-3.039693	-0.874059
H	2.850696	-3.770479	-1.963877
H	1.740043	-4.799931	-1.046419
H	2.396701	-0.513259	-3.598643
H	5.195985	0.475663	-4.367253
H	3.835023	0.313928	-5.484666
H	4.510404	-1.121790	-4.695360
H	4.233787	-1.504623	-2.244493
H	3.193143	-0.460872	-1.258710

H	4.783579	0.114665	-1.783972
H	4.831162	2.259292	-2.942610
H	4.179894	4.636869	-2.758435
H	1.807913	5.284275	-2.991455
H	-0.891668	2.873687	-3.926504
H	-0.042376	5.814748	-4.068882
H	-1.507477	5.080542	-4.722995
H	0.073347	4.681692	-5.422213
H	-1.061963	3.229129	-1.488884
H	-2.093378	4.368492	-2.369878
H	-0.551150	4.909369	-1.674719
H	2.589806	-0.318071	4.475852
H	4.175384	-0.525513	5.412882
H	3.600001	-1.315803	2.496840
H	6.150470	-1.141890	2.557250
H	6.120032	-1.507382	4.281389
H	5.162435	-3.772494	3.771007
H	5.151973	-3.392189	2.055030
H	7.663910	-3.516627	3.807960
H	7.649659	-3.129033	2.094948
H	6.720132	-5.805469	3.303592
H	8.247535	-5.533798	2.450553
H	6.711790	-5.415676	1.577782

166

[TS-BC] scf done: -3114.874775 / Energies= -3113.330379 / Enthalpies= -3113.329435 / Free Energies= -3113.533857

C	25.434600	20.825176	2.745417
C	24.028993	20.867815	2.914029
C	23.383337	22.086410	3.229026
C	24.164329	23.230049	3.423456
C	25.546656	23.193999	3.284567
C	26.168488	21.999384	2.938977
N	23.266237	19.662438	2.783795
C	22.832510	19.336398	1.565284
C	23.070836	20.287213	0.409827
C	21.868230	22.192778	3.324618
C	21.303051	23.269976	2.385703
C	26.164121	19.550259	2.341989
C	27.030360	19.745912	1.088705
C	22.178602	18.138152	1.231997
C	21.889361	17.002005	2.011040
C	21.253024	15.854034	1.247251
N	22.127612	16.892073	3.317143
C	21.779340	15.661617	3.955279
C	20.430850	15.396501	4.293410
C	20.124385	14.184987	4.920845
C	21.111968	13.251618	5.210811
C	22.435342	13.526240	4.882949
C	22.798166	14.722671	4.255895
C	19.319781	16.397324	4.005641
C	18.048519	15.742375	3.448258
C	24.249450	14.976556	3.863992
C	24.515827	14.623569	2.391328
C	18.989115	17.222668	5.254925
C	25.261682	14.240383	4.749173
C	21.406555	22.454160	4.762865
C	27.012463	18.997925	3.496012
Mg	22.966617	18.449793	4.480679
Mg	22.240263	19.397044	7.883684
N	20.761279	18.814530	9.305797

C	21.047770	19.120647	10.568717
C	22.104767	19.969091	10.974309
C	22.805719	20.962942	10.266889
N	22.746033	21.149303	8.943363
C	23.175060	22.420866	8.435467
C	24.485488	22.613406	7.942829
C	24.839869	23.866033	7.429334
C	23.945742	24.927316	7.419869
C	22.666089	24.738619	7.927941
C	22.254707	23.501538	8.432424
C	25.542075	21.520988	7.966955
C	26.792921	21.945037	8.753468
C	20.845108	23.377548	8.995583
C	20.705312	24.109747	10.340307
C	19.580765	18.036359	9.058504
C	19.593131	16.626880	9.210783
C	18.417393	15.911976	8.961904
C	17.242376	16.550154	8.583131
C	17.240650	17.930132	8.431266
C	18.392443	18.691052	8.656361
C	20.839684	15.855341	9.620092
C	20.585080	14.849114	10.752998
C	18.310114	20.201120	8.496015
C	17.381585	20.824562	9.549401
C	20.194763	18.575362	11.697690
C	17.870028	20.600971	7.080753
C	21.453406	15.131706	8.413580
C	23.674646	21.871219	11.115734
C	25.932011	21.099945	6.546138
C	19.779681	23.884849	8.014643
C	23.934062	17.856220	8.382288
C	24.575118	17.226369	7.331196
C	26.070487	17.294338	7.159411
C	26.780057	16.330327	8.127006
C	28.301822	16.353823	7.971378
C	29.014045	15.394845	8.923116
H	24.278381	17.939711	5.645722
H	22.004177	19.229271	6.042284
H	24.731692	21.670166	10.904684
H	23.506487	22.927076	10.889552
H	23.502609	21.707425	12.181858
H	22.282463	19.974572	12.043661
H	19.140567	18.823730	11.535704
H	20.251056	17.484416	11.756566
H	20.504498	18.990147	12.659132
H	25.111040	20.650551	8.473929
H	26.546352	22.291219	9.763097
H	27.489570	21.102522	8.842621
H	27.326727	22.758884	8.248666
H	26.304874	21.951770	5.968058
H	26.722535	20.341734	6.567607
H	25.080873	20.683045	5.998721
H	25.844162	24.012076	7.039203
H	24.245077	25.896423	7.027927
H	21.969163	25.573311	7.937299
H	20.653457	22.315354	9.175837
H	19.686092	24.001765	10.731413
H	21.394611	23.716468	11.093990
H	20.908999	25.181876	10.227315
H	19.851364	23.377193	7.048300

H	18.776649	23.710116	8.421610
H	19.870621	24.962589	7.834076
H	19.313993	20.605421	8.659938
H	16.353033	20.459670	9.440105
H	17.711658	20.588906	10.567129
H	17.359266	21.916108	9.447308
H	17.850662	21.691926	6.979297
H	18.553471	20.200445	6.324958
H	16.863597	20.230425	6.851886
H	16.323255	18.433773	8.135272
H	16.336806	15.975204	8.405513
H	18.422429	14.830744	9.074673
H	21.574089	16.578575	9.990169
H	19.953608	14.016652	10.421684
H	21.534953	14.419592	11.093022
H	20.093881	15.311458	11.616433
H	21.647003	15.815036	7.580503
H	22.398516	14.648022	8.689391
H	20.776142	14.357317	8.037484
H	20.165598	15.854336	1.385514
H	21.617136	14.882529	1.590334
H	21.451160	15.947172	0.176546
H	21.894901	18.052917	0.189263
H	22.592160	19.926327	-0.503209
H	24.142677	20.401406	0.214842
H	22.688440	21.287340	0.634357
H	19.684371	17.099628	3.249531
H	18.262214	15.097163	2.588263
H	17.340051	16.515103	3.126877
H	17.537798	15.132220	4.202425
H	18.642285	16.584668	6.074470
H	18.197486	17.949356	5.035473
H	19.864886	17.775994	5.610241
H	19.092810	13.967868	5.185427
H	20.852973	12.311458	5.692046
H	23.199931	12.790616	5.115019
H	24.434441	16.052388	3.983636
H	25.573870	14.782130	2.147516
H	23.924183	15.239533	1.709292
H	24.279497	13.569835	2.196694
H	25.066963	14.396128	5.815642
H	26.273488	14.601843	4.533025
H	25.261801	13.159409	4.561363
H	25.407882	18.794962	2.103860
H	27.864193	20.433890	1.273810
H	26.451294	20.147158	0.249404
H	27.460823	18.787615	0.773456
H	27.516952	18.071582	3.192552
H	26.389676	18.778373	4.369237
H	27.784188	19.716371	3.799491
H	27.248761	21.976870	2.813920
H	26.135957	24.095670	3.433758
H	23.677659	24.167474	3.681320
H	21.449046	21.229781	3.012938
H	21.627867	24.274453	2.681928
H	20.206685	23.257912	2.412691
H	21.615829	23.115346	1.347000
H	21.745041	21.661761	5.437288
H	20.310808	22.493136	4.807681
H	21.796329	23.405086	5.143448

H	23.089506	17.351528	8.850610
H	24.503281	18.526547	9.027594
H	24.101924	16.335961	6.922743
H	26.348665	17.050264	6.129241
H	26.417209	18.315793	7.353726
H	26.509820	16.586021	9.160357
H	26.414594	15.306452	7.959429
H	28.666040	17.377377	8.140201
H	28.565157	16.104955	6.933555
H	28.793121	15.637958	9.969769
H	30.101744	15.436475	8.794180
H	28.697916	14.358521	8.751198

166

[C] scf done: -3114.914673 / Energies= -3113.364154 / Enthalpies= -3113.363210 / Free Energies= -3113.567879

C	3.217228	1.692554	-3.053359
C	1.810034	1.782691	-3.185416
C	1.155054	3.034136	-3.131447
C	1.926412	4.182899	-2.930142
C	3.308555	4.111982	-2.816205
C	3.942510	2.875994	-2.882495
N	1.045355	0.584139	-3.376598
C	0.721295	0.253961	-4.629536
C	1.099134	1.164959	-5.781709
C	-0.353520	3.164740	-3.290514
C	-0.749437	4.251365	-4.301295
C	3.944923	0.354707	-3.130995
C	4.265713	-0.052214	-4.579105
C	-0.009321	-0.888425	-5.000725
C	-0.554542	-1.928636	-4.229133
C	-1.313425	-2.976831	-5.020211
N	-0.478979	-2.019298	-2.897566
C	-1.062435	-3.176809	-2.282836
C	-2.445735	-3.203870	-1.993717
C	-2.977721	-4.346002	-1.386851
C	-2.181788	-5.447067	-1.098907
C	-0.823614	-5.411451	-1.395422
C	-0.234151	-4.283871	-1.975630
C	-3.364147	-2.035932	-2.324481
C	-4.648561	-2.476392	-3.042335
C	1.257800	-4.277531	-2.289489
C	1.557973	-4.740419	-3.724969
C	-3.710431	-1.229198	-1.066829
C	2.082129	-5.112998	-1.303985
C	-1.035242	3.424333	-1.941045
C	5.231974	0.318094	-2.299055
Mg	0.244681	-0.442395	-1.757231
Mg	-0.017928	0.399064	1.835234
N	-1.519535	-0.224018	3.224117
C	-1.276123	0.169167	4.472931
C	-0.424734	1.234861	4.831604
C	0.092757	2.293923	4.056769
N	0.072247	2.344799	2.725190
C	0.412192	3.580109	2.090075
C	1.754615	3.899824	1.778491
C	2.029178	5.112692	1.137291
C	1.019426	6.011672	0.822402
C	-0.297238	5.695140	1.139828
C	-0.628203	4.487715	1.761904
C	2.914131	2.977715	2.121685

C	3.996859	3.680148	2.956171
C	-2.079887	4.190214	2.113965
C	-2.443360	4.724652	3.509526
C	-2.552891	-1.190928	3.016281
C	-2.245135	-2.572972	2.976442
C	-3.270614	-3.488313	2.719554
C	-4.582035	-3.073607	2.523175
C	-4.880835	-1.717888	2.588661
C	-3.890390	-0.761767	2.835403
C	-0.840095	-3.099098	3.227312
C	-0.783585	-4.076863	4.411365
C	-4.292563	0.703808	2.914876
C	-5.360818	0.950859	3.991233
C	-1.978196	-0.507618	5.633538
C	-4.781536	1.223568	1.556143
C	-0.264238	-3.762196	1.971318
C	0.719107	3.420063	4.859862
C	3.538313	2.382481	0.853984
C	-3.079276	4.732911	1.085653
C	1.825965	-0.720529	1.724611
C	2.259301	-1.287895	0.375931
C	3.691285	-1.851914	0.285166
C	4.015199	-2.942560	1.305622
C	5.434884	-3.494485	1.164860
C	5.767705	-4.570042	2.197236
H	2.207135	-0.489536	-0.404915
H	-0.427255	0.236473	-0.215509
H	1.807547	3.288507	4.887016
H	0.525817	4.399940	4.417564
H	0.357031	3.419285	5.890899
H	-0.274242	1.348068	5.899494
H	-3.020459	-0.173089	5.693002
H	-2.003020	-1.593919	5.518264
H	-1.492807	-0.260893	6.580939
H	2.518112	2.147811	2.716033
H	3.581841	4.159484	3.849255
H	4.754663	2.955981	3.279276
H	4.512389	4.456112	2.377329
H	3.963652	3.165937	0.216765
H	4.340850	1.681356	1.114306
H	2.794546	1.843675	0.258964
H	3.059011	5.358842	0.889699
H	1.254947	6.956004	0.337127
H	-1.084409	6.403813	0.897200
H	-2.184885	3.099145	2.143345
H	-3.498488	4.523859	3.733961
H	-1.842814	4.258275	4.295180
H	-2.288789	5.809865	3.561194
H	-2.834818	4.408523	0.069592
H	-4.088112	4.377076	1.322399
H	-3.121231	5.828908	1.091161
H	-3.402730	1.278832	3.189592
H	-6.308984	0.460252	3.739592
H	-5.046082	0.575660	4.971067
H	-5.561993	2.024487	4.089910
H	-5.084745	2.274551	1.629975
H	-3.997326	1.148473	0.796235
H	-5.647909	0.652032	1.201547
H	-5.909358	-1.391137	2.450694
H	-5.367234	-3.801668	2.333142

H	-3.035148	-4.549440	2.683700
H	-0.202705	-2.243685	3.472527
H	-1.366912	-4.984274	4.213758
H	0.252648	-4.384611	4.597498
H	-1.170369	-3.629510	5.333442
H	-0.279074	-3.076123	1.119118
H	0.773885	-4.071511	2.142472
H	-0.840342	-4.649801	1.686247
H	-2.388590	-2.763710	-4.982648
H	-1.175095	-3.983316	-4.620157
H	-1.009003	-2.966314	-6.069604
H	-0.183368	-0.982048	-6.066720
H	1.136519	0.606419	-6.720340
H	2.057490	1.663328	-5.625078
H	0.342714	1.951416	-5.893891
H	-2.825242	-1.367208	-3.004946
H	-4.437085	-3.098766	-3.919348
H	-5.212188	-1.597388	-3.377036
H	-5.307718	-3.050414	-2.380910
H	-4.238663	-1.846526	-0.332285
H	-4.354096	-0.378442	-1.322361
H	-2.811519	-0.839646	-0.577126
H	-4.036415	-4.374991	-1.143512
H	-2.617114	-6.332097	-0.641298
H	-0.208857	-6.275892	-1.162715
H	1.601766	-3.237563	-2.211152
H	2.641677	-4.777951	-3.891185
H	1.133142	-4.066400	-4.473294
H	1.155827	-5.745959	-3.900416
H	1.831707	-4.878624	-0.264842
H	3.150107	-4.917077	-1.450990
H	1.930232	-6.188741	-1.454930
H	3.265924	-0.408101	-2.727236
H	4.877494	0.714493	-5.070595
H	3.363083	-0.203880	-5.176813
H	4.831313	-0.992192	-4.591555
H	5.608435	-0.709005	-2.240721
H	5.068610	0.678203	-1.278731
H	6.025661	0.925354	-2.751126
H	5.023674	2.831156	-2.791481
H	3.892746	5.017848	-2.674280
H	1.435241	5.150213	-2.869280
H	-0.735659	2.211609	-3.672357
H	-0.515053	5.255698	-3.930096
H	-1.829933	4.218291	-4.485370
H	-0.236267	4.125161	-5.261267
H	-0.827499	2.625513	-1.221160
H	-2.122902	3.490485	-2.071674
H	-0.685527	4.362428	-1.496589
H	1.762021	-1.552036	2.443332
H	2.638725	-0.079795	2.105392
H	1.555026	-2.081966	0.062442
H	3.877573	-2.238562	-0.729858
H	4.395570	-1.020483	0.430477
H	3.880948	-2.538196	2.317248
H	3.295228	-3.768202	1.209029
H	6.154086	-2.667238	1.253581
H	5.569843	-3.904616	0.153089
H	5.676776	-4.179333	3.218320
H	6.790630	-4.944782	2.074074

H	5.087044	-5.426213	2.110611

166

[D] scf done: -3114.932759 / Energies= -3113.382997 / Enthalpies= -3113.382053 / Free Energies= -3113.607625

C	13.404582	15.789081	6.625771
C	14.101180	15.128299	7.664152
C	15.118009	15.798170	8.385224
C	15.422934	17.118730	8.043179
C	14.747956	17.776372	7.021072
C	13.747240	17.110762	6.323519
N	13.843977	13.744214	7.921105
C	12.975101	13.392286	8.868221
C	12.676294	12.062284	9.221954
C	13.184481	10.849316	8.717207
C	12.663867	9.583317	9.361665
C	15.904077	15.110665	9.493330
C	15.923963	15.923057	10.795784
C	12.310134	15.100224	5.822516
C	10.979539	15.864540	5.877565
Mg	14.867206	12.390391	6.754822
N	14.076209	10.760173	7.732071
C	14.528988	9.476905	7.289657
C	15.706601	8.923020	7.843096
C	16.186891	7.715980	7.326304
C	15.532449	7.061579	6.289494
C	14.375664	7.615014	5.753124
C	13.853463	8.819020	6.235109
C	16.476289	9.618595	8.956717
C	16.708197	8.707190	10.169970
C	12.600803	9.399877	5.594394
C	11.434422	8.402125	5.567518
C	17.810615	10.176008	8.436844
C	12.898867	9.919504	4.179177
C	12.245620	14.470191	9.640021
C	17.336105	14.794221	9.035980
C	12.745252	14.877521	4.365855
C	16.158959	12.470960	5.073078
C	17.201930	13.592065	4.955065
C	18.023654	13.555056	3.659958
C	19.067829	14.667263	3.548529
C	19.876028	14.625267	2.250387
C	20.918089	15.737399	2.148096
C	22.811659	18.373790	6.495912
C	23.505592	17.517148	7.563854
C	22.504539	17.093938	8.650962
C	24.711647	18.207486	8.184150
C	25.968238	17.566089	8.284518
C	27.056222	18.211846	8.915957
C	26.866141	19.499714	9.426349
C	25.638710	20.143474	9.325049
C	24.573764	19.495057	8.710499
N	26.113713	16.215796	7.831542
Mg	25.704791	14.743807	9.194070
N	26.118836	13.219301	7.892370
C	26.494219	13.414556	6.628623
C	26.659206	14.681194	6.034756
C	26.485747	15.970308	6.575557
C	26.742676	17.132136	5.641686
C	28.406781	17.531204	9.085908
C	29.565087	18.372319	8.531578

C	25.970638	11.888797	8.399639
C	24.716169	11.241533	8.310955
C	24.572661	9.978117	8.891722
C	25.630199	9.358945	9.547728
C	26.855610	10.008367	9.636391
C	27.051036	11.273087	9.072775
C	23.517475	11.901181	7.644417
C	22.499145	12.364794	8.698522
C	28.398120	11.963217	9.230597
C	29.565976	11.097295	8.737970
C	26.765398	12.216425	5.746129
C	28.621421	12.396848	10.688212
C	22.843081	10.997072	6.603527
C	28.655051	17.170304	10.559202
H	25.206587	14.777964	10.842696
H	12.950057	15.084041	10.212349
H	11.722400	15.150805	8.959825
H	11.519407	14.040672	10.333416
H	11.948107	11.955356	10.017662
H	11.927181	9.804594	10.136934
H	12.202075	8.926557	8.616730
H	13.483729	9.012954	9.812512
H	15.411523	14.156504	9.710339
H	14.911530	16.168033	11.136182
H	16.419855	15.353674	11.590855
H	16.471089	16.865834	10.679433
H	17.889045	15.713860	8.809263
H	17.884265	14.255540	9.818692
H	17.342050	14.178953	8.129030
H	16.207337	17.641054	8.585846
H	15.001249	18.803253	6.769536
H	13.220504	17.626741	5.523944
H	12.137897	14.115282	6.271552
H	11.057895	16.848009	5.399147
H	10.196273	15.304610	5.352522
H	10.647053	16.023886	6.909394
H	13.671477	14.294777	4.307340
H	11.968015	14.341155	3.807525
H	12.924993	15.832063	3.856575
H	12.282694	10.255904	6.199941
H	11.206966	8.017394	6.568078
H	10.530544	8.885471	5.177569
H	11.649193	7.541687	4.922737
H	13.212388	9.102586	3.517960
H	12.006836	10.383726	3.740494
H	13.704601	10.662414	4.184160
H	13.868449	7.102955	4.938672
H	15.923388	6.125050	5.899579
H	17.094137	7.282659	7.741148
H	15.873679	10.467937	9.297465
H	17.357871	7.858933	9.923411
H	17.192401	9.266671	10.979384
H	15.766344	8.301191	10.555753
H	17.660857	10.858476	7.591988
H	18.338701	10.721384	9.228601
H	18.466164	9.367847	8.090050
H	25.863972	11.602947	5.637882
H	27.528946	11.569474	6.191360
H	27.099640	12.520834	4.751995
H	26.968555	14.660637	4.996116

H	25.836307	17.735091	5.516668
H	27.069636	16.788350	4.658050
H	27.506169	17.801991	6.051677
H	23.876769	12.792258	7.117706
H	23.556815	10.645104	5.850067
H	22.045799	11.542845	6.085184
H	22.386486	10.113909	7.065653
H	22.090738	11.508936	9.249566
H	21.661898	12.892323	8.225222
H	22.955557	13.036535	9.435469
H	23.611909	9.472029	8.834047
H	25.497846	8.376192	9.993469
H	27.678149	9.525913	10.159324
H	28.382457	12.869338	8.614361
H	29.684857	10.192534	9.345542
H	30.506701	11.657823	8.797183
H	29.424392	10.782603	7.697873
H	27.808559	13.038644	11.048272
H	29.564600	12.948041	10.788819
H	28.668301	11.526537	11.354073
H	28.382609	16.595794	8.515560
H	29.406338	18.634091	7.479394
H	30.507617	17.816279	8.603325
H	29.692426	19.306899	9.090432
H	28.711556	18.072215	11.180897
H	29.600886	16.625817	10.670828
H	27.849757	16.545807	10.963788
H	27.694800	20.005348	9.916653
H	25.510535	21.144794	9.728662
H	23.611771	19.997538	8.642614
H	23.860922	16.606340	7.069041
H	22.358678	19.274624	6.926613
H	22.008811	17.803072	6.014477
H	23.512078	18.695090	5.716720
H	22.973862	16.454333	9.408069
H	21.662707	16.544681	8.211562
H	22.100561	17.969989	9.172852
H	16.673629	11.496674	5.007476
H	15.520751	12.498530	4.171191
H	16.715284	14.576919	5.029302
H	18.521169	12.576096	3.581465
H	17.336254	13.613099	2.801901
H	18.568922	15.645103	3.631925
H	19.757419	14.606845	4.405031
H	19.187893	14.688651	1.395123
H	20.373168	13.648120	2.165843
H	20.447181	16.727309	2.195755
H	21.477897	15.680714	1.207051
H	21.642908	15.678047	2.970035
H	17.901598	13.548486	5.805260

184

[E] scf done: -3350.686244 / Energies= -3348.958590 / Enthalpies= -3348.957646 / Free Energies= -3349.203346

Mg	-4.921025	-0.234437	-1.703407
N	-6.560778	-1.513218	-1.796608
C	-6.556983	-2.569302	-2.759442
C	-6.147095	-3.865299	-2.351471
C	-6.076188	-4.876967	-3.310512
C	-6.386060	-4.632889	-4.645645
C	-6.777805	-3.358253	-5.032791

C	-6.875784	-2.308395	-4.112090
N	-5.449644	0.519205	0.169608
C	-6.736229	0.545792	0.504257
C	-7.745889	-0.208273	-0.132026
C	-7.671796	-1.187384	-1.143088
C	-8.991221	-1.846873	-1.490574
C	-5.834434	-4.171260	-0.892312
C	-7.058858	-4.751994	-0.166609
C	-7.339696	-0.942231	-4.601281
C	-8.741872	-1.010717	-5.224749
C	-4.476110	1.197803	0.967498
C	-3.887481	0.525478	2.069819
C	-2.895809	1.182733	2.803373
C	-2.469208	2.460775	2.466125
C	-3.042798	3.106917	1.377931
C	-4.047342	2.500188	0.613915
C	-4.329279	-0.870506	2.492963
C	-5.250658	-0.819707	3.722841
C	-4.658998	3.278613	-0.544186
C	-5.413731	4.519652	-0.039005
C	-3.150668	-1.813414	2.776102
C	-3.612021	3.674678	-1.593193
C	-7.230783	1.468254	1.603952
C	-4.634745	-5.113444	-0.713807
C	-6.343854	-0.319295	-5.588438
C	-2.700626	-1.553606	-0.903376
C	-2.175008	-1.442933	-2.133764
C	-1.945397	-2.568422	-3.094701
C	-2.519637	-2.318348	-4.496995
C	-2.156218	-3.427528	-5.483431
C	-2.730025	-3.192603	-6.879799
C	1.535014	4.399387	-0.618769
C	2.588960	3.681190	0.236703
C	2.045821	3.411279	1.650454
C	3.907033	4.439884	0.299818
C	5.137504	3.817105	-0.013756
C	6.351965	4.540215	0.085697
C	6.306052	5.874459	0.495518
C	5.099825	6.497087	0.802713
C	3.915282	5.776188	0.705196
N	5.167371	2.423631	-0.333452
Mg	5.329798	1.133025	1.263090
C	5.631657	1.555205	3.322103
C	4.820367	0.801442	4.384555
C	5.166432	1.179631	5.831428
C	4.352206	0.428202	6.886603
C	4.707968	0.808153	8.323924
C	3.891009	0.050886	9.369657
C	7.696788	3.890163	-0.205486
C	8.525802	4.683937	-1.227492
C	5.091678	2.028509	-1.601471
C	4.974297	3.070132	-2.692580
C	5.111328	0.683612	-2.016115
C	5.180776	-0.500917	-1.259660
C	5.170022	-1.797137	-2.038986
N	5.248720	-0.545602	0.070866
C	5.288286	-1.807019	0.746523
C	4.078594	-2.423775	1.147433
C	4.144838	-3.597061	1.901992
C	5.363207	-4.164005	2.255808

C	6.545083	-3.554526	1.849118
C	6.533889	-2.377377	1.093353
C	2.720324	-1.829989	0.802989
C	2.038368	-1.250142	2.051015
C	7.855501	-1.741768	0.680811
C	8.752453	-2.712410	-0.101568
C	8.604324	-1.181102	1.898319
C	1.796053	-2.841687	0.107869
C	8.495316	3.682413	1.089607
H	-2.455434	-4.001428	-7.567275
H	-6.436140	1.771035	2.283366
H	-7.642908	2.377061	1.147263
H	-8.034601	0.998879	2.175254
H	-8.744763	-0.039710	0.260354
H	-9.604597	-1.989799	-0.595856
H	-9.556015	-1.196802	-2.172617
H	-8.855369	-2.806526	-1.990901
H	-4.908767	-1.294877	1.665103
H	-6.146060	-0.218233	3.540662
H	-5.575986	-1.828102	4.004205
H	-4.724802	-0.385284	4.584794
H	-2.600098	-1.518935	3.677419
H	-3.517811	-2.832105	2.942081
H	-2.438344	-1.838029	1.945067
H	-2.447336	0.680062	3.659701
H	-1.699368	2.955670	3.051772
H	-2.711965	4.108216	1.117967
H	-5.383959	2.627875	-1.042178
H	-4.732009	5.239455	0.430540
H	-5.909525	5.029712	-0.875365
H	-6.175535	4.258055	0.701258
H	-3.128850	2.789830	-2.015988
H	-4.087597	4.220043	-2.416338
H	-2.838103	4.328298	-1.165217
H	-7.394006	-0.276562	-3.732096
H	-9.469860	-1.467873	-4.547875
H	-9.093604	-0.003020	-5.477801
H	-8.738389	-1.599354	-6.151213
H	-6.220231	-0.947183	-6.480810
H	-6.702841	0.663191	-5.919193
H	-5.363957	-0.174062	-5.122612
H	-7.024978	-3.170570	-6.076319
H	-6.323053	-5.434046	-5.377948
H	-5.776177	-5.877415	-3.009474
H	-5.589443	-3.216997	-0.409149
H	-7.375905	-5.691536	-0.631417
H	-6.823032	-4.957425	0.885520
H	-7.909004	-4.063805	-0.189994
H	-3.760317	-4.773260	-1.275334
H	-4.358365	-5.168695	0.345921
H	-4.862844	-6.135429	-1.039608
H	5.472723	2.639447	3.455892
H	6.704257	1.408505	3.538233
H	3.740839	0.981635	4.236681
H	4.954341	-0.285300	4.270826
H	5.024237	2.263398	5.962104
H	6.238372	0.995722	5.996551
H	4.497127	-0.655213	6.752199
H	3.280313	0.615041	6.717608
H	5.778350	0.622212	8.493360

H	4.564095	1.889838	8.459396
H	4.044085	-1.032414	9.284160
H	4.169795	0.342762	10.389840
H	2.817923	0.242053	9.250103
H	4.305703	-2.412266	-1.763125
H	6.059764	-2.395455	-1.811670
H	5.137649	-1.615767	-3.114605
H	5.055954	0.535057	-3.089142
H	4.080127	3.686390	-2.544863
H	4.917103	2.605966	-3.681998
H	5.829010	3.751690	-2.674448
H	2.882966	-1.001744	0.105622
H	2.264125	-3.264984	-0.788200
H	0.860093	-2.356926	-0.192932
H	1.537201	-3.675855	0.768954
H	1.826576	-2.036419	2.788272
H	1.084927	-0.775523	1.788829
H	2.668289	-0.504196	2.546328
H	3.221348	-4.078124	2.222469
H	5.394108	-5.076514	2.844672
H	7.496982	-3.999702	2.125215
H	7.625145	-0.901834	0.017991
H	9.069426	-3.559511	0.517378
H	9.658996	-2.198488	-0.445892
H	8.240154	-3.118081	-0.979752
H	7.992255	-0.457184	2.451421
H	9.528960	-0.678793	1.588135
H	8.879308	-1.980110	2.598210
H	7.503556	2.904052	-0.638479
H	7.971971	4.844949	-2.158361
H	9.446400	4.141309	-1.471306
H	8.816485	5.665525	-0.839660
H	8.734047	4.640065	1.565268
H	9.443094	3.165609	0.884224
H	7.933971	3.086582	1.818960
H	7.231413	6.437514	0.579124
H	5.084777	7.535121	1.122995
H	2.972648	6.263734	0.950647
H	2.781765	2.712440	-0.232165
H	1.243460	5.361159	-0.181393
H	0.627160	3.787417	-0.695800
H	1.900848	4.591116	-1.631843
H	2.775054	2.868266	2.264729
H	1.124443	2.822239	1.605961
H	1.822328	4.351173	2.169550
H	-1.856888	-0.456515	-2.475844
H	-0.856095	-2.706132	-3.185878
H	-3.610998	-2.227403	-4.433808
H	-2.151766	-1.353283	-4.874870
H	-1.062022	-3.520796	-5.542798
H	-2.525388	-4.388204	-5.096192
H	-2.359407	-2.251889	-7.306171
H	-3.824048	-3.134389	-6.848580
H	-4.294059	0.713510	-3.024302
H	-2.345458	-3.501521	-2.683602
H	-2.736160	-0.698754	-0.224481
H	-3.022460	-2.510860	-0.502986

184

[TS-EF] scf done: -3350.667974 / Energies= -3348.941821 / Enthalpies= -3348.940877 / Free Energies= -3349.183124

Mg	15.850313	12.959545	6.896545
N	14.303282	11.608524	7.112794
C	14.448423	10.327837	6.497269
C	15.150196	9.309513	7.191010
C	15.342676	8.078292	6.557231
C	14.876350	7.847286	5.267652
C	14.205108	8.857936	4.591489
C	13.974947	10.105097	5.182303
N	15.043263	14.367181	8.170423
C	13.785027	14.278093	8.594882
C	12.930829	13.184172	8.351338
C	13.162936	11.951954	7.706731
C	11.982649	11.001248	7.688395
C	15.658253	9.525021	8.610618
C	14.618078	9.083716	9.652910
C	13.246725	11.176770	4.382108
C	11.897253	10.683444	3.838555
C	15.863016	15.475876	8.544426
C	16.537066	15.474244	9.791143
C	17.413282	16.525115	10.080351
C	17.623884	17.563860	9.181006
C	16.947286	17.563093	7.967238
C	16.064867	16.533730	7.623583
C	16.341510	14.374086	10.826858
C	15.880809	14.935234	12.181545
C	15.315312	16.612173	6.299898
C	14.258938	17.728795	6.328702
C	17.613894	13.534809	11.015107
C	16.252486	16.796828	5.099386
C	13.192388	15.391207	9.433634
C	16.999966	8.836086	8.888113
C	14.123748	11.705069	3.237689
C	18.037789	12.360525	7.108305
C	18.049300	12.887529	5.812438
C	18.298883	12.029495	4.590956
C	18.210948	12.751824	3.248508
C	18.530535	11.845333	2.059204
C	18.453739	12.571536	0.717891
C	22.111148	18.006925	7.998312
C	23.087168	17.118230	8.781771
C	22.460124	16.666170	10.109987
C	24.431338	17.791076	9.021383
C	25.652427	17.163868	8.681059
C	26.886495	17.798411	8.956022
C	26.871528	19.057799	9.562919
C	25.677487	19.686162	9.896446
C	24.471224	19.050387	9.627372
N	25.641782	15.838181	8.142270
Mg	25.676844	14.301771	9.512430
C	25.846139	14.369907	11.626998
C	24.949017	13.473808	12.494012
C	25.195914	13.598979	14.003058
C	24.297635	12.703404	14.857974
C	24.537679	12.842759	16.362218
C	23.638190	11.940559	17.205027
C	28.220094	17.134064	8.644372
C	29.142940	18.025938	7.802299
C	25.616118	15.659221	6.822749
C	25.590907	16.868124	5.913375
C	25.612528	14.399733	6.192101

C	25.622785	13.105794	6.747817
C	25.608887	11.954589	5.766220
N	25.637375	12.846220	8.054924
C	25.634495	11.490336	8.514411
C	24.405356	10.828265	8.745385
C	24.430751	9.541721	9.291945
C	25.629259	8.910360	9.603599
C	26.830570	9.568259	9.368274
C	26.860579	10.856268	8.825009
C	23.065107	11.485633	8.445574
C	22.330740	11.854491	9.743576
C	28.202464	11.539912	8.601972
C	29.131627	10.718325	7.696794
C	28.892822	11.857481	9.937310
C	22.167652	10.615934	7.553720
C	28.925937	16.694325	9.936831
H	18.682007	11.898416	-0.116487
H	13.579150	16.370365	9.140641
H	12.102273	15.399228	9.354501
H	13.447058	15.247003	10.490234
H	11.933038	13.293518	8.762890
H	11.322731	11.182698	8.540665
H	11.394393	11.154565	6.775503
H	12.297343	9.955132	7.696521
H	15.558792	13.701809	10.460047
H	14.994878	15.571388	12.081097
H	15.636424	14.117363	12.870019
H	16.665030	15.537971	12.654769
H	18.448787	14.151669	11.369144
H	17.446623	12.743419	11.756265
H	17.921627	13.059325	10.079074
H	17.938478	16.530794	11.032846
H	18.306081	18.373329	9.428419
H	17.101145	18.384332	7.271117
H	14.787224	15.662658	6.157653
H	14.726482	18.711453	6.467156
H	13.700511	17.753215	5.384877
H	13.538786	17.586425	7.141512
H	16.981900	15.983457	5.037993
H	15.674629	16.796408	4.167458
H	16.798051	17.746958	5.148480
H	13.047698	12.020855	5.050964
H	11.265030	10.267519	4.630758
H	11.353267	11.510654	3.367074
H	12.027072	9.903747	3.078540
H	14.388990	10.901384	2.539613
H	13.591826	12.480448	2.672038
H	15.048630	12.145442	3.625166
H	13.849569	8.676799	3.579811
H	15.039102	6.883430	4.791552
H	15.869768	7.284803	7.079974
H	15.807309	10.603901	8.741627
H	14.386118	8.017138	9.542467
H	14.999900	9.243213	10.668977
H	13.683755	9.644455	9.555534
H	17.743300	9.063752	8.116421
H	17.398682	9.169903	9.853090
H	16.898164	7.745586	8.943308
H	25.698140	15.420722	11.930925
H	26.898404	14.156669	11.887553

H	23.887792	13.703001	12.303700
H	25.075962	12.417968	12.208312
H	25.055638	14.649596	14.300650
H	26.251082	13.364166	14.211676
H	24.446459	11.652802	14.564366
H	23.242804	12.931076	14.638971
H	25.591345	12.618336	16.582563
H	24.384262	13.891058	16.656722
H	23.797768	10.882741	16.961120
H	23.830164	12.066952	18.277124
H	22.578095	12.162118	17.028560
H	24.687085	11.371051	5.868436
H	26.436598	11.264623	5.960638
H	25.680907	12.308948	4.735616
H	25.597538	14.431182	5.108659
H	24.713852	17.491167	6.120393
H	25.570799	16.576050	4.861241
H	26.468198	17.501452	6.084278
H	23.262883	12.416755	7.903075
H	22.674119	10.329637	6.624939
H	21.252917	11.160167	7.290197
H	21.864306	9.692941	8.062118
H	22.094936	10.957579	10.329154
H	21.388084	12.369367	9.521897
H	22.937337	12.507836	10.380627
H	23.492032	9.025685	9.479995
H	25.626773	7.909721	10.028701
H	27.767135	9.072884	9.614343
H	28.010176	12.490996	8.092834
H	29.410690	9.765350	8.161752
H	30.057748	11.271513	7.499035
H	28.661359	10.491943	6.733352
H	28.257711	12.476333	10.581106
H	29.834808	12.393755	9.768339
H	29.124849	10.939008	10.489878
H	28.014101	16.231827	8.057635
H	28.654285	18.356057	6.878734
H	30.052310	17.478473	7.526607
H	29.454735	18.922259	8.351477
H	29.170804	17.559675	10.564691
H	29.861552	16.168658	9.708896
H	28.294118	16.026719	10.533763
H	27.814192	19.553617	9.782811
H	25.687203	20.665426	10.368608
H	23.538847	19.540448	9.897892
H	23.265719	16.220920	8.178762
H	21.831683	18.902859	8.565512
H	21.187784	17.457310	7.779974
H	22.541802	18.338853	7.046785
H	23.128023	15.994402	10.662160
H	21.513043	16.141745	9.934091
H	22.254792	17.526012	10.759353
H	18.372391	13.923759	5.709630
H	19.318764	11.630957	4.715282
H	17.206728	13.178479	3.132448
H	18.908717	13.603210	3.251219
H	19.534480	11.414503	2.185399
H	17.833556	10.995070	2.055416
H	19.165385	13.405601	0.676820
H	17.451658	12.984449	0.548943

H	16.439229	13.432496	5.231131
H	17.634166	11.156310	4.604756
H	18.380713	12.980040	7.933743
H	18.053875	11.281871	7.252976

184

[F] scf done: -3350.729943 / Energies= -3348.997816 / Enthalpies= -3348.996872 / Free Energies= -3349.244362

Mg	-5.618064	-1.019804	-1.094307
N	-7.458154	-1.924682	-0.908810
C	-7.591660	-3.210355	-1.522359
C	-7.255810	-4.373688	-0.791481
C	-7.300980	-5.610155	-1.442146
C	-7.666952	-5.711407	-2.779274
C	-7.993153	-4.561364	-3.488312
C	-7.963291	-3.300990	-2.883911
N	-6.196360	0.652344	-0.040329
C	-7.407744	0.803909	0.495787
C	-8.451978	-0.134111	0.386543
C	-8.487481	-1.386196	-0.258218
C	-9.794269	-2.144709	-0.184430
C	-6.811952	-4.309228	0.663359
C	-7.618486	-5.250495	1.568894
C	-8.293973	-2.066144	-3.709879
C	-9.654373	-2.173167	-4.412710
C	-5.210335	1.676690	0.125364
C	-4.283760	1.590426	1.190895
C	-3.265870	2.545549	1.273681
C	-3.154339	3.567191	0.338212
C	-4.074518	3.646780	-0.700621
C	-5.111008	2.717296	-0.828508
C	-4.353395	0.485430	2.235752
C	-4.433145	1.038543	3.666116
C	-6.082718	2.838383	-1.994161
C	-6.736038	4.225662	-2.072606
C	-3.168973	-0.483838	2.099303
C	-5.395822	2.493045	-3.324122
C	-7.715030	2.059198	1.282401
C	-5.307448	-4.596995	0.788545
C	-7.180270	-1.774964	-4.728122
C	-3.842297	-1.762658	-1.988551
C	-3.072329	-0.894246	-2.994945
C	-1.816272	-1.557012	-3.575137
C	-1.062523	-0.688094	-4.583554
C	0.183078	-1.357749	-5.166341
C	0.930307	-0.478922	-6.167717
C	1.997308	4.549325	-0.321138
C	2.995435	3.680130	0.455685
C	2.354060	3.139211	1.743228
C	4.295767	4.407488	0.765837
C	5.556125	3.837200	0.470305
C	6.748710	4.517836	0.809067
C	6.654151	5.765112	1.433757
C	5.421380	6.337119	1.724556
C	4.255675	5.656415	1.392784
N	5.625986	2.521908	-0.088784
Mg	5.688288	0.962925	1.254301
C	5.784025	1.002113	3.373965
C	4.880750	0.071673	4.197422
C	5.068968	0.186092	5.715700
C	4.164938	-0.742345	6.528471

C	4.349766	-0.615023	8.041559
C	3.444339	-1.548304	8.843137
C	8.122216	3.916885	0.545200
C	9.031939	4.855313	-0.260047
C	5.654522	2.364331	-1.411225
C	5.602110	3.585783	-2.302406
C	5.734662	1.117419	-2.060927
C	5.791748	-0.184560	-1.527008
C	5.879965	-1.315421	-2.528044
N	5.769731	-0.467880	-0.224942
C	5.824078	-1.829633	0.213234
C	4.624388	-2.557602	0.395442
C	4.698427	-3.847826	0.928596
C	5.917287	-4.420638	1.272702
C	7.090053	-3.699890	1.081725
C	7.070917	-2.405222	0.553883
C	3.263141	-1.969503	0.050403
C	2.451681	-1.668666	1.319655
C	8.383287	-1.654194	0.376742
C	9.374850	-2.418325	-0.512745
C	9.021941	-1.321640	1.733907
C	2.458770	-2.871963	-0.896231
C	8.800058	3.504671	1.861435
H	1.816711	-0.985772	-6.567314
H	-7.604008	2.950083	0.654854
H	-8.731234	2.040089	1.681963
H	-7.013809	2.177331	2.115688
H	-9.375266	0.151937	0.877031
H	-10.555581	-1.577154	0.355181
H	-10.170531	-2.369689	-1.188338
H	-9.656470	-3.108521	0.318022
H	-5.271578	-0.085268	2.056619
H	-5.277063	1.726833	3.787749
H	-4.558613	0.218990	4.383906
H	-3.521013	1.580261	3.942906
H	-2.216764	0.028314	2.283097
H	-3.253859	-1.302747	2.824118
H	-3.118370	-0.919595	1.095016
H	-2.546035	2.486824	2.086905
H	-2.355133	4.299714	0.419324
H	-3.985124	4.447664	-1.431028
H	-6.883900	2.107577	-1.838461
H	-6.002324	5.007801	-2.301006
H	-7.492738	4.244616	-2.866060
H	-7.226434	4.497288	-1.131084
H	-4.956772	1.489489	-3.302831
H	-6.112874	2.535695	-4.153345
H	-4.585386	3.198566	-3.544051
H	-8.347048	-1.212320	-3.025203
H	-10.461229	-2.381031	-3.700946
H	-9.891098	-1.234748	-4.928399
H	-9.661600	-2.970898	-5.164758
H	-7.084900	-2.593761	-5.451640
H	-7.394658	-0.855467	-5.286736
H	-6.206349	-1.659146	-4.237953
H	-8.273440	-4.642277	-4.536056
H	-7.694448	-6.682328	-3.267602
H	-7.038587	-6.510654	-0.891776
H	-6.983894	-3.287281	1.019220
H	-7.446071	-6.303751	1.317876

H	-7.327254	-5.111980	2.616994
H	-8.695350	-5.063516	1.489796
H	-4.712604	-3.908479	0.177280
H	-4.978888	-4.501429	1.830875
H	-5.073917	-5.614973	0.453900
H	5.599330	2.044419	3.687209
H	6.831859	0.811284	3.667441
H	3.821204	0.273859	3.970304
H	5.047397	-0.976898	3.905556
H	4.890059	1.229222	6.018798
H	6.121810	-0.023186	5.960203
H	4.350605	-1.785215	6.228431
H	3.112745	-0.538711	6.275628
H	5.400427	-0.816838	8.295642
H	4.161126	0.425814	8.341991
H	3.636378	-2.598939	8.591744
H	3.598231	-1.431465	9.922447
H	2.385699	-1.348772	8.634642
H	4.990253	-1.952590	-2.473492
H	6.737951	-1.960892	-2.312967
H	5.971225	-0.937523	-3.548693
H	5.754765	1.167275	-3.143526
H	4.689795	4.163269	-2.116554
H	5.631320	3.310662	-3.358870
H	6.441821	4.257046	-2.091941
H	3.431863	-1.018234	-0.466204
H	3.024459	-3.117931	-1.801912
H	1.531233	-2.373175	-1.201094
H	2.178882	-3.816671	-0.415194
H	2.240920	-2.588440	1.878547
H	1.491717	-1.203322	1.064069
H	2.990560	-0.994452	1.994669
H	3.782218	-4.414295	1.078882
H	5.952806	-5.425223	1.686853
H	8.042877	-4.150519	1.350203
H	8.159304	-0.706658	-0.126032
H	9.682489	-3.365441	-0.054059
H	10.280404	-1.821166	-0.674544
H	8.943388	-2.649933	-1.492963
H	8.339705	-0.745148	2.368708
H	9.939394	-0.736032	1.596725
H	9.286288	-2.234894	2.280538
H	7.980049	3.009243	-0.052009
H	8.562768	5.164168	-1.200907
H	9.976772	4.353899	-0.502021
H	9.278050	5.764144	0.301803
H	8.978950	4.377474	2.500991
H	9.767971	3.026429	1.666552
H	8.179591	2.803864	2.431594
H	7.564762	6.295653	1.702125
H	5.368997	7.307551	2.211901
H	3.292610	6.103007	1.628897
H	3.238285	2.819346	-0.177200
H	1.646990	5.400214	0.275210
H	1.116017	3.958656	-0.598637
H	2.439321	4.948925	-1.240876
H	3.043162	2.487327	2.293232
H	1.447899	2.564651	1.514742
H	2.074487	3.958202	2.417341
H	-2.776892	0.058424	-2.528145

H	-1.141762	-1.826597	-2.747681
H	-1.741687	-0.413099	-5.405455
H	-0.772201	0.259000	-4.102969
H	0.860924	-1.636117	-4.346516
H	-0.107342	-2.300903	-5.651208
H	1.263516	0.456920	-5.701483
H	0.288852	-0.212356	-7.017109
H	-3.727814	-0.616967	-3.836908
H	-2.102975	-2.506835	-4.052056
H	-3.146368	-2.056456	-1.182825
H	-4.111754	-2.715149	-2.477471

199
[G] scf done: -3873.549815 / Energies= -3871.691918 / Enthalpies= -3871.690974 / Free Energies= -3871.968673

C	26.977003	10.836563	10.125609
C	26.167833	11.622411	9.272167
C	24.853389	11.209139	8.949015
C	24.373595	10.014732	9.494191
C	25.159678	9.235337	10.335181
C	26.449798	9.649177	10.643329
N	26.649223	12.887083	8.805631
C	27.233720	12.973230	7.610207
C	27.400861	11.721090	6.777526
C	23.944261	12.043073	8.056921
C	23.330542	11.228625	6.909533
C	28.392050	11.249751	10.504219
C	28.491285	11.593020	11.998434
C	27.714938	14.167787	7.041177
C	27.707474	15.483922	7.543567
C	28.291003	16.550562	6.643350
N	27.227473	15.829783	8.736446
C	27.279747	17.196217	9.158068
C	26.175679	18.046553	8.916652
C	26.211846	19.350739	9.418887
C	27.303586	19.817546	10.141591
C	28.381933	18.972222	10.375077
C	28.394232	17.658910	9.895741
C	24.943735	17.566509	8.163051
C	23.758825	17.370623	9.122192
C	29.580640	16.758721	10.210414
C	29.605829	16.388693	11.701844
C	24.553145	18.498881	7.008000
C	30.920056	17.375729	9.784428
C	22.840738	12.722360	8.882502
C	29.430063	10.183559	10.124731
H	23.253277	16.288935	2.510935
Si	21.827442	16.041724	2.165424
C	20.742286	17.284276	3.055610
C	19.373435	17.039253	3.255338
C	18.561638	17.975458	3.894187
C	19.107010	19.176365	4.348938
C	20.463925	19.436431	4.163503
C	21.272381	18.497557	3.523469
H	21.691324	16.139348	0.686388
C	14.658278	12.001080	4.577373
C	15.587146	12.908234	3.756722
C	15.412381	12.781656	2.237771
C	16.341635	13.682627	1.422561
C	16.167119	13.542666	-0.090689
C	17.098978	14.446992	-0.895025

Mg	26.390901	14.496658	10.063484
C	17.957588	9.689499	6.196620
C	17.269725	9.186047	7.475357
C	15.942279	8.518147	7.146918
C	14.704412	9.135756	7.441366
C	13.487172	8.508825	7.086766
C	13.535174	7.266120	6.447968
C	14.745592	6.647196	6.158909
C	15.935753	7.275652	6.506013
N	14.686246	10.445590	8.017720
C	14.635970	10.586145	9.341139
C	14.608043	9.351834	10.215503
C	12.138201	9.163930	7.347406
C	11.496846	9.634033	6.032341
C	18.211681	8.274244	8.273348
C	14.607061	11.826848	10.007664
C	14.647556	13.136978	9.493713
C	14.609951	14.256294	10.510931
N	14.720916	13.437544	8.196751
C	14.786201	14.806227	7.783367
C	13.600362	15.481790	7.410498
C	13.699347	16.784959	6.913490
C	14.929499	17.418932	6.785292
C	16.088409	16.747607	7.158567
C	16.043751	15.443455	7.660949
C	12.229997	14.829346	7.524455
C	11.616394	14.575580	6.138936
C	17.342157	14.740944	8.032829
C	18.147641	14.375021	6.776627
C	11.176351	8.250939	8.120642
C	11.267961	15.646337	8.399094
C	18.202775	15.565380	9.000638
C	25.699057	14.825145	12.041836
C	24.365619	14.218938	12.504887
C	24.009898	14.519446	13.966597
C	22.674331	13.927879	14.420772
C	22.328183	14.236413	15.878442
C	20.991621	13.644946	16.322474
Mg	14.733658	12.024879	6.697980
H	15.537666	14.838609	10.483000
H	13.797094	14.956413	10.291242
H	14.476045	13.869613	11.523528
H	14.555044	11.762772	11.088443
H	14.573648	9.613833	11.275128
H	13.739387	8.726897	9.981207
H	15.493368	8.731609	10.037059
H	17.082596	13.805627	8.540543
H	17.644692	15.847917	9.900391
H	19.079877	14.987361	9.315192
H	18.568271	16.487725	8.534437
H	18.454359	15.273809	6.228400
H	19.054450	13.819468	7.045972
H	17.561008	13.756356	6.087747
H	17.050335	17.243962	7.055091
H	14.983830	18.433774	6.398702
H	12.794228	17.312013	6.620159
H	12.365082	13.856597	8.010697
H	11.046184	16.623198	7.953182
H	10.315991	15.114867	8.517478
H	11.681809	15.824357	9.397918

H	12.275448	13.962876	5.513606
H	10.653684	14.057821	6.231360
H	11.439205	15.517653	5.605862
H	12.312554	10.051709	7.965530
H	11.618882	7.897755	9.058870
H	10.253598	8.791074	8.364421
H	10.893285	7.368340	7.534842
H	11.285775	8.783942	5.372235
H	10.549797	10.152924	6.225892
H	12.156477	10.316550	5.484474
H	12.605763	6.776352	6.167042
H	14.761519	5.680997	5.660776
H	16.881419	6.792826	6.270899
H	17.056338	10.060210	8.100765
H	18.531668	7.404702	7.687076
H	19.115455	8.823423	8.563613
H	17.735120	7.901487	9.186976
H	17.309556	10.370752	5.632565
H	18.886482	10.221011	6.437353
H	18.209725	8.855297	5.530557
H	25.660796	15.918587	12.189224
H	26.489491	14.485305	12.735068
H	23.541888	14.586833	11.871542
H	24.374780	13.126420	12.367724
H	23.994851	15.610691	14.111121
H	24.815992	14.140348	14.613566
H	22.690935	12.836455	14.276971
H	21.869493	14.305751	13.771184
H	23.130239	13.857391	16.528169
H	22.312645	15.326411	16.022572
H	20.989832	12.552043	16.224812
H	20.771693	13.885244	17.369425
H	20.165528	14.030916	15.712023
H	26.425647	11.308597	6.495800
H	27.915626	10.939618	7.346224
H	27.966640	11.922483	5.865339
H	28.155379	14.058280	6.056814
H	27.544920	17.322332	6.423931
H	28.642792	16.127784	5.699852
H	29.128487	17.059778	7.132559
H	24.552021	12.835835	7.607263
H	24.098671	10.721151	6.315221
H	22.763502	11.885293	6.238919
H	22.638987	10.462086	7.278857
H	22.191035	11.977477	9.357988
H	22.215511	13.359254	8.244539
H	23.259069	13.344537	9.681737
H	23.362916	9.689973	9.258146
H	24.767695	8.309572	10.749021
H	27.062805	9.037718	11.301709
H	28.636218	12.156125	9.938455
H	29.280726	9.254878	10.688289
H	30.442959	10.542460	10.343816
H	29.383182	9.935294	9.058545
H	27.778979	12.377071	12.278584
H	29.500784	11.941964	12.248520
H	28.277105	10.716232	12.621492
H	29.454254	15.830278	9.642424
H	30.922212	17.644669	8.722126
H	31.737490	16.664739	9.954513

H	31.150858	18.282529	10.356011
H	29.745911	17.279597	12.326171
H	30.429126	15.695797	11.915727
H	28.668574	15.914598	12.015113
H	29.231963	19.337515	10.946725
H	27.312413	20.835044	10.524437
H	25.366048	20.011554	9.243856
H	25.180304	16.589545	7.726758
H	24.243236	19.486491	7.369465
H	23.710605	18.076578	6.447771
H	25.384976	18.647263	6.310096
H	24.008155	16.679236	9.935936
H	22.888002	16.971992	8.587381
H	23.464651	18.321581	9.582874
H	14.822108	10.954172	4.267624
H	13.616836	12.213130	4.275402
H	15.434788	13.962140	4.037512
H	15.574373	11.732203	1.947651
H	14.366256	13.008015	1.980223
H	16.173657	14.732564	1.709035
H	17.387423	13.459237	1.686142
H	15.123124	13.763993	-0.355491
H	16.335769	12.494685	-0.377562
H	16.929643	15.504395	-0.656060
H	16.949493	14.322634	-1.974031
H	18.151551	14.225024	-0.678367
H	21.466412	14.653814	2.560141
H	18.930141	16.106766	2.911063
H	17.504842	17.765295	4.038838
H	18.475413	19.908106	4.846957
H	20.893965	20.370023	4.518126
H	22.330513	18.714577	3.389599

199

[TS-GH] scf done: -3873.511759 / Energies= -3871.654823 / Enthalpies= -3871.653879 / Free Energies= -3871.913181

C	27.696919	11.116438	9.573384
C	26.611380	11.757533	8.931671
C	25.378044	11.084648	8.761708
C	25.255568	9.780954	9.251310
C	26.313471	9.143237	9.889078
C	27.522291	9.811034	10.043464
N	26.734597	13.127808	8.536249
C	27.100498	13.429477	7.291087
C	27.384691	12.310938	6.312843
C	24.181702	11.748295	8.093486
C	23.573019	10.887861	6.977426
C	29.039184	11.806385	9.772770
C	29.322602	12.060756	11.261197
C	27.241225	14.741182	6.797567
C	27.042558	15.979966	7.437307
C	27.269718	17.215716	6.594625
N	26.673989	16.118435	8.709789
C	26.493067	17.424677	9.265095
C	25.214609	18.028945	9.229722
C	25.038806	19.262377	9.863994
C	26.088692	19.895797	10.518531
C	27.340349	19.292342	10.548015
C	27.568179	18.058501	9.930800
C	24.026735	17.357481	8.555907
C	23.027243	16.838928	9.601789

C	28.947569	17.421342	10.019593
C	29.245687	16.952494	11.452463
C	23.322186	18.274966	7.546635
C	30.058957	18.352312	9.514743
C	23.109587	12.119190	9.129556
C	30.194032	11.023258	9.131255
H	19.869437	12.911470	6.454541
Si	18.548804	13.033395	5.669708
C	18.935628	14.784611	5.028025
C	17.995218	15.807198	4.834858
C	18.377438	17.073222	4.390067
C	19.715130	17.336489	4.100224
C	20.668988	16.334616	4.280883
C	20.281817	15.084159	4.760417
H	18.799137	11.803414	4.833176
C	16.579474	12.910390	4.764714
C	16.835474	13.036167	3.255215
C	15.699372	12.556831	2.346867
C	16.008396	12.736270	0.859190
C	14.883190	12.261163	-0.061452
C	15.198033	12.450408	-1.544042
Mg	26.318680	14.537412	9.978865
C	18.937302	9.593097	7.707109
C	17.513217	9.648845	8.273917
C	16.458268	9.183165	7.279052
C	15.256147	9.899751	7.064687
C	14.268736	9.403058	6.179166
C	14.519209	8.203049	5.506419
C	15.698140	7.494139	5.702794
C	16.651194	7.982417	6.588231
N	15.073150	11.172173	7.695974
C	14.298069	11.278326	8.774886
C	13.680346	10.041762	9.393345
C	12.952158	10.127979	5.929152
C	12.897216	10.725794	4.516225
C	17.428902	8.846673	9.582942
C	13.953054	12.493525	9.398997
C	14.225247	13.828275	9.044942
C	13.592416	14.877211	9.934362
N	14.998817	14.204368	8.023661
C	15.170319	15.599014	7.740584
C	14.364758	16.203891	6.744321
C	14.565042	17.556941	6.451761
C	15.540667	18.301067	7.104815
C	16.336063	17.691868	8.067026
C	16.172030	16.345362	8.406842
C	13.268301	15.438980	6.013915
C	13.339374	15.615469	4.490675
C	17.081884	15.739731	9.467272
C	18.541056	15.683086	8.990097
C	11.735619	9.220508	6.167205
C	11.872853	15.832421	6.524550
C	17.000712	16.495750	10.802924
C	25.887208	14.510279	12.056908
C	24.794610	13.572823	12.592886
C	24.642098	13.583733	14.119495
C	23.554420	12.644519	14.643803
C	23.420165	12.644029	16.167591
C	22.334271	11.699413	16.679469
Mg	16.045086	12.813118	6.950866

H	13.252360	15.744374	9.363111
H	12.748022	14.462341	10.489572
H	14.324978	15.244041	10.662627
H	13.325900	12.381090	10.276774
H	13.551197	10.177302	10.470622
H	12.687411	9.860936	8.964440
H	14.280720	9.147661	9.213654
H	16.751574	14.710352	9.646650
H	15.971610	16.584607	11.167458
H	17.589065	15.976311	11.568502
H	17.405024	17.510954	10.712525
H	18.938270	16.690653	8.818677
H	19.170420	15.198320	9.746203
H	18.644749	15.126264	8.054434
H	17.105571	18.274327	8.567749
H	15.681364	19.351675	6.863309
H	13.941985	18.036790	5.700892
H	13.405718	14.373310	6.233102
H	11.680328	16.898579	6.353067
H	11.095871	15.261959	6.001006
H	11.763741	15.641565	7.596942
H	14.331525	15.368454	4.097814
H	12.607802	14.962748	3.999869
H	13.111305	16.644175	4.187928
H	12.884129	10.962080	6.635373
H	11.762834	8.752456	7.157194
H	10.808245	9.800633	6.089564
H	11.679769	8.415629	5.424853
H	12.968275	9.942974	3.751379
H	11.952170	11.261001	4.360672
H	13.717865	11.429591	4.345975
H	13.773112	7.814607	4.817143
H	15.871495	6.561706	5.171425
H	17.566256	7.418416	6.748897
H	17.299607	10.695076	8.523894
H	17.614960	7.781234	9.399945
H	18.179973	9.201776	10.298952
H	16.445135	8.937502	10.055174
H	19.016873	10.129542	6.756429
H	19.639182	10.052736	8.411768
H	19.269569	8.561201	7.541259
H	25.653002	15.543896	12.364705
H	26.829384	14.275783	12.584934
H	23.820490	13.836497	12.150007
H	24.989963	12.537219	12.273587
H	24.430468	14.612004	14.450932
H	25.607790	13.312584	14.573449
H	23.763697	11.619788	14.299884
H	22.587096	12.920999	14.196348
H	24.386156	12.368060	16.614576
H	23.208862	13.666475	16.512747
H	22.535581	10.664035	16.377011
H	22.264784	11.718664	17.773600
H	21.350031	11.973115	16.278939
H	26.482088	11.716060	6.134169
H	28.136342	11.621879	6.712216
H	27.738125	12.699348	5.355216
H	27.546779	14.807448	5.759685
H	26.352268	17.810681	6.523552
H	27.593988	16.955617	5.584625

H	28.025648	17.864670	7.049703
H	24.528826	12.680253	7.633847
H	24.322051	10.607026	6.228005
H	22.771097	11.437010	6.470676
H	23.134954	9.962577	7.369814
H	22.717602	11.222559	9.625226
H	22.269216	12.635027	8.649851
H	23.513582	12.770516	9.913193
H	24.311092	9.255298	9.132012
H	26.196052	8.129101	10.263067
H	28.349615	9.309356	10.540409
H	28.989643	12.780225	9.272594
H	30.337739	10.048831	9.613047
H	31.133617	11.580682	9.228035
H	30.016970	10.842489	8.065205
H	28.525844	12.650983	11.727309
H	30.268261	12.602399	11.387398
H	29.400893	11.117929	11.816021
H	28.946657	16.534584	9.375783
H	29.865684	18.695330	8.492088
H	31.023918	17.831202	9.520133
H	30.162697	19.240940	10.148519
H	29.278535	17.801809	12.145652
H	30.216421	16.443384	11.499376
H	28.478190	16.261119	11.818563
H	28.158563	19.787428	11.066131
H	25.931279	20.854434	11.006725
H	24.058554	19.733010	9.848651
H	24.403435	16.491171	8.001000
H	22.852851	19.135399	8.038357
H	22.531947	17.727134	7.020075
H	24.022082	18.664013	6.798137
H	23.505711	16.156067	10.314108
H	22.200281	16.305433	9.118187
H	22.602553	17.667634	10.181569
H	16.189903	11.877778	4.920939
H	15.729051	13.606241	4.971308
H	17.069837	14.081683	3.021727
H	15.494134	11.495447	2.549905
H	14.774645	13.101065	2.591411
H	16.218272	13.797422	0.657253
H	16.932921	12.193137	0.612337
H	13.958636	12.801640	0.188472
H	14.676821	11.199625	0.137208
H	15.376311	13.507042	-1.779265
H	14.373955	12.102870	-2.177914
H	16.097695	11.892808	-1.832427
H	17.882353	12.872145	7.131076
H	16.943182	15.627053	5.043520
H	17.627959	17.851918	4.270867
H	20.014233	18.318708	3.741361
H	21.715434	16.531107	4.057616
H	21.039887	14.322900	4.938209
H	17.742951	12.464802	3.009457

199

[H] scf done: -3873.564272 / Energies= -3871.707369 / Enthalpies= -3871.706425 / Free Energies= -3871.980767

C	27.790703	11.066671	9.457563
C	26.837586	11.696419	8.623281
C	25.693127	10.990620	8.181363

C	25.524293	9.665200	8.592566
C	26.453248	9.037356	9.414528
C	27.575472	9.738382	9.838378
N	26.985037	13.083879	8.303324
C	27.577685	13.444675	7.165109
C	28.095647	12.373367	6.230722
C	24.633192	11.643446	7.304857
C	24.293130	10.807601	6.063279
C	29.030943	11.792003	9.960601
C	28.983934	11.986077	11.483909
C	27.762234	14.777494	6.749945
C	27.413440	15.984876	7.386331
C	27.760887	17.259604	6.649599
N	26.811783	16.062376	8.571647
C	26.529809	17.340738	9.149568
C	25.275973	17.954057	8.920872
C	24.988253	19.153760	9.578546
C	25.905144	19.745610	10.439603
C	27.135039	19.135139	10.654899
C	27.470948	17.934032	10.023237
C	24.228973	17.324633	8.012986
C	23.083223	16.717850	8.838180
C	28.816830	17.286403	10.316389
C	28.865209	16.748073	11.754908
C	23.678858	18.306862	6.969900
C	29.996183	18.233565	10.054422
C	23.362468	11.943445	8.114591
C	30.326820	11.078836	9.547636
H	20.509860	12.287186	4.897536
Si	20.107560	13.000625	3.649874
C	19.986531	14.849903	3.966224
C	18.978879	15.379018	4.792685
C	18.885586	16.750767	5.023466
C	19.799483	17.623290	4.431316
C	20.805599	17.119076	3.608207
C	20.895040	15.745599	3.379373
H	21.200216	12.792460	2.652758
C	18.490054	12.268098	3.013721
C	17.989806	12.881929	1.699343
C	16.677337	12.268236	1.204848
C	16.168973	12.885858	-0.098514
C	14.858337	12.273510	-0.595548
C	14.356211	12.898513	-1.895573
Mg	26.240802	14.422865	9.678192
C	18.585955	10.712723	9.141626
C	17.281957	10.152572	9.731616
C	16.459809	9.454464	8.657893
C	15.261393	10.009578	8.153396
C	14.541769	9.356327	7.125916
C	15.039196	8.149965	6.623923
C	16.213945	7.592218	7.114967
C	16.913525	8.245020	8.122822
N	14.827689	11.293104	8.617216
C	13.988290	11.383764	9.648488
C	13.500223	10.117137	10.315472
C	13.271994	9.949650	6.532886
C	13.534943	10.502069	5.122884
C	17.579329	9.243078	10.931979
C	13.510129	12.597354	10.180958
C	13.780416	13.927535	9.804275

C	13.098139	15.009159	10.611642
N	14.585808	14.275914	8.801267
C	14.806515	15.657300	8.498698
C	14.007681	16.294400	7.520638
C	14.304669	17.614833	7.170102
C	15.359212	18.300300	7.762458
C	16.139533	17.662401	8.720230
C	15.884835	16.342487	9.105168
C	12.864878	15.571442	6.822354
C	13.226029	15.257014	5.361783
C	16.791968	15.671051	10.126449
C	18.171388	15.372534	9.517784
C	12.103996	8.954059	6.514533
C	11.541753	16.345788	6.898073
C	16.938030	16.488496	11.417493
C	25.370931	14.351741	11.612926
C	24.206111	13.394542	11.905607
C	23.700378	13.441222	13.353335
C	22.534821	12.493095	13.640573
C	22.037026	12.549543	15.085903
C	20.872341	11.601074	15.363919
Mg	15.557505	12.920171	7.619611
H	12.502242	15.661567	9.964104
H	12.444899	14.584033	11.376599
H	13.837911	15.650995	11.102990
H	12.832173	12.490709	11.019894
H	12.801657	10.336835	11.125620
H	13.003246	9.462089	9.591736
H	14.340828	9.545874	10.725061
H	16.336715	14.712081	10.398081
H	15.964169	16.711335	11.867978
H	17.532253	15.932941	12.152645
H	17.447381	17.442840	11.240020
H	18.688827	16.299317	9.242441
H	18.804129	14.835004	10.234462
H	18.091063	14.764487	8.608884
H	16.967848	18.198957	9.176809
H	15.572817	19.327647	7.477869
H	13.700409	18.114054	6.416309
H	12.711423	14.616999	7.338474
H	11.590410	17.291512	6.345558
H	10.729470	15.753105	6.460533
H	11.269819	16.580206	7.933398
H	14.150578	14.671659	5.289808
H	12.422213	14.689112	4.877169
H	13.379700	16.178879	4.787711
H	12.970239	10.791467	7.166279
H	11.905688	8.545242	7.511762
H	11.190131	9.448025	6.163250
H	12.297637	8.109787	5.842401
H	13.829159	9.699829	4.435410
H	12.633879	10.979297	4.718079
H	14.344185	11.242135	5.122464
H	14.498389	7.639829	5.830289
H	16.584799	6.653579	6.710785
H	17.836204	7.809516	8.499142
H	16.695154	11.000195	10.103117
H	18.215278	8.395052	10.652014
H	18.107022	9.804252	11.712289
H	16.659996	8.837658	11.369609

H	18.393189	11.388959	8.300153
H	19.151335	11.264297	9.902498
H	19.226100	9.904603	8.767472
H	25.052859	15.380991	11.854453
H	26.178647	14.140308	12.336571
H	23.356081	13.616773	11.240234
H	24.496216	12.358161	11.672737
H	23.399835	14.473184	13.591741
H	24.536964	13.206816	14.029509
H	22.837163	11.461546	13.402561
H	21.699684	12.726729	12.961831
H	22.870408	12.315268	15.763801
H	21.735259	13.579867	15.323741
H	21.155148	10.558713	15.170384
H	20.537647	11.666532	16.406002
H	20.011809	11.832609	14.723333
H	27.273241	11.754093	5.855843
H	28.779878	11.697151	6.754144
H	28.617689	12.808749	5.375871
H	28.255021	14.892758	5.791337
H	26.858374	17.847998	6.449929
H	28.256835	17.049218	5.699541
H	28.417219	17.894246	7.254843
H	25.032230	12.601612	6.954721
H	25.188151	10.567538	5.477987
H	23.600406	11.356921	5.415243
H	23.808011	9.861197	6.329715
H	22.909903	11.018075	8.491057
H	22.617514	12.455008	7.493314
H	23.577083	12.576141	8.983511
H	24.645676	9.114895	8.264487
H	26.302562	8.005829	9.723261
H	28.301259	9.245856	10.481644
H	29.045406	12.785738	9.498882
H	30.403322	10.084643	10.003725
H	31.200768	11.657935	9.869791
H	30.388678	10.950377	8.461272
H	28.081170	12.524810	11.792729
H	29.856581	12.554544	11.828505
H	28.985918	11.021584	12.006082
H	28.929268	16.432176	9.639655
H	29.982023	18.623128	9.030306
H	30.946869	17.707193	10.202586
H	29.987501	19.092687	10.735682
H	28.773507	17.562752	12.483506
H	29.815122	16.233065	11.944939
H	28.048433	16.043469	11.948822
H	27.849077	19.597755	11.332414
H	25.661136	20.678320	10.942206
H	24.025370	19.632115	9.415274
H	24.710894	16.506048	7.466763
H	23.118256	19.125272	7.437491
H	22.994552	17.791170	6.286539
H	24.482737	18.754406	6.374071
H	23.452615	15.983743	9.564339
H	22.356065	16.220109	8.185268
H	22.554045	17.494932	9.403380
H	18.632627	11.183372	2.895298
H	17.730371	12.386369	3.800511
H	17.853651	13.964805	1.829644

```
 H    16.813582    11.184875     1.067289
 H    15.911228    12.381265     1.986259
 H    16.032581    13.968807     0.041594
 H    16.938105    12.776779    -0.878145
 H    14.090502    12.381619     0.184098
 H    14.995217    11.191778    -0.737472
 H    14.176440    13.973991    -1.774502
 H    13.417342    12.439479    -2.226537
 H    15.089048    12.776353    -2.702849
 H    16.596135    13.113715     6.256019
 H    18.253830    14.716118     5.263582
 H    18.097354    17.137525     5.665316
 H    19.725377    18.693917     4.608072
 H    21.517142    17.795035     3.139285
 H    21.682930    15.367112     2.730502
 H    18.756606    12.765236     0.919115
  18
[hexene] scf done:  -235.758330 / Energies= -235.583836 / Enthalpies= -235.582891
/ Free Energies= -235.623758
 C    21.277124    13.336836     5.541683
 C    20.985690    13.275225     4.071232
 C    22.047551    13.986662     3.220210
 C    21.775655    13.896538     1.718092
 C    22.833305    14.604485     0.873881
 C    20.492300    13.900249     6.460605
 H    20.763682    13.918784     7.513228
 H    19.543202    14.362729     6.194081
 H    22.220988    12.887179     5.857885
 H    20.931358    12.221713     3.754490
 H    19.997568    13.711596     3.871689
 H    22.101549    15.041617     3.523370
 H    23.036469    13.556352     3.437517
 H    20.786686    14.325938     1.503089
 H    21.717576    12.838933     1.423038
 H    22.892368    15.670786     1.124612
 H    22.610019    14.526365    -0.196352
 H    23.827670    14.171388     1.038678
  20
[Hexane] scf done:  -236.997222 / Energies= -236.798164 / Enthalpies= -236.797219
/ Free Energies= -236.838809
 C     4.839661    -3.514738     2.443914
 C     6.083296    -2.727327     2.032233
 C     7.112334    -2.534420     3.151929
 C     7.756976    -3.826377     3.658916
 C     8.839421    -3.586721     4.713141
 C     9.481855    -4.876147     5.220441
 H     5.774080    -1.738717     1.666165
 H     5.084471    -4.543049     2.732562
 H     4.337104    -3.041493     3.297087
 H     6.566820    -3.227445     1.180529
 H     7.905003    -1.863189     2.790582
 H     6.635637    -2.012320     3.995545
 H     6.988814    -4.489018     4.081804
 H     8.194294    -4.371651     2.808419
 H     8.404350    -3.036009     5.559489
 H     9.615193    -2.930834     4.292494
 H     8.736075    -5.536542     5.680081
 H    10.253527    -4.672427     5.971787
 H     9.953971    -5.432690     4.401232
 H     4.116958    -3.568666     1.621549
```

15
[PhSiH3] scf done: -522.819422 / Energies= -522.697650 / Enthalpies= -522.696705 / Free Energies= -522.734125

```
Si    16.079044     7.804851    -2.458877
 C    17.724407     8.451838    -1.836846
 C    18.065648     9.807240    -1.974905
 C    19.276498    10.296922    -1.488297
 C    20.172620     9.436473    -0.855112
 C    19.854421     8.086528    -0.712461
 C    18.642426     7.600610    -1.200508
 H    15.643165     8.578150    -3.652204
 H    16.201519     6.368521    -2.825037
 H    15.008698     7.921361    -1.430351
 H    17.381214    10.491520    -2.473226
 H    19.522583    11.349374    -1.606704
 H    21.118732     9.816360    -0.477372
 H    20.552303     7.410559    -0.224331
 H    18.413253     6.542503    -1.087500
```

33
[Hexyl-phenyl-silane] scf done: -758.628462 / Energies= -758.324147 / Enthalpies= -758.323203 / Free Energies= -758.384802

```
 H    20.311970    12.278829     5.023428
Si    20.004590    12.976458     3.741727
 C    19.959392    14.828045     4.057799
 C    19.454049    15.342779     5.263829
 C    19.382924    16.716300     5.489258
 C    19.819576    17.607049     4.508892
 C    20.328531    17.118611     3.306416
 C    20.397937    15.743421     3.086398
 H    21.102673    12.690722     2.773701
 C    18.374868    12.311150     3.054630
 C    17.968616    12.883183     1.689271
 C    16.639682    12.324177     1.175207
 C    16.228645    12.878677    -0.189839
 C    14.899907    12.318489    -0.699571
 C    14.496924    12.872886    -2.064613
 H    18.459117    11.215703     2.992121
 H    17.582945    12.508568     3.792251
 H    17.898848    13.978320     1.753355
 H    16.707802    11.227433     1.117997
 H    15.849866    12.540715     1.909981
 H    16.161734    13.975563    -0.132965
 H    17.018876    12.661546    -0.924313
 H    14.110138    12.537844     0.033154
 H    14.966505    11.222410    -0.753803
 H    14.389144    13.964200    -2.033208
 H    13.541890    12.454236    -2.402416
 H    15.250860    12.638154    -2.826253
 H    19.116882    14.662146     6.044127
 H    18.991069    17.092409     6.431362
 H    19.767629    18.678936     4.683993
 H    20.675943    17.808863     2.541308
 H    20.807165    15.378738     2.145892
 H    18.756731    12.671989     0.951676
```

2
[H2] scf done: -1.174562 / Energies= -1.162071 / Enthalpies= -1.161127 / Free Energies= -1.175919

```
 H     0.671429     0.476999     1.359304
 H     0.857901    -0.239179     1.293596
```

148

[A1] scf done: -2879.136412 / Energies= -2877.769142 / Enthalpies= -2877.768198 / Free Energies= -2877.972043

Mg	-3.730446	-0.333874	-0.007987
N	-5.278958	-1.500333	-0.663811
C	-4.930874	-2.632736	-1.467475
C	-4.634631	-3.867721	-0.845467
C	-4.211551	-4.934652	-1.643726
C	-4.078784	-4.799804	-3.020769
C	-4.366991	-3.579389	-3.620210
C	-4.793504	-2.481619	-2.866775
N	-4.944935	0.977149	0.988530
C	-6.271770	0.861409	1.031786
C	-7.001152	-0.165236	0.400556
C	-6.557181	-1.249506	-0.381310
C	-7.627109	-2.170579	-0.924391
C	-4.725406	-4.051092	0.662731
C	-5.588284	-5.256417	1.061381
C	-5.058178	-1.153624	-3.561350
C	-6.048440	-1.283585	-4.727002
C	-4.296747	2.063249	1.659127
C	-3.823597	1.886044	2.979751
C	-3.112460	2.928422	3.581971
C	-2.867700	4.119998	2.909682
C	-3.333813	4.280952	1.610144
C	-4.048586	3.268820	0.962739
C	-4.037087	0.586946	3.743545
C	-4.693980	0.809930	5.112841
C	-4.501872	3.475444	-0.475582
C	-5.313633	4.765299	-0.660670
C	-2.714711	-0.181491	3.896056
C	-3.300991	3.451931	-1.434810
C	-7.077083	1.886761	1.798368
C	-3.323341	-4.161475	1.282870
C	-3.743031	-0.515066	-4.036161
C	1.614102	4.635456	-0.140003
C	2.572382	3.751872	0.670395
C	1.929595	3.336414	2.003352
C	3.922815	4.412395	0.908209
C	5.138887	3.761066	0.596853
C	6.378761	4.378024	0.884086
C	6.375304	5.649282	1.465848
C	5.186475	6.304502	1.764485
C	3.974238	5.682853	1.489522
N	5.114514	2.427747	0.076674
Mg	5.094834	0.910273	1.451838
H	5.138787	0.892973	3.173858
C	7.704452	3.678677	0.619744
C	8.676078	4.537870	-0.200918
C	5.099516	2.227929	-1.241115
C	5.109858	3.424125	-2.167075
C	5.073816	0.959874	-1.853160
C	5.054170	-0.326621	-1.278502
C	5.012661	-1.494249	-2.238974
N	5.065263	-0.564563	0.031996
C	5.041858	-1.911879	0.516444
C	3.802969	-2.537704	0.787859
C	3.811070	-3.821178	1.342337
C	5.001563	-4.479338	1.627835
C	6.212645	-3.849956	1.365381
C	6.259854	-2.567207	0.811548

C	2.475125	-1.836713	0.538863
C	1.834010	-1.403373	1.866989
C	7.608501	-1.900099	0.581262
C	8.556472	-2.760000	-0.266035
C	8.265731	-1.524987	1.919119
C	1.493892	-2.687096	-0.279690
C	8.355305	3.234764	1.939621
H	-6.749013	1.939410	2.842086
H	-6.931499	2.887663	1.376961
H	-8.143667	1.652941	1.777920
H	-8.075237	-0.110398	0.535491
H	-8.622763	-1.860031	-0.600471
H	-7.605458	-2.185824	-2.019819
H	-7.457707	-3.201957	-0.596834
H	-4.717647	-0.038135	3.154329
H	-5.642115	1.351952	5.022603
H	-4.898913	-0.152279	5.597407
H	-4.046217	1.384151	5.785553
H	-1.994555	0.389756	4.494194
H	-2.880123	-1.143044	4.397372
H	-2.246655	-0.378116	2.924181
H	-2.738654	2.801356	4.595236
H	-2.311946	4.918229	3.395189
H	-3.133552	5.210920	1.083242
H	-5.155637	2.638047	-0.743592
H	-4.704825	5.658448	-0.477295
H	-5.691891	4.832436	-1.687823
H	-6.172481	4.804912	0.018871
H	-2.727903	2.521646	-1.342579
H	-3.633243	3.548207	-2.475877
H	-2.614519	4.280587	-1.222773
H	-5.508427	-0.477164	-2.826055
H	-6.992621	-1.737993	-4.406202
H	-6.273670	-0.295799	-5.146684
H	-5.643325	-1.899444	-5.538500
H	-3.250660	-1.143624	-4.788140
H	-3.929174	0.467015	-4.488226
H	-3.035563	-0.383836	-3.208716
H	-4.253311	-3.473697	-4.696590
H	-3.747119	-5.641346	-3.623838
H	-3.976349	-5.887966	-1.176139
H	-5.202742	-3.157186	1.079443
H	-5.143436	-6.202369	0.730779
H	-5.690402	-5.306226	2.152075
H	-6.593785	-5.194409	0.630088
H	-2.702175	-3.292112	1.036616
H	-3.386752	-4.239379	2.375268
H	-2.800299	-5.051046	0.911351
H	4.094803	-2.075941	-2.097773
H	5.846453	-2.180289	-2.055418
H	5.055171	-1.158656	-3.277400
H	5.062465	0.975839	-2.936973
H	4.240429	4.064536	-1.982313
H	5.101341	3.117597	-3.215227
H	5.995146	4.044633	-1.990025
H	2.678183	-0.928765	-0.039944
H	1.935587	-3.013603	-1.228393
H	0.589699	-2.109648	-0.504392
H	1.181086	-3.585095	0.266481
H	1.595997	-2.275686	2.488125

H	0.903450	-0.853178	1.684032
H	2.505788	-0.763234	2.451997
H	2.865097	-4.311497	1.559651
H	4.985559	-5.477110	2.059539
H	7.142417	-4.362584	1.601320
H	7.430419	-0.971414	0.027569
H	8.835879	-3.686459	0.249542
H	9.482162	-2.210812	-0.476619
H	8.102444	-3.037077	-1.224152
H	7.614676	-0.881259	2.522421
H	9.212594	-0.996468	1.752419
H	8.480321	-2.420499	2.514852
H	7.494571	2.776183	0.034776
H	8.225890	4.869316	-1.143498
H	9.579884	3.964907	-0.440885
H	8.992883	5.432278	0.348486
H	8.601016	4.101020	2.565929
H	9.283795	2.682347	1.748818
H	7.686570	2.590955	2.523142
H	7.322368	6.132371	1.694845
H	5.204883	7.292908	2.217059
H	3.045766	6.191938	1.737456
H	2.743567	2.840099	0.087390
H	1.333739	5.542264	0.409210
H	0.690604	4.087325	-0.360669
H	2.059090	4.947811	-1.091657
H	2.585965	2.672022	2.578124
H	0.978874	2.817497	1.831962
H	1.727295	4.213897	2.629666
H	-2.022033	-0.417108	-0.223862

166

[A2] scf done: -3114.888923 / Energies= -3113.343999 / Enthalpies= -3113.343054 / Free Energies= -3113.569419

Mg	-4.792067	-0.223346	-1.336274
N	-6.306413	-1.613014	-1.014931
C	-6.279348	-2.850474	-1.726760
C	-5.746229	-3.998314	-1.085936
C	-5.668474	-5.194050	-1.805323
C	-6.085843	-5.269637	-3.130125
C	-6.593844	-4.136020	-3.752299
C	-6.705724	-2.916037	-3.075001
N	-5.238496	0.855093	0.393628
C	-6.491079	0.849330	0.844925
C	-7.472738	-0.090947	0.471657
C	-7.389357	-1.243969	-0.333938
C	-8.667152	-2.054459	-0.428611
C	-5.297858	-3.957964	0.369208
C	-6.398441	-4.471064	1.312174
C	-7.295225	-1.718636	-3.808751
C	-8.726972	-1.999556	-4.292147
C	-4.277391	1.764928	0.931091
C	-3.491263	1.360467	2.042298
C	-2.515594	2.236249	2.528027
C	-2.294789	3.475215	1.936345
C	-3.058625	3.853539	0.839811
C	-4.056157	3.021784	0.319105
C	-3.705622	0.015822	2.726778
C	-4.602616	0.148640	3.968563
C	-4.874885	3.514751	-0.866168
C	-5.664884	4.785241	-0.513388

C	-2.393389	-0.679724	3.115774
C	-4.001567	3.754788	-2.105877
C	-6.972555	1.933107	1.790489
C	-3.999540	-4.735053	0.623385
C	-6.413604	-1.276600	-4.985865
C	-2.379085	-1.174164	-0.524149
C	-1.981991	-1.327035	-1.797825
C	-1.724155	-2.631358	-2.485286
C	-2.436196	-2.775503	-3.836853
C	-2.057164	-4.066170	-4.564100
C	-2.752578	-4.212791	-5.915460
C	2.320410	4.807305	0.275397
C	3.292816	3.927289	1.072794
C	2.651471	3.470022	2.392788
C	4.628056	4.609790	1.333423
C	5.859116	3.985342	1.025858
C	7.084483	4.620747	1.333477
C	7.051589	5.883697	1.932198
C	5.848043	6.512756	2.227677
C	4.650118	5.872969	1.932156
N	5.865608	2.658700	0.487276
Mg	5.881184	1.123932	1.841755
H	5.886502	1.070544	3.563095
C	8.425778	3.950720	1.071649
C	9.384142	4.836674	0.263871
C	5.859967	2.476788	-0.832799
C	5.846949	3.684913	-1.742807
C	5.865364	1.216049	-1.461740
C	5.879985	-0.077609	-0.904760
C	5.887352	-1.232799	-1.881191
N	5.890113	-0.333486	0.403055
C	5.906459	-1.686981	0.869126
C	4.686542	-2.338983	1.163643
C	4.729695	-3.629043	1.700541
C	5.938163	-4.269641	1.947402
C	7.130961	-3.615048	1.663468
C	7.142453	-2.324612	1.125760
C	3.341037	-1.658976	0.954363
C	2.706214	-1.285219	2.303348
C	8.472722	-1.627870	0.877513
C	9.434786	-2.469887	0.028273
C	9.131711	-1.229468	2.207866
C	2.372906	-2.505486	0.116448
C	9.078026	3.509083	2.391616
H	-2.463418	-5.144775	-6.414693
H	-6.157273	2.384294	2.358812
H	-7.449136	2.732078	1.208350
H	-7.722356	1.544478	2.485302
H	-8.447885	0.076676	0.917982
H	-9.248069	-1.979980	0.494594
H	-9.289159	-1.659796	-1.241769
H	-8.476201	-3.106870	-0.647693
H	-4.230733	-0.630073	2.012742
H	-5.590493	0.544909	3.717998
H	-4.744519	-0.828695	4.446484
H	-4.147127	0.821324	4.705900
H	-1.887231	-0.166615	3.942045
H	-2.598026	-1.702683	3.453744
H	-1.690706	-0.732041	2.277530
H	-1.916131	1.943419	3.386159

H	-1.531318	4.141604	2.330062
H	-2.884977	4.822930	0.378190
H	-5.598905	2.733610	-1.124200
H	-4.993866	5.628855	-0.310783
H	-6.314945	5.073642	-1.348222
H	-6.291425	4.642831	0.373575
H	-3.507453	2.831575	-2.423457
H	-4.619724	4.103940	-2.941929
H	-3.235529	4.516741	-1.915373
H	-7.339239	-0.881535	-3.103093
H	-9.375924	-2.337324	-3.476994
H	-9.166332	-1.093157	-4.725761
H	-8.741701	-2.774951	-5.067538
H	-6.306677	-2.078382	-5.727120
H	-6.863132	-0.412841	-5.490989
H	-5.417463	-0.979139	-4.644387
H	-6.922802	-4.197974	-4.787243
H	-6.017379	-6.209065	-3.673195
H	-5.275119	-6.083820	-1.320314
H	-5.115628	-2.906253	0.620599
H	-6.654253	-5.512213	1.079225
H	-6.061526	-4.431665	2.355521
H	-7.312357	-3.875479	1.233426
H	-3.206780	-4.443957	-0.073924
H	-3.642770	-4.548618	1.643425
H	-4.142843	-5.817904	0.527550
H	5.033545	-1.896073	-1.705379
H	6.786825	-1.844508	-1.749831
H	5.852143	-0.882206	-2.914910
H	5.858586	1.246484	-2.545259
H	4.964141	4.304900	-1.552029
H	5.847441	3.392550	-2.794975
H	6.718796	4.321119	-1.555150
H	3.519352	-0.728911	0.403135
H	2.809933	-2.779925	-0.850452
H	1.448658	-1.947083	-0.074659
H	2.094405	-3.433258	0.630175
H	2.490027	-2.182169	2.896327
H	1.763508	-0.745037	2.152648
H	3.372461	-0.652912	2.902305
H	3.798399	-4.138848	1.936303
H	5.950514	-5.272816	2.366241
H	8.074978	-4.113990	1.869865
H	8.268353	-0.706559	0.320616
H	9.747370	-3.381927	0.550245
H	10.341669	-1.896679	-0.198858
H	8.978435	-2.770925	-0.921498
H	8.470638	-0.598762	2.814161
H	10.064001	-0.679054	2.030752
H	9.372231	-2.116565	2.806219
H	8.238094	3.049279	0.477464
H	8.931907	5.167880	-0.677765
H	10.300236	4.283991	0.023142
H	9.680486	5.732045	0.823013
H	9.301512	4.374659	3.026994
H	10.019182	2.978358	2.201787
H	8.419881	2.846162	2.965869
H	7.987126	6.381057	2.177139
H	5.843646	7.495051	2.693522
H	3.710305	6.362225	2.176968

H	3.486956	3.031398	0.472389
H	2.017925	5.696445	0.841326
H	1.409162	4.246018	0.036731
H	2.763630	5.149220	-0.666735
H	3.318895	2.809709	2.959417
H	1.714758	2.931666	2.203021
H	2.423562	4.329289	3.035368
H	-1.805755	-0.432906	-2.397116
H	-0.637514	-2.704364	-2.654605
H	-3.522346	-2.746875	-3.680927
H	-2.193723	-1.910210	-4.470035
H	-0.966711	-4.097706	-4.704878
H	-2.310100	-4.925970	-3.927937
H	-2.497361	-3.382385	-6.585223
H	-3.842081	-4.220124	-5.795250
H	-4.347025	0.475484	-2.866391
H	-1.991658	-3.465864	-1.825059
H	-2.441051	-0.186334	-0.065926
H	-2.556770	-2.028134	0.126476

166

[TS-A2A3] scf done: -3114.870769 / Energies= -3113.327045 / Enthalpies= -3113.326101 / Free Energies=-3113.547348

Mg	15.845201	12.966027	6.912874
N	14.301959	11.606120	7.100031
C	14.458243	10.332937	6.471837
C	15.158897	9.310352	7.160468
C	15.362370	8.086958	6.515049
C	14.907798	7.867807	5.219236
C	14.237734	8.882722	4.548305
C	13.997086	10.122439	5.150474
N	15.017535	14.358658	8.189705
C	13.756392	14.258828	8.602746
C	12.910640	13.162115	8.342602
C	13.154684	11.937834	7.687326
C	11.979692	10.981254	7.648214
C	15.654745	9.512979	8.586303
C	14.607680	9.058172	9.615818
C	13.272259	11.200042	4.355139
C	11.930707	10.707424	3.791711
C	15.827857	15.469421	8.578259
C	16.493576	15.461850	9.829436
C	17.363132	16.514501	10.131977
C	17.574973	17.560680	9.241632
C	16.905821	17.566100	8.023773
C	16.030390	16.535296	7.666824
C	16.295523	14.353946	10.856406
C	15.819519	14.904472	12.210223
C	15.288508	16.620676	6.339231
C	14.230164	17.735428	6.368820
C	17.571060	13.521095	11.051776
C	16.232452	16.814455	5.145446
C	13.150540	15.361727	9.445404
C	16.996253	8.825265	8.867751
C	14.159049	11.744882	3.226037
C	18.032361	12.375100	7.144502
C	18.056427	12.912217	5.852907
C	18.324286	12.064685	4.628026
C	18.249456	12.797148	3.290287
C	18.584639	11.900923	2.097527
C	18.522264	12.637747	0.761249

C	22.114824	18.003704	7.882605
C	23.082251	17.123067	8.685466
C	22.446167	16.694641	10.017607
C	24.427872	17.793118	8.924635
C	25.648858	17.153062	8.609268
C	26.884011	17.778827	8.897618
C	26.870756	19.047425	9.485005
C	25.677051	19.691527	9.788023
C	24.469592	19.061344	9.511567
N	25.635821	15.821346	8.083831
Mg	25.645131	14.299660	9.453793
H	25.664894	14.270058	11.175663
C	28.215378	17.092190	8.628306
C	29.176556	17.962064	7.806574
C	25.618753	15.626241	6.765586
C	25.610830	16.825573	5.843818
C	25.608310	14.359616	6.149207
C	25.617507	13.071251	6.719262
C	25.603119	11.906088	5.754878
N	25.637374	12.828832	8.029222
C	25.647851	11.479650	8.508574
C	24.425374	10.831658	8.801242
C	24.464520	9.546494	9.350114
C	25.670915	8.907062	9.609995
C	26.866031	9.557852	9.327386
C	26.881776	10.843484	8.778324
C	23.081548	11.510680	8.578326
C	22.448081	11.910978	9.920379
C	28.214532	11.535244	8.529311
C	29.170592	10.690028	7.676396
C	28.878199	11.928913	9.858696
C	22.110177	10.651767	7.756878
C	28.874133	16.651965	9.945524
H	18.760718	11.971502	-0.075802
H	13.530957	16.345688	9.160193
H	12.060812	15.361640	9.360994
H	13.401446	15.213235	10.502284
H	11.908880	13.262088	8.746747
H	11.306601	11.155458	8.491632
H	11.403784	11.134944	6.727569
H	12.299787	9.936722	7.656482
H	15.520145	13.679363	10.478314
H	14.930605	15.535777	12.105759
H	15.574323	14.081230	12.891980
H	16.596013	15.509375	12.693367
H	18.398720	14.140557	11.418012
H	17.401107	12.724117	11.786319
H	17.890900	13.053232	10.116001
H	17.882232	16.515316	11.087811
H	18.252355	18.371025	9.499262
H	17.060069	18.393241	7.334758
H	14.762800	15.671223	6.188060
H	14.695332	18.718107	6.514897
H	13.676416	17.764090	5.422362
H	13.506233	17.587520	7.177288
H	16.962279	16.001601	5.082129
H	15.659905	16.820939	4.210242
H	16.777389	17.764362	5.204891
H	13.062691	12.035607	5.031431
H	11.292255	10.278784	4.572064

H	11.387756	11.537986	3.324959
H	12.071996	9.937985	3.023372
H	14.434434	10.950126	2.521709
H	13.630093	12.524756	2.663783
H	15.078212	12.184046	3.628218
H	13.891730	8.710952	3.531703
H	15.078906	6.909881	4.734178
H	15.888938	7.290258	7.033398
H	15.799518	10.590936	8.729418
H	14.379384	7.992204	9.492558
H	14.981227	9.208097	10.636408
H	13.672732	9.617592	9.517076
H	17.745110	9.062610	8.104361
H	17.386236	9.150728	9.839131
H	16.897254	7.733984	8.911439
H	24.723315	11.275284	5.923039
H	26.477886	11.264688	5.907828
H	25.595789	12.246510	4.717194
H	25.592984	14.379228	5.065502
H	24.737545	17.458064	6.037272
H	25.597550	16.522878	4.794628
H	26.492095	17.453000	6.016588
H	23.261800	12.430413	8.010527
H	22.549659	10.349248	6.799392
H	21.190991	11.211830	7.547515
H	21.822260	9.739054	8.292132
H	22.232755	11.025428	10.530604
H	21.505122	12.447457	9.759503
H	23.114666	12.554709	10.506896
H	23.531341	9.039547	9.584210
H	25.679698	7.907558	10.037671
H	27.808415	9.059852	9.543484
H	28.012917	12.458040	7.973814
H	29.476634	9.773712	8.194806
H	30.081396	11.257743	7.450938
H	28.711569	10.396073	6.725716
H	28.221020	12.561702	10.467002
H	29.812415	12.475619	9.680115
H	29.116655	11.040159	10.455422
H	28.012489	16.188953	8.041939
H	28.720289	18.291610	6.866289
H	30.084662	17.397843	7.562336
H	29.487059	18.857987	8.357056
H	29.113627	17.519249	10.572729
H	29.807128	16.108590	9.751139
H	28.213287	16.001065	10.530299
H	27.814044	19.537272	9.715041
H	25.687855	20.677926	10.245050
H	23.537563	19.562163	9.762781
H	23.260200	16.215875	8.097130
H	21.835124	18.908899	8.434675
H	21.190727	17.454216	7.667086
H	22.552255	18.319563	6.928678
H	23.108484	16.031410	10.586759
H	21.498720	16.169911	9.844438
H	22.238684	17.566515	10.649977
H	18.376064	13.950655	5.761959
H	19.344178	11.669315	4.761828
H	17.245434	13.222075	3.166363
H	18.944887	13.650281	3.307475

H	19.587824	11.470814	2.231982
H	17.889303	11.049528	2.078651
H	19.233402	13.472828	0.734814
H	17.521704	13.050955	0.584191
H	16.450926	13.453825	5.257718
H	17.662912	11.188893	4.627249
H	18.363141	12.989639	7.978547
H	18.051649	11.295409	7.280899

166

[A3] scf done: -3114.932955 / Energies= -3113.383419 / Enthalpies= -3113.382475 / Free Energies= -3113.608059

Mg	-4.621925	-0.627571	-0.319000
N	-6.118344	-2.029243	-0.127303
C	-5.851113	-3.317565	-0.690210
C	-5.202370	-4.302110	0.091145
C	-4.862472	-5.517581	-0.510081
C	-5.150304	-5.769601	-1.846428
C	-5.788114	-4.794807	-2.604710
C	-6.148179	-3.562522	-2.050942
N	-5.701606	0.836328	0.646205
C	-6.917034	0.640567	1.158120
C	-7.633016	-0.568760	1.074359
C	-7.279541	-1.796709	0.481182
C	-8.308211	-2.903156	0.560662
C	-4.837756	-4.059213	1.548764
C	-5.357197	-5.163950	2.479488
C	-6.813396	-2.512032	-2.928902
C	-8.060096	-3.045970	-3.647734
C	-5.071992	2.116227	0.769293
C	-4.179790	2.357072	1.840413
C	-3.492897	3.574385	1.880870
C	-3.674286	4.540575	0.898537
C	-4.559615	4.296932	-0.145015
C	-5.270455	3.096253	-0.232176
C	-3.936919	1.327011	2.934557
C	-4.204111	1.891623	4.337369
C	-6.212123	2.870457	-1.407049
C	-7.256578	3.987524	-1.546622
C	-2.516010	0.749048	2.847948
C	-5.431047	2.702804	-2.719309
C	-7.603465	1.776649	1.884157
C	-3.319400	-3.885941	1.712087
C	-5.813265	-1.932331	-3.941620
C	-2.689894	-0.844182	-1.172229
C	-2.188278	0.195583	-2.185294
C	-0.783713	-0.080644	-2.737889
C	-0.299103	0.952971	-3.755891
C	1.098106	0.667161	-4.309536
C	1.566859	1.703143	-5.329616
C	1.947850	4.126280	-0.071690
C	2.923748	3.240388	0.714866
C	2.309008	2.818079	2.058869
C	4.278151	3.900939	0.928541
C	5.488383	3.245284	0.603378
C	6.733158	3.863649	0.864295
C	6.740369	5.139377	1.436436
C	5.557345	5.797509	1.750755
C	4.340059	5.175276	1.499897
N	5.455021	1.906302	0.097822
Mg	5.478791	0.404900	1.489709

H	5.530945	0.403521	3.211446
C	8.054017	3.162967	0.580170
C	9.003648	4.012972	-0.275109
C	5.403761	1.691175	-1.216521
C	5.383008	2.876235	-2.156219
C	5.368057	0.415320	-1.812696
C	5.384829	-0.864658	-1.223619
C	5.338087	-2.043603	-2.169880
N	5.436639	-1.087568	0.089033
C	5.453166	-2.429350	0.588674
C	4.234983	-3.076153	0.901554
C	4.282335	-4.352548	1.469741
C	5.492589	-4.985195	1.728621
C	6.683381	-4.336050	1.425281
C	6.690892	-3.058620	0.856988
C	2.887653	-2.404411	0.678613
C	2.272258	-1.967854	2.017791
C	8.019910	-2.369449	0.582538
C	8.959857	-3.221094	-0.281830
C	8.706475	-1.968521	1.898010
C	1.905720	-3.285778	-0.105614
C	8.736563	2.739615	1.890755
H	2.568257	1.470415	-5.711074
H	-7.762475	2.628471	1.213942
H	-8.570369	1.466276	2.286080
H	-6.981808	2.142160	2.708641
H	-8.611841	-0.552031	1.539691
H	-9.211828	-2.571552	1.076720
H	-8.584590	-3.248693	-0.441566
H	-7.904103	-3.774041	1.088373
H	-4.640948	0.502327	2.776279
H	-5.216079	2.303371	4.421664
H	-4.095560	1.103514	5.092107
H	-3.498833	2.690986	4.593508
H	-1.762551	1.528017	3.016055
H	-2.366915	-0.029586	3.606097
H	-2.319878	0.309647	1.863174
H	-2.801240	3.767310	2.697759
H	-3.129367	5.480070	0.946597
H	-4.699961	5.054807	-0.912341
H	-6.754623	1.936272	-1.224905
H	-6.791745	4.948382	-1.796981
H	-7.964442	3.746775	-2.348800
H	-7.828244	4.127790	-0.622401
H	-4.701347	1.888195	-2.655115
H	-6.113282	2.486972	-3.550877
H	-4.877277	3.616134	-2.968316
H	-7.138057	-1.692263	-2.278502
H	-8.785004	-3.466683	-2.941778
H	-8.555229	-2.238622	-4.200596
H	-7.807253	-3.830327	-4.370689
H	-5.457970	-2.708004	-4.630761
H	-6.280296	-1.139418	-4.538752
H	-4.932125	-1.512104	-3.442665
H	-6.008671	-4.992513	-3.651190
H	-4.876635	-6.721063	-2.295563
H	-4.358738	-6.279921	0.079785
H	-5.312550	-3.121843	1.859085
H	-4.875458	-6.127444	2.275659
H	-5.148781	-4.908736	3.525451

H	-6.438785	-5.305745	2.374971
H	-2.935488	-3.076064	1.080938
H	-3.064300	-3.658974	2.754646
H	-2.786908	-4.801505	1.427168
H	4.443807	-2.650011	-1.987881
H	6.198166	-2.703254	-2.012199
H	5.331148	-1.718663	-3.212429
H	5.325030	0.418612	-2.895719
H	4.519031	3.518251	-1.952629
H	5.342518	2.557659	-3.199889
H	6.272829	3.499176	-2.013366
H	3.059133	-1.500333	0.083834
H	2.336379	-3.625548	-1.054359
H	0.990295	-2.726765	-0.331598
H	1.613296	-4.176206	0.463643
H	2.067037	-2.836691	2.655019
H	1.326356	-1.436830	1.855358
H	2.945722	-1.307404	2.577277
H	3.352525	-4.858162	1.719304
H	5.507624	-5.978180	2.171106
H	7.629055	-4.828731	1.639318
H	7.809568	-1.449974	0.024722
H	9.270758	-4.136315	0.235695
H	9.868767	-2.657306	-0.524287
H	8.484520	-3.517252	-1.223727
H	8.059876	-1.332056	2.513751
H	9.637487	-1.422967	1.700119
H	8.955620	-2.853729	2.495714
H	7.832863	2.251881	0.012834
H	8.531333	4.326730	-1.212841
H	9.904913	3.440454	-0.525405
H	9.327209	4.917724	0.252906
H	8.996446	3.615312	2.497875
H	9.660850	2.185101	1.686357
H	8.082788	2.104325	2.500104
H	7.691619	5.623610	1.644799
H	5.583998	6.789044	2.195912
H	3.416401	5.687476	1.758913
H	3.084685	2.331217	0.124995
H	1.674439	5.028581	0.488194
H	1.021575	3.578061	-0.280507
H	2.374440	4.446593	-1.028963
H	2.978825	2.155410	2.619878
H	1.358233	2.293780	1.902837
H	2.113536	3.692718	2.691256
H	-2.191943	1.200127	-1.734176
H	-0.073911	-0.127006	-1.897539
H	-1.014026	1.002566	-4.591810
H	-0.306927	1.951943	-3.293243
H	1.815739	0.620824	-3.477603
H	1.105437	-0.330761	-4.771411
H	1.603909	2.706474	-4.886945
H	0.886886	1.748814	-6.189532
H	-2.882188	0.256206	-3.039506
H	-0.773109	-1.080603	-3.198095
H	-1.951391	-0.914848	-0.353942
H	-2.668259	-1.840392	-1.647603

184

[C1] scf done: -3350.685391 / Energies= -3348.956282 / Enthalpies= -3348.955337 / Free Energies= -3349.187176

C	-2.009335	-2.781310	2.892860
C	-2.342128	-1.406046	2.852783
C	-3.695401	-0.997524	2.774601
C	-4.692584	-1.977817	2.751106
C	-4.376616	-3.330746	2.783479
C	-3.043794	-3.720755	2.841605
N	-1.313424	-0.410194	2.945343
C	-0.884651	-0.136632	4.179759
C	-1.404619	-0.928577	5.366182
C	-4.095183	0.471781	2.785771
C	-5.120432	0.814050	1.697394
C	-0.569987	-3.263621	3.012553
C	-0.032151	-3.732753	1.657618
C	0.040508	0.867899	4.513231
C	0.539037	1.937683	3.751601
C	1.333854	2.961082	4.541105
N	0.345934	2.104488	2.440216
C	0.748461	3.377297	1.913116
C	2.082691	3.590892	1.494075
C	2.436782	4.852986	1.006095
C	1.522286	5.898820	0.969128
C	0.220959	5.685564	1.407978
C	-0.194596	4.433939	1.873745
C	3.144949	2.506458	1.603580
C	3.452247	1.880244	0.240773
C	-1.622377	4.270373	2.380522
C	-2.665633	4.688007	1.335795
C	4.444409	3.012041	2.249667
C	-1.849635	5.044813	3.688903
C	-4.623761	0.901662	4.163482
C	-0.396979	-4.376268	4.057841
Mg	-0.426644	0.524021	1.244974
C	3.927060	-0.805516	4.613698
C	4.393086	-1.584939	3.636235
C	5.786863	-2.134273	3.553071
C	5.825125	-3.669319	3.570522
C	7.237556	-4.239845	3.434338
C	7.271496	-5.766562	3.453722
C	-1.687946	1.275961	-0.567629
C	-2.692889	1.056911	-1.726111
C	-3.700490	2.195453	-1.931900
C	-4.699924	1.940701	-3.061757
C	-5.700470	3.080279	-3.262065
C	-6.697857	2.816944	-4.388506
Mg	-0.150360	-0.127328	-1.579645
N	0.723371	0.837138	-3.290976
C	0.278098	0.493951	-4.498913
C	-0.563042	-0.599919	-4.782279
C	-0.862555	-1.741808	-4.019575
N	-0.680368	-1.857973	-2.701044
C	-0.854570	-3.177121	-2.158758
C	-2.145204	-3.674916	-1.854255
C	-2.267648	-4.984363	-1.378557
C	-1.159594	-5.807926	-1.221881
C	0.102809	-5.310205	-1.518100
C	0.282261	-4.001217	-1.976836
C	-3.405891	-2.840407	-2.030668
C	-3.938078	-2.374566	-0.671658
C	1.691820	-3.532918	-2.308649
C	2.639434	-3.673838	-1.109739

C	1.758067	1.833705	-3.247146
C	1.429982	3.205894	-3.129044
C	2.466470	4.144907	-3.095375
C	3.798607	3.762214	-3.190115
C	4.109253	2.415259	-3.328753
C	3.112006	1.435516	-3.366244
C	-0.006792	3.708323	-3.109679
C	-0.275227	4.626497	-1.914030
C	3.526388	-0.012312	-3.590211
C	4.402377	-0.542884	-2.447613
C	0.724107	1.230213	-5.751936
C	4.250064	-0.189135	-4.934926
C	-0.374161	4.444037	-4.409826
C	-1.385160	-2.917457	-4.824207
C	-4.520730	-3.578125	-2.789268
C	2.254097	-4.271226	-3.533365
H	0.747704	-0.472547	0.089355
H	2.386167	2.947346	4.238371
H	0.972842	3.978190	4.367114
H	1.285591	2.751518	5.611845
H	0.308351	0.899762	5.562592
H	-0.975011	-1.935721	5.381290
H	-1.141673	-0.436290	6.304994
H	-2.489928	-1.051998	5.326384
H	2.748000	1.712801	2.245114
H	4.260019	3.545410	3.189248
H	5.105420	2.164877	2.465643
H	4.990450	3.693042	1.585875
H	3.808437	2.631597	-0.471710
H	4.226813	1.110475	0.342060
H	2.565172	1.406893	-0.190758
H	3.456282	5.024611	0.670744
H	1.824232	6.877925	0.604987
H	-0.489324	6.509024	1.390882
H	-1.775763	3.207916	2.598161
H	-2.876170	4.898309	4.046891
H	-1.170028	4.715536	4.481340
H	-1.697165	6.121322	3.542899
H	-2.544655	4.127595	0.404303
H	-3.677721	4.502198	1.715984
H	-2.597687	5.756511	1.098436
H	-3.190785	1.058822	2.588947
H	-5.513934	0.322934	4.439704
H	-3.873964	0.759981	4.948211
H	-4.902142	1.962734	4.154431
H	-5.289642	1.896916	1.665022
H	-4.778863	0.492658	0.708446
H	-6.091217	0.340357	1.886320
H	-5.736050	-1.674796	2.712727
H	-5.166115	-4.078364	2.765177
H	-2.800311	-4.779703	2.866414
H	0.044270	-2.414554	3.329713
H	-0.858409	-5.315828	3.731601
H	0.669067	-4.577920	4.215274
H	-0.838525	-4.111053	5.025340
H	-0.049909	-2.927741	0.916868
H	1.004367	-4.078054	1.755074
H	-0.628708	-4.560338	1.260494
H	-0.767746	-3.806790	-4.662416
H	-1.389351	-2.688176	-5.891833

H	-2.402036	-3.186710	-4.526688
H	-0.844166	-0.673735	-5.827424
H	1.424752	0.604698	-6.317230
H	1.215684	2.180272	-5.547836
H	-0.139968	1.406510	-6.401264
H	-3.139944	-1.950031	-2.610216
H	-4.176143	-3.994834	-3.742078
H	-5.347848	-2.889986	-3.000791
H	-4.931310	-4.405622	-2.198933
H	-4.225812	-3.228806	-0.050610
H	-4.822962	-1.738150	-0.797263
H	-3.186194	-1.810374	-0.111454
H	-3.255659	-5.371094	-1.141845
H	-1.279312	-6.829624	-0.869275
H	0.971447	-5.953535	-1.397098
H	1.635715	-2.469489	-2.563750
H	3.257548	-3.901922	-3.778257
H	1.619830	-4.131034	-4.415391
H	2.332193	-5.349271	-3.346126
H	2.286049	-3.084201	-0.258242
H	3.643421	-3.322680	-1.373168
H	2.730536	-4.717218	-0.785107
H	2.617002	-0.620730	-3.622029
H	5.213377	0.334920	-4.942760
H	3.657178	0.199198	-5.769320
H	4.449975	-1.250733	-5.124561
H	4.711236	-1.575160	-2.651581
H	3.866664	-0.529219	-1.493776
H	5.313059	0.056966	-2.330692
H	5.150513	2.114827	-3.421516
H	4.588243	4.509504	-3.168633
H	2.220427	5.200161	-3.008194
H	-0.663593	2.836259	-3.024556
H	0.261881	5.326344	-4.552437
H	-1.415255	4.787358	-4.368855
H	-0.266426	3.808451	-5.293288
H	0.016842	4.161564	-0.969479
H	-1.340238	4.881078	-1.858461
H	0.279303	5.568345	-1.996922
H	2.900194	-0.447633	4.620762
H	4.561208	-0.494494	5.443057
H	3.719760	-1.872429	2.825809
H	6.262494	-1.783686	2.623537
H	6.390807	-1.739521	4.381422
H	5.370077	-4.027898	4.504519
H	5.196143	-4.057652	2.755748
H	7.866447	-3.848268	4.246690
H	7.686731	-3.874093	2.499766
H	6.860013	-6.160482	4.391149
H	8.294872	-6.146254	3.354106
H	6.679121	-6.186514	2.631396
H	-2.327734	1.408412	0.330085
H	-1.236851	2.274717	-0.700728
H	-3.248952	0.126475	-1.555537
H	-2.174293	0.906497	-2.690269
H	-4.244866	2.361406	-0.990896
H	-3.152047	3.127266	-2.133089
H	-4.153096	1.769822	-4.001570
H	-5.250127	1.009236	-2.858437
H	-5.151960	4.010669	-3.468293

H	-6.245371	3.251840	-2.322606
H	-6.184162	2.674528	-5.347473
H	-7.399620	3.650571	-4.507967
H	-7.286536	1.912042	-4.192697

184

[TS-C1C2] scf done: -3350.636576 / Energies= -3348.909566 / Enthalpies= -3348.908622 / Free Energies= -3349.130180

C	20.220696	16.362324	9.256352
C	19.908199	17.737910	9.388762
C	18.553523	18.152891	9.408736
C	17.547314	17.188040	9.297791
C	17.845774	15.837235	9.172787
C	19.175822	15.438259	9.149536
N	20.950169	18.714366	9.539212
C	21.289408	19.048392	10.784177
C	20.736851	18.294718	11.982185
C	18.142433	19.608284	9.579504
C	17.351547	20.115652	8.365724
C	21.647250	15.833998	9.289337
C	21.988402	15.036988	8.024645
C	22.124376	20.128358	11.144252
C	22.510992	21.281354	10.430577
C	23.071139	22.402036	11.288935
N	22.402617	21.435534	9.110411
C	22.635465	22.745406	8.576283
C	23.946741	23.209603	8.312453
C	24.117124	24.503451	7.808490
C	23.034030	25.337529	7.569535
C	21.748741	24.872527	7.821986
C	21.521533	23.585669	8.319497
C	25.180407	22.350453	8.544263
C	25.714082	21.794739	7.217763
C	20.098352	23.132428	8.616405
C	19.053124	23.770485	7.694178
C	26.299745	23.089804	9.292605
C	19.713004	23.393774	10.082011
C	17.327108	19.825979	10.863708
C	21.913714	14.976588	10.537302
Mg	22.200092	19.605986	8.026117
C	24.215353	18.640635	8.630123
C	24.575049	17.716288	7.662694
C	25.946450	17.726689	7.038923
C	26.977112	17.158639	8.032664
C	28.394601	17.086811	7.462643
C	29.409358	16.537944	8.463919
C	21.207108	20.095310	6.059833
C	20.488892	20.192473	4.690676
C	19.410571	21.279512	4.601526
C	18.679341	21.301678	3.257842
C	17.629813	22.408929	3.148723
C	16.898511	22.414577	1.807735
Mg	22.626682	18.615954	4.789897
N	23.529306	19.597641	3.085468
C	22.982613	19.434881	1.881150
C	21.970051	18.507935	1.558500
C	21.468109	17.395700	2.260684
N	21.669012	17.148030	3.555062
C	21.261328	15.863060	4.045717
C	19.921345	15.617954	4.436688
C	19.581024	14.353442	4.927326

C	20.516459	13.329927	5.012894
C	21.823447	13.570897	4.609402
C	22.219924	14.823142	4.129059
C	18.835806	16.678973	4.337060
C	18.506637	17.260663	5.716089
C	23.656714	15.005426	3.663846
C	24.665688	14.639696	4.759329
C	24.687953	20.437060	3.196607
C	24.579201	21.846378	3.277770
C	25.745575	22.607386	3.423942
C	26.998395	22.015119	3.476370
C	27.100636	20.632513	3.369626
C	25.969698	19.824272	3.223751
C	23.254571	22.589915	3.180508
C	22.912187	23.306255	4.491652
C	26.135869	18.321890	3.031088
C	27.450200	17.770669	3.594625
C	23.439402	20.266179	0.693098
C	26.033547	17.925227	1.547964
C	23.245755	23.607430	2.027218
C	20.678186	16.413814	1.414300
C	17.551991	16.165255	3.667056
C	23.937809	14.201064	2.385025
H	23.736207	17.901239	6.074643
H	24.154572	22.488915	11.150042
H	22.639504	23.369592	11.018960
H	22.880915	22.217375	12.348585
H	22.344866	20.171951	12.205676
H	21.511583	18.199150	12.749514
H	19.909791	18.857886	12.429968
H	20.361570	17.303017	11.729850
H	24.879016	21.500913	9.164639
H	25.938720	23.553014	10.217672
H	27.103110	22.390161	9.552714
H	26.746794	23.880877	8.679518
H	26.011235	22.603967	6.541881
H	26.593234	21.161059	7.387399
H	24.960548	21.195981	6.694843
H	25.122212	24.863719	7.604251
H	23.187957	26.343061	7.185480
H	20.903604	25.527207	7.631125
H	20.067066	22.047636	8.457599
H	18.669599	23.104980	10.259510
H	20.336574	22.825052	10.777092
H	19.811617	24.459352	10.324662
H	19.330109	23.681095	6.639228
H	18.084468	23.277611	7.831849
H	18.903064	24.833744	7.918026
H	19.055290	20.207093	9.658282
H	16.366287	19.298807	10.821812
H	17.860422	19.467876	11.749988
H	17.112942	20.892181	11.005333
H	17.082128	21.170490	8.495978
H	17.933749	20.023692	7.443488
H	16.420650	19.551912	8.229130
H	16.506709	17.503766	9.320538
H	17.048581	15.101498	9.098337
H	19.413043	14.380845	9.059905
H	22.318377	16.696266	9.338953
H	21.281811	14.080297	10.546069

H	22.959404	14.645581	10.553812
H	21.722246	15.527950	11.463312
H	21.867594	15.636281	7.116849
H	23.024921	14.679655	8.065195
H	21.347237	14.155764	7.918357
H	21.089634	15.403167	1.499859
H	20.689799	16.704399	0.361637
H	19.635683	16.353909	1.741906
H	21.641809	18.564322	0.526376
H	23.373126	19.685710	-0.230897
H	24.461336	20.631767	0.805108
H	22.783384	21.137291	0.579103
H	19.224458	17.491411	3.714346
H	17.750893	15.691592	2.699021
H	16.856599	16.996821	3.501060
H	17.032594	15.430030	4.292796
H	18.099705	16.492002	6.381102
H	17.762541	18.062316	5.631649
H	19.396582	17.669305	6.204166
H	18.556352	14.164940	5.237375
H	20.226796	12.349776	5.384337
H	22.554374	12.767032	4.660607
H	23.794090	16.065065	3.425846
H	24.968725	14.363156	2.047077
H	23.268283	14.492368	1.568803
H	23.807601	13.125233	2.555955
H	24.482457	15.222491	5.667052
H	25.688738	14.844247	4.420968
H	24.614653	13.576048	5.022166
H	25.310335	17.832120	3.564050
H	26.791240	18.454319	0.956382
H	25.051055	18.147873	1.126126
H	26.207352	16.848337	1.430801
H	27.423694	16.674908	3.589967
H	27.632024	18.101751	4.621002
H	28.311335	18.069958	2.983956
H	28.084682	20.174537	3.391146
H	27.892136	22.624395	3.587784
H	25.664250	23.689930	3.488655
H	22.468332	21.854183	2.983753
H	23.931231	24.439890	2.225758
H	22.242567	24.034241	1.906174
H	23.543577	23.158280	1.074800
H	22.885389	22.616821	5.339479
H	21.935596	23.799543	4.421037
H	23.653254	24.078174	4.725886
H	23.623532	18.321170	9.488473
H	24.866463	19.494394	8.803056
H	24.098515	16.739127	7.701212
H	25.949259	17.134535	6.119179
H	26.224772	18.751219	6.765410
H	26.977320	17.772676	8.943067
H	26.663176	16.151515	8.345762
H	28.706420	18.090040	7.138175
H	28.394763	16.460421	6.560024
H	29.458632	17.165009	9.362700
H	30.415830	16.495573	8.031841
H	29.141298	15.523391	8.783707
H	20.380570	19.899399	6.774945
H	21.539990	21.122765	6.295346

H	20.023503	19.230380	4.443999
H	21.206825	20.388185	3.879389
H	18.683126	21.133227	5.412774
H	19.877417	22.257810	4.782605
H	19.412034	21.415166	2.444715
H	18.195484	20.327359	3.091738
H	18.113892	23.383558	3.305977
H	16.899957	22.298410	3.963631
H	17.598458	22.558124	0.975139
H	16.154273	23.217875	1.758704
H	16.375275	21.465305	1.638084

184

[C2] scf done: -3350.728814 / Energies= -3348.996597 / Enthalpies= -3348.995652 / Free Energies= -3349.242087

C	-0.419368	-1.964879	6.792628
C	-0.966953	-1.104784	5.811881
C	-1.774351	-1.624698	4.772613
C	-2.011329	-3.001955	4.733850
C	-1.475176	-3.856784	5.689991
C	-0.687264	-3.334770	6.708680
N	-0.624328	0.285118	5.819390
C	-1.422552	1.163094	6.426325
C	-2.677665	0.669873	7.112352
C	-2.362864	-0.735366	3.685910
C	-1.660285	-0.973182	2.340372
C	0.455370	-1.446073	7.925290
C	1.897251	-1.960894	7.800277
C	-1.188907	2.550847	6.477903
C	-0.137961	3.317450	5.937682
C	-0.215673	4.812708	6.154081
N	0.890794	2.810388	5.260876
C	1.906495	3.678590	4.748755
C	3.067514	3.926547	5.517471
C	4.088109	4.702176	4.959103
C	3.978901	5.226331	3.676467
C	2.833985	4.974677	2.929485
C	1.785028	4.205358	3.441655
C	3.244823	3.351184	6.915427
C	4.322312	2.255508	6.925950
C	0.568343	3.923321	2.571421
C	0.916191	2.928291	1.453012
C	3.561775	4.430377	7.960139
C	-0.050158	5.199298	1.983768
C	-3.880276	-0.914669	3.535119
C	-0.121694	-1.789662	9.306549
Mg	1.132932	0.802619	4.876821
C	2.745580	-0.223411	3.952733
C	2.437019	-1.280187	2.880602
C	3.660790	-1.948023	2.228806
C	4.484768	-2.826659	3.170239
C	5.679175	-3.497797	2.489647
C	6.498037	-4.374531	3.435328
C	-1.788814	0.318993	-3.274834
C	-3.320942	0.422290	-3.317361
C	-3.948690	1.051327	-2.066588
C	-5.473113	1.164060	-2.122137
C	-6.091632	1.789312	-0.870488
C	-7.613542	1.900880	-0.937922
Mg	-0.755030	-0.561109	-4.906200
N	0.893446	0.095731	-5.949030

C	1.492690	-0.605727	-6.909366
C	1.077406	-1.881842	-7.337803
C	-0.000744	-2.679300	-6.907948
N	-0.869437	-2.326670	-5.960585
C	-1.948412	-3.203094	-5.618210
C	-3.177562	-3.109409	-6.313568
C	-4.249026	-3.901166	-5.890282
C	-4.125631	-4.771878	-4.813685
C	-2.911828	-4.861713	-4.143152
C	-1.809737	-4.090271	-4.525041
C	-3.370616	-2.158794	-7.487103
C	-4.287289	-0.986510	-7.105577
C	-0.506528	-4.220049	-3.748930
C	-0.659449	-3.702219	-2.310637
C	1.406638	1.377915	-5.573722
C	0.880261	2.545064	-6.174250
C	1.326174	3.790868	-5.722776
C	2.268482	3.896613	-4.706454
C	2.778806	2.742746	-4.123234
C	2.365080	1.473256	-4.537779
C	-0.178320	2.477419	-7.265828
C	-1.548289	2.916467	-6.725086
C	2.922877	0.239123	-3.843121
C	2.357166	0.109894	-2.420014
C	2.704093	-0.024473	-7.604563
C	4.457549	0.216567	-3.816828
C	0.199376	3.294023	-8.509226
C	-0.157081	-4.019232	-7.593048
C	-3.906489	-2.870613	-8.737905
C	0.027258	-5.660065	-3.748905
H	1.845615	-0.823940	2.071784
H	0.692460	5.186221	6.638983
H	-0.293072	5.337060	5.194925
H	-1.078000	5.083014	6.767362
H	-1.942066	3.120612	7.010013
H	-2.443544	-0.118205	7.835608
H	-3.192793	1.481233	7.631099
H	-3.369170	0.229231	6.385647
H	2.296286	2.885933	7.206151
H	2.800514	5.218462	7.970539
H	3.604648	3.988015	8.962727
H	4.530548	4.907541	7.770700
H	5.303561	2.666573	6.659087
H	4.407285	1.804180	7.922352
H	4.094861	1.459888	6.207299
H	4.987192	4.895806	5.539689
H	4.784011	5.826407	3.259653
H	2.753587	5.380949	1.923976
H	-0.193341	3.453992	3.203917
H	-0.969465	4.958259	1.436953
H	-0.302071	5.924550	2.765931
H	0.628384	5.693973	1.278968
H	1.330527	1.996593	1.856244
H	0.026772	2.679678	0.861500
H	1.665766	3.349246	0.771886
H	-2.182428	0.306656	3.972200
H	-4.136275	-1.918538	3.176321
H	-4.405187	-0.759347	4.484503
H	-4.276575	-0.196643	2.807395
H	-2.049329	-0.293265	1.572803

H	-0.578096	-0.819473	2.416823
H	-1.818615	-2.000496	1.990644
H	-2.625782	-3.412536	3.935962
H	-1.670769	-4.925119	5.641052
H	-0.268627	-4.004914	7.456222
H	0.485058	-0.353253	7.848367
H	-0.147205	-2.872792	9.475107
H	0.494252	-1.346782	10.098616
H	-1.143785	-1.412552	9.423660
H	2.335945	-1.695229	6.832100
H	2.528617	-1.536622	8.590756
H	1.937658	-3.053125	7.891114
H	-1.100701	-4.065672	-8.147834
H	-0.188322	-4.831195	-6.858951
H	0.662223	-4.208161	-8.290005
H	1.682972	-2.318647	-8.123662
H	3.076027	-0.693617	-8.383494
H	3.512784	0.160745	-6.888965
H	2.464623	0.943098	-8.059058
H	-2.390839	-1.740885	-7.742946
H	-3.271264	-3.714497	-9.029821
H	-3.950537	-2.172599	-9.582619
H	-4.919741	-3.259049	-8.581306
H	-5.294156	-1.339754	-6.851866
H	-4.379439	-0.279710	-7.939488
H	-3.905287	-0.441314	-6.235461
H	-5.199868	-3.832067	-6.413462
H	-4.971650	-5.378119	-4.499562
H	-2.815833	-5.544476	-3.301910
H	0.242008	-3.595594	-4.249678
H	0.997641	-5.706584	-3.240224
H	0.160374	-6.042431	-4.767070
H	-0.652646	-6.342355	-3.225054
H	-1.005356	-2.662726	-2.292170
H	0.297521	-3.754458	-1.777262
H	-1.387595	-4.301092	-1.750291
H	2.594321	-0.639006	-4.410017
H	4.866840	1.031191	-3.207884
H	4.881189	0.309815	-4.823241
H	4.815162	-0.725341	-3.384184
H	2.715642	-0.809425	-1.941189
H	1.261073	0.090931	-2.421107
H	2.667835	0.955895	-1.795264
H	3.510930	2.828296	-3.323738
H	2.602338	4.874664	-4.368807
H	0.924379	4.695235	-6.173673
H	-0.266383	1.430817	-7.578216
H	0.245033	4.367904	-8.293399
H	-0.547730	3.150790	-9.299147
H	1.175000	2.993763	-8.907774
H	-1.849841	2.318090	-5.857090
H	-2.321805	2.816005	-7.496483
H	-1.526368	3.965179	-6.404433
H	3.350076	-0.685297	4.751606
H	3.407029	0.544333	3.513943
H	1.795797	-2.071047	3.300838
H	3.325470	-2.563230	1.378286
H	4.309007	-1.164979	1.805755
H	4.846663	-2.223874	4.014748
H	3.833331	-3.601336	3.604316

H	6.327894	-2.724201	2.053773
H	5.322397	-4.104535	1.644510
H	6.898264	-3.787229	4.271292
H	7.346091	-4.842383	2.921288
H	5.883562	-5.177222	3.862070
H	-1.505769	-0.228633	-2.358164
H	-1.369768	1.329772	-3.129566
H	-3.766457	-0.573986	-3.463963
H	-3.636645	1.016121	-4.190846
H	-3.658068	0.456321	-1.186900
H	-3.512789	2.050635	-1.913509
H	-5.761791	1.759155	-3.002395
H	-5.906654	0.164296	-2.278684
H	-5.657262	2.787201	-0.713155
H	-5.806771	1.193622	0.009033
H	-7.927723	2.519874	-1.787655
H	-8.026423	2.351739	-0.027724
H	-8.077787	0.914320	-1.060673

181

[D1] scf done: -3637.752165 / Energies= -3636.076840 / Enthalpies= -3636.075895 / Free Energies= -3636.333279

C	10.496741	15.477121	6.345280
C	11.288714	14.794982	7.298062
C	12.263788	15.492117	8.050333
C	12.425831	16.863082	7.830992
C	11.653087	17.543472	6.896991
C	10.698640	16.848934	6.163732
N	11.167587	13.375770	7.435621
C	10.364409	12.865636	8.369678
C	10.198283	11.488876	8.613141
C	10.795285	10.375858	7.988645
C	10.417896	9.015926	8.533235
C	13.157760	14.786066	9.060710
C	13.142685	15.462275	10.438872
C	9.449389	14.760723	5.504298
C	8.053290	15.381305	5.658949
Mg	12.269314	12.229267	6.128105
N	11.652828	10.455637	6.973122
C	12.207806	9.262648	6.410617
C	13.439536	8.767772	6.899303
C	14.010555	7.655213	6.274528
C	13.392890	7.037108	5.193556
C	12.183981	7.533275	4.720376
C	11.571129	8.643045	5.310168
C	14.170020	9.435407	8.055831
C	14.553607	8.446270	9.165073
C	10.261475	9.165151	4.736749
C	9.174961	8.082267	4.672014
C	15.409426	10.192855	7.553691
C	10.479522	9.793618	3.351526
C	9.567715	13.803327	9.250014
C	14.596922	14.673651	8.534191
C	9.860283	14.719128	4.024293
C	13.489151	12.543723	4.422226
C	14.468050	13.726075	4.368403
C	15.242783	13.842481	3.049512
C	16.219956	15.018231	2.997619
C	16.981038	15.127639	1.675254
C	17.956300	16.302577	1.632841
Si	20.770736	12.779604	7.617041

C	21.128608	14.237197	6.494784
C	20.238285	15.319667	6.412246
C	20.514373	16.417469	5.597701
C	21.688072	16.450342	4.846124
C	22.583025	15.381971	4.910566
C	22.303565	14.287196	5.726391
C	25.569820	18.066984	7.175854
C	26.499307	17.475746	8.244850
C	25.750398	17.303093	9.576101
C	27.767111	18.294381	8.441978
C	29.050615	17.700697	8.419093
C	30.207315	18.479593	8.653773
C	30.055094	19.847506	8.899500
C	28.799514	20.443065	8.918291
C	27.669226	19.666197	8.692791
N	29.173390	16.283537	8.255899
Mg	29.132340	15.158796	9.966029
N	29.360102	13.379483	8.978326
C	29.461006	13.283800	7.653202
C	29.431634	14.382531	6.771996
C	29.304804	15.760272	7.037562
C	29.323825	16.681925	5.838384
C	31.598304	17.862656	8.686710
C	32.588196	18.583423	7.761222
C	29.413597	12.200055	9.788241
C	28.212976	11.553692	10.162154
C	28.284090	10.454734	11.023736
C	29.501662	9.997427	11.513271
C	30.674670	10.646577	11.146082
C	30.657984	11.749015	10.286740
C	26.853225	12.044129	9.686085
C	26.067560	12.681247	10.843430
C	31.962657	12.456518	9.949550
C	33.041724	11.496944	9.428955
C	29.622668	11.918294	7.022953
C	32.480965	13.245116	11.163135
C	26.028263	10.935491	9.017683
C	32.138212	17.814648	10.125025
H	28.976950	15.558487	11.633942
H	21.510294	12.878477	8.906012
H	21.179269	11.505952	6.966025
H	10.233051	14.440093	9.843290
H	8.952407	14.477512	8.644572
H	8.916297	13.252665	9.932149
H	9.510458	11.247231	9.415351
H	9.708138	9.099425	9.359127
H	9.973133	8.391364	7.750930
H	11.306119	8.481745	8.888436
H	12.775617	13.767698	9.191437
H	12.124589	15.560070	10.832102
H	13.727204	14.874217	11.156524
H	13.581878	16.466255	10.404363
H	15.039947	15.666087	8.387218
H	15.228249	14.123613	9.242890
H	14.634404	14.155047	7.569425
H	13.175533	17.407531	8.400407
H	11.795485	18.609819	6.740428
H	10.096529	17.381845	5.431352
H	9.386710	13.726381	5.861405
H	8.023338	16.409683	5.280067

H	7.313690	14.800712	5.094509
H	7.735132	15.405168	6.707233
H	10.838498	14.243503	3.890870
H	9.124279	14.159416	3.433951
H	9.926614	15.730390	3.604871
H	9.896566	9.953740	5.404318
H	9.001902	7.622545	5.651543
H	8.227176	8.514658	4.329226
H	9.441624	7.280963	3.972795
H	10.832393	9.045554	2.631202
H	9.543212	10.215627	2.965665
H	11.228148	10.593087	3.385511
H	11.706525	7.050390	3.870776
H	13.853527	6.173835	4.719725
H	14.959506	7.267868	6.638133
H	13.489828	10.171856	8.498371
H	15.293823	7.714393	8.821034
H	14.994049	8.981244	10.015028
H	13.682344	7.890519	9.529733
H	15.147309	10.932701	6.787953
H	15.907526	10.715791	8.379331
H	16.135251	9.504448	7.104187
H	28.788464	11.262587	7.295499
H	30.532785	11.428918	7.387047
H	29.674608	11.984843	5.934105
H	29.526893	14.127881	5.722701
H	28.398961	17.266533	5.782734
H	29.440633	16.123187	4.907249
H	30.143041	17.405037	5.917968
H	27.024542	12.821948	8.933320
H	26.568697	10.476665	8.181925
H	25.086879	11.342807	8.629813
H	25.772798	10.138023	9.724997
H	25.850740	11.942019	11.624063
H	25.111628	13.084941	10.487904
H	26.630729	13.495835	11.314143
H	27.366852	9.951445	11.320688
H	29.536095	9.142070	12.183415
H	31.624826	10.293558	11.539927
H	31.754832	13.177465	9.151045
H	33.357873	10.783784	10.199102
H	33.931223	12.058166	9.118501
H	32.688628	10.919479	8.567060
H	31.736056	13.961874	11.528802
H	33.392835	13.798560	10.906815
H	32.718318	12.571456	11.995507
H	31.513044	16.830084	8.329667
H	32.216672	18.632273	6.731427
H	33.549898	18.056531	7.749382
H	32.782198	19.610231	8.093000
H	32.259417	18.826012	10.531600
H	33.116610	17.319453	10.156561
H	31.458664	17.274800	10.795204
H	30.936609	20.456662	9.085640
H	28.701619	21.508139	9.113218
H	26.687839	20.133951	8.717728
H	26.796462	16.478530	7.901505
H	25.163852	19.038533	7.481582
H	24.719109	17.398548	6.999542
H	26.091569	18.211287	6.222759

H	26.387677	16.849279	10.344557
H	24.865890	16.668037	9.443780
H	25.414380	18.272137	9.964647
H	14.051324	11.609009	4.251912
H	12.815942	12.615195	3.548525
H	13.933251	14.672789	4.541714
H	15.790673	12.903857	2.874291
H	14.522138	13.930143	2.221679
H	15.670736	15.955587	3.177114
H	16.943125	14.928258	3.822805
H	16.260074	15.219057	0.849992
H	17.528845	14.191333	1.495973
H	17.433102	17.256520	1.776666
H	18.483401	16.354753	0.672703
H	18.712182	16.217401	2.423390
H	19.317535	12.727474	7.926569
H	19.313866	15.306699	6.986451
H	19.811003	17.244882	5.547033
H	21.903007	17.303673	4.207739
H	23.495339	15.398848	4.319254
H	23.009219	13.458556	5.756640
H	15.199640	13.649562	5.188917

181

[TS-D1D2] scf done: -3637.714543 / Energies= -3636.039826 / Enthalpies= -3636.038882 / Free Energies= -3636.275156

C	16.186403	16.317091	8.407647
C	15.180746	15.577654	7.739716
C	14.376181	16.190028	6.747242
C	14.581185	17.543589	6.460427
C	15.560532	18.281088	7.115457
C	16.355043	17.664498	8.073669
N	15.003904	14.182553	8.016976
Mg	16.042208	12.792025	6.935512
N	15.065833	11.151515	7.676289
C	14.292408	11.256128	8.756488
C	13.952691	12.470034	9.386013
C	14.230123	13.805182	9.037610
C	13.603152	14.852737	9.932809
C	13.275851	15.432377	6.015036
C	11.882660	15.827290	6.530661
C	17.095845	15.703231	9.463769
C	17.019136	16.453034	10.803196
C	15.245573	9.880931	7.040401
C	16.446082	9.160824	7.251999
C	16.636535	7.962487	6.556347
C	15.682436	7.479752	5.668981
C	14.504971	8.191896	5.475568
C	14.257100	9.389821	6.153015
C	17.501616	9.619924	8.249326
C	17.413754	8.812995	9.555191
C	12.941839	10.118235	5.906397
C	11.723822	9.212952	6.145259
C	18.926011	9.562358	7.683537
C	13.670748	10.019549	9.371054
C	12.885626	10.718493	4.494570
C	13.344236	15.616496	4.492584
C	18.554166	15.644256	8.984171
C	16.572143	12.895882	4.749288
C	16.826405	13.028618	3.240091
C	15.687152	12.557764	2.331218

C	15.993044	12.745778	0.843958
C	14.864029	12.280069	-0.076832
C	15.175668	12.477814	-1.558991
Si	18.544505	13.007654	5.651942
C	18.936824	14.760334	5.017708
C	17.999865	15.787026	4.829554
C	18.386234	17.053622	4.390073
C	19.724719	17.313536	4.100745
C	20.675196	16.307640	4.276683
C	20.283936	15.056389	4.750806
C	23.364800	18.338039	7.508188
C	24.059124	17.418454	8.522365
C	23.055335	16.920610	9.574642
C	25.256499	18.076996	9.192238
C	26.522597	17.448657	9.244286
C	27.601977	18.061938	9.921436
C	27.393891	19.305809	10.525324
C	26.156807	19.936928	10.472916
C	25.100523	19.319841	9.813095
N	26.685299	16.137411	8.693547
Mg	26.312122	14.564026	9.949651
H	25.860153	14.471850	11.609673
C	28.961315	17.388624	10.042090
C	30.110984	18.289608	9.570310
C	27.048558	15.988311	7.419880
C	27.297598	17.218273	6.575329
C	27.221337	14.744659	6.782088
C	27.070449	13.435709	7.281283
C	27.326117	12.309375	6.304592
N	26.721223	13.143605	8.533353
C	26.604083	11.776323	8.942143
C	27.716446	11.124058	9.522778
C	27.557475	9.815551	9.988997
C	26.337036	9.157704	9.892742
C	25.248511	9.812682	9.328932
C	25.354308	11.121201	8.848132
C	29.060690	11.818685	9.687122
C	30.219564	11.015074	9.080913
C	24.121711	11.813557	8.284799
C	23.132592	12.161114	9.409159
C	23.424810	10.995250	7.188971
C	29.334915	12.134245	11.166235
C	29.204987	16.906880	11.481271
H	19.866285	12.876976	6.433256
H	18.787355	11.780013	4.809980
H	13.267134	15.724604	9.366366
H	12.757039	14.439249	10.486446
H	14.338095	15.211954	10.662466
H	13.325989	12.356269	10.263973
H	13.551314	10.148458	10.450306
H	12.672904	9.849387	8.949208
H	14.263031	9.122393	9.180266
H	16.762453	14.674034	9.638626
H	15.990904	16.543197	11.169865
H	17.607260	15.928217	11.565258
H	17.426331	17.467437	10.716974
H	18.954060	16.651388	8.816475
H	19.183263	15.154602	9.737325
H	18.654865	15.091130	8.045979
H	17.127552	18.241717	8.575832

H	15.704855	19.332240	6.878507
H	13.958895	18.029139	5.712599
H	13.410419	14.365125	6.228342
H	11.693059	16.894941	6.365291
H	11.102867	15.262016	6.005663
H	11.775183	15.630998	7.602227
H	14.334856	15.368687	4.096377
H	12.609790	14.968337	4.000017
H	13.118385	16.647331	4.195485
H	12.877060	10.951321	6.614129
H	11.751624	8.743789	7.134693
H	10.797449	9.794944	6.069594
H	11.665426	8.408986	5.402107
H	12.953527	9.936854	3.728229
H	11.941466	11.256021	4.341680
H	13.707490	11.420790	4.324039
H	13.757913	7.807754	4.784910
H	15.853820	6.549116	5.133839
H	17.550433	7.395993	6.714791
H	17.290704	10.665791	8.503066
H	17.597410	7.747818	9.368282
H	18.164861	9.163494	10.273442
H	16.429610	8.904438	10.026528
H	19.007887	10.102096	6.734927
H	19.628598	10.017482	8.390429
H	19.255667	8.530208	7.514125
H	26.427901	11.694130	6.181656
H	28.111201	11.641112	6.674599
H	27.623642	12.690504	5.325352
H	27.516492	14.803759	5.740788
H	26.391606	17.830325	6.503501
H	27.617269	16.950623	5.565876
H	28.065476	17.853292	7.030359
H	24.446435	12.755633	7.828870
H	24.110839	10.748511	6.370438
H	22.583571	11.560467	6.771566
H	23.022275	10.052807	7.578738
H	22.761593	11.252156	9.898624
H	22.269010	12.705114	9.008113
H	23.601067	12.778324	10.185450
H	24.292096	9.299242	9.264859
H	26.232914	8.140085	10.261147
H	28.405028	9.304801	10.440100
H	29.011058	12.772223	9.149490
H	30.375332	10.065584	9.606559
H	31.154328	11.584559	9.149344
H	30.041478	10.783707	8.024705
H	28.530479	12.734821	11.607035
H	30.276123	12.687010	11.276856
H	29.414818	11.213221	11.756575
H	28.953498	16.504601	9.394411
H	29.954000	18.640402	8.544025
H	31.060438	17.741558	9.599507
H	30.223116	19.173045	10.209777
H	29.242491	17.753487	12.177556
H	30.158311	16.368862	11.554273
H	28.406345	16.238481	11.824254
H	28.215534	19.786080	11.051740
H	26.014620	20.903389	10.950223
H	24.130432	19.810352	9.784421

H	24.422892	16.543382	7.972531
H	22.906525	19.207186	7.994896
H	22.566922	17.796275	6.986864
H	24.067916	18.713465	6.755738
H	23.526229	16.239449	10.293633
H	22.221413	16.392662	9.096788
H	22.641383	17.760016	10.146723
H	16.180427	11.863437	4.901017
H	15.723239	13.592445	4.959988
H	17.063637	14.074575	3.011532
H	15.479689	11.495658	2.528132
H	14.764398	13.102801	2.581364
H	16.205574	13.807572	0.648258
H	16.915291	12.201487	0.591240
H	13.941843	12.821867	0.178907
H	14.654782	11.217945	0.115575
H	15.356863	13.535349	-1.787864
H	14.348785	12.137129	-2.192934
H	16.072707	11.919102	-1.853319
H	17.880272	12.842231	7.113340
H	16.947361	15.609610	5.038119
H	17.639407	17.835428	4.274638
H	20.027009	18.296279	3.745998
H	21.722241	16.501502	4.053895
H	21.039434	14.291723	4.924851
H	17.731640	12.455655	2.989883

181

[D2] scf done: -3637.768771 / Energies= -3636.093838 / Enthalpies= -3636.092893 / Free Energies= -3636.341797

C	24.632790	12.751809	6.539821
C	25.327088	12.943120	7.757272
C	25.361020	11.923565	8.735947
C	24.698831	10.720702	8.471271
C	24.016796	10.518950	7.277249
C	23.985296	11.531544	6.324984
N	25.919531	14.216220	8.040265
C	27.178242	14.461599	7.680258
C	27.969549	13.379065	6.980872
C	26.067417	12.111890	10.070865
C	25.050645	12.222015	11.218056
C	24.537191	13.850923	5.491536
C	24.877532	13.360647	4.077680
C	27.843973	15.684213	7.899771
C	27.385301	16.876981	8.491280
C	28.372140	18.021619	8.551898
N	26.159144	17.049737	8.985834
Mg	24.726744	15.589692	8.972252
C	25.787061	18.308579	9.557057
C	25.182156	19.294564	8.743067
C	24.745962	20.480953	9.340446
C	24.894883	20.700761	10.704820
C	25.484617	19.721843	11.495894
C	25.937534	18.518187	10.947500
C	24.958710	19.079709	7.252887
C	23.468203	18.855521	6.952662
C	26.538775	17.458936	11.859859
C	25.456838	16.828330	12.751199
C	25.514952	20.226894	6.398175
C	27.695891	17.998743	12.711577
C	27.088623	11.002326	10.358122

C	23.145508	14.502411	5.516505
C	20.144302	16.821522	2.114914
C	19.781147	17.159889	3.417625
C	19.253731	16.185921	4.266689
C	19.090187	14.878067	3.812181
C	19.454140	14.515067	2.503028
C	19.982172	15.511064	1.665313
Si	19.240772	12.754405	1.884130
C	17.453341	12.153855	1.942981
C	17.267444	10.736840	1.382468
C	15.817987	10.245718	1.431065
C	15.634770	8.842729	0.850926
C	14.190771	8.341166	0.904495
C	14.017842	6.941050	0.319061
Mg	16.223667	12.615889	7.424101
N	14.878073	11.301444	8.230544
C	14.411947	10.247866	7.381441
C	15.138040	9.036437	7.304265
C	14.705724	8.052640	6.410036
C	13.588785	8.246598	5.605588
C	12.889157	9.445383	5.682319
C	13.280659	10.460999	6.559892
C	16.391005	8.797177	8.134275
C	17.646947	8.850337	7.249331
C	12.508322	11.772238	6.580901
C	12.767300	12.577547	5.297584
N	16.369448	13.745593	9.119870
C	15.712051	13.469739	10.244153
C	15.913291	14.356901	11.452222
C	17.255527	14.870379	9.060113
C	18.622255	14.702114	9.384162
C	19.488953	15.787636	9.221347
C	19.024815	17.013505	8.756185
C	17.679190	17.167919	8.441994
C	16.774193	16.110917	8.583075
C	19.171407	13.366939	9.865661
C	19.963253	12.669201	8.748396
C	15.316442	16.314562	8.195195
C	15.168595	16.463475	6.672784
C	14.437796	11.386810	9.486893
C	13.455228	10.354726	9.994741
C	14.825619	12.384554	10.400816
C	14.675683	17.506579	8.919882
C	20.032405	13.501228	11.129474
C	16.340237	7.480129	8.920883
C	11.002207	11.569871	6.797307
H	23.112150	15.536443	9.579921
H	20.089571	11.819996	2.682153
H	19.745622	12.711147	0.478937
H	15.688032	15.401284	11.211335
H	15.281823	14.044519	12.286830
H	16.959387	14.332064	11.777926
H	14.372616	12.305306	11.382370
H	13.170687	10.551585	11.030571
H	12.549318	10.341277	9.378994
H	13.884745	9.348441	9.935644
H	18.318430	12.726181	10.116942
H	19.492176	14.015120	11.933175
H	20.320147	12.508249	11.495875
H	20.954809	14.059446	10.934756

H	20.843232	13.258681	8.467113
H	20.311768	11.682552	9.077610
H	19.357675	12.533322	7.843530
H	20.545032	15.673374	9.455427
H	19.713823	17.846516	8.640063
H	17.322239	18.128542	8.077219
H	14.763689	15.417203	8.495390
H	15.139776	18.456181	8.628586
H	13.608986	17.573610	8.673761
H	14.766565	17.413371	10.007987
H	15.590922	15.603692	6.139229
H	14.112151	16.555463	6.391447
H	15.690972	17.358021	6.312532
H	12.881418	12.364042	7.424144
H	10.798288	10.997018	7.708943
H	10.498297	12.539724	6.886945
H	10.538091	11.036508	5.959407
H	12.401064	12.038594	4.415301
H	12.254399	13.546488	5.336902
H	13.837253	12.762365	5.144950
H	12.021323	9.598408	5.045147
H	13.266877	7.467604	4.918956
H	15.257774	7.118340	6.339502
H	16.466290	9.609618	8.865676
H	16.333688	6.608259	8.256212
H	17.220558	7.390905	9.568636
H	15.447505	7.421409	9.553676
H	17.717745	9.798441	6.703065
H	18.554249	8.735703	7.855106
H	17.634714	8.045320	6.504377
H	27.494280	13.106340	6.031863
H	28.002273	12.466233	7.585283
H	28.992893	13.701221	6.777031
H	28.869179	15.714214	7.549133
H	27.986442	18.895778	8.015868
H	29.334030	17.742479	8.116570
H	28.535601	18.340402	9.587177
H	25.269082	14.624185	5.751027
H	25.859039	12.873798	4.043156
H	24.892734	14.205589	3.379188
H	24.136656	12.645009	3.703342
H	22.366853	13.777832	5.252010
H	23.086668	15.330197	4.800129
H	22.897603	14.893435	6.511215
H	23.441315	11.373632	5.397062
H	23.507078	9.577072	7.090029
H	24.715409	9.929358	9.217225
H	26.619270	13.057508	10.024966
H	26.605143	10.025396	10.475903
H	27.629883	11.214434	11.288102
H	27.824887	10.912299	9.551507
H	24.334259	13.034061	11.046346
H	25.559462	12.409273	12.171818
H	24.474011	11.294723	11.321136
H	26.946714	16.664979	11.224288
H	28.479571	18.446909	12.090393
H	28.148868	17.187875	13.294648
H	27.358052	18.763056	13.421455
H	25.021437	17.573327	13.428375
H	25.880110	16.023180	13.364412

H	24.636168	16.410740	12.155809
H	25.590641	19.892819	12.564604
H	24.548067	21.629554	11.150925
H	24.275807	21.244890	8.725461
H	25.494326	18.168950	6.962832
H	24.982214	21.167303	6.582177
H	25.406872	19.992732	5.332369
H	26.577630	20.403040	6.599620
H	23.060374	18.018753	7.532095
H	23.313453	18.640715	5.888353
H	22.879449	19.745465	7.206658
H	17.106033	12.197300	2.985599
H	16.828180	12.865403	1.383959
H	17.622262	10.701661	0.341694
H	15.466892	10.260255	2.473023
H	15.178601	10.953820	0.882636
H	15.983654	8.831132	-0.192926
H	16.282051	8.137432	1.393910
H	13.542338	9.046508	0.364945
H	13.845837	8.349403	1.948261
H	14.324144	6.911272	-0.734022
H	12.974407	6.608606	0.370845
H	14.628291	6.206972	0.859716
H	16.943915	12.721183	5.858975
H	18.666975	14.137233	4.489123
H	18.970209	16.442843	5.284456
H	19.906124	18.181076	3.770852
H	20.553301	17.577452	1.448151
H	20.271618	15.261185	0.646092
H	17.900655	10.032445	1.941699

184

[F1] scf done: -3350.704541 / Energies= -3348.970694 / Enthalpies= -3348.969750 / Free Energies= -3349.189792

C	22.467545	17.675732	8.121982
C	23.135182	16.622403	8.794181
C	23.588896	16.808577	10.123987
C	23.375883	18.043219	10.745531
C	22.718893	19.079153	10.093545
C	22.262238	18.883722	8.796627
N	23.417402	15.379066	8.130697
C	24.511167	15.372127	7.360537
C	25.259377	16.663676	7.080276
C	24.362732	15.733035	10.872937
C	23.865087	15.541157	12.311230
C	21.979282	17.552428	6.686214
C	20.456501	17.393250	6.639161
C	25.086593	14.233217	6.774089
C	24.918780	12.863686	7.060213
C	26.073521	12.018620	6.543437
N	23.913562	12.335182	7.755366
C	24.040402	10.946653	8.103245
C	23.582333	9.937584	7.220365
C	23.779884	8.597608	7.571543
C	24.415415	8.239927	8.753730
C	24.857614	9.236044	9.614536
C	24.684008	10.590951	9.314116
C	22.947614	10.245072	5.871199
C	21.579473	9.571585	5.717895
C	25.251606	11.626644	10.274537
C	24.692873	11.467411	11.694751

C	23.854076	9.827274	4.700663
C	26.787601	11.578438	10.305411
C	25.872058	16.024243	10.869267
C	22.395875	18.739689	5.803222
Mg	22.224468	13.593318	8.227351
C	21.251603	13.012671	10.264852
C	20.316247	13.147005	11.491031
C	20.876711	12.589783	12.805793
C	19.930377	12.758101	13.996007
C	20.487507	12.201569	15.307450
C	19.533962	12.372043	16.488437
C	20.527124	13.216425	6.741827
C	21.300982	13.888819	5.581980
C	20.907015	13.419682	4.175572
C	21.669583	14.127810	3.054244
C	21.275874	13.657752	1.652838
C	22.038964	14.372670	0.539526
Mg	19.473143	13.101951	8.822823
N	17.791871	14.379431	9.215064
C	17.654971	15.751228	8.808298
C	17.983314	16.811217	9.689213
C	17.786075	18.128333	9.262120
C	17.254766	18.416179	8.011520
C	16.931995	17.371887	7.154112
C	17.130009	16.037500	7.523256
C	18.525949	16.574013	11.091036
C	20.028701	16.866080	11.152790
C	16.711662	14.938217	6.557256
C	17.221778	15.180313	5.131065
C	16.776159	13.888417	9.933422
C	16.600051	12.538379	10.278084
C	17.176217	11.367524	9.746167
C	16.354860	10.125375	10.057609
C	15.668883	14.807117	10.419519
N	18.274446	11.315880	8.994582
C	18.569926	10.045550	8.389989
C	18.023320	9.735316	7.120606
C	18.268700	8.473474	6.568994
C	19.030624	7.524895	7.238419
C	19.565654	7.838478	8.481260
C	19.349749	9.084554	9.080443
C	17.128325	10.699182	6.354157
C	17.680923	11.009051	4.956760
C	19.901885	9.319024	10.479784
C	21.414943	9.086591	10.542911
C	17.799022	17.396375	12.166923
C	15.186034	14.750872	6.548157
C	15.690798	10.166337	6.245753
C	19.205589	8.434855	11.528557
H	15.269906	15.416999	9.603933
H	14.849700	14.231802	10.855919
H	16.037583	15.503413	11.178382
H	15.739358	12.349200	10.910824
H	16.098338	10.114057	11.122292
H	15.409825	10.162435	9.503823
H	16.855246	9.192341	9.803842
H	18.381385	15.515314	11.329148
H	16.710572	17.280617	12.118761
H	18.129096	17.081594	13.164170
H	18.019269	18.466547	12.077388

H	20.235512	17.914290	10.914815
H	20.421507	16.667184	12.157670
H	20.591796	16.255941	10.440063
H	18.040067	18.945460	9.932672
H	17.092348	19.447436	7.707121
H	16.509565	17.596282	6.177846
H	17.151656	14.001795	6.917151
H	14.680973	15.673832	6.237015
H	14.899026	13.957780	5.846652
H	14.805499	14.475895	7.536945
H	18.302905	15.350883	5.114822
H	17.003971	14.311738	4.498980
H	16.738869	16.047887	4.665545
H	17.091372	11.637960	6.916326
H	15.261636	9.952182	7.229517
H	15.046154	10.900270	5.746879
H	15.654743	9.239658	5.659999
H	17.733444	10.107103	4.334935
H	17.030716	11.725081	4.439519
H	18.685323	11.438718	5.010813
H	17.841065	8.226816	5.599894
H	19.203001	6.546356	6.796808
H	20.153363	7.092922	9.011180
H	19.711286	10.362620	10.750177
H	19.370548	7.370991	11.318316
H	19.611296	8.642601	12.526270
H	18.125570	8.603392	11.564353
H	21.951610	9.683879	9.801573
H	21.799323	9.347613	11.536046
H	21.669927	8.036341	10.361430
H	22.155247	13.596205	10.532751
H	21.611693	11.967968	10.236969
H	20.069784	14.204514	11.642404
H	19.345832	12.649927	11.314844
H	21.833459	13.085569	13.024939
H	21.110681	11.523206	12.673600
H	18.972043	12.265503	13.771833
H	19.697326	13.825876	14.127654
H	20.721896	11.135253	15.176740
H	21.443178	12.696155	15.533385
H	18.582070	11.857846	16.306208
H	19.960354	11.964654	17.412533
H	19.307651	13.431001	16.665212
H	25.912196	10.946435	6.642012
H	26.988957	12.275348	7.088417
H	26.256054	12.255523	5.489620
H	25.943075	14.441746	6.141572
H	24.677598	17.319940	6.426214
H	26.215342	16.461276	6.592672
H	25.449146	17.225364	7.999116
H	22.801168	11.328093	5.803902
H	24.828124	10.323544	4.730809
H	23.377696	10.079735	3.745358
H	24.030351	8.744611	4.707221
H	21.670994	8.479954	5.684161
H	21.101865	9.887424	4.782910
H	20.906678	9.815460	6.544082
H	23.440197	7.818899	6.893091
H	24.567184	7.192010	9.000920
H	25.362585	8.958281	10.536791

H	24.958269	12.615980	9.908510
H	27.144580	10.618109	10.697331
H	27.184274	12.370321	10.952420
H	27.218269	11.708912	9.307866
H	23.601414	11.537835	11.705230
H	25.090936	12.251585	12.350069
H	24.973990	10.502554	12.133633
H	24.214256	14.788842	10.337651
H	26.275065	16.059256	9.852194
H	26.415948	15.245751	11.418396
H	26.086189	16.986989	11.349937
H	24.083400	16.412565	12.940113
H	24.361230	14.678888	12.771496
H	22.784463	15.368676	12.342383
H	23.740339	18.199293	11.757789
H	22.565151	20.032810	10.593010
H	21.747375	19.693846	8.286265
H	22.424071	16.647134	6.260511
H	21.865129	19.657202	6.083346
H	22.149101	18.531400	4.755271
H	23.469202	18.950793	5.862925
H	20.119079	16.525495	7.213814
H	20.110008	17.273981	5.605131
H	19.957112	18.272505	7.058504
H	20.557778	12.124508	6.573708
H	19.464756	13.476895	6.560215
H	22.390543	13.738605	5.683149
H	21.157792	14.974816	5.635262
H	21.070220	12.334738	4.098042
H	19.826999	13.574365	4.038356
H	21.503495	15.213340	3.130241
H	22.750561	13.976973	3.196289
H	20.195763	13.807528	1.511595
H	21.443974	12.573861	1.574617
H	21.864861	15.455453	0.572175
H	21.733228	14.016591	-0.451170
H	23.120047	14.210056	0.632319

199

[F2] scf done: -3873.550147 / Energies= -3871.692649 / Enthalpies= -3871.691705 / Free Energies= -3871.971910

C	-3.665529	2.252017	0.635903
C	-5.029019	1.883910	0.718124
C	-6.041161	2.781273	0.303770
C	-5.665121	4.036780	-0.183138
C	-4.328709	4.410186	-0.266208
C	-3.341855	3.518514	0.139627
N	-5.380412	0.561542	1.136225
Mg	-5.476745	-0.869894	-0.341256
N	-5.931038	-2.380137	0.980076
C	-6.102302	-2.192418	2.288149
C	-5.956629	-0.952766	2.940310
C	-5.609350	0.311284	2.424547
C	-5.489085	1.436241	3.428907
C	-7.517030	2.410406	0.348095
C	-8.350482	3.414752	1.156852
C	-2.550764	1.296481	1.037139
C	-1.543789	1.930625	2.006648
C	-6.065577	-3.691515	0.422810
C	-4.937507	-4.543179	0.355143
C	-5.069144	-5.777591	-0.287357

C	-6.276467	-6.176742	-0.849218
C	-7.378991	-5.334381	-0.772188
C	-7.299867	-4.088305	-0.142180
C	-3.588271	-4.140014	0.934124
C	-2.997495	-5.211939	1.860683
C	-8.529898	-3.192986	-0.099482
C	-9.740851	-3.892751	0.533884
C	-2.592593	-3.789579	-0.182511
C	-6.474487	-3.371382	3.160131
C	-8.880441	-2.666635	-1.499874
C	-8.088816	2.255375	-1.069809
C	-1.831013	0.747342	-0.204677
C	-5.306754	-0.694288	-2.449488
C	-4.895098	-1.920659	-3.277768
C	-4.884592	-1.688556	-4.794300
C	-4.480923	-2.919100	-5.608307
C	-4.474502	-2.685484	-7.120107
C	-4.075828	-3.923355	-7.921403
Si	0.418511	1.622475	-6.295193
C	-0.662205	3.048075	-5.738895
C	-1.577002	2.894979	-4.683933
C	-2.361254	3.963102	-4.250990
C	-2.245978	5.206848	-4.871520
C	-1.347862	5.377969	-5.924179
C	-0.564855	4.307300	-6.353059
C	2.996012	2.693066	-1.341681
C	4.280239	3.529442	-1.227445
C	4.342185	4.576763	-2.347905
C	4.404522	4.144932	0.159051
C	5.350994	3.678733	1.101286
C	5.417631	4.248422	2.394003
C	4.531574	5.279958	2.718601
C	3.598879	5.747630	1.800237
C	3.541204	5.179142	0.533058
N	6.182105	2.559317	0.777846
Mg	5.418939	0.702564	1.230847
C	3.520540	0.153252	2.004908
C	3.433251	-0.883702	3.134758
C	2.003501	-1.225896	3.574199
C	1.927096	-2.249874	4.707966
C	0.498951	-2.587482	5.139902
C	0.434431	-3.607197	6.275379
C	6.399710	3.746935	3.442902
C	7.250316	4.874508	4.043819
C	7.369504	2.756025	0.207648
C	7.823554	4.168600	-0.086454
C	8.257341	1.723915	-0.154359
C	8.139283	0.328871	-0.004057
C	9.298487	-0.500643	-0.510403
N	7.092179	-0.284559	0.547489
C	7.084361	-1.710392	0.674341
C	7.609836	-2.314119	1.841348
C	7.495435	-3.699712	1.988382
C	6.884739	-4.483268	1.016082
C	6.378095	-3.880551	-0.129118
C	6.465653	-2.498696	-0.324307
C	8.269441	-1.499300	2.945420
C	9.681347	-2.000923	3.281076
C	5.883350	-1.886386	-1.590465
C	4.351609	-2.005192	-1.612744

C	6.488242	-2.495633	-2.863764
C	7.396380	-1.472216	4.209167
C	5.667045	2.972301	4.549819
H	1.670754	1.526537	-5.493460
H	0.810982	1.809259	-7.717947
H	-4.466039	1.828924	3.446006
H	-6.138722	2.275413	3.159029
H	-5.748314	1.101508	4.435641
H	-6.125179	-0.977789	4.010804
H	-5.695855	-4.141320	3.127181
H	-6.619416	-3.068851	4.199415
H	-7.394649	-3.844604	2.800461
H	-3.010301	0.446833	1.554467
H	-2.040031	2.338258	2.894776
H	-0.814068	1.183129	2.338089
H	-0.982017	2.746085	1.536210
H	-1.344388	1.555386	-0.764202
H	-1.058242	0.023984	0.082188
H	-2.529090	0.250162	-0.889219
H	-2.296993	3.811660	0.070365
H	-4.056908	5.393197	-0.643199
H	-6.435031	4.734453	-0.505039
H	-7.601084	1.439042	0.847800
H	-8.361328	4.404737	0.685628
H	-9.390492	3.074533	1.229884
H	-7.963232	3.536681	2.174565
H	-7.528476	1.514527	-1.650911
H	-9.138524	1.938712	-1.031781
H	-8.044514	3.204275	-1.617960
H	-8.289178	-2.327553	0.528457
H	-9.509756	-4.273215	1.535200
H	-10.581091	-3.193634	0.623116
H	-10.082023	-4.740240	-0.072311
H	-9.144742	-3.488840	-2.175969
H	-9.736675	-1.982274	-1.453459
H	-8.037719	-2.129867	-1.950441
H	-8.322448	-5.648660	-1.212698
H	-6.357078	-7.140618	-1.345602
H	-4.207470	-6.437855	-0.352193
H	-3.737981	-3.236098	1.534596
H	-2.739460	-6.126481	1.313953
H	-2.078385	-4.841863	2.330129
H	-3.696611	-5.488725	2.657820
H	-2.973598	-2.988951	-0.826936
H	-1.635234	-3.460648	0.239958
H	-2.398492	-4.657888	-0.823798
H	2.903446	-0.203481	1.160681
H	3.021155	1.080839	2.334135
H	3.935266	-1.817009	2.835759
H	3.983434	-0.525558	4.020103
H	1.445517	-1.602531	2.702872
H	1.494809	-0.299648	3.883255
H	2.486401	-1.871860	5.577724
H	2.439037	-3.174105	4.398705
H	-0.013591	-1.664229	5.446928
H	-0.059586	-2.968798	4.272631
H	0.955743	-3.239054	7.167908
H	-0.600703	-3.827270	6.562233
H	0.907696	-4.553629	5.985453
H	8.957531	-1.238941	-1.244084

H	9.759410	-1.066187	0.306669
H	10.064099	0.125104	-0.974180
H	9.178218	2.056374	-0.619602
H	7.131256	4.663933	-0.776192
H	8.822907	4.183236	-0.526734
H	7.834518	4.771609	0.827976
H	6.134102	-0.819512	-1.589312
H	7.580777	-2.412127	-2.873338
H	6.102101	-1.981696	-3.752220
H	6.236405	-3.558114	-2.962839
H	4.037531	-3.055889	-1.632049
H	3.938275	-1.515867	-2.503197
H	3.899166	-1.543553	-0.727858
H	5.901411	-4.495161	-0.889325
H	6.804674	-5.559146	1.150282
H	7.889977	-4.173515	2.884404
H	8.364149	-0.467916	2.588486
H	9.661915	-3.011133	3.706674
H	10.153274	-1.342370	4.020167
H	10.324506	-2.028978	2.394381
H	6.393725	-1.084086	3.998375
H	7.852625	-0.841644	4.982330
H	7.274382	-2.479257	4.626056
H	7.085661	3.049309	2.949720
H	7.780237	5.437873	3.267523
H	7.997801	4.461842	4.732079
H	6.640154	5.586433	4.611866
H	4.967803	3.623544	5.088243
H	6.379284	2.566532	5.278868
H	5.084829	2.138578	4.139656
H	4.571254	5.722554	3.711201
H	2.918027	6.550600	2.071695
H	2.807110	5.543532	-0.181869
H	5.129337	2.851237	-1.368014
H	3.482053	5.255903	-2.318939
H	4.336008	4.084660	-3.327742
H	5.249599	5.187721	-2.281542
H	2.951612	1.914337	-0.571422
H	2.934250	2.208963	-2.323929
H	2.106004	3.322646	-1.222047
H	-6.283314	-0.336672	-2.824023
H	-4.615443	0.136544	-2.672683
H	-3.891537	-2.260928	-2.976631
H	-5.884999	-1.354050	-5.110038
H	-4.202127	-0.856196	-5.025076
H	-3.481161	-3.253723	-5.290896
H	-5.164782	-3.749415	-5.373779
H	-3.787759	-1.859705	-7.355488
H	-5.471909	-2.348570	-7.437722
H	-3.069077	-4.263965	-7.648634
H	-4.078810	-3.724858	-8.999739
H	-4.766153	-4.755470	-7.734216
H	-0.304865	0.331453	-6.151766
H	-1.688312	1.928587	-4.195646
H	-3.062126	3.823000	-3.431755
H	-2.859912	6.040380	-4.538750
H	-1.260225	6.344398	-6.414928
H	0.125495	4.455362	-7.181611
H	-5.562987	-2.769072	-3.062625

199

[TS-F2F3] scf done: -3873.493039 / Energies= -3871.636157 / Enthalpies= -3871.635213 / Free Energies= -3871.883268

C	17.616554	16.958531	9.242003
C	16.755336	15.989734	8.672217
C	15.888293	16.335782	7.605203
C	15.905274	17.649578	7.126257
C	16.759287	18.604428	7.666791
C	17.602060	18.253844	8.713084
N	16.792174	14.626846	9.113621
Mg	17.851605	13.246914	8.016490
N	17.067899	11.597006	8.950223
C	16.477311	11.719415	10.144192
C	16.137575	12.943930	10.742905
C	16.186837	14.263075	10.242980
C	15.477291	15.284775	11.108437
C	14.900671	15.333820	7.022858
C	13.543325	15.430538	7.738333
C	18.543382	16.660775	10.413207
C	18.258276	17.582160	11.611021
C	17.057234	10.320399	8.293889
C	18.168339	9.450217	8.369474
C	18.139150	8.261160	7.631750
C	17.045612	7.917688	6.847891
C	15.947079	8.768002	6.800159
C	15.925508	9.970828	7.512077
C	19.384544	9.742783	9.235020
C	19.581188	8.668552	10.316328
C	14.673853	10.838986	7.444092
C	13.462142	10.129632	8.069860
C	20.660052	9.875482	8.392878
C	16.125982	10.476605	10.931604
C	14.345796	11.274910	6.009235
C	14.714116	15.466500	5.506698
C	20.021070	16.770912	10.017373
C	18.174770	13.008071	5.866819
C	18.668929	12.519927	4.496437
C	17.572958	11.964010	3.580917
C	18.098371	11.508619	2.218063
C	17.010387	10.951473	1.298779
C	17.540911	10.501514	-0.060993
Si	20.273383	13.832569	6.675342
C	20.137277	15.213271	5.395829
C	19.054637	16.099518	5.312343
C	19.006867	17.084336	4.325698
C	20.032979	17.187238	3.387225
C	21.123370	16.320070	3.460410
C	21.180600	15.356369	4.466768
C	21.804794	18.152253	6.827452
C	23.335733	18.223931	6.873482
C	23.822032	19.444878	6.076673
C	23.840535	18.232730	8.308392
C	24.600946	17.175450	8.858804
C	25.046724	17.244829	10.204599
C	24.721009	18.372154	10.963833
C	23.969262	19.413709	10.431432
C	23.535975	19.335214	9.115163
N	24.908445	16.002839	8.095037
Mg	23.731425	14.268365	8.387101
C	22.915000	14.062232	10.351806
C	21.729772	13.186189	10.767277

C	21.383025	13.267867	12.261389
C	20.201804	12.400011	12.697845
C	19.877522	12.507816	14.189269
C	18.698958	11.636918	14.619832
C	25.910447	16.151449	10.819530
C	27.406246	16.472990	10.667177
C	25.985725	16.009085	7.314081
C	26.744101	17.298079	7.059577
C	26.518754	14.876053	6.669400
C	26.171004	13.513736	6.744092
C	27.228368	12.580063	6.177633
N	25.063603	13.025991	7.305794
C	24.900538	11.602874	7.319865
C	25.400508	10.839001	8.400567
C	25.259847	9.446924	8.364334
C	24.643531	8.809947	7.297247
C	24.125421	9.571310	6.254534
C	24.227129	10.965817	6.244707
C	26.090018	11.467312	9.603526
C	27.552109	11.014125	9.736578
C	23.616627	11.762709	5.094952
C	22.449305	11.034930	4.416871
C	24.633927	12.163072	4.012314
C	25.328105	11.167082	10.902885
C	25.581839	15.869563	12.290567
H	21.707563	14.270742	7.219160
H	20.705366	12.454571	6.348583
H	15.268552	16.212479	10.573240
H	14.537233	14.872177	11.486347
H	16.096718	15.530109	11.979052
H	15.661882	12.857015	11.714024
H	17.042980	10.008853	11.308242
H	15.488220	10.711053	11.786509
H	15.624399	9.731214	10.309005
H	18.368163	15.629101	10.735672
H	17.203222	17.566753	11.903792
H	18.858823	17.274015	12.474884
H	18.522014	18.621938	11.384698
H	20.272191	17.784473	9.685970
H	20.664348	16.531840	10.871016
H	20.276330	16.080244	9.208441
H	18.265643	19.003663	9.136471
H	16.762685	19.619284	7.277111
H	15.235787	17.931190	6.318267
H	15.290305	14.326829	7.220810
H	13.106136	16.427653	7.604762
H	12.838752	14.694274	7.332614
H	13.641049	15.248585	8.813225
H	15.672762	15.464550	4.976467
H	14.116057	14.629752	5.128748
H	14.183667	16.387607	5.237752
H	14.859529	11.745825	8.029108
H	13.650236	9.842267	9.109578
H	12.584952	10.788031	8.056383
H	13.203389	9.219530	7.515408
H	14.133592	10.414136	5.364099
H	13.457331	11.918324	6.001182
H	15.173936	11.830433	5.557925
H	15.083500	8.490070	6.200166
H	17.044681	6.987820	6.284527

H	18.991663	7.587806	7.680649
H	19.214550	10.698939	9.743852
H	19.823809	7.696481	9.870549
H	20.409642	8.944165	10.979079
H	18.683924	8.534186	10.929840
H	20.559047	10.649319	7.625665
H	21.516909	10.137347	9.022833
H	20.900770	8.935163	7.883128
H	22.688956	15.104404	10.641633
H	23.775160	13.791858	10.989898
H	20.833877	13.462218	10.187624
H	21.923783	12.131125	10.515178
H	21.176801	14.318409	12.518948
H	22.273459	12.987612	12.845100
H	20.412508	11.347735	12.451336
H	19.308109	12.675136	12.115684
H	20.767903	12.233697	14.773149
H	19.666167	13.558218	14.437185
H	18.896527	10.576350	14.418942
H	18.490788	11.738272	15.691621
H	17.786358	11.911292	14.075605
H	26.821258	11.633464	5.820333
H	27.960089	12.347527	6.960651
H	27.772008	13.064193	5.361448
H	27.422387	15.071418	6.101998
H	26.275287	17.842695	6.231377
H	27.780184	17.095022	6.776735
H	26.736538	17.961896	7.926968
H	23.225250	12.691927	5.531405
H	25.380579	12.868417	4.382234
H	24.115787	12.642011	3.171764
H	25.155014	11.280020	3.621970
H	22.799156	10.200370	3.796238
H	21.916908	11.725169	3.753405
H	21.731279	10.640653	5.142831
H	23.626548	9.066490	5.432913
H	24.555616	7.726236	7.279553
H	25.651306	8.853575	9.187649
H	26.089119	12.553362	9.460165
H	27.618656	9.935329	9.923644
H	28.035544	11.526143	10.577530
H	28.131615	11.227340	8.832736
H	24.285980	11.491977	10.836282
H	25.792963	11.688373	11.748589
H	25.336198	10.093915	11.130338
H	25.717295	15.229206	10.260006
H	27.697690	16.563903	9.616467
H	28.017331	15.680057	11.116663
H	27.654289	17.416354	11.169834
H	25.880605	16.696386	12.946662
H	26.127643	14.980468	12.628295
H	24.512850	15.684317	12.432550
H	25.063349	18.437860	11.992980
H	23.727227	20.282159	11.039631
H	22.952059	20.151789	8.696099
H	23.724195	17.323769	6.384202
H	23.381139	20.372915	6.460547
H	23.525682	19.353073	5.024654
H	24.910366	19.559337	6.116548
H	21.433226	17.262990	7.343339

H	21.448229	18.113888	5.793042
H	21.356196	19.031186	7.307187
H	17.637246	12.110865	6.258238
H	17.384230	13.760572	5.677827
H	19.178037	13.338830	3.973128
H	17.076910	11.118152	4.079194
H	16.799050	12.732330	3.432031
H	18.596204	12.353645	1.718948
H	18.874659	10.742466	2.364727
H	16.233347	11.716003	1.153662
H	16.514362	10.105900	1.796870
H	18.011763	11.334364	-0.597858
H	16.738239	10.108391	-0.695780
H	18.294941	9.712137	0.048536
H	19.693967	14.041350	8.082205
H	18.237093	16.037692	6.026293
H	18.164261	17.770636	4.292752
H	19.988629	17.944894	2.608523
H	21.933377	16.398088	2.738902
H	22.051390	14.705164	4.524221
H	19.425152	11.737803	4.647846

199

[F3] scf done: -3873.495376 / Energies= -3871.637919 / Enthalpies= -3871.636975 / Free Energies= -3871.881773

C	-4.473978	2.667745	-0.364939
C	-3.697312	2.297691	0.761383
C	-2.859697	3.240213	1.403365
C	-2.796858	4.535786	0.878886
C	-3.542321	4.908002	-0.232200
C	-4.380027	3.979320	-0.839674
N	-3.717217	0.930557	1.190522
C	-4.405141	0.556812	2.268483
C	-4.493420	-0.769926	2.739443
C	-4.131457	-1.986601	2.134964
C	-4.621361	-3.229809	2.845842
C	-2.036504	2.905881	2.639622
C	-2.354924	3.851553	3.809124
C	-5.458397	1.697725	-1.004576
C	-6.860435	1.872700	-0.397322
Mg	-2.563934	-0.432339	0.199800
N	-3.454775	-2.097336	0.986165
C	-3.431318	-3.361644	0.308708
C	-2.258050	-4.152996	0.314771
C	-2.243486	-5.333816	-0.436440
C	-3.346224	-5.737100	-1.177667
C	-4.500480	-4.963004	-1.160244
C	-4.574375	-3.779075	-0.420759
C	-1.036859	-3.800756	1.151422
C	-0.938981	-4.710409	2.387094
C	-5.878964	-2.990131	-0.438429
C	-7.086622	-3.858786	-0.052689
C	0.268788	-3.867209	0.350538
C	-6.122571	-2.332630	-1.803858
C	-5.157517	1.576542	3.097512
C	-0.531453	2.923434	2.346963
C	-5.530725	1.805543	-2.532370
C	-2.034238	-0.663978	-2.060079
C	-1.896382	-1.072051	-3.537995
C	-3.081140	-1.837747	-4.130711
C	-2.853619	-2.245292	-5.587744

C	-4.033206	-2.998995	-6.203936
C	-3.796849	-3.400396	-7.658524
Si	-0.211788	0.050042	-1.405509
C	-0.071375	1.449758	-2.686754
C	-1.156091	2.230945	-3.112477
C	-1.005939	3.205373	-4.099555
C	0.235015	3.399503	-4.705362
C	1.328186	2.636167	-4.295058
C	1.176580	1.689681	-3.281963
C	0.925517	4.416009	-0.907545
C	2.455850	4.511102	-0.898469
C	2.897049	5.771585	-1.659568
C	3.003582	4.472583	0.519924
C	3.809920	3.415486	1.001820
C	4.300958	3.448370	2.333857
C	3.973536	4.537175	3.146533
C	3.175898	5.576861	2.681532
C	2.698693	5.535130	1.378978
N	4.125339	2.281217	0.186242
Mg	2.980324	0.496833	0.411673
C	2.284144	0.230529	2.425893
C	1.088167	-0.601796	2.898722
C	0.835864	-0.537391	4.412881
C	-0.381497	-1.324571	4.900515
C	-0.610916	-1.230593	6.410321
C	-1.834344	-2.009801	6.889199
C	5.215266	2.357144	2.875569
C	6.695970	2.720126	2.674723
C	5.185149	2.346417	-0.614673
C	5.889609	3.669872	-0.850856
C	5.751926	1.250571	-1.294652
C	5.476233	-0.129780	-1.230360
C	6.590608	-0.999427	-1.791620
N	4.395044	-0.681100	-0.678244
C	4.316171	-2.111643	-0.680725
C	3.723360	-2.781658	-1.784113
C	3.717007	-4.179921	-1.791657
C	4.251139	-4.917731	-0.740362
C	4.784983	-4.252782	0.353800
C	4.827896	-2.854836	0.409378
C	3.097418	-2.019363	-2.948491
C	1.963333	-2.795267	-3.630685
C	5.433281	-2.198114	1.641992
C	6.929362	-2.514968	1.792653
C	4.682965	-2.606679	2.918025
C	4.108799	-1.592628	-4.026637
C	4.954516	2.019507	4.348359
H	1.184200	0.532261	-0.692640
H	0.379689	-1.292005	-1.677658
H	-5.351902	2.496986	2.544104
H	-6.107460	1.159556	3.443915
H	-4.573004	1.839406	3.987120
H	-5.033732	-0.875896	3.674793
H	-3.977173	-4.090115	2.654265
H	-4.673048	-3.053922	3.923707
H	-5.629673	-3.494371	2.506863
H	-2.293411	1.889302	2.955230
H	-3.428303	3.898162	4.022831
H	-1.838496	3.515951	4.716049
H	-2.015536	4.872910	3.601022

H	-0.194730	3.917321	2.033270
H	0.035452	2.648350	3.242816
H	-0.263902	2.214132	1.557739
H	-2.148190	5.266587	1.354986
H	-3.476307	5.920996	-0.620956
H	-4.976451	4.281761	-1.696147
H	-5.132050	0.679234	-0.756743
H	-7.238208	2.885021	-0.585632
H	-7.566134	1.159026	-0.839809
H	-6.852748	1.713803	0.685572
H	-4.541882	1.730959	-2.998507
H	-6.155372	1.000147	-2.935341
H	-5.980250	2.751859	-2.855053
H	-5.803581	-2.184676	0.298642
H	-6.927620	-4.384653	0.894925
H	-7.983951	-3.236771	0.049165
H	-7.299786	-4.616343	-0.815864
H	-6.198895	-3.084285	-2.598593
H	-7.057660	-1.758855	-1.793450
H	-5.307563	-1.653316	-2.072339
H	-5.367118	-5.286710	-1.731660
H	-3.310698	-6.655855	-1.757811
H	-1.349263	-5.951547	-0.433133
H	-1.157679	-2.772865	1.515916
H	-0.838586	-5.761086	2.088395
H	-0.060414	-4.445046	2.986221
H	-1.821525	-4.627605	3.029824
H	0.217568	-3.263575	-0.560398
H	1.104090	-3.492990	0.951245
H	0.515921	-4.894264	0.058338
H	2.113038	1.274677	2.746985
H	3.164846	-0.082155	3.013957
H	0.170968	-0.271656	2.382369
H	1.215032	-1.659338	2.615021
H	0.725746	0.518231	4.705953
H	1.735351	-0.898387	4.935065
H	-0.269603	-2.383043	4.619833
H	-1.282838	-0.967240	4.377427
H	0.283962	-1.595549	6.934469
H	-0.716120	-0.173336	6.694096
H	-1.742326	-3.076882	6.650187
H	-1.971290	-1.923072	7.973662
H	-2.749612	-1.641769	6.407808
H	6.242257	-1.961954	-2.167511
H	7.319413	-1.203419	-0.998056
H	7.122893	-0.476758	-2.591467
H	6.639024	1.496534	-1.868317
H	5.361271	4.233019	-1.629028
H	6.916263	3.515288	-1.192363
H	5.904644	4.296334	0.044040
H	2.666318	-1.102968	-2.526425
H	4.824043	-0.854516	-3.658452
H	3.579659	-1.143619	-4.876564
H	4.668760	-2.458568	-4.401581
H	2.346474	-3.625911	-4.236725
H	1.419223	-2.129168	-4.309654
H	1.249492	-3.203527	-2.908124
H	3.282356	-4.706461	-2.636155
H	4.239777	-6.004677	-0.772495
H	5.189652	-4.828140	1.183528

H	5.331254	-1.113386	1.527170
H	7.094080	-3.588692	1.946071
H	7.345509	-1.988432	2.660261
H	7.503077	-2.216196	0.909777
H	3.615131	-2.384918	2.838513
H	5.078113	-2.063228	3.784789
H	4.794555	-3.679000	3.121070
H	5.019385	1.450334	2.292774
H	6.943707	2.848797	1.616835
H	7.342473	1.929222	3.075673
H	6.942143	3.653823	3.196277
H	5.259002	2.832415	5.019278
H	5.535930	1.134687	4.634187
H	3.897287	1.800861	4.526186
H	4.351695	4.573772	4.164541
H	2.933342	6.415134	3.330608
H	2.080381	6.350857	1.010566
H	2.843172	3.637591	-1.433873
H	2.447905	6.674873	-1.228783
H	2.574190	5.712383	-2.706031
H	3.983474	5.907980	-1.643450
H	0.582208	3.497049	-0.425426
H	0.544226	4.419992	-1.933867
H	0.475592	5.264119	-0.376096
H	-2.291704	-1.620628	-1.545852
H	-2.927522	-0.000733	-1.993534
H	-1.711942	-0.172616	-4.136299
H	-3.281143	-2.734923	-3.527003
H	-3.986373	-1.215623	-4.067937
H	-2.643692	-1.347380	-6.188364
H	-1.950818	-2.870819	-5.652568
H	-4.936015	-2.374540	-6.138238
H	-4.240853	-3.897310	-5.604831
H	-3.622414	-2.519956	-8.289295
H	-4.657004	-3.940052	-8.071370
H	-2.919146	-4.051756	-7.752119
H	-0.779497	0.228839	0.071808
H	-2.136717	2.093420	-2.662610
H	-1.859444	3.809642	-4.398401
H	0.351599	4.146662	-5.486792
H	2.301999	2.784924	-4.755872
H	2.048516	1.129597	-2.948329
H	-0.991780	-1.689137	-3.633186

199

[TS-F3F4] scf done: -3873.494407 / Energies= -3871.638248 / Enthalpies= -3871.637304 / Free Energies= -3871.881527

C	3.930277	-2.840244	-1.604064
C	4.495089	-2.041394	-0.574788
C	5.106569	-2.656149	0.544417
C	5.189495	-4.052483	0.587166
C	4.680168	-4.840484	-0.434730
C	4.048068	-4.230740	-1.512322
N	4.440698	-0.610653	-0.666280
C	5.439443	0.008454	-1.294574
C	6.603099	-0.774371	-1.884144
C	5.694969	-1.863032	1.703029
C	5.101932	-2.303161	3.049376
C	3.207325	-2.230854	-2.800673
C	4.139875	-1.904366	-3.978885
C	5.589486	1.405671	-1.420912

C	4.980963	2.472963	-0.732168
C	5.575838	3.839297	-1.017804
N	3.969539	2.347971	0.123411
Mg	2.980566	0.499684	0.430857
C	2.325421	0.159422	2.439949
C	1.250860	-0.831353	2.897032
C	1.018787	-0.849860	4.415592
C	-0.062787	-1.820007	4.892891
C	-0.263634	-1.822183	6.409665
C	-1.349353	-2.788682	6.879109
C	3.601634	3.472055	0.931062
C	2.703756	4.457552	0.458794
C	2.349293	5.511275	1.309342
C	2.864030	5.612166	2.594728
C	3.749278	4.641945	3.050669
C	4.130061	3.564766	2.245266
C	2.103050	4.417443	-0.936997
C	2.365962	5.708469	-1.727890
C	5.140157	2.555185	2.773921
C	4.930045	2.199459	4.250607
C	0.595761	4.140483	-0.884546
C	6.581143	3.042344	2.549286
C	7.227585	-1.964789	1.748934
C	2.061200	-3.111955	-3.314987
C	0.288576	1.216906	-2.923302
C	-0.685594	1.886492	-3.680203
C	-0.338527	2.772608	-4.701065
C	1.003728	2.991802	-5.006522
C	1.991507	2.335369	-4.271710
C	1.632910	1.477330	-3.232936
Si	-0.118367	-0.020263	-1.520673
C	-1.880140	-0.663358	-2.175725
C	-1.815469	-1.281609	-3.581494
C	-3.112051	-1.899059	-4.107618
C	-2.949931	-2.524632	-5.494572
C	-4.238288	-3.138193	-6.044828
C	-4.064174	-3.762149	-7.428110
Mg	-2.619410	-0.338448	0.308359
N	-3.667490	-1.978599	0.946844
C	-3.701472	-3.210535	0.211550
C	-2.596711	-4.093341	0.241983
C	-2.631604	-5.236049	-0.565671
C	-3.719768	-5.516743	-1.380928
C	-4.811531	-4.656009	-1.382775
C	-4.833826	-3.503762	-0.591412
C	-1.402567	-3.885327	1.162021
C	-0.066951	-3.919693	0.409110
C	-6.074304	-2.617851	-0.625309
C	-6.196770	-1.863798	-1.956073
N	-3.757746	1.045070	1.294063
C	-4.523724	0.664301	2.316406
C	-5.264175	1.697489	3.138761
C	-3.661357	2.433740	0.950032
C	-2.834281	3.302803	1.700156
C	-2.731079	4.636263	1.291272
C	-3.418812	5.112850	0.182335
C	-4.226815	4.248962	-0.546922
C	-4.361156	2.904561	-0.187256
C	-2.037262	2.837962	2.910956
C	-0.545805	2.705249	2.577053

C	-5.295313	2.002065	-0.981293
C	-5.229354	2.237913	-2.495085
C	-4.385565	-1.880510	2.072253
C	-4.972919	-3.126511	2.699528
C	-4.705994	-0.674947	2.720582
C	-6.744517	2.138408	-0.486345
C	-2.224824	3.756759	4.127919
C	-1.392647	-4.926631	2.293543
C	-7.361415	-3.411405	-0.351294
H	1.088317	0.555817	-0.627594
H	0.525743	-1.363288	-1.635118
H	-5.626966	2.521051	2.518345
H	-6.110793	1.244002	3.659580
H	-4.601273	2.133792	3.894405
H	-5.292641	-0.794768	3.625011
H	-4.340460	-4.000866	2.532740
H	-5.109761	-2.982734	3.774553
H	-5.955732	-3.349123	2.268543
H	-2.393127	1.841216	3.191866
H	-3.282289	3.924036	4.362807
H	-1.744255	3.315007	5.008498
H	-1.763395	4.737934	3.967412
H	-0.112590	3.668527	2.286320
H	0.008946	2.338544	3.447450
H	-0.375543	2.001489	1.755687
H	-2.093875	5.313316	1.854014
H	-3.324068	6.154246	-0.114610
H	-4.766210	4.627502	-1.410824
H	-4.994652	0.962650	-0.793466
H	-7.096040	3.170731	-0.603254
H	-7.413282	1.485462	-1.060354
H	-6.836941	1.869230	0.570628
H	-4.198076	2.220380	-2.864542
H	-5.795777	1.461319	-3.021744
H	-5.666939	3.202276	-2.778110
H	-5.979667	-1.863967	0.162301
H	-7.291130	-4.000640	0.569421
H	-8.213862	-2.728614	-0.253997
H	-7.591291	-4.105392	-1.168144
H	-6.285375	-2.557656	-2.800329
H	-7.086266	-1.221772	-1.953237
H	-5.321007	-1.233671	-2.139970
H	-5.669391	-4.886254	-2.010111
H	-3.723491	-6.408391	-2.002859
H	-1.789957	-5.923769	-0.545901
H	-1.500155	-2.898709	1.631363
H	-1.299104	-5.942285	1.890233
H	-0.543200	-4.750983	2.962948
H	-2.307150	-4.889483	2.894838
H	-0.042212	-3.192087	-0.407763
H	0.759148	-3.685471	1.088713
H	0.130250	-4.910104	-0.017813
H	2.028854	1.167984	2.778971
H	3.245083	-0.051610	3.013596
H	0.292408	-0.605237	2.400413
H	1.508728	-1.853406	2.575136
H	0.763648	0.168696	4.746973
H	1.970307	-1.094300	4.912336
H	0.190894	-2.839298	4.563204
H	-1.018609	-1.572862	4.404105

H	0.688322	-2.075335	6.897892
H	-0.511735	-0.803793	6.742551
H	-1.113745	-3.820512	6.589724
H	-1.465548	-2.767904	7.969231
H	-2.321615	-2.536565	6.436533
H	6.366526	-1.818124	-2.090852
H	7.439814	-0.757492	-1.175676
H	6.956547	-0.299588	-2.804480
H	6.423004	1.701863	-2.049279
H	4.949080	4.375435	-1.739873
H	6.577554	3.752267	-1.445876
H	5.627862	4.458708	-0.118777
H	2.770359	-1.285356	-2.459906
H	4.865108	-1.126672	-3.731605
H	3.551694	-1.546488	-4.832902
H	4.690963	-2.797424	-4.299772
H	2.434120	-3.992583	-3.852915
H	1.447949	-2.541493	-4.021384
H	1.413888	-3.458300	-2.503273
H	3.633419	-4.852027	-2.300815
H	4.764654	-5.923718	-0.389409
H	5.671635	-4.527976	1.438097
H	5.434725	-0.809487	1.553594
H	7.549041	-3.001586	1.908082
H	7.627421	-1.361334	2.573132
H	7.688777	-1.615070	0.820313
H	4.009095	-2.262134	3.035152
H	5.456630	-1.647472	3.853612
H	5.400544	-3.327262	3.305253
H	5.013746	1.634200	2.193916
H	6.796741	3.197493	1.487754
H	7.299661	2.307007	2.932845
H	6.757498	3.991031	3.071959
H	5.165246	3.039745	4.915456
H	5.595171	1.374077	4.531723
H	3.900123	1.883749	4.442795
H	4.155730	4.725292	4.055053
H	2.581878	6.442901	3.237503
H	1.659932	6.271682	0.948666
H	2.567548	3.588959	-1.480571
H	1.820223	6.559372	-1.301831
H	2.028739	5.588217	-2.764191
H	3.428138	5.976129	-1.741947
H	0.386801	3.189030	-0.386650
H	0.177955	4.091184	-1.895803
H	0.066815	4.928907	-0.335617
H	-2.190034	-1.499625	-1.506366
H	-2.678377	0.109600	-2.148284
H	-1.467883	-0.518856	-4.288280
H	-3.469823	-2.662656	-3.402798
H	-3.893468	-1.125424	-4.150151
H	-2.582219	-1.762405	-6.197971
H	-2.169851	-3.299462	-5.453006
H	-5.018783	-2.364651	-6.087511
H	-4.603586	-3.900511	-5.341729
H	-3.732878	-3.015549	-8.160435
H	-5.001894	-4.195016	-7.795433
H	-3.312961	-4.561396	-7.410154
H	-0.876508	0.177087	-0.022716
H	-1.740956	1.724361	-3.472126

H	-1.116458	3.287666	-5.260437
H	1.278902	3.672286	-5.808882
H	3.042466	2.500340	-4.497274
H	2.423711	1.007043	-2.649885
H	-1.033425	-2.053834	-3.568162

199

[F4] scf done: -3873.562958 / Energies= -3871.705879 / Enthalpies= -3871.704935 / Free Energies= -3871.977887

C	-4.009405	2.956418	1.656821
C	-4.023925	1.624011	2.130182
C	-2.928501	1.107299	2.860122
C	-1.838196	1.945149	3.113229
C	-1.816146	3.259893	2.661326
C	-2.894934	3.753195	1.936126
N	-5.115303	0.763026	1.785563
C	-6.165086	0.660443	2.600005
C	-7.283590	-0.161229	2.356682
C	-7.558421	-1.028618	1.281522
C	-8.871223	-1.777503	1.337263
C	-2.890541	-0.337975	3.336467
C	-2.562255	-0.462491	4.830521
C	-5.151114	3.525470	0.826452
C	-5.687448	4.850577	1.385030
Mg	-4.943235	-0.274080	0.030169
N	-6.744735	-1.221684	0.243626
C	-7.114505	-2.132925	-0.796581
C	-6.703191	-3.483771	-0.721614
C	-7.001623	-4.334569	-1.790340
C	-7.686239	-3.876750	-2.910188
C	-8.079153	-2.545022	-2.975726
C	-7.805138	-1.653824	-1.934019
C	-5.917584	-4.020270	0.466736
C	-6.551819	-5.277971	1.076531
C	-8.219703	-0.195251	-2.065806
C	-9.706664	-0.030439	-2.408619
C	-4.454669	-4.286536	0.077289
C	-7.342652	0.529130	-3.099684
C	-6.185193	1.464050	3.881026
C	-1.901656	-1.160557	2.494786
C	-4.727563	3.688847	-0.642207
C	-2.215152	-0.675920	-4.835815
C	-1.911182	-2.136760	-5.194855
C	-3.160993	-3.020335	-5.217790
C	-2.869287	-4.484787	-5.548155
C	-4.122153	-5.361523	-5.583471
C	-3.823765	-6.827250	-5.892867
Si	-0.712656	0.464661	-4.834144
C	0.093870	0.611431	-6.528095
C	-0.384649	1.529246	-7.478702
C	0.183604	1.618715	-8.748263
C	1.249400	0.788675	-9.095047
C	1.743438	-0.126862	-8.166990
C	1.170945	-0.211174	-6.898130
C	1.498391	1.849125	0.097652
C	2.381164	2.955780	-0.501186
C	1.659812	3.650221	-1.663849
C	2.834258	3.926897	0.579939
C	4.148117	3.897334	1.103847
C	4.531639	4.786839	2.134390
C	3.585951	5.693927	2.622341

C	2.292781	5.733393	2.114565
C	1.926704	4.853563	1.101906
N	5.066445	2.897723	0.650722
Mg	5.032840	1.103043	1.661280
C	3.987766	0.527627	3.417391
C	3.776240	-0.962372	3.723494
C	3.060156	-1.240966	5.051583
C	2.854690	-2.727417	5.348559
C	2.141929	-2.999972	6.674460
C	1.944963	-4.487539	6.960585
C	5.930627	4.772514	2.734192
C	6.619961	6.141383	2.638952
C	5.861811	3.149627	-0.387101
C	5.785163	4.498180	-1.068581
C	6.788571	2.231715	-0.919841
C	7.060356	0.899618	-0.554610
C	8.115406	0.182526	-1.367787
N	6.451152	0.250209	0.437191
C	6.773645	-1.118879	0.700464
C	6.049945	-2.146230	0.050122
C	6.309955	-3.474057	0.401779
C	7.257182	-3.795045	1.367154
C	7.965630	-2.777891	1.996041
C	7.744727	-1.433976	1.679376
C	4.983257	-1.843040	-0.992924
C	3.577054	-2.124996	-0.441655
C	8.534131	-0.353515	2.404953
C	10.050690	-0.572676	2.310772
C	8.095131	-0.238129	3.873097
C	5.206962	-2.608326	-2.304841
C	5.905659	4.283565	4.190559
H	-1.123044	1.828218	-4.391940
H	0.314235	-0.051942	-3.882750
H	-6.075190	2.533727	3.672549
H	-7.113383	1.308479	4.435062
H	-5.343605	1.185940	4.525265
H	-8.052137	-0.123284	3.120197
H	-8.701137	-2.859853	1.341064
H	-9.444855	-1.512178	2.227905
H	-9.478905	-1.562460	0.451464
H	-3.888258	-0.765173	3.185824
H	-3.252851	0.124436	5.446692
H	-2.631836	-1.509772	5.147964
H	-1.545203	-0.120743	5.053886
H	-0.878588	-0.784576	2.613966
H	-1.911496	-2.213473	2.802340
H	-2.142411	-1.113352	1.425799
H	-0.987299	1.560197	3.669908
H	-0.957164	3.892780	2.869142
H	-2.871020	4.778916	1.575294
H	-5.975127	2.803445	0.855072
H	-4.938242	5.649047	1.329652
H	-6.560023	5.180308	0.808419
H	-5.991589	4.753392	2.433269
H	-4.373267	2.743175	-1.069653
H	-5.566633	4.047590	-1.251169
H	-3.909729	4.413634	-0.736015
H	-8.054299	0.286980	-1.095767
H	-10.348426	-0.542232	-1.682615
H	-9.980172	1.031495	-2.412302

H	-9.942433	-0.432113	-3.400938
H	-7.477917	0.098013	-4.099117
H	-7.604376	1.593163	-3.153679
H	-6.277158	0.449960	-2.853252
H	-8.606390	-2.187471	-3.857144
H	-7.908394	-4.554125	-3.730865
H	-6.687974	-5.374944	-1.745358
H	-5.917272	-3.246915	1.242930
H	-6.530534	-6.123552	0.379129
H	-6.002818	-5.581623	1.975887
H	-7.596703	-5.107526	1.359531
H	-3.973476	-3.387544	-0.326003
H	-3.876484	-4.624324	0.946178
H	-4.389615	-5.064169	-0.693371
H	3.006875	1.033813	3.409876
H	4.517842	0.983812	4.273346
H	3.196528	-1.436712	2.916015
H	4.741496	-1.491694	3.733543
H	2.084769	-0.730209	5.045792
H	3.635693	-0.781166	5.869971
H	3.831385	-3.235204	5.352368
H	2.280268	-3.186255	4.528952
H	2.715153	-2.542169	7.493599
H	1.165311	-2.494690	6.670366
H	2.907342	-5.012884	7.002104
H	1.435081	-4.651333	7.917392
H	1.343743	-4.965456	6.176884
H	7.684285	-0.677109	-1.892911
H	8.904746	-0.213453	-0.719803
H	8.568918	0.847235	-2.106241
H	7.370602	2.603798	-1.755165
H	4.788044	4.658901	-1.494083
H	6.521098	4.581345	-1.871251
H	5.951976	5.310408	-0.353316
H	5.037175	-0.773791	-1.225213
H	6.207264	-2.427490	-2.714074
H	4.472059	-2.295548	-3.056123
H	5.095775	-3.690528	-2.168616
H	3.456588	-3.186858	-0.194793
H	2.809784	-1.860684	-1.179462
H	3.379891	-1.554225	0.473043
H	5.755639	-4.271939	-0.087058
H	7.442187	-4.833976	1.628657
H	8.705955	-3.031489	2.751373
H	8.312842	0.602645	1.917237
H	10.362562	-1.485395	2.832118
H	10.585679	0.266839	2.770887
H	10.382954	-0.655621	1.269800
H	7.021425	-0.034623	3.958696
H	8.637635	0.571246	4.377401
H	8.296208	-1.168126	4.418709
H	6.532393	4.061941	2.156548
H	6.657356	6.505676	1.606265
H	7.649365	6.076837	3.011696
H	6.099956	6.898239	3.238003
H	5.326699	4.964574	4.826194
H	6.923027	4.229366	4.597735
H	5.450062	3.290653	4.273193
H	3.868893	6.382064	3.415892
H	1.573093	6.448440	2.505924

H	0.913664	4.883817	0.708412
H	3.275631	2.475938	-0.913859
H	0.702805	4.083424	-1.350872
H	1.443259	2.927717	-2.459023
H	2.266992	4.455895	-2.092279
H	2.016256	1.309493	0.900458
H	1.210272	1.122686	-0.671641
H	0.579852	2.265676	0.527420
H	-2.679363	-0.620752	-3.839837
H	-2.955701	-0.265308	-5.538634
H	-1.417354	-2.185857	-6.176166
H	-3.663840	-2.958315	-4.241962
H	-3.873068	-2.615693	-5.952827
H	-2.356167	-4.546540	-6.519881
H	-2.164152	-4.891326	-4.807212
H	-4.820046	-4.963022	-6.333945
H	-4.641760	-5.285965	-4.617870
H	-3.330214	-6.934104	-6.866918
H	-4.739898	-7.429000	-5.917163
H	-3.158532	-7.264398	-5.137730
H	-3.742100	-0.334516	-1.205178
H	-1.210258	2.191917	-7.223284
H	-0.201911	2.338830	-9.466318
H	1.696043	0.858463	-10.084042
H	2.577740	-0.773039	-8.430034
H	1.574664	-0.925908	-6.183038
H	-1.193224	-2.549938	-4.471056

References

1. S. J. Bonyhady, C. Jones, S. Nembenma, A. Stasch, A. J. Edwards, G. J. McIntyre, *Chem. Eur. J.*, **2010**, *16*, 938.
2. Dolomanov, O. V., Bourhis, L. J., Gildea, R. J., Howard, J. A. K. & Puschmann, H., *J. Appl. Cryst.*, **2009**, *42*, 339.
3. Bourhis, L. J., Dolomanov, O. V., Gildea, R. J., Howard, J. A. K. & Puschmann, H., *Acta Cryst.*, **2015**, *A71*, 59.
4. Sheldrick, G. M., *Acta Cryst.*, **2015**, *C71*, 3.
5. 5. Gaussian09, revision D.01. Frisch, M. J.; Trucks, G. W.; Schlegel, H. B.; Scuseria, G. E.; Robb, M. A.; Cheeseman, J. R.; Scalmani, G.; Barone, V.; Mennucci, B.; Petersson, G. A.; Nakatsuji, H.; Caricato, M.; Li, X.; Hratchian, H. P.; Izmaylov, A. F.; Bloino, J.; Zheng, G.; Sonnenberg, J. L.; Hada, M.; Ehara, M.; Toyota, K.; Fukuda, R.; Hasegawa, J.; Ishida, M.; Nakajima, T.; Honda, Y.; Kitao, O.; Nakai, H.; Vreven, T.; Montgomery, Jr., J. A.; Peralta, J. E.; Ogliaro, F.; Bearpark, M.; Heyd, J. J.; Brothers, E.; Kudin, K. N.; Staroverov, V. N.; Keith, T.; Kobayashi, R.; Normand, J.; Raghavachari, K.; Rendell, A.; Burant, J. C.; Iyengar, S. S.; Tomasi, J.; Cossi, M.; Rega, N.; Millam, J. M.; Klene, M.; Knox, J. E.; Cross, J. B.; Bakken, V.; Adamo, C.; Jaramillo, J.; Gomperts, R.; Stratmann, R. E.; Yazyev, O.; Austin, A. J.; Cammi, R.; Pomelli, C.; Ochterski, J. W.; Martin, R. L.; Morokuma, K.; Zakrzewski, V. G.; Voth, G. A.; Salvador, P.; Dannenberg, J. J.; Dapprich, S.; Daniels, A. D.; Farkas, O.; Foresman, J. B.; Ortiz, J. V.; Cioslowski, J.; Fox, D. J.; Gaussian, Inc., Wallingford CT, **2013**.
6. (a) J. P. Perdew, J. A. Chevary, S. H. Vosko, K. A. Jackson, M. R. Pederson, D. J. Singh, C. Fiolhais, *Phys. Rev. B* **1992**, *46*, 6671; (b) A. D. Becke, *J. Chem. Phys.* **1993**, *98*, 5648.
